# A CRISPR-based ‘dam-break drainage’ sensing architecture for rapid, affordable and ultrasensitive pathogen detection

**DOI:** 10.1038/s41467-026-77042-w

**Published:** 2026-08-22

**Authors:** Kai Liu, Xiang Li, Meng Zhao, Zhuo Yang, Jingshuai Wen, Jinpeng Liu, Haoli Zhang, Yuecan Zhao, Yang Sun, Zhengping Li

**Affiliations:** Beijing Key Laboratory for Bioengineering and Sensing Technology, School of Chemistry and Biological Engineering, University of Science and Technology Beijing, 30 Xueyuan Road, Haidian District, Beijing, PR China

**Keywords:** Assay systems, Biochemical assays, CRISPR-Cas systems, Infectious-disease diagnostics, Diagnostic devices

## Abstract

Nucleic acid testing is important for infectious-disease diagnosis, but combining high sensitivity with accessible testing remains challenging. Here we show a chip-based visual detection strategy for one or two viral RNA targets that involves only sample loading, without further instrumentation or manual actuation. Target-activated CRISPR-Cas13a alters the wettability of a molecular barrier, causing liquid to cross a preset threshold and generate a dam-break drainage signal. The chip detects a single target at 10 aM within 2 min and two targets at 100 aM within 5 min, with costs of $0.20 and $0.30 per test, respectively. We further establish a practical clinical-sample workflow combining room-temperature lysis with RNase inhibition and validate the platform across 40 nasal-swab samples tested for SARS-CoV-2, influenza A virus and influenza B virus, with results concordant with RT–PCR. This threshold-based fluidic strategy provides a route towards rapid, low-cost and instrument-free molecular testing outside centralized laboratories.

## Abstract (web summary)

Nucleic acid testing is central to infectious-disease diagnosis, and methods which combine high sensitivity with operational simplicity and ease of interpretation are particularly desirable. Here the authors present their CRISPR-based ‘dam-break drainage’ chip, where a positive result is clearly visualised via the release of test liquid from a sample reservoir.

## Introduction

Infectious pathogens have persisted throughout human history, continuously posing serious threats to public health and social stability^1,2^. Some pathogens display periodic outbreaks—such as influenza A (IAV) and B (IBV) viruses–that cause seasonal epidemics and significant healthcare burdens^3^. Meanwhile, newly emerging and highly pathogenic agents have repeatedly triggered sudden global health crises, including severe acute respiratory syndrome (SARS), Middle East respiratory syndrome (MERS), Ebola virus disease, coronavirus disease 2019 (COVID-19) and more recently, chikungunya virus (CHIKV) outbreaks^4–9^. These events highlight that the threat of infectious diseases is not incidental but an enduring challenge. Consequently, the development of affordable, rapid, accurate and easily deployable diagnostic technologies is seriously needed for ‌infectious-disease prevention and control-particularly in resource-limited and low-income regions where such capabilities hold irreplaceable strategic value.

The COVID-19 pandemic has highlighted a persistent gap in diagnostic capabilities–lacking technologies that can simultaneously perform highly sensitivity, affordability, rapid turnaround and operational simplicity without reliance on specialized equipment^10^. Lateral flow immunoassays (LFAs)^11^ offer simple, low-cost, rapid and instrument-free operation, yet their limited sensitivity restricts their use for early-stage pathogen screening. In contrast, nucleic acid amplification techniques represented by reverse transcription-polymerase chain reaction (RT–PCR) provide high sensitivity and flexible probe redesign for emerging variants^12^, but require sophisticated temperature-control instruments, trained personnel and long assay times, making them unsuitable for low-cost, field-deployable and rapid testing. Alternative approaches based on SHERLOCK^13^, DETECTR^14,15^, electrochemical^16,17^, colorimetric^18,19^ and optical signal transduction^20,21^ have further diversified the detection landscape, yet most still depend on nucleic acid pre-amplification or external readout instruments. Consequently, achieving high sensitivity, affordability, operational simplicity and rapidity within a single diagnostic platform remains a formidable challenge.

At the mechanistic level, this persistent trade-off arises because molecular recognition events in most highly sensitive assays do not directly produce macroscopic readouts. Instead, they must be transduced through instrument-dependent optical or electrical excitation and signal-processing steps, resulting in an indirect “microscopic molecular interaction–instrument modulation–macroscopic signal readout” pathway that fundamentally constrains the simultaneous achievement of sensitivity, affordability, rapidity and instrument-free deployment. In our previous study, we established a direct signal-transduction route in which CRISPR-Cas mediated target recognition drives interfacial wettability switching, converting microscopic molecular recognition events into macroscopic visible liquid motion readouts^22^. This route was initially implemented in a tube-based sensor, where signal readout was achieved by simply observing whether the liquid droplet fell upon tube inversion. That work served as a proof-of-concept platform, demonstrating that a direct coupling between molecular recognition and visual fluidic output could, in principle, bypass the conventional indirect instrument-dependent signal transduction chain.

However, conceptual feasibility alone does not suffice to establish a practical sensing methodology. The initial tube-based proof-of-concept platform still retained two intrinsic methodological limitations that restricted further development of this direct signal-transduction route toward a standardized and deployable diagnostic format. First, signal triggering in the tube format remained closely coupled to manual flipping, making the readout dependent on operational factors such as flipping angle, speed and consistency, and therefore difficult to standardize for robust use. Second, although the tube system effectively demonstrated binary yes/no visual readout, it did not provide a structured solution for organized parallel readout under shared-system conditions. When multiple targets need to be detected within a common fluidic environment, how to maintain signal independence, avoid threshold perturbation and preserve readout distinguishability remain unresolved.

Here, we address these methodological challenges by developing a chip-based dam-break drainage sensing architecture that enables fixed-boundary, steady-state triggering together with organized parallel visual readout in a shared system. The architecture is deliberately engineered to operate in a threshold-confined fluidic state, in which the assay liquid in reservoir is maintained near a critical transition point prior to target introduction. Under this condition, target-activated Cas13a generates amplified molecular perturbations through collateral cleavage of tri-block probes, whereas the threshold-confined fluidic architecture converts the resulting wettability perturbation into a macroscopic dam-break drainage event through threshold-triggered fluidic amplification. Specifically, we construct a reservoir–dam–channel–collector geometry in which signal triggering is governed by boundary conditions and interfacial states following one-step sample loading, without requiring any subsequent manual actuation. To further support shared-system multi-target detection, we integrate a multi-dam layout, anchored-and-releasable crRNA strategies and a directional self-replenishment module, thereby enabling independent triggering and distinguishable visual outputs of two pathogen targets on a single chip. Under these conditions, the single-plex chip achieved a detection limit of 10 aM within 2 min at a cost below $0.2 per test, while the duplex chip simultaneously detected Influenza A virus and Influenza B virus with a sensitivity of 100 aM for both targets within 5 min at a cost below $0.3 per test. We further establish a practical nasal-swab workflow based on room-temperature lysis and RNase inhibition and validate the platform using 40 clinical samples tested for SARS-CoV-2, influenza A virus and influenza B virus, obtaining complete agreement with RT–PCR. Together, these results demonstrate that coupling CRISPR-Cas-mediated molecular amplification with threshold-triggered fluidic amplification establishes a dual-stage signal amplification framework, enabling localized molecular events to be converted into robust macroscopic visual outputs. Combined with innovations in triggering control and shared-system readout organization, this framework advances CRISPR-Cas-mediated wettability-switching detection beyond conceptual validation toward a standardized, deployable and instrument-free sensing methodology.

## Results

### Architecture and working principle of the single-plex chip

**Fig. 1:**
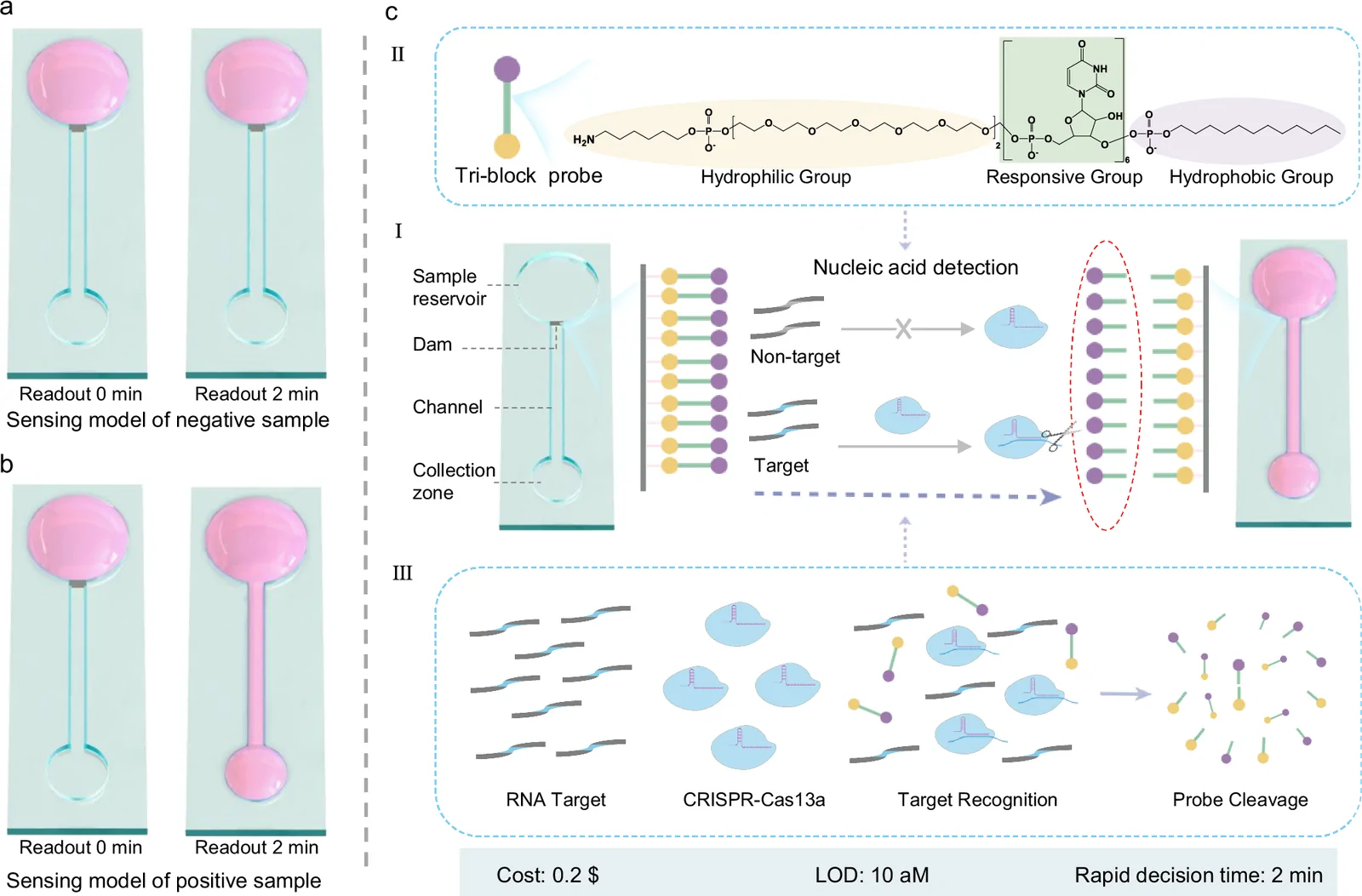
Working flow of the single-plex chip. **a** Negative readout of the single-plex chip: the test liquid remains confined in the sample reservoir within the optimized 2 min decision time. **b** Positive readout of the single-plex chip: the liquid breaches the hydrophobic dam and undergoes a visible dam-break drainage event within the optimized 2 min decision time. **c** Chip structure and sensing pathway of the single-plex chip (**cⅠ**); Architecture of the tri-block probe, consisting of a proximal hydrophilic moiety, a central responsive segment and a distal hydrophobic tail (**cⅡ**). Mechanism of target RNA-induced tri-block probe cleavage by CRISPR-Cas13a mediation (**cⅢ**). Note: Under the validated performance of single-plex chip, both the blank and negative samples remained confined throughout a 10 min observation, while all positive samples with different concentration consistently trigger the dam-break drainage event within 2 min. Thus, 2 min is defined as the rapid decision time.

As shown in Fig. 1, the developed single-plex chip is capable of instrument-free, visual nucleic acid detection of pathogens at a cost $ 0.2, with a limit of detection down to 10 aM and a 2 min rapid decision time. The detection model of the chip operates as follows: when the sample is negative, the liquid is confined within the sample reservoir (Fig. 1a). When the sample is positive, the liquid breaches the dam and flows along the channel (Fig. 1b). To achieve this behavior, the chip was structurally divided into four functional zones–sample reservoir, dam, channel and collection zone (Fig. 1cⅠ). At the dam region, we immobilized a tri-block probe composed of a hydrophilic group at the proximal end, a responsive spacer in the middle and a hydrophobic tail at the distal end (Fig. 1cⅡ). The immobilized tri-block probes render the overall dam surface hydrophobic, thereby increasing the wetting barrier for forward spreading of the liquid and preventing spontaneous flow beyond the sample reservoir. Notably, hydrophobic dam does not imply that it is inaccessible to the aqueous phase, rather, the loaded sample remains in direct contact with the dam surface and forms a confined liquid–dam interface. As validated in Supplementary Fig. 1, the CRISPR-Cas13a system can function as a target-activated mediator to efficiently cleave the responsive spacer of surface-immobilized tri-block probes (Supplementary Table 1), thereby inducing a hydrophobic-to-hydrophilic wettability transition. Owing to the programmability of crRNA, Cas13a′s RNase activity can be specifically activated by the target nucleic acid of any pathogen and a single activated Cas13a is able to cleave thousands of single-stranded RNA (ssRNA) substrates^23–25^ (Fig. 1cⅢ). Accordingly, we designed the responsive spacer as an ssRNA consisting of six uridine residues (6U), while the proximal hydrophilic group was formed by two PEG_6_ chains and the distal hydrophobic group featured a C_12_H_25_ tail (Fig. 1cⅡ). To examine whether the central RNA-responsive segment in the tri-block probe might be less accessible to Cas13a due to the flanking hydrophobic module and possible steric hindrance, we performed parallel control experiment while keeping all other components of the CRISPR-Cas13a system unchanged. One group used the conventional FAM-6U-BHQ fluorescent reporter, whereas another one used the FAM-(PEG_6_)_2_−6U-(CH_2_)_12_-BHQ fluorescent reporter. The two groups exhibited nearly identical fluorescence kinetic profiles, indicating that the (PEG_6_)_2_−6U-(CH_2_)_12_H tri-block architecture itself does not impair Cas13a access to the central RNA-responsive segment (Supplementary Fig. 2). As shown in Fig. 1cⅠ, upon target recognition, CRISPR-Cas13a-mediated cleavage of the middle ssRNA responsive group disrupts the interfacial hydrophobicity of the molecular dam, allowing the liquid to breach the dam and flow along the channel, thereby producing a dam-break drainage event. Conversely, in the absence of target nucleic acid, the Cas13a protein remains inactive, preserving the integrity of the tri-block probe and maintaining liquid confinement.

### Engineering a threshold-confined droplet for chip-level signal amplification

**Fig. 2:**
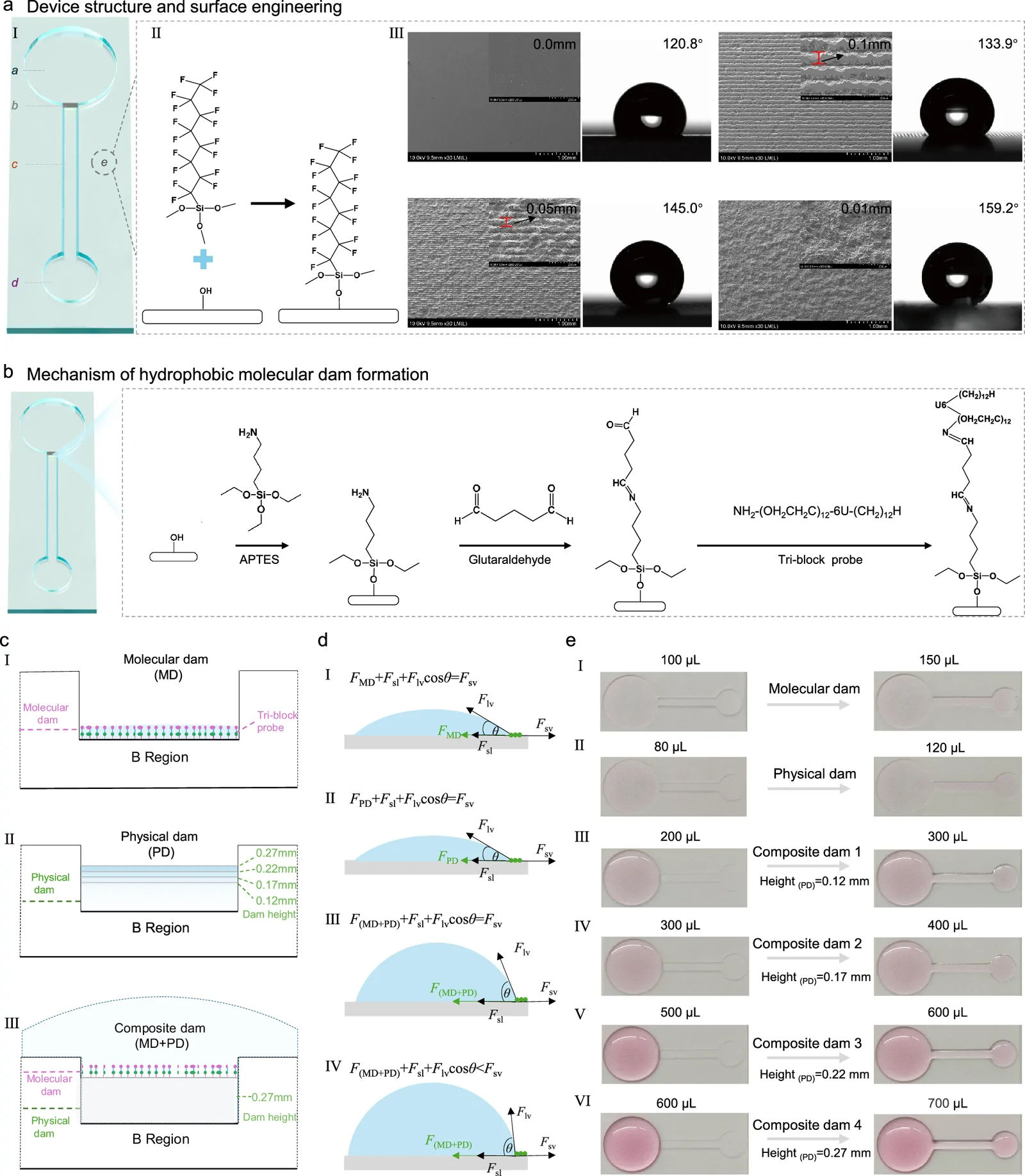
Design and optimization of the chip structure. **a** Chip structure and surface engineering. Schematic of the chip layout (**aⅠ**), comprising five functional zones: sample reservoir (A), dam (B), channel (C), collection zone (D) and exterior surface (E). Hydrophobic treatment for the E region using FAS-17 (**aⅡ**). Hydrophobic degree of the E region with different surface structure under identical FAS-17 modification (**aⅢ**). **b** Mechanism of immobilizing tri-block probe to form hydrophobic dam. Schematic illustrating the covalent conjugation of the aminated tri-block probe onto the APTES-functionalized molecular dam (B region) via glutaraldehyde crosslinking. **c** Dam design. Alone molecular dam formed by tri-block probe (**cⅠ**); Alone physical dam formed by raised chip substrate with different height (**cⅡ**); Composite dam integrating both molecular dam and physical dam (**cⅢ**). **d** Force profiles of liquid in A region for distinct B region designs: molecular dam alone (**dⅠ**), physical dams with varying heights (**dⅡ**), composite dam (**dⅢ**) and force evolution upon increasing liquid volume (**dⅣ**). **e** Influence of B region structures on liquid threshold volume in A region: liquid threshold volume under molecular dam alone (**eⅠ**) or physical dam alone (**eⅡ**); Evolution of liquid threshold volume as the height of the composite dam increases from 0.12 mm to 0.27 mm (**eⅢ**–**Ⅵ**).

Although CRISPR-Cas13a collateral cleavage provides molecular signal amplification, this amplification alone is insufficient to support highly sensitive visual detection given its pM-level standalone sensitivity^26^. We therefore sought to introduce an additional chip-level amplification strategy by regulating the threshold state of the assay liquid. Specifically, we aimed to create a threshold-confined droplet, namely an oversized droplet that could remain stably restricted in the sample reservoir but was maintained close to the drainage threshold. In this threshold state, the forward spreading tendency of the oversized droplet is nearly balanced by the wetting barrier generated at the hydrophobic dam interface: the barrier is sufficient to prevent spontaneous overflow in the absence of target, but small enough that limited target-induced degradation of the tri-block probes can easily break the balance, reduce the local wetting barrier and trigger a clear dam-break drainage readout. To achieve this, both the E region and the B region were designed to be hydrophobic, so that the oversized droplet could be effectively confined without premature overflow or collapse beyond the A region. The hydrophobic E region was designed to prevent lateral collapse of the oversized droplet, whereas the hydrophobic B region served as the tunable molecular dam that directly defined the drainage threshold. The process for E region hydrophobic modification began with a laser micromachining to create the micro-nanostructured surface, followed by chemical modification with fluoroalkyl silane (FAS-17) to introduce hydrophobic groups^27,28^ (Fig. 2aⅡ). As shown in Fig. 2aⅠ, Ⅲ, the obtained E region displayed a superhydrophobic contact angle of 159.2° under a laser matrix scanning interval of 0.01 mm (Laser power and scanning speed are provided in Supplementary Table 2). For the B region, hydrophobic modification was realized by covalently anchoring amine-modified tri-block probes onto the amine-modified B region via glutaraldehyde crosslinking (Fig. 2b). The surface amine modification of B region was performed by 3-Aminopropyltriethoxysilane (APTES)^29^. All other regions remained untreated and hydrophilic, facilitating liquid loading in A region and promoting dam-break drainage as soon as the liquid exceeds A region and contacts C region (Supplementary Fig. 3). Then, we optimized tri-block probe concentration to improve dam′s containment efficacy, with the fixed time and concentration condition of APTES, glutaraldehyde and initial 90 µm molecular dam width. As a result, modification with 1 µM tri-block probe in 60 min allowed the B region to completely prevent leakage of 100 µL liquid from the A region, with 100% success across 12 chips (Fig. 2eⅠ and Supplementary Fig. 4). However, although 100 µL could still be held at the threshold state, this retained volume was too small to produce a sufficiently sustained and visible dam-break drainage event after triggering. In other words, the liquid was exhausted before a clear macroscopic dam-break drainage event could fully develop. Under this condition, the liquid in A region experiences the following forces at the interface of B region (Fig. 2dⅠ):

1 $${F}_{{{\rm{MD}}}}+{F}_{{{\rm{sl}}}}+{F}_{{{\rm{lv}}}}\cos \theta={F}_{{{\rm{sv}}}}$$

Here, *F*_MD_ denotes the resistance exerted by the molecular dam; *F*_sl_ is the solid-liquid interfacial force; *F*_lv_ represents the liquid-air interfacial force; *F*_sv_ represents the solid-air interfacial force and *θ* is the contact angle between the liquid and the dam surface. When the volume in region A exceeds 100 µL, the contact angle (*θ*) between the liquid and the dam surface increases, leading to a decrease in *F*_lv_cos*θ*. This shifts the force balance to:

2 $${F}_{{{\rm{MD}}}}+{F}_{{{\rm{sl}}}}+{F}_{{{\rm{lv}}}}\cos \theta < {F}_{{{\rm{sv}}}}$$

Consequently, the liquid in A region overflows into C and D regions (Fig. 2eⅠ, right picture). Similarly, a standalone physical dam also fails to establish a threshold state to generate a sufficiently continuous and obvious dam-break drainage event readout and hard to perform sensing (Fig. 2cⅡ, eⅡ). Specifically, when the liquid volume in A region was 80 μL, the liquid in A region was subjected to forces as depicted in Fig. 2 dⅡ:

3 $${F}_{{{\rm{PD}}}}+{F}_{{{\rm{sl}}}}+{F}_{{{\rm{lv}}}}\cos \theta={F}_{{{\rm{sv}}}}$$

where *F*_PD_ represents the resistance provided by a standalone physical dam. Under this condition, the liquid remained stably confined within A region. However, when the liquid volume exceeded 80 μL, the force balance in A region changed to:

4 $${F}_{{{\rm{PD}}}}+{F}_{{{\rm{sl}}}}+{F}_{{{\rm{lv}}}}\cos \theta < {F}_{{{\rm{sv}}}}$$

As a result, the liquid from A region spilled over into C and D regions (Fig. 2eⅡ). Together, these analyses indicate that Fig. 2d establishes a volume-dependent force balance model for threshold state regulation: as the retained liquid volume increases, the contact angle *θ* correspondingly increases, the *F*_lv_cos*θ* decreases, and the original balance is progressively destabilized until forward dam-break drainage event occurs. Guided by this model, we next asked how structural design in B region could increase the retained liquid volume while still maintaining the system at a clear dam-break drainage event threshold. To address this, we developed a composite dam (Fig. 2cⅢ). Unlike the alone molecular dam, this composite structure incorporates a microscale glass protrusion (physical dam) in the B region, upon which the tri-block probes were immobilized. To identify the optimal dam dimensions required to form a hemispherical droplet, we fixed the dam width at 90 µm and systematically varied its height from 0.12 to 0.27 mm by adjusting the laser etching power (Supplementary Fig. 5). Progressively raising the height increased the sample retention volume from 150 µL to 600 µL (Fig. 2eⅢ–Ⅵ). Similarly, when the height of the physical dam within the composite dam increased to 0.27 mm and 600 µL of liquid was loaded into A region, the forces acting on the liquid at the interface between A and B regions were as shown in Fig. 2dⅢ:

5 $${F}_{\left({{\rm{MD}}}+{{\rm{PD}}}\right)}+{F}_{{{\rm{sl}}}}+{F}_{{{\rm{lv}}}}\cos \theta={F}_{{{\rm{sv}}}}$$

where *F*_(MD+PD)_ represents the resistance provided by the composite dam. Upon further increasing the liquid volume in A region, the force balance changes to (Fig. 2dⅣ):

6 $${F}_{\left({{\rm{MD}}}+{{\rm{PD}}}\right)}+{F}_{{{\rm{sl}}}}+{F}_{{{\rm{lv}}}}\cos \theta < {F}_{{{\rm{sv}}}}$$

Experimentally, when the physical dam height in the composite dam reached 0.27 mm, three independent chips consistently confined 600 µL of liquid within the A region, whereas increasing the loaded volume to 700 µL caused spontaneous breakthrough into the downstream regions (Fig. 2eⅥ). Thus, 600 µL represented the maximum stable retention volume immediately below the drainage threshold and was selected as the threshold-confined volume for sensing. Under this condition, the oversized droplet remained stable in the absence of target but was poised for target-triggered drainage, enabling limited molecular perturbation at the dam interface to be converted into a clear macroscopic dam-break drainage readout. Logically, for a given dam height, a narrower dam facilitates the dam-break drainage by requiring less probe degradation, thus improving detection sensitivity. Next, we tuned the dam width from 60 to 140 µm by adjusting the laser etching pattern, while keeping other conditions constant (Supplementary Fig. 6). When the width was below 90 µm, the tri block probe-modified surface is too narrow to provide adequate hydrophobicity. In this case, the liquid failed to be confined in A region, irrespective of target presence. When the dam width ranged from 90 to 100 µm, the liquid is stably confined within A region in the absence of targets, whereas the presence of targets produced a distinct dam-break drainage event within 2 min rapid decision time. Conversely, when the dam width exceeded 100 µm, the liquid was confined within A region regardless of the presence or absence of the target within 10 min observation window. This behavior is most consistent with an interface-dependent, layer-by-layer degradation process of the tri-block probes mediated by activated Cas13a on the molecular dam. The probes directly contacting the liquid are expected to be cleaved first, followed by progressive degradation toward the inner region of the dam. These results suggest that the hydrophobicity of the dam primarily acts as a macroscopic wetting barrier that restricts spontaneous liquid spreading, rather than preventing aqueous molecules from accessing the dam surface. Accordingly, probe cleavage initiated at the liquid–dam interface rather than occurring uniformly across the entire hydrophobic barrier. When the dam width is excessive, the wettability transition cannot propagate across the full width of the dam within the allowed time, and the liquid therefore remains confined in A region despite the presence of target nucleic acid. Together, these width-dependent results define the operational threshold window of the molecular dam, in which the assay liquid is stably confined without target but remains sensitive to target-induced weakening of the wetting barrier. Therefore, we determined 90 µm in width and 0.27 mm in height as the optimal configuration for the physical dam.

### The single-plex chip achieved clinical-grade accuracy within 2 min rapid decision time

**Fig. 3:**
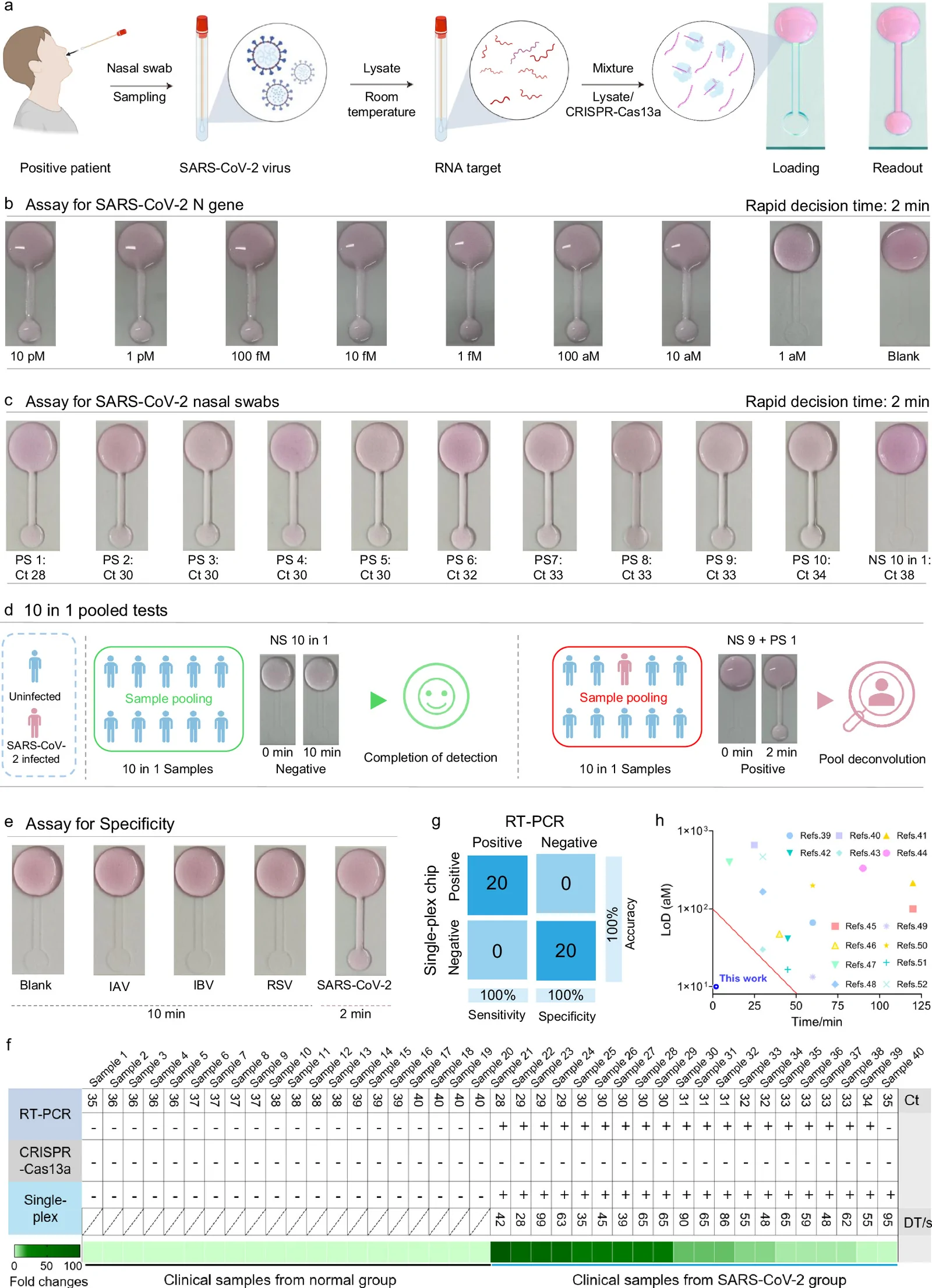
Performance evaluation of the single-plex chip for SARS-CoV−2 detection. **a** Workflow schematic for SARS-CoV-2 detection (Created in BioRender. Li, Z. (2026) https://BioRender.com/fbfrhlm). **b** Detection of serial dilutions of the SARS-CoV-2 *N* gene ranging from 1 aM to 10 pM. **c** Analysis of clinical nasal swab samples with Ct values between 28 and 34. **d** 10 in 1 pooled testing: negative pools were confined, whereas pools containing a single positive sample produced a distinct dam-break drainage signal within 2 min (Created in BioRender. Li, Z. (2026) https://BioRender.com/92xs4fo). **e** Specificity assessment using Influenza A virus, Influenza B virus and respiratory syncytial virus (RSV) samples; only SARS-CoV-2 triggered the dam-break drainage event. **f** Comparative evaluation of detection performance among the single-plex chip, RT‑PCR and CRISPR-Cas13a. Fold changes denote the normalized relative abundance of SARS-CoV-2 target RNA estimated from RT–PCR Ct values, using the positive sample with Ct=34 as the reference (set to 1). **g** Confusion matrix relative to clinical RT‑PCR results, indicating 100% sensitivity, specificity and accuracy. **h** Benchmarking of the developed chip against previously reported detection technologies. Each data point represents a previously reported nucleic acid detection method. Detailed information is provided in Supplementary Table 3. Source data are provided as a Source Data file.

As shown in Fig. 3, we evaluated the performance of the single-plex chip in detecting infectious pathogens using SARS-CoV-2 as a model. As shown in Fig. 3a, SARS-CoV-2 detection is achieved simply by adding a mixture of the target nucleic acid and the CRISPR-Cas13a to the sample reservoir, with the presence or absence of liquid flow providing a direct visual readout. Before testing the full-length SARS-CoV-2 RNA (29,903 nt)^30^, we first assessed the chip′s response to the *N* gene (1260 nt)^31^ to validate its feasibility. Due to the target-sequence dependence of CRISPR-Cas13a′s activity, we selected the optimized *N* gene region spanning nucleotides from 38 to 65 as the recognition site^22,32,33^. As shown in Fig. 3b and Supplementary Movie 1, the chip enabled rapid and highly sensitive detection of the SARS-CoV-2 *N* gene over a wide concentration range (1 aM–10 pM), with a limit of detection (LOD) of 10 aM. In three independent measurements (*n* = 3), all tested positive groups consistently triggered visible dam-break drainage event within 2 min, whereas blank samples remained confined throughout the 10 min observation window, supporting the single-plex visual readout rule of rapid decision time (Supplementary Fig. 7). As shown in Supplementary Figs. 8, 9, the developed chip outperforms both commercial RT–PCR kits (100 aM, 35 min) and standalone CRISPR-Cas13a assay (10 pM, 40 min) in terms of detection limit and readout time.

Given the satisfied performance in *N* gene detection, we next evaluated the chip′s response to full-length SARS-CoV-2 RNA of nasal swabs to validate its performance in complex matrices (Fig. 3c). During clinical testing, RNases in sample matrices will work as the activated Cas13a protein to degrade tri-block probes, causing false-positive signals. To reduce matrix-derived false triggering and identify a practical operating workflow, we optimized the sample pretreatment conditions for clinical swab samples (Supplementary Discussion). As shown in Supplementary Fig. 10a, complete RNase inhibition for throat swabs required both heating to lysate and the addition of RNase inhibitor (RRI). While for nasal swabs, adding RRI to the lysate was sufficient to suppress matrix-derived RNase interference without the need for heating (Supplementary Fig. 11a). Notably, room-temperature lysis of nasal swabs provided higher RNA extraction efficiency than heating lysis (Supplementary Fig. 12). We further confirm that the activity of CRISPR-Cas13a will not be compromised by the addition of RRI alone or in combination with the lysate under the proposed loading (Supplementary Figs. 10b, 11b, 13). Based on these results, we selected nasal swabs processed by room-temperature lysis with RRI as the working sample workflow for subsequent clinical validation, owing to its operational simplicity, compatibility with Cas13a activity and lower susceptibility to matrix-derived false triggering under the tested conditions. In assay, ten RT–PCR positive nasal swabs (Ct = 28–34) and ten RT–PCR pooled negative nasal swabs (Ct = 38) were tested (Supplementary Fig. 14). As a result, all positive clinical nasal swab samples generated a clear dam-break drainage event, whereas pooled negative samples remained confined in the sample reservoir within 2 min and remained confined through the 10 min observation window (Fig. 3c and Supplementary Movie 2). These results were confirmed by three independent measurements (*n* = 3), further supporting the robustness of the developed workflow (nasal swab + room-temperature lysis + RRI) (Supplementary Fig. 15a). We further evaluated chips′ performance in pooled testing scenarios, which are advantageous for large-scale screening and cost reduction. Figure 3d shows that pooled samples containing one positive sample and nine negative samples triggered the dam-break drainage event within 2 min rapid decision time, whereas fully negative pooled samples remained blocked after 10 min observation window. To assess specificity, the chip was tested against three common respiratory viruses-H1N1^34,35^, IBV^36,37^ and respiratory syncytial virus^38^. As shown in Fig. 3e, the dam-break drainage event was exclusively triggered by SARS-CoV-2, while all other viruses showed blank-like responses over 10 min observation window. Finally, we evaluated its clinical accuracy using 40 nasal swab samples (RT–PCR-negative samples, *n* = 21 and RT–PCR-positive samples, *n* = 19, Fig. 3f). The single-plex chip enabled reliable identification of positive samples with Ct values up to 35, while the pure CRISPR-Cas13a system failed to discriminate positive samples from negative samples, even for positive sample with Ct values down to 28 (Supplementary Fig. 16). Confusion matrix analysis indicated that the chip achieves 100% sensitivity, specificity and accuracy (Fig. 3g), outperforming RT–PCR (sensitivity 95%, specificity 100%, accuracy 97.5%; Supplementary Fig. 15b). Compared to other detection platforms^39–52^, such as diagnostics with coronavirus enzymatic reporting (DISCoVER)^39^, colorimetric detection^40^ and CRISPR-Cas13a cascade-based viral RNA (Cas13C) assay^41^, our single-plex chip demonstrates a lower detection limit and faster speed (Fig. 3h and Supplementary Table 3).

### The surface energy-driven self-replenishment strategy for reliable duplex detection

**Fig. 4:**
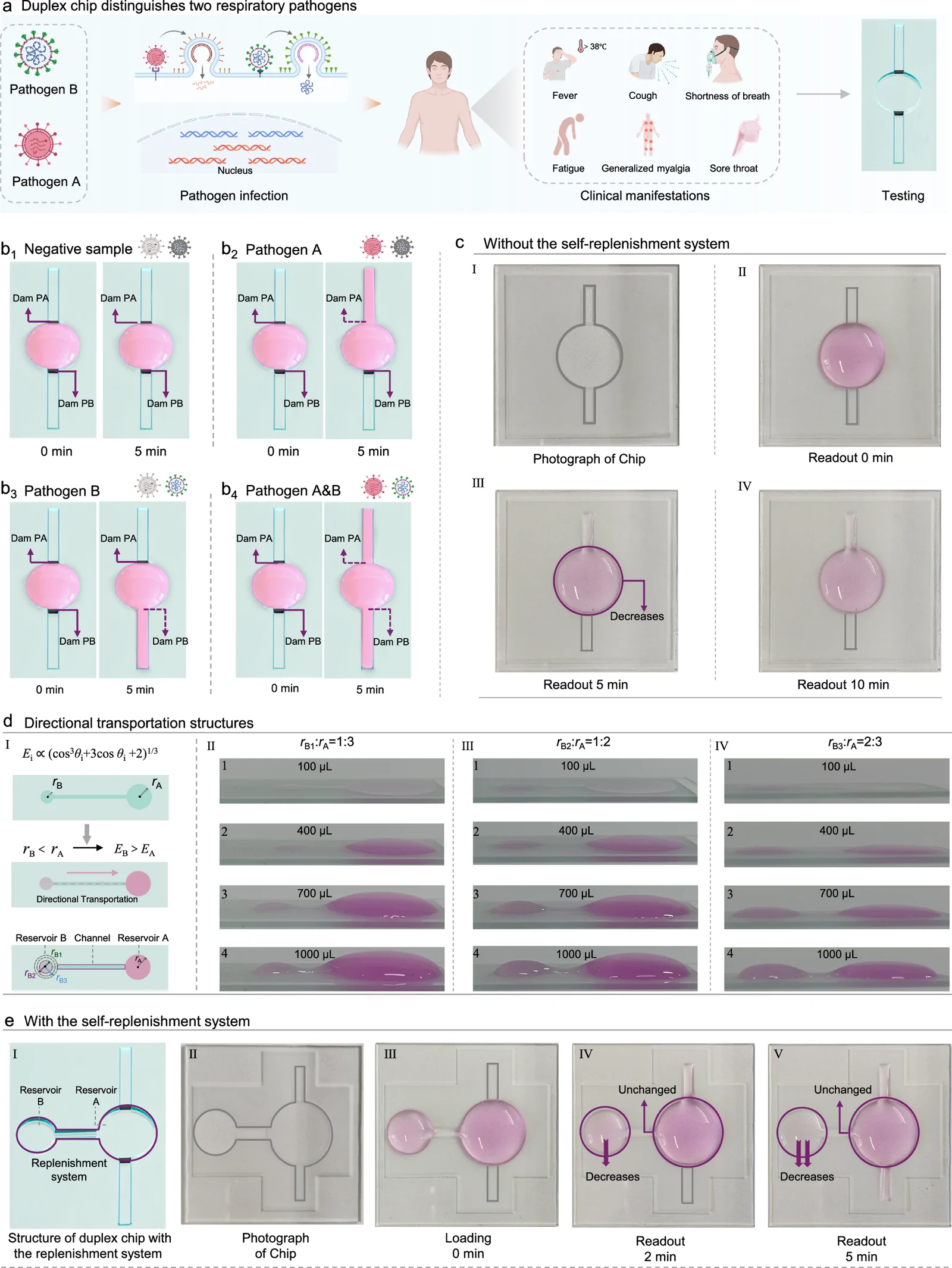
Design and performance of the duplex chip in detecting two pathogens. **a** Schematic of the duplex chip designed to differentiate two respiratory pathogens (PA and PB) with similar symptoms (Created in BioRender. Li, Z. (2026) https://BioRender.com/6wjl2xr). **b** Representative results showing chip′s response to: negative sample (**b**_**1**_); sample containing only PA (**b**_**2**_); sample containing only PB (**b**_**3**_); sample containing both PA and PB (**b**_**4**_). Dam-break drainage signals specific to each pathogen dam (Dam PA or Dam PB) appear within 5 min (Created in BioRender. Li, Z. (2026) https://BioRender.com/3rkcath). **c** Limitation of the chip without a self-replenishment system. Image of the duplex chip (**cⅠ**). Sequential images demonstrate that with a finite sample volume, only the first target is detected, as the liquid is consumed and cannot initiate the second dam-break drainage signal (**cⅡ–Ⅳ**). **d** Directional transportation structure. Schematic of the directional transportation mechanism (**dⅠ**). Demonstration of the system’s efficacy across different *r*_B_:*r*_A_ ratios, showing sustained operation with liquid volumes from 100 to 1000 µL (**dⅡ–Ⅳ**). **e** Enhanced performance with integrated self-replenishment. Design of the optimized duplex chip with the self-replenishment system (**eⅠ**). Photograph of the fabricated chip (**eⅡ**). Sequential detection of two targets from a mixed sample: the first target is detected at 2 min, followed by the second at 5 min, enabled by continuous liquid supply from the self-replenishment reservoir (**eⅢ–V**).

As shown in Fig. 4a, respiratory infections caused by different pathogens often exhibit overlapping clinical manifestations. For example, Influenza A virus infection typically causes fever, headache and cough, symptoms that closely resemble those of Influenza B virus and other respiratory viruses^53^. Consequently, relying solely on clinical symptoms makes it difficult to accurately determine the underlying pathogen, leading to potential misdiagnosis or delayed treatment. To address this issue, we further developed a duplex chip capable of simultaneously identifying two pathogens. As shown in Fig. 4b, the detection model of duplex chip is operated as follows: when the detection solution is added into the sample reservoir, the presence of pathogen A (PA) nucleic acid triggers a dam-break drainage signal at Dam PA (similarly for pathogen B), whereas the coexistence of two viral targets generates signal in both Dam PA and Dam PB. However, a critical challenge arises from the chip′s structural design: once one target nucleic acid induces the dam-break drainage event, the liquid volume in the sample reservoir decreases significantly (Fig. 4c). This diminishes the threshold state required for the subsequent dam-break drainage event readout, thereby reducing the detection sensitivity of the other target and even leading to false-negative results. To address this issue, we engineered the duplex chip with a self-replenishment system based on a directional transportation structure^54^ (Fig. 4dⅠ). The directional transportation structure comprised reservoir A, channel and reservoir B. When the dimension of reservoir B is smaller than that of reservoir A, the liquid in reservoir B presents a higher surface energy (*E*_B_) than that in reservoir A (*E*_A_). The surface energy difference spontaneously drives liquid directional transport from reservoir B to reservoir A^55^. Under a fixed channel, we systematically investigated the effect of dimension ratio between reservoir B and reservoir A (*r*_B_:*r*_A_) on directional transport efficiency. As shown in Fig. 4dⅡ and Supplementary Movie 3, when *r*_B_:*r*_A_ = 1:3, liquid added to reservoir B will smoothly be transferred to reservoir A. Reservoir A collects most of the liquid and only a minimal amount retained in reservoir B, a trend consistent across volumes from 100 μL to 1000 μL. Similarly, when *r*_B_:*r*_A_ = 1:2, the liquid also will be automatically transferred from reservoir B to reservoir A except for a small amount of liquid began to accumulate in reservoir B, as the loading volume growing from 100 to 1000 μL (Fig. 4dⅢ). When the ratio was set to 2:3, the liquid initially accumulated in reservoir B and then gradually flowed into reservoir A. As the loading volume increased, substantial liquid accumulation occurred in both reservoirs, while the liquid increment in reservoir A being markedly greater than that in reservoir B (Fig. 4dⅣ). The directional transportation structure is promising to resolve the challenge in duplex detection, where the liquid volume reduction induced by the first target disrupts the drainage threshold and consequently impairs the sensitivity of the subsequent target detection. Thus, we incorporated this directional transportation structure into the duplex chip, equipping it with a self-replenishment capability (Fig. 4eⅠ, Ⅱ). However, this design involves a trade-off between the efficiency and capacity of the self-replenishment process. As shown in Supplementary Fig. 17, at *r*_B_:*r*_A_ ratios of 1:3 or 1:2, the self-replenishment system responded promptly to the first target, while the replenished liquid volume was inadequate to maintain the drainage threshold, as reservoir B initially retained only a limited amount of liquid. In contrast, when the ratio of *r*_B_:*r*_A_ was increased to 2:3, the system achieved a more balanced performance. It not only ensured efficient directional liquid transfer after the first detection event but also supplied sufficient replenishment to preserve the drainage threshold, thereby supporting reliable dual-target detection. As shown in Fig. 4eⅢ, Ⅳ, after the first target triggered the dam-break drainage event, reservoir A maintained a constant liquid volume while reservoir B performed a decreased fluid volume. Then, the second target was reliably detected within 5 min, during which the liquid in A reservoir maintains threshold state continuously (Fig. 4eⅤ).

### A dam-anchored and self-releasing crRNA design to restore selective and efficient Cas13a activation

**Fig. 5:**
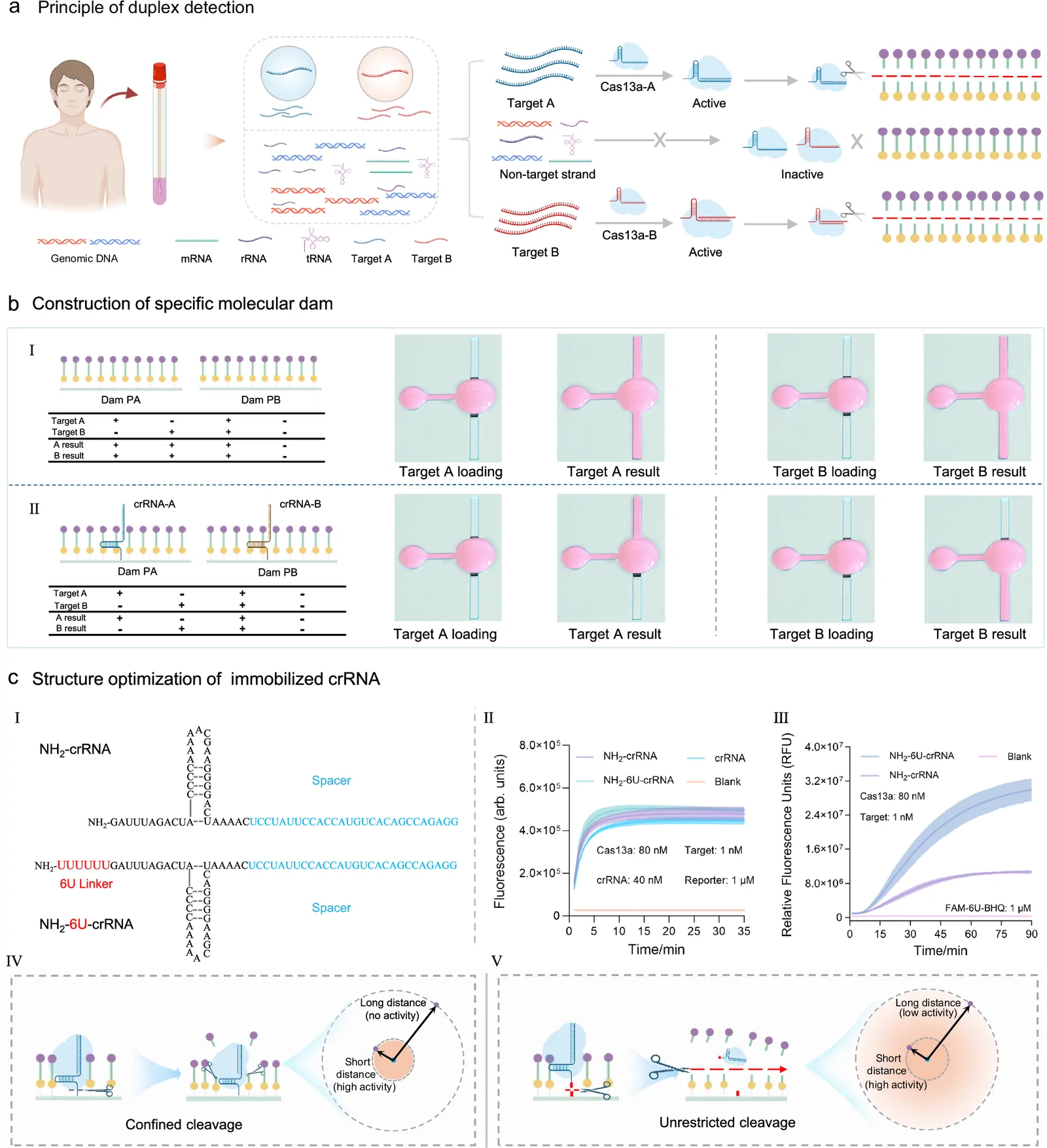
Engineering specific molecular dams and optimizing crRNA architecture for duplex detection. **a** Working principle: two target RNAs orthogonally activate their respective CRISPR-Cas13a systems, leading to the specific cleavage of their corresponding tri-block probes on the molecular dams (Created in BioRender. Li, Z. (2026) https://BioRender.com/btdam4r). **b** Formation of specific molecular dams: A non-specific dam, constructed from tri-block probes alone, is susceptible to be break by any activated Cas13a, leading to potential false-positive signals (**bⅠ**). A specific molecular dam, incorporating both tri-block probes and matched crRNA, ensures that drainage occurs only upon recognition of the intended target, thereby conferring specificity (**bⅡ**). **c** crRNA design for duplex chip. **cⅠ** Structures of the two crRNA designs: amino-modified crRNA and amino-6U-modified crRNA. **cⅡ** Comparison of Cas13a collateral kinetics between amino-crRNA and amino-6U-crRNA in free solution. **cⅢ** Comparison of activated Cas13a-mediated cleavage efficiency between amino-crRNA and amino-6U-crRNA under surface-anchored conditions. Three independent kinetic traces are shown for each condition. For (**cⅡ**) and (**cⅢ**), Solid lines represent the mean fluorescence intensity and the shaded areas indicate the min-max range (*n* = 3 independent experiments). Schematic comparison of linker-free and 6U-linker crRNA designs for duplex chip construction, showing that the linker-free design confines the activated Cas13a complex near the anchoring site (**cⅣ**), whereas the 6U-linker design allows activation-triggered release of the Cas13a/crRNA complex, thereby extending its effective cleavage range (**cⅤ**). Source data are provided as a Source Data file.

In the single-plex assay, SARS-CoV-2 detection is simply achieved by adding a mixture of the target nucleic acid and the CRISPR-Cas13a solution to the sample reservoir, with the presence or absence of liquid flow providing a direct visual readout. However, this strategy is not applicable to the duplex chip, where two distinct targets both can trigger the tri-block probe degradation (Fig. 5a). The CRISPR-Cas13a activated by either target would cleave the probes in both dams, resulting in a complete loss of detection specificity (Fig. 5bⅠ). To address this issue, we functionalized the 5’ end of the crRNA with an amino group for covalent immobilization on different dams (Fig. 5bⅡ). In this configuration, each molecular dam contains both the tri-block probes and crRNAs that specifically recognize the fixed pathogen nucleic acid. During detection, each pathogen′s nucleic acid specifically activates only its cognate CRISPR-Cas13a complex at the fixed channel, which in turn cleaves the nearby tri-block probes. This strategy effectively eliminates cross-reactivity and prevents false-positive signals. Although this strategy successfully ensures specificity, it arise two questions: (1) whether the amino-modified crRNA will decrease the ability of Cas13a collateral efficiency; (2) How to improve the degradation efficiency of the immobilized CRISPR-Cas13a. Once the CRISPR-Cas13a complex is immobilized on the molecular dam, the activated Cas13a proteins are spatially confined and can only degrade nearby tri-block probes, which weakens the overall degradation efficiency and hampers the wettability transition of the dam surface. To overcome this, we further optimized the crRNA design by inserting a Cas13a-responsive linker (6U) between the amino group and the crRNA sequence (Fig. 5cⅠ). Then, we conducted the comparison experiments to evaluate how the amino-crRNA without the 6U linker and the amino-6U-crRNA containing the 6U linker affect the Cas13a collateral efficiency using the and conventional crRNA as reference. We first compared these three crRNA designs in free solution. Results from three independent experiments showed that the three crRNA types exhibited essentially the same Cas13a collateral efficiency in free solution (Fig. 5cⅡ). This indicates that amino modification of the crRNA does not impair Cas13a collateral efficiency, and that introduction of the 6U linker also does not affect Cas13a collateral efficiency. We then further examined how surface-immobilized amino-crRNA without the 6U linker and surface-immobilized amino-6U-crRNA containing the 6U linker affect Cas13a collateral efficiency. To make this comparison consistent with the actual chip-working condition, we used the same glass substrate and immobilization procedure as those used in the chip system. Specifically, the two crRNA designs were immobilized onto glass surfaces using the same method as in chip fabrication. The modified glass substrates were then cut into small disks with a diameter of 6.5 mm to fit a 96-well microplate reader. During the assay, all other components of the CRISPR-Cas13a reaction system were kept the same, and only the surface-immobilized crRNA design differed between the two groups. The glass disks carrying immobilized crRNA without spacer or with spacer were added into the corresponding reaction solutions, and the fluorescence reporter-cleavage signal was monitored in real time using a microplate reader. Each group included three independent experiments. Through this design, we were able to directly compare Cas13a collateral efficiency of the two crRNA configurations under surface-immobilized conditions, rather than simply comparing their enzymatic activity in free solution. The results showed that the introduction of the 6U linker significantly increased molecular cleavage efficiency under surface-anchored conditions (Fig. 5cⅢ). Mechanistically, in the linker-free design, the activated Cas13a/crRNA complex remains locally tethered after target recognition and therefore mainly acts on nearby tri-block probes (Fig. 5cⅣ). In contrast, in the 6U-containing design, the activated Cas13a rapidly cleaves the linker, thereby releasing the Cas13a/crRNA complex from this confined local state and enabling degradation of more distant tri-block probes (Fig. 5cⅤ). Thus, we selected the amino-6U-crRNA as the optimized crRNA configuration for the subsequent duplex chip experiments.

### The duplex chip offered reliable pathogens identification in synthetic fragments, clinical samples and pooled scenarios

**Fig. 6:**
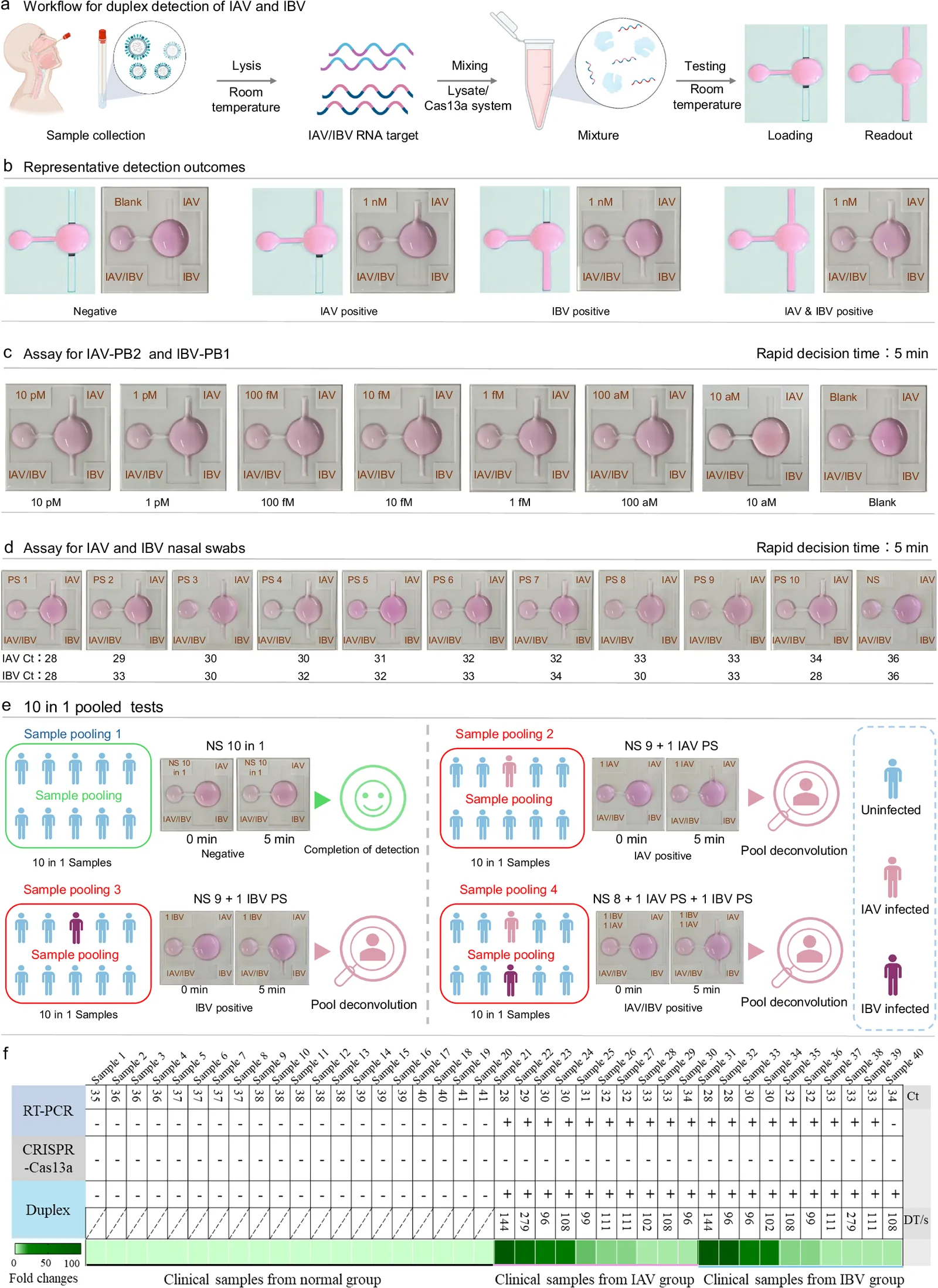
Duplex chip evaluation for Influenza A (IAV) and B (IBV) virus detection. **a** Schematic illustration of the workflow for duplex detection of IAV and IBV on the duplex chip (Created in BioRender. Li, Z. (2026) https://BioRender.com/g6kgq5h). **b** Representative detection outcomes for negative, IAV-positive, IBV-positive and dual-positive samples. The dam-break drainage event appears exclusively at the corresponding channel within 5 min. **c** Sensitivity assay for IAV *PB2* and IBV *PB1* RNA pooled samples with concentration ranging from 10 aM–10 pM in 5 min rapid decision time. **d** Clinical validation with nasal swab samples: pooled analysis of ten IAV-positive (Ct = 28–33) and ten IBV-positive (Ct = 28–33) clinical specimens showed accurate target identification without cross-reactivity. **e** 10 in 1 pooled testing: the negative pool produced no dam-break drainage event, whereas pools containing a single IAV-positive or IBV-positive sample generated clear dam-break drainage event on their corresponding channel within 5 min rapid decision time. Dual-positive pools present dam-break drainage events on both channels (Created in BioRender. Li, Z. (2026) https://BioRender.com/orn93om). **f** Comparative analysis of detection performance across the duplex chip, RT–PCR and CRISPR-Cas13a assays in sensitivity and detection time. Fold changes denote the normalized relative abundance of the corresponding target estimated from RT–PCR Ct values, using the positive sample with Ct = 34 as the reference (set to 1) for each target. Note: For the duplex assay, blank and negative controls showed stable liquid confinement over 10 min observation period, whereas all target-positive samples, across the tested concentration range, produced a dam-break drainage event within 5 min. Accordingly, 5 min was defined as the result rapid decision time. Source data are provided as a Source Data file.

As shown in Fig. 6, we employed Influenza A virus (IAV)^56^ and Influenza B virus (IBV)^57,58^—two common respiratory pathogens—as models to evaluate the duplex chip′s capability for simultaneous dual-target detection. As shown in Fig. 6a, the result can be interpreted simply loading the mixture of lysate/Cas13a protein and observing whether a dam-break drainage event appeared in fixed channel. Before testing full-length IAV and IBV RNA, we first examined the chip′s performance toward the IAV *PB2* (314–342 nt)^59^ and IBV *PB1* (1296–1324 nt)^58^ gene fragments. As shown in Fig. 6b and Supplementary Movies 4–6, within 5 min rapid decision time, in the absence of targets, the liquid is confined within the sample reservoir with no observable dam-break drainage event occur; in contrast, the presence of the *IAV-PB2* gene alone triggers a dam-break drainage event specifically in the IAV channel; Similarly, the presence of the *IBV-PB1* gene alone triggers a dam-break drainage event specifically in the IBV channel; When both targets coexisted, the chip sequentially detected each target. As shown in Fig. 6c and Supplementary Movie 7, the duplex chip allowed IAV *PB2* and IBV *PB1* RNA targets to be simultaneously detected when they were mixed at equal concentrations, with an LOD of 100 aM. In three independent measurements (*n* = 3), each positive group consistently generated visible dam-break drainage event within the 5 min readout period, while blank sample showed no dam-break drainage event and remained confined during the 10 min observation window, validating the duplex visual interpretation rule of 5 min rapid decision time (Supplementary Fig. 18). The sensitivity of the duplex chip rivals that of conventional RT–PCR assays (Supplementary Figs. 19, 20) and vastly surpass the pure CRISPR-Cas13a system (Supplementary Fig. 21). Moreover, the decision time of the developed duplex chip is 5 min, which is much shorter than that of the two methods compared.

To assess the potential for real-world use, the chip performance was further examined by testing ten groups of randomly mixed IAV RT–PCR-positive (Ct = 28–34, Supplementary Fig. 22) and IBV RT–PCR-positive samples (Ct = 28–34, Supplementary Fig. 23). As a result, both channels produced distinct dam-break drainage events within 5 min, whereas negative samples were confined in the sample reservoir without any dam-break drainage event even after 10 min (Fig. 6d and Supplementary Movie 8). The reproducibility of these results was verified across three independent measurements (*n* = 3), demonstrating the robustness of the duplex visual assay in clinical sample matrices under the developed working sample flow (Supplementary Fig. 24a). Given the advantages of pooled testing for large-scale screening, we systematically evaluated the performance of our duplex chip in this senior. Four groups were prepared: (i) ten negative samples, (ii) one IAV-positive sample mixed with nine negative samples, (iii) one IBV-positive sample mixed with nine negative samples and (iv) one IAV-positive sample and one IBV-positive sample mixed with eight negative samples. As shown in Fig. 6e, each positive pooled group triggered a dam-break drainage signal exclusively in the fixed channel, while the dual-positive pool generated signals in both channels. Conversely, the liquid in the negative pool remained entirely restricted to the sample reservoir. Then, we compare the clinical performance among the duplex chip, the RT–PCR and the pure CRISPR-Cas13a. As summarized in Fig. 6f, both RT‑PCR and our duplex chip assay correctly identified all IAV-positive, IBV-positive and negative nasal swab samples, while the duplex chip delivering results in a significantly shorter time. In contrast, the CRISPR-Cas13a system alone failed to distinguish RT–PCR-positive from RT–PCR-negative nasal swab samples of both IAV and IBV (Supplementary Fig. 25). In addition, confusion matrix analysis confirmed that, against RT‑PCR as the reference method, our dual-detection chip achieved 100% sensitivity, specificity and accuracy for both targets (Supplementary Fig. 24b). A comparative analysis with previously reported methods for IAV and IBV detection^60,61^ (Supplementary Fig. 24c and Supplementary Table 4) demonstrated that the duplex chip achieves superior sensitivity and faster response, highlighting its potential for early screening and precision diagnosis.

Given that, in practical use, the time from sample collection to testing may vary among samples, and the RNase-related interference may also differ across patient-derived sample matrices, we further performed a short-term storage robustness evaluation based on the final nasal swab workflow for both single-plex and duplex chip (Supplementary Figs. 26, 27). A total of 60 samples were tested, including 20 SARS-CoV-2, 20 IAV and 20 IBV samples, with 10 positive and 10 negative samples in each group. We compared three handling conditions: immediate testing after collection and testing after 1 h or 2 h of storage at room temperature following collection. These time points were selected because they cover the most practically relevant operating window for the intended use of this method. This includes immediate testing, short on-site delay and moderate delay caused by parallel handling of multiple samples. Specifically, because fresh positive clinical nasal swab samples were difficult to obtain, we added known concentrations of target RNA into 10 fresh negative nasal swab samples collected from 20 volunteers to form simulated positive samples. This produced 10 positive samples with Ct values from 28 to 34 and 10 negative samples with Ct values from 35 to 39. The results showed that, under these short-term handling variations, both the single-plex and duplex chips correctly distinguished negative and positive nasal swab samples within their respective rapid decision times. All 10 negative samples remained stably confined in the sample reservoir, with no obvious nonspecific dam-break drainage event. In contrast, all 10 positive samples produced the expected dam-break drainage event. As this method is designed for rapid, instrument-free testing in family and community settings, the 0–2 h window reflects the main use scenario considered in this study. The proposed results show that the developed nasal swab workflow remained feasible and stable under short-term handling variation within this window. We have therefore described these data as a focused robustness evaluation of the developed workflow, rather than as a full pre-analytical stability study covering all sample media, storage times and matrix conditions.

## Discussion

In this study, we established single-plex and duplex dam-break drainage event chips based on CRISPR-Cas13a-mediated interfacial wettability switching and a visible macroscopic fluidic readout. The central advance of this work lies in creating a steady-state detection mode under fixed-boundary conditions, in which target recognition is directly converted into organized liquid-motion output without subsequent instrumentation or manual actuation. Compared with the previous tube-based implementation, the present chip architecture removes the operational uncertainty associated with manual flipping and enables stable signal triggering after one-step sample loading. In parallel, by establishing a practical nasal swab workflow based on room-temperature lysis and RNase inhibition, we further show that this sensing strategy can operate reliably in clinical sample matrices rather than only under idealized reaction conditions. Mechanistically, the rapid and highly sensitive performance of the chip arises from the coupling of CRISPR-Cas13a-mediated molecular amplification with chip-level threshold conversion. Cas13a collateral cleavage provides target-dependent molecular perturbations, while the chip architecture regulates the force balance at the liquid–dam interface and maintains the assay liquid in a threshold-confined state. Under this condition, a limited reduction in the local wetting barrier is sufficient to break the interfacial balance and nonlinearly trigger a macroscopic dam-break drainage transition, establishing a threshold-dependent relationship between molecular probe degradation and macroscopic drainage behavior. This coupled amplification process enables molecular recognition events to be read out beyond the sensitivity range achievable by the standalone CRISPR-Cas13a assay, without requiring nucleic acid pre-amplification or external instrumentation. The significance of developed duplex module lies not merely in placing two targets on one chip, but in showing that parallel and distinguishable visual outputs can be organized under shared-system conditions. Achieving this required preserving the threshold state after the first triggering event and restoring local selectivity when both targets could activate Cas13a within the same reaction environment. The directional self-replenishment structure, together with the dam-anchored and self-releasing crRNA design, provides the structural basis for this organized duplex readout. At the same time, the current study remains limited to qualitative visual detection, and multiplexing has been experimentally established only at the duplex level. Further expansion to higher-order multiplexing will require systematic investigation of signal independence, threshold maintenance and readout organization in more complex shared systems. Likewise, although the underlying wettability switching readout principle may, in principle, be extendable to other analyte classes through appropriate upstream recognition modules, such broader applicability has not been experimentally demonstrated here. In this sense, the methodology established in the present work provides a foundation for future exploration of higher-order multiplex visual biosensing based on interfacial wettability switching and macroscopic fluidic readout.

## Methods

### Ethics statement

The collection and use of clinical samples complied with all relevant ethical regulations for research involving human participants. The study protocol was reviewed and approved by the Research Ethics Board of the University of Science and Technology Beijing (Approval No. 2025-4-154). All residual clinical specimens applied in this study were fully de-identified before delivery. The institutional ethics committee reviewed this research and waived the requirement to obtain individual informed consent from sample donors. No compensation was provided to the sample donors.

### Materials

Glass slides were purchased from Yufan Laboratory Equipment Co., Ltd. (Yancheng, Jiangsu, China). 1H,1H,2H,2H-Perfluorodecyltrimethoxysilane (FAS-17), 3-Aminopropyltriethoxysilane (APTES), glutaraldehyde (50%) and phosphate-buffered saline (10× PBS) were purchased from Aladdin Reagent Co., Ltd. (Shanghai, China). Toluene and ethanol were obtained from Sinopharm Chemical Reagent Co., Ltd. TaqProbe 2× qPCR-Multiplex Premix and RNase Inhibitor (RRI) were obtained from Sangon Biotech Co., Ltd. (Shanghai, China). The SARS-CoV-2 *N* gene plasmid (pUC57-2019-nCoV-N), the Influenza A virus (IAV *PB2*) target, Influenza B virus (IBV *PB1*) target and respiratory syncytial virus (RSV) target were obtained from GenScript (Nanjing, China). The DNA amplification kit, all TaqMan probes, all forward primers, all reverse primers, tri-block probes, all crRNAs, InviTrans T7 In Vitro Transcription Kit (AG51201) and SteadyPure RNA Extraction Kit (AG21017) were purchased from Accurate biotechnology (Hunan) Co., Ltd. (Changsha, China). NEBuffer 4 was obtained from New England Biolabs International Trading Co., Ltd. (Shanghai, China). LwaCas13a protein was purchased from Huicheng Biotechnology Co., Ltd. (Shanghai, China). The lysis buffer was purchased from Shandong Huifan Medical Instrument Co., Ltd. (China). Confocal fluorescence images were acquired using a LeiCa-TCS SP8 laser scanning confocal microscope (Leica Microsystems, Germany). A laser pattern system was obtained from Wuhan Harmonious Horizon Laser Equipment Co., Ltd. (Wuhan, China). Contact angles were measured using an OCA 20 contact-angle system (Data Physics Instruments, Germany). Dam heights and widths were measured using an SU8010 scanning electron microscope (Hitachi, Japan) and an LV100ND metallurgical microscope (Nikon, Japan), respectively. PCR amplification, real-time quantitative PCR analysis and nucleic acid quantification were performed using an Applied Biosystems 2720 Thermal Cycler, the StepOne Real-Time PCR System and a Thermo Fisher NanoDrop One spectrophotometer, respectively. The sequences of all nucleic acids used in this work are provided in Supplementary Tables 5–9.

### Protocol for the single-plex chip

Step 1. Structure pattern for chip surface: The glass slide (76.2 mm × 25.4 mm) surface was patterned with microstructure ready for chemical modification by the laser pattern system. The pattern measures 22 mm in width and 43 mm in length, which works as the chip substrate (Supplementary Fig. 28a step Ⅰ, S28bⅠ, Ⅱ and Supplementary Table 10). The detailed laser settings are provided in Supplementary Table 2.

Step 2. FAS-17 treatment (Supplementary Fig. 28a step Ⅱ): The laser-treated glass slide was immersed in a 0.1% (v/v) FAS-17/toluene solution and reacted at room temperature for 60 min. Then the modified glass slide was washed with deionized water and ethanol. Finally, the obtained glass slide was dried at 60 °C for 5 min.

Step 3. Functional zones pattern: Four functional zones were patterned on the chip substrate using the laser pattern system. The detailed dimensions of four patterned zones are shown in Supplementary Fig. 28a step Ⅲ, S28bⅢ–Ⅵ and Supplementary Table 10.

Step 4. APTES treatment: As shown in Supplementary Fig. 28a step Ⅳ, to achieve amination of the chip surface, the glass slide was immersed in a 60% (v/v) APTES/ethanol solution and react at room temperature for 60 min. Then the glass slide was washed with ethanol and dried at 60 °C for 5 min.

Step 5. Glutaraldehyde treatment: In order to immobilize the amino-modified tri-block probe on region B to form dam, the chip surface was further modified with aldehyde group by reacting with glutaraldehyde solution (pH 7.5) at room temperature for 60 min. Then, the glass slide was sequentially washed with deionized water and ethanol. Finally, the glass slide was dried at 60 °C for 5 min (Supplementary Fig. 28a step Ⅴ).

Step 6. Probe immobilization: As shown in Supplementary Fig. 28a step Ⅵ, the 5’ end aminated tri-block probe was immobilized on the chip surface via an amine-aldehyde condensation reaction. Briefly, 600 µL of a 1 µM probe solution in ethanol was introduced into the chip and allowed to react at room temperature for 60 min. Subsequently, the chip was rinsed with ethanol and dried at room temperature for 30 min.

Step 7. Region B construction: The immobilized tri-block probe was removed from chip surface except for those in region B by using the laser pattern system to form the molecular dam (Supplementary Fig. 28a step Ⅶ).

### Procedure for the single-plex chip detection

10 μL of target solution was mixed with 590 μL of CRISPR-Cas13a reagent containing 60 μL of 10× NEBuffer 4, 5 μL of LwaCas13a protein (final concentration, 80 nM), 2 μL of crRNA (final concentration, 40 nM), 33 μL of phenol red solution (200 mg/L) and 490 μL of RNase-free water. The 600 μL mixture was then loaded into the single-plex chip for detection.

### The source and pretreatment of clinical samples

Clinical samples classified as SARS-CoV-2, influenza A virus (IAV), and influenza B virus (IBV) positive were provided by the Shandong Provincial Center for Disease Control and Prevention, Prof. Lei Chen’s research group at the School of Life Sciences, Shandong Normal University, and volunteers from the School of Chemistry and Biological Engineering, University of Science and Technology Beijing. For chip analysis, swab samples were first immersed in 3 mL of lysis buffer and incubated at room temperature for 10 min. RNase inhibitor (RRI) was then added to the lysate at a ratio of 2 μL RRI per 100 μL lysate to suppress endogenous RNase activity.

### SARS-CoV-2 N gene preparation

The SARS-CoV-2 *N* gene was prepared using the following procedure: Step 1 (Amplification): An amplification mixture (50 μL total volume) was prepared, consisting of 100 ng plasmid DNA (*SARS-CoV-2 N* gene plasmid, pUC57-2019-nCoV-N), 2.5 μL forward primer (final concentration, 500 nM), 2.5 μL reverse primer (final concentration, 500 nM) and 25 μL TaqProbe 2× qPCR-Multiplex Master Mix (BBI, B630005). The reaction was then subjected to thermal cycling following the TaqProbe 2× qPCR-Multiplex Master Mix′s instructions. Step 2 (Transcription): A 50 μL transcription mixture containing 8 μL amplified product, 5 μL 10× In vitro transcription buffer, 20 μL NTP Mix, 5 μL T7 RNA Polymerase Mix and 12 μL RNase free water was incubated at 37 °C for 5 h. Subsequently, 5 U DNase I was added to digest the DNA template. Step 3 (Purification and quantification): The transcribed RNA was purified with the SteadyPure RNA Extraction Kit (AG21017) and quantified using a NanoDrop spectrophotometer. The sequences of the used primers are shown in Supplementary Table 5.

### RT–PCR kit for RNA detection

To benchmark the chips performance against the gold standard RT–PCR, RNA amplification was conducted using a one-step RT-qPCR probe kit (BBI, B639278). We employed the U.S. Centers for Disease Control and Prevention (CDC)-recommended N2 RT–PCR target site for SARS-CoV-2 *N* gene detection, amplifying the corresponding fragment with gene-specific TaqMan probe, forward primer and reverse primer on the StepOne Real-Time PCR System. Detection of Influenza A and B genes (IAV *PB2* and IBV *PB1*) followed the same procedure, with the corresponding TaqMan probe and primers sets substituted. For clinical samples, the lysates of nasal swabs were directly used as templates. The sequences of all oligonucleotides are provided in Supplementary Table 6.

### RNase activity test of Cas13a with modified crRNAs

A 10 μL reaction mixture containing 1 μL LwaCas13a (final concentration, 40 nM), 1 μL modified crRNA (final concentration, 20 nM), 1 μL ssRNA reporter (6U FAM–BHQ final concentration, 1 μM), 1 μL SARS-CoV-2 *N* gene (final concentration, 1 nM), 1 μL 10× NEBuffer 4 and 5 μL RNase-free water. Three control groups were included in this assay: Cas13a complexed with wild-type crRNA, amino-modified crRNA, or amino-6U-modified crRNA. Reactions were incubated at 37 °C and fluorescence signals were recorded every minute for 50 min using a StepOne Real-Time PCR System. Negative control was performed by replacing the target with RNase-free water.

### Protocol for the duplex chip

Step 1. Structure pattern for chip surface: The surface of the 50 mm × 50 mm glass slide was patterned with microstructures using the laser pattern system for subsequent chemical modification. Details are provided in Supplementary Fig. 29a step Ⅰ, S29bⅠ, Ⅱ and Supplementary Table 11.

Step 2. Functional zones pattern: The glass slide surface was patterned to form the functional zones using a laser pattern system. The detailed process and parameters are listed in Supplementary Fig. 29a step Ⅱ, S29bⅢ–Ⅴ and Supplementary Table 11.

Step 3. FAS-17 treatment: The detailed treatment process follows step 2 of the “Protocol for the single-plex chip” (Supplementary Fig. 29a step Ⅲ).

Step 4. Hydrophobic isolation zone formation: To construct specific molecular dams in the duplex chip, a hydrophobic isolation zone was introduced in reservoir A, effectively partitioning the chip into two structurally independent segments (A and B) for separate crRNAs immobilization. The process and parameters of constructing the hydrophobic isolation zone are provided in Supplementary Fig. 29a step Ⅳ, Supplementary Fig. 29bⅥ and Supplementary Table 11.

Step 5. APTES treatment: To functionalize the chip surface with amino groups, the glass slide was immersed in a 50% (v/v) APTES/ethanol solution and incubated at room temperature for 60 min. The slide was then rinsed with ethanol and dried at 60 °C for 5 min (Supplementary Fig. 29a step Ⅴ).

Step 6. Glutaraldehyde treatment: To immobilize the amino-modified tri-block probe and crRNAs on the chip surface for constructing specific dams′ structure, 200 µL of glutaraldehyde solution (pH 7.5) was added to each of the two independent segments on the chip surface. Then, the chip was incubated at room temperature for 60 min to achieve aldehyde modification. After the reaction, the slide was sequentially rinsed with deionized water and ethanol. Finally, the slide was dried at 60 °C for 5 min (Supplementary Fig. 29a step Ⅵ).

Step 7. Probe immobilization: Since the solvent of the tri-block probe is ethanol, the hydrophobic isolation zone prepared with FAS-17 in step 4 will not introduce phase separation with solution loading. Therefore, adding the tri-block probe solution to reservoir A will effectively enable uniform immobilization across all the chip functional zones. The detailed immobilization procedure follows step 6 of “protocol for the single-plex chip” (Supplementary Fig. 29a step Ⅶ).

Step 8. Position-specific immobilization of crRNA: To construct distinct molecular dams, a hydrophobic isolation zone was introduced into reservoir A during step 4, enabling crRNA1 (targeting IAV) and crRNA2 (targeting IBV) to be individually loaded into segments A and B, respectively. This strategy ensured the formation of crRNA-specific regions (segment A and segment B) without cross-interference. In detail, 200 µL of a 40 nM crRNA solution (prepared in 1× PBS) was added to each designated segment and incubated at room temperature for 60 min. Then the surface was rinsed with ethanol and air-dried at room temperature for 30 min. The overall workflow is illustrated in Supplementary Fig. 29a step Ⅷ.

Step 9. B region (Dam) constructions: As shown in Supplementary Fig. 29a step Ⅸ, except for tri-block probes and crRNAs retained on dams 1 and 2, all other immobilized tri-block probes and crRNAs on the chip surface were removed using the laser pattern system to construct the specific molecular dam. In addition, the hydrophobic isolation zone within reservoir A was ablated by the same laser pattern process, converting reservoir A into a single unified chamber for subsequent dual-target sample loading.

Step 10. Self-replenishment system construction: The glass slide surface was engineered using the laser pattern system to construct the directional transportation structure within the duplex chip. As shown in Supplementary Fig. 29a step X, reservoir B was fabricated to the left of reservoir A and connected to reservoir A by a channel. Reservoir B had a radius that was two-thirds of reservoir A′s, while the channel measured 3 mm in width and 10 mm in length. The parameters are as shown in Supplementary Fig. 29bⅦ and Supplementary Table 11.

### Procedure for the duplex chip detection

10 µL of target solution was added to 990 µL of Cas13a reaction mixture consisting of 100 µL of 10× NEBuffer 4, 8 µL of LwaCas13a protein (final concentration, 80 nM), 55 µL of phenol red solution (200 mg/L) and 827 µL of RNase-free water. The resulting 1 mL mixture was loaded into the duplex chip for detection.

### Statistics & reproducibility

No statistical method was used to predetermine sample size. Sample sizes for the analytical experiments were selected to evaluate reproducibility and were generally based on three independent experiments unless otherwise stated. The number of clinical samples was determined by sample availability during the study period. For analytical chip experiments, one independently fabricated chip tested with an independently prepared reaction mixture was considered one independent experimental unit. Repeated measurements using aliquots from the same sample or reaction mixture were considered technical replicates and were not treated as independent observations. Statistical analyses were performed using Nano Measurer v1.2, GraphPad Prism 10.2.0 and OriginPro 9.1 (OriginLab). Exact *n* values, replicate types, definitions of error bars and statistical tests, where applicable, are provided in the corresponding figure legends. No data was excluded from the analysis. The experiments were not randomized. The investigators were not blinded to allocation during experiments and outcome assessment. Each participant contributed one independent clinical sample, whereas repeated measurements of the same sample were considered technical replicates.

### Reporting summary

Further information on research design is available in the Nature Portfolio Reporting Summary linked to this article.

## Supplementary information

Supplementary Information

Description of Additional Supplementary Information

Supplementary Movie 1

Supplementary Movie 2

Supplementary Movie 3

Supplementary Movie 4

Supplementary Movie 5

Supplementary Movie 6

Supplementary Movie 7

Supplementary Movie 8

Reporting Summary

Transparent Peer Review file

## Source data

Source Data

## Data Availability

All data supporting the findings of this study are available within the Article, Supplementary Information and Source Data file. The Source Data file contains the underlying numerical data for all relevant main and Supplementary Figs. The sequences of used crRNA, tri-block probe and targets are provided in the Supplementary Information. No custom code was developed or used in this study. Source data are provided with this paper.
