# Supplementary material for: A CRISPR-based ‘dam-break drainage’ sensing architecture for rapid, affordable and ultrasensitive pathogen detection
Source: Nat Commun. 2026 Aug 22;17:11194. doi: 10.1038/s41467-026-77042-w (PMC13714288; doi:10.1038/s41467-026-77042-w)
Supplement: Supplementary file 1 [file 41467_2026_77042_MOESM11_ESM.pdf]

nature portfolio

Corresponding author(s): Zhengping Li and Yang Sun

Last updated by author(s): Jul 22, 2026

Reporting Summary

Nature Portfolio wishes to improve the reproducibility of the work that we publish. This form provides structure for consistency and transparency in reporting. For further information on Nature Portfolio policies, see our Editorial Policies and the Editorial Policy Checklist.

Statistics

For all statistical analyses, confirm that the following items are present in the figure legend, table legend, main text, or Methods section.

n/a	Confirmed	
☐	☒	The exact sample size (*n*) for each experimental group/condition, given as a discrete number and unit of measurement
☐	☒	A statement on whether measurements were taken from distinct samples or whether the same sample was measured repeatedly
☒	☐	The statistical test(s) used AND whether they are one- or two-sided<br>*Only common tests should be described solely by name; describe more complex techniques in the Methods section.*
☒	☐	A description of all covariates tested
☒	☐	A description of any assumptions or corrections, such as tests of normality and adjustment for multiple comparisons
☐	☒	A full description of the statistical parameters including central tendency (e.g. means) or other basic estimates (e.g. regression coefficient) AND variation (e.g. standard deviation) or associated estimates of uncertainty (e.g. confidence intervals)
☒	☐	For null hypothesis testing, the test statistic (e.g. *F*, *t*, *r*) with confidence intervals, effect sizes, degrees of freedom and *P* value noted<br>*Give P values as exact values whenever suitable.*
☒	☐	For Bayesian analysis, information on the choice of priors and Markov chain Monte Carlo settings
☒	☐	For hierarchical and complex designs, identification of the appropriate level for tests and full reporting of outcomes
☒	☐	Estimates of effect sizes (e.g. Cohen's *d*, Pearson's *r*), indicating how they were calculated

Our web collection on statistics for biologists contains articles on many of the points above.

Software and code

Policy information about availability of computer code

Data collection	Fluorescence data were collected using StepOne Software v2.3 (Applied Biosystems). Confocal images were acquired using Leica Application Suite X v4.7.0.28176 (Leica Microsystems). Contact-angle measurements were collected using Data physics oca20.Dam heights and widths were measured using an SU8010 scanning electron microscope (Hitachi, Japan) and an LV100ND metallurgical microscope (Nikon, Japan), respectively.
Data analysis	Data analysis was performed using Nano Measurer v1.2, OriginPro 9.1 (OriginLab) and GrapPad Prism 10.2.0. No custom algorithms were used for data analysis.

For manuscripts utilizing custom algorithms or software that are central to the research but not yet described in published literature, software must be made available to editors and reviewers. We strongly encourage code deposition in a community repository (e.g. GitHub). See the Nature Portfolio guidelines for submitting code & software for further information.

Data

Policy information about availability of data

All manuscripts must include a data availability statement. This statement should provide the following information, where applicable:

- Accession codes, unique identifiers, or web links for publicly available datasets
- A description of any restrictions on data availability
- For clinical datasets or third party data, please ensure that the statement adheres to our policy

All data supporting the findings of this study are available within the Article, Supplementary Information and Source Data file. The Source Data file contains the

underlying numerical data for all relevant main and Supplementary Figures. The oligonucleotide, crRNA, probe and target sequences used in this study are provided in the Supplementary Information. No custom code was developed or used in this study. Source data are provided with this paper.

Research involving human participants, their data, or biological material

Policy information about studies with human participants or human data. See also policy information about sex, gender (identity/presentation), and sexual orientation and race, ethnicity and racism.

Reporting on sex and gender	Sex was not considered as an experimental variable in the study design because this study was designed to evaluate the analytical performance of the pathogen-detection platform rather than sex-related biological differences. The study was not designed or statistically powered to detect sex-dependent differences in assay performance; therefore, no sex-disaggregated statistical analysis was performed. Gender identity was not collected and was therefore not analyzed.
Reporting on race, ethnicity, or other socially relevant groupings	Race, ethnicity and other socially relevant groupings were not available for the de-identified clinical samples and were therefore not analyzed.
Population characteristics	Not applicable. This study focused on analytical performance evaluation using de-identified clinical samples and did not involve population characteristic analysis.
Recruitment	Clinical specimens were obtained from collaborating institutions and volunteers. For de-identified residual clinical specimens, the requirement for individual informed consent was waived by the ethics committee. Samples were selected based on availability and predefined diagnostic information for analytical performance evaluation.
Ethics oversight	The collection and use of clinical samples complied with all relevant ethical regulations for research involving human participants. The study protocol was reviewed and approved by the Research Ethics Board of the University of Science and Technology Beijing (Approval No. 2025-4-154). All residual clinical specimens used in this study were fully de-identified before being provided to the research team. The requirement for individual informed consent was waived by the ethics committee. No compensation was provided to the sample donors.

Note that full information on the approval of the study protocol must also be provided in the manuscript.

Field-specific reporting

Please select the one below that is the best fit for your research. If you are not sure, read the appropriate sections before making your selection.

☒ Life sciences ☐ Behavioural & social sciences ☐ Ecological, evolutionary & environmental sciences

For a reference copy of the document with all sections, see nature.com/documents/nr-reporting-summary-flat.pdf

Life sciences study design

All studies must disclose on these points even when the disclosure is negative.

Sample size	No statistical method was used to predetermine sample size. Unless otherwise stated, analytical experiments were performed in three independent experiments to assess reproducibility. Each independent experiment used a separately prepared reaction mixture and an independently fabricated chip. Clinical sample sizes were determined by the number of eligible samples available during the study period.
Data exclusions	No data were excluded from the analyses.
Replication	Unless otherwise stated, analytical experiments were performed three times. Exact n values and whether the measurements represent independent or technical replicates are specified in the corresponding figure legends. All attempts at replication were successful.
Randomization	The experiments were not randomized. Samples were assigned to predefined experimental groups according to target identity, target concentration or reference RT-PCR classification.
Blinding	The investigators were not blinded to sample allocation or outcome assessment. Blinding was not applied because the experiments involved predefined target identities, control conditions and target-specific chip channels for analytical performance evaluation.

Reporting for specific materials, systems and methods

We require information from authors about some types of materials, experimental systems and methods used in many studies. Here, indicate whether each material, system or method listed is relevant to your study. If you are not sure if a list item applies to your research, read the appropriate section before selecting a response.

Materials & experimental systems

n/a	Involved in the study	
x		Antibodies
x		Eukaryotic cell lines
x		Palaeontology and archaeology
x		Animals and other organisms
	x	Clinical data
x		Dual use research of concern
x		Plants

Methods

n/a	Involved in the study	
x		ChIP-seq
x		Flow cytometry
x		MRI-based neuroimaging

Clinical data

Policy information about clinical studies

All manuscripts should comply with the ICMJE guidelines for publication of clinical research and a completed CONSORT checklist must be included with all submissions.

Clinical trial registration	Not applicable. This study was not a clinical trial and was therefore not registered.
Study protocol	Not applicable. This study was not a clinical trial; the analytical study design and experimental procedures are described in the Methods.
Data collection	Clinical samples were obtained from collaborating clinical institutions and volunteers for method validation. Residual clinical specimens were provided in a de-identified form. Volunteer samples were collected following ethical approval procedures.
Outcomes	The primary analytical outcomes were the limit of detection, decision time, sensitivity, specificity and agreement of the chip results with reference RT-PCR classifications. No clinical efficacy, treatment response or patient outcomes were assessed.

Plants

Seed stocks	*Report on the source of all seed stocks or other plant material used. If applicable, state the seed stock centre and catalogue number. If plant specimens were collected from the field, describe the collection location, date and sampling procedures.*
Novel plant genotypes	*Describe the methods by which all novel plant genotypes were produced. This includes those generated by transgenic approaches, gene editing, chemical/radiation-based mutagenesis and hybridization. For transgenic lines, describe the transformation method, the number of independent lines analyzed and the generation upon which experiments were performed. For gene-edited lines, describe the editor used, the endogenous sequence targeted for editing, the targeting guide RNA sequence (if applicable) and how the editor was applied.*
Authentication	*Describe any authentication procedures for each seed stock used or novel genotype generated. Describe any experiments used to assess the effect of a mutation and, where applicable, how potential secondary effects (e.g. second site T-DNA insertions, mosiacism, off-target gene editing) were examined.*
