# Supplementary material for: A CRISPR-based ‘dam-break drainage’ sensing architecture for rapid, affordable and ultrasensitive pathogen detection
Source: Nat Commun. 2026 Aug 22;17:11194. doi: 10.1038/s41467-026-77042-w (PMC13714288; doi:10.1038/s41467-026-77042-w)
Supplement: Supplementary file 2 [file 41467_2026_77042_MOESM12_ESM.pdf]

nature portfolio

Peer Review File

A CRISPR-based 'dam-break drainage' sensing architecture for rapid, affordable and ultrasensitive pathogen detection

Corresponding Author: Dr Yang Sun

This file contains all reviewer reports in order by version, followed by all author rebuttals in order by version.

A version of this paper was originally rejected for publication by Nature Communications, however that decision was reconsidered after appeal by the authors.

Version 0:

Reviewer comments:

Reviewer #1

(Remarks to the Author)
In this manuscript, Liu et al. report a CRISPR-Cas13a–based nucleic acid detection platform that converts target recognition into a visible “dam-break drainage” event via Cas13a-mediated interfacial wettability switching. By integrating a molecular dam composed of cleavable tri-block probes with a carefully engineered microfluidic architecture, the authors achieve instrument-free, visual detection with reported attomolar sensitivity and minute-scale assay times. Both single-plex and duplex detection modes are demonstrated, along with clinical validation using SARS-CoV-2, Influenza A, and Influenza B nasal swab samples. While the “dam-break drainage” assay is conceptually interesting, significant concerns remain regarding the attomolar sensitivity, minute-scale detection speed, mechanistic understanding, and experimental rigor.
Major Comments
1. The authors claim a detection sensitivity of 10 aM without pre-amplification using Cas13a within 2–3 min. However, prior studies have shown that amplification-free CRISPR diagnostics (including Cas12a and Cas13a) are fundamentally limited to picomolar sensitivity due to enzyme kinetics and background signal (Huyke DA et al., Analytical Chemistry, 2022, 94(27): 9826–9834). Cas13a-based assays typically require nucleic acid amplification or additional signal amplification strategies to achieve attomolar sensitivity. In this context, the manuscript lacks a tube-based benchmark (e.g., a conventional fluorescence reporter assay) performed under the same Cas13a/crRNA/buffer conditions across a concentration range of 10 aM–10 pM to enable a fair sensitivity comparison. Notably, the authors state that the “CRISPR-Cas13a system alone failed to discriminate between positive and negative samples,” further underscoring the need for quantitative in-tube controls using a standard fluorescence reporter. Therefore, the authors should provide a tube-based enzyme kinetic analysis with a conventional fluorescence reporter and determine the corresponding sensitivity.
2. The authors may attribute the ultrahigh sensitivity of their platform to CRISPR-Cas13a–mediated probe degradation; however, the accessibility of surface-anchored tri-block probes raises concerns compared with conventional tube-based CRISPR reactions. Covalent immobilization at the molecular dam, together with hydrophobic and sterically bulky moieties, may substantially restrict access to the Cas13a-cleavable RNA segment. This limitation is further exacerbated in the duplex configuration, where crRNAs are also tethered prior to 6U-mediated release. Immobilization of both the substrate and guiding RNA is expected to reduce the effective encounter rate with activated Cas13a, potentially lowering apparent catalytic efficiency. Although Fig. S14 demonstrates retained RNase activity of modified crRNAs, it does not quantitatively address kinetic constraints imposed by surface confinement. Comparative cleavage-kinetics measurements using surface-tethered versus free probes would help determine whether the reported attomolar sensitivity arises from enzymatic amplification or from structural instability of the dam.
3. While the authors highlighted the impressive sensitivity (e.g., 10aM) and speed (e.g., 2 min), the mechanistic basis of the claimed amplification-free performance in their platform is not sufficiently articulated. Specifically, clearer explanation of how the dam-break drainage readout and wettability switching provide effective signal amplification in the absence of nucleic acid pre-amplification would enable a fairer comparison with existing amplification-free CRISPR assays and help readers better appreciate the fundamental innovation of this work.
4. An additional uncertainty concerns molecular transport to the hydrophobic dam. The dam is designed to be strongly hydrophobic to resist wetting and retain large sample volumes, raising the question of how RNA targets and activated Cas13a in aqueous solution efficiently access immobilized probes at this interface. Because hydrophobic surfaces are typically depleted of aqueous solutes, enzyme–surface encounter rates may be intrinsically low, particularly at attomolar

concentrations. The manuscript does not address how this apparent mismatch between macroscopic hydrophobicity and microscopic enzymatic accessibility is resolved, nor whether cleavage is confined to a narrow interfacial region such as the pinned contact line.

Minor Comments

1. The blank (no-template control) amplification curve is missing in Supplementary Fig. 6 (RT-PCR curves).
2. Consistency in Ct value annotation across supplementary RT-PCR figures.

In several supplementary figures showing RT-PCR amplification curves (e.g., Supplementary Fig. 6 and related panels), Ct values are not explicitly annotated on the curves. For clarity and consistency, the authors are encouraged to label Ct values directly on the amplification plots, similar to the presentation style used in Supplementary Fig. 9.

3. In Supplementary Fig. 12 and other figures presenting statistical plots, it is unclear what individual data points represent (e.g., independent experiments, technical replicates, or averaged values). The authors should explicitly state the nature of the data points in the figure captions or methods.
4. For all figures that include statistical analysis, the authors should specify how error bars are. This information should be consistently reported either in the figure captions or in the Methods section.
5. Fig.3f and Fig.6f, the “Flod Changes” should be explained in the manuscript.

Reviewer #2

(Remarks to the Author)

Overall Summary:

This manuscript reports a visual nucleic acid chip based detection strategy that converts target nucleic acid recognition into a “dam break drainage” readout. The assay relies on Cas13a collateral RNase activity to induce a surface wettability switch at a molecular dam region, transforming it from hydrophobic to hydrophilic and thereby controlling whether the liquid can overcome the barrier and drain through microchannels. The authors claim aM sensitivity, short turnaround time, and low per-test cost, and they present validation on a relatively large set of clinical specimens, suggesting potential translational utility. However, from the standpoint of conceptual advance and mechanistic contribution, the central workflow closely parallels recently reported approaches in the same space. In its current form, the manuscript does not clearly articulate a substantive innovation beyond existing wettability-switching, fluid-motion readouts, nor does it provide sufficiently compelling data to support a meaningful mechanistic advance. Given Nature Communications high bar for originality and mechanistic insight, I do not find the level of innovation adequate for publication in this journal. On this basis, I recommend rejection.

Major Concern

1、The core approach uses Cas13a collateral cleavage to switch surface wettability and produce a naked-eye liquid-motion readout. This is conceptually very similar to a recent Science Advances single-tube liquid-motion assay (DOI: 10.1126/sciadv.adu2271), which also converts Cas13a-triggered wettability changes into instrument-free fluid movement. Beyond a different readout format, key performance metrics (time, cost, and LoD) appear broadly comparable. Thus, while this work improves chip design and enables dual-target detection, the advance is mainly in device/workflow optimization rather than a clear mechanistic breakthrough, and the incremental conceptual contribution is limited.

2、The manuscript lacks a systematic evaluation of sample preparation and matrix interference, and the RNase-related error risk requires stronger data support. Although the authors note that adding an RNase inhibitor can mitigate RNase contamination, key interference factors are not quantitatively assessed. The authors should perform a structured stability evaluation and report misclassification rates across conditions such as collection/storage

3、The study remains primarily qualitative, and multiplexing is demonstrated only at the duplex level. Without broader experimental evidence, the platform’s scalability and general applicability cannot be reliably assessed.

Minor Concerns:

1、Figure 1: The readout rule is not clearly defined. Please clarify whether 2 min is the optimized decision time, how delayed drainage is handled (especially at low copy), and what calls apply beyond the time window (negative/invalid/retest).

2、Figure 2(e): The parameter φ is not adequately defined or measured. Please define φ, describe the measurement method, and report fabrication tolerances/statistics to support reproducibility and sensitivity analysis.

3、Figure 5(c) proposes a 6U spacer on the crRNA to expand Cas13a’s effective cleavage range, but this is supported mainly by a schematic. Please add a with/without-spacer comparison and quantify the impact (e.g., trigger time, detection rate, or LOD).

4、Although the manuscript includes validation on a relatively large set of clinical specimens, several key conclusions rely primarily on representative images and schematics, without sufficient replication, error estimates, or clear statistical definitions (including the criteria used to define the limit of detection). The authors should strengthen the main performance claims with replicated measurements and appropriate statistical analysis.

5、The manuscript contains multiple English issues (grammar, word choice, typos, and formatting consistency). A thorough language edit is recommended.

Reviewer #3

(Remarks to the Author)

I co-reviewed this manuscript with one of the reviewers who provided the listed reports. This is part of the Nature Communications initiative to facilitate training in peer review and to provide appropriate recognition for Early Career Researchers who co-review manuscripts.

Version 1:

Reviewer comments:

Reviewer #1

(Remarks to the Author)
We appreciate the authors' efforts in providing additional experiments, which have addressed some of our previous comments. However, several major concerns, including the claimed attomolar sensitivity, minute-scale detection speed, and mechanistic understanding, remain insufficiently addressed.
The authors performed a tube-based enzyme kinetic analysis using a conventional fluorescence reporter and demonstrated a detection sensitivity in the picomolar range (e.g., 10 pM) (Supplementary Figures 8 and 20), which is consistent with the existing literature. In addition, they showed that CRISPR-Cas13a reactions using FAM-$(PEG6)_2$-6U-$(CH_2)_{12}$-BHQ and FAM–6U–BHQ reporters exhibited nearly identical fluorescence kinetic profiles (Supplementary Fig. 1), indicating that the $(PEG6)_2$-6U-$(CH_2)_{12}$-BHQ tri-block probe does not enhance the trans-cleavage activity of Cas13a. Therefore, the reported 10^6-fold improvement in sensitivity (from 10 pM to 10 aM) and the 30-fold reduction in detection time (from 60 minutes to 2 minutes) must be attributed entirely to the “Dam-Break Drainage” nucleic acid chip.
The claimed detection limit of 10 aM RNA corresponds to approximately 60 RNA copies. Based on both the published literature (e.g., Huyke DA et al., Analytical Chemistry, 2022, 94(27): 9826–9834) and the authors’ own tube-based kinetic data obtained with conventional fluorescence reporters, we are not convinced that such a small number of SARS-CoV-2 genomic RNA molecules can consistently diffuse to and activate sufficient Cas13a enzymes, resulting in enough cleavage of the immobilized probes to generate a reliable visual readout within only 2 minutes.
Furthermore, the authors have not clearly explained how the “dam-break drainage” readout and wettability-switching mechanism achieve such dramatic signal amplification without nucleic acid pre-amplification. The current experimental data do not adequately support this conclusion. Additional characterization at the single-molecule level is needed to validate the proposed amplification mechanism. For example, direct evidence of Cas13a-mediated degradation of the immobilized tri-block probes, as well as a quantitative correlation between molecular cleavage events and the resulting macroscopic drainage behavior, would substantially strengthen the mechanistic basis for the claimed performance. In the absence of such evidence, it remains unclear how a small number of target RNA molecules can trigger a sufficiently large wettability change to produce a robust and reproducible visual signal.

Reviewer #2

(Remarks to the Author)
No more comments.

Reviewer #3

(Remarks to the Author)
I co-reviewed this manuscript with one of the reviewers who provided the listed reports. This is part of the Nature Communications initiative to facilitate training in peer review and to provide appropriate recognition for Early Career Researchers who co-review manuscripts.

Version 2:

Reviewer comments:

Reviewer #1

(Remarks to the Author)
The authors have adequately addressed the reviewers’ comments.

Reviewer #3

(Remarks to the Author)
I co-reviewed this manuscript with one of the reviewers who provided the listed reports. This is part of the Nature Communications initiative to facilitate training in peer review and to provide appropriate recognition for Early Career Researchers who co-review manuscripts.

Open Access This Peer Review File is licensed under a Creative Commons Attribution 4.0 International License, which permits use, sharing, adaptation, distribution and reproduction in any medium or format, as long as you give appropriate credit to the original author(s) and the source, provide a link to the Creative Commons license, and indicate if changes were made.
In cases where reviewers are anonymous, credit should be given to 'Anonymous Referee' and the source.
The images or other third party material in this Peer Review File are included in the article's Creative Commons license, unless indicated otherwise in a credit line to the material. If material is not included in the article's Creative Commons license and your intended use is not permitted by statutory regulation or exceeds the permitted use, you will need to obtain permission directly from the copyright holder.
To view a copy of this license, visit https://creativecommons.org/licenses/by/4.0/

Reviewer 1

➢ Major Comment

Comment 1. The authors claim a detection sensitivity of 10 aM without pre-amplification using Cas13a within 2–3 min. However, prior studies have shown that amplification-free CRISPR diagnostics (including Cas12a and Cas13a) are fundamentally limited to picomolar sensitivity due to enzyme kinetics and background signal (Huyke DA et al., Analytical Chemistry, 2022, 94(27): 9826–9834). Cas13a-based assays typically require nucleic acid amplification or additional signal amplification strategies to achieve attomolar sensitivity. In this context, the manuscript lacks a tube-based benchmark (e.g., a conventional fluorescence reporter assay) performed under the same Cas13a/crRNA/buffer conditions across a concentration range of 10 aM–10 pM to enable a fair sensitivity comparison. Notably, the authors state that the "CRISPR-Cas13a system alone failed to discriminate between positive and negative samples," further underscoring the need for quantitative in-tube controls using a standard fluorescence reporter. Therefore, the authors should provide a tube-based enzyme kinetic analysis with a conventional fluorescence reporter and determine the corresponding sensitivity.

Response: We sincerely thank the reviewer for this very important and helpful comment. We fully appreciate the reviewer's concern regarding the claimed attomolar-level sensitivity without nucleic acid pre-amplification. As you suggested, in the revised manuscript, we performed tube-based enzyme kinetic analyses using a standard fluorescent RNA reporter, with SARS-CoV-2 N, IAV PB2 and IBV PB1 as model targets over a concentration range from 10 aM to 1 nM, under the same Cas13a/crRNA/buffer conditions as those used in the chip experiments. The additional results consistently showed that the detection sensitivity of the pure CRISPR-Cas13a system remained at the pM level and did not reach the aM-level sensitivity achieved by the chip platform.

The corresponding modification has been made:

1.Page 10, SI

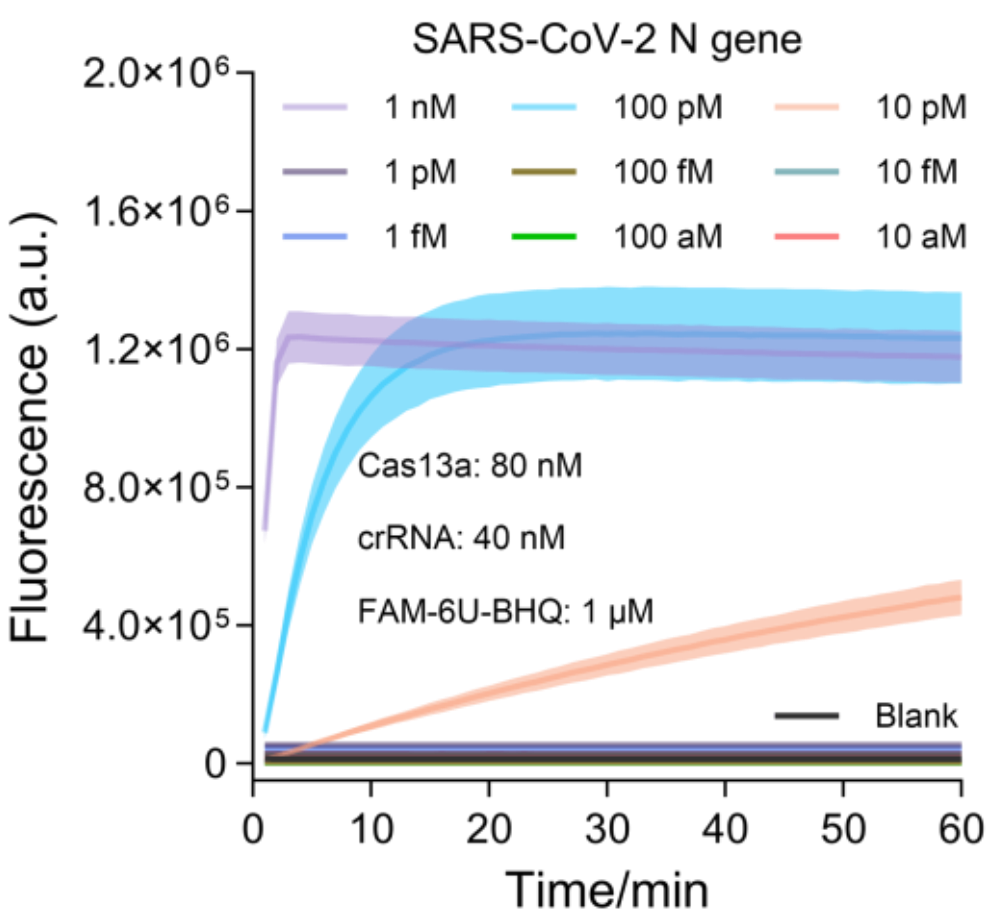

Supplementary Fig. 8. Sensitivity of the pure CRISPR–Cas13a system for SARS-CoV-2 N gene detection. Solid lines indicate the mean fluorescence intensity from three independent experiments, and shaded areas denote

the error band. For SARS-CoV-2 N gene concentrations ranging from 10 aM to 1 pM, the fluorescence signal traces were comparable to the Blank control.

Was added

2.Page 22, SI

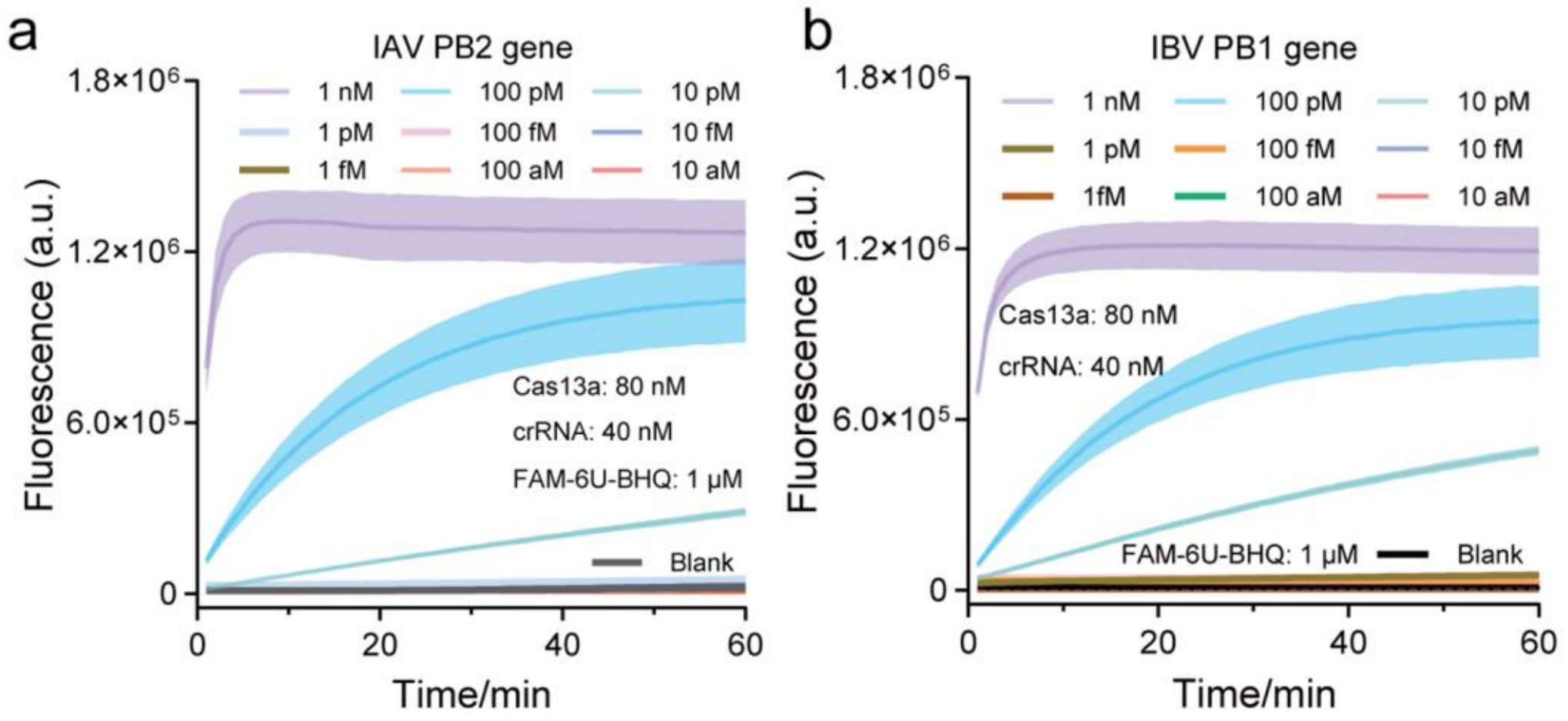

Supplementary Fig. 20. Sensitivity of the pure CRISPR–Cas13a system in detecting the IAV-PB2 gene (a), IBV-PB1 gene (b). Solid lines represent the mean fluorescence intensity from three independent experiments, and the shaded areas represent the variation across the three measurements. In the CRISPR–Cas13a systems containing IAV-PB2 or IBV-PB1 RNA targets at concentrations ranging from 10 aM to 1 pM, real-time fluorescence signals were indistinguishable from the Blank control.

Was added

3.Page 17, SI

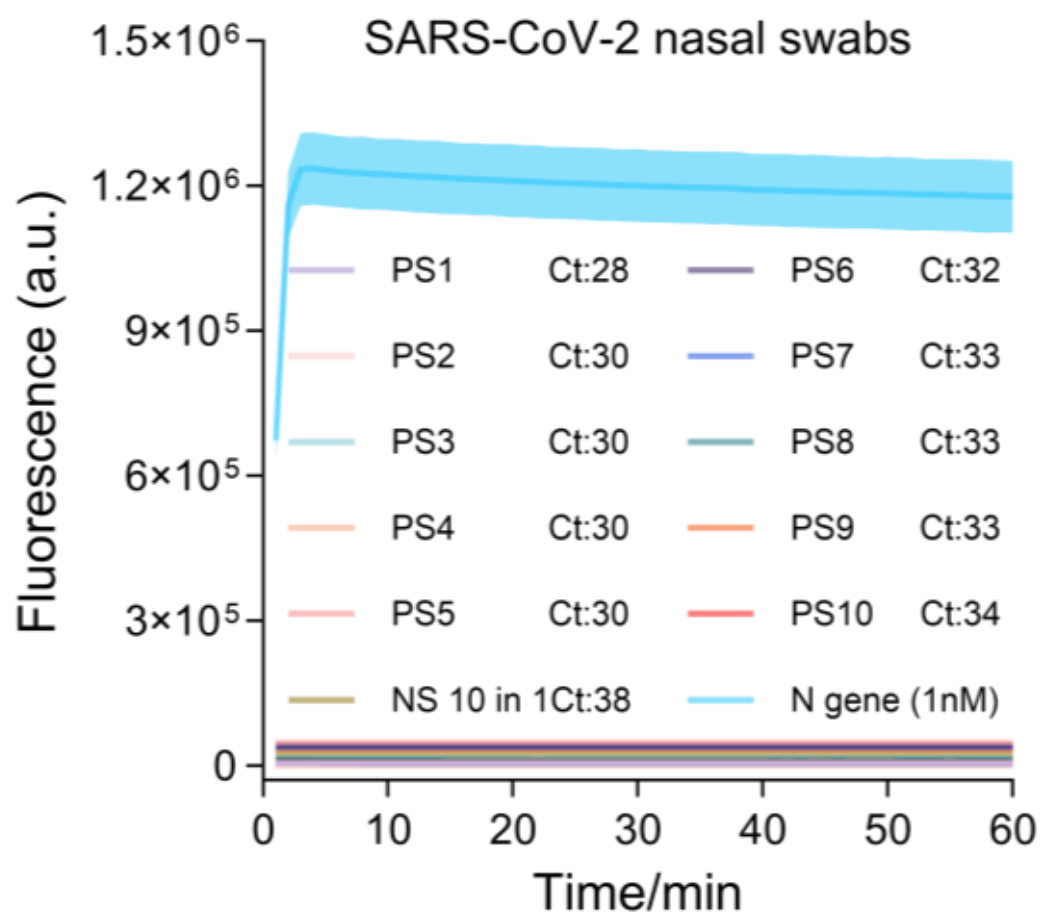

Supplementary Fig. 15. Sensitivity of the pure CRISPR-Cas13a system in detecting real samples. 10 SARS-CoV-2 RT-PCR positive nasal swabs with Ct values range from 28 to 34 and SARS-CoV-2 RT-PCR negative 10 in 1 sample with Ct values 38, were tested. Solid lines represent the mean fluorescence intensity from three independent experiments, and shaded areas indicate the error band of three independent measurements. 1 nM SARS-CoV-2 N gene was used as the control group.

Was added

4.Page 26, SI

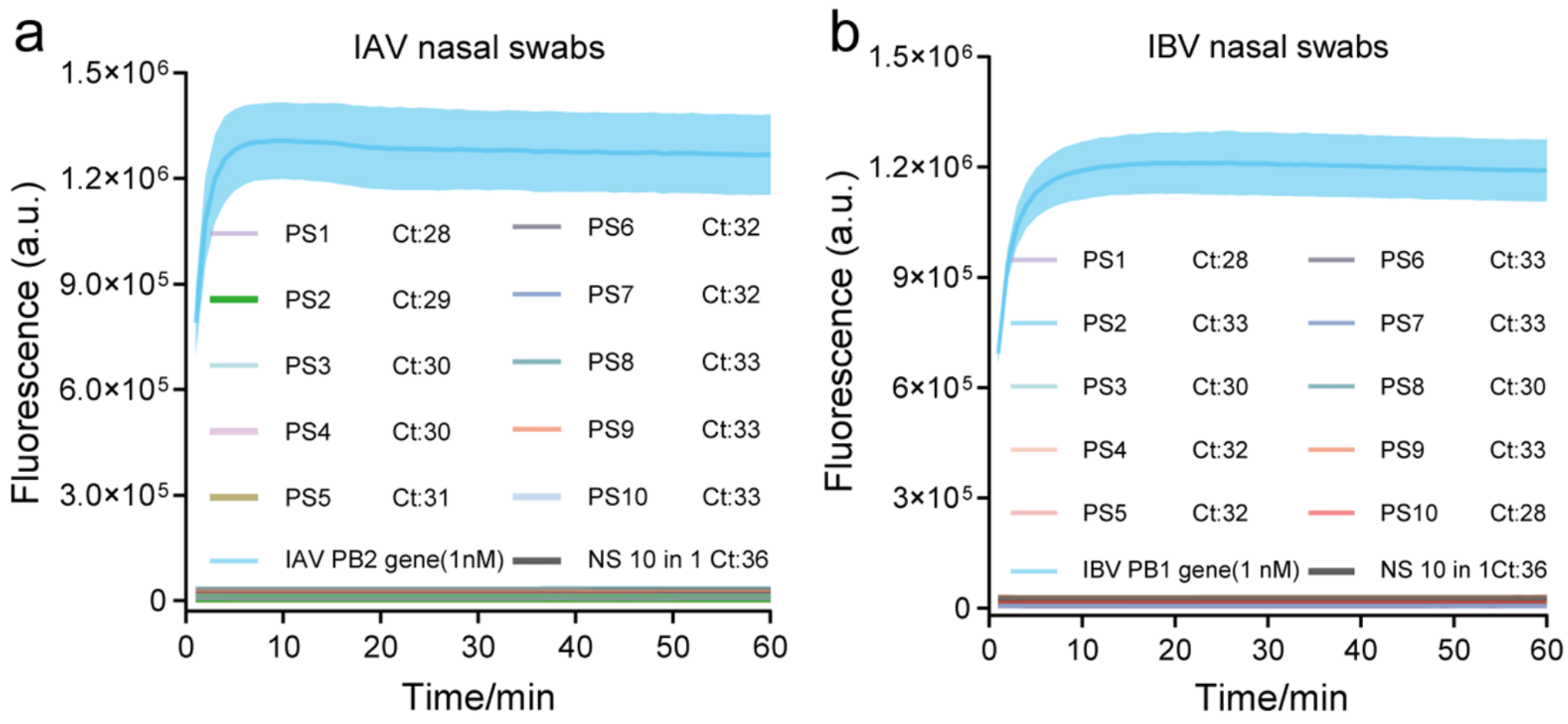

Supplementary Fig. 24. Sensitivity of the pure CRISPR-Cas13a system in detecting IAV nasal swabs and IBV nasal swabs. Real-time fluorescence responses of IAV-positive nasal swab samples (**a**), IBV-positive nasal swab samples (**b**) and negative 10 in 1 pooled nasal swab samples. The IAV-positive and IBV-positive samples had Ct values ranging from 28 to 34, whereas the negative pooled samples had Ct values of 36. 1 nM IAV-PB2 and IBV-PB1 RNA targets served as controls for their corresponding assays. Solid lines represent the mean fluorescence intensity from three independent experiments, and shaded areas indicate the error band of three independent measurements.

Was added

5. Page 9, lines 238-245

As shown in Fig. 3b, Fig. S5 and Movie S1, the chip enabled rapid (within 2 min), highly sensitive detection of the SARS-CoV-2 N gene over a wide concentration range (1 aM-10 pM), with a limit of detection (LOD) of 10 aM. Compared with the conventional RT-PCR assay, the developed chip achieves both a lower detection limit and a faster readout, whereas RT-PCR fails to detect the N gene at 10 aM, yielding Ct values that overlap with the blank (Fig. S6).

Was replaced by

As shown in Fig. 3b and Movie S1, the chip enabled rapid and highly sensitive detection of the SARS-CoV-2 N gene over a wide concentration range (1 aM–10 pM), with a limit of detection (LOD) of 10 aM. In three independent measurements (n=3), all tested positive groups consistently triggered visible “dam-break drainage” event within 2 min, whereas blank samples remained confined throughout the 10 min observation window, supporting the single-plex visual readout rule of rapid decision time (Fig. S6). As shown in Fig. S7 and Fig. S8, the developed chip outperforms both commercial RT-PCR kits (100 aM, 35 min) and pure CRISPR-Cas13a technology (10 pM, 40 min), exhibiting a lower detection limit and a faster readout.

6. Page 11, lines 286-289

Impressively, the single-plex chip sensitively identified positive samples with Ct values up to 35, while the pure CRISPR-Cas13a system failed to distinguish any positive samples from negative samples, even Ct values down to 28.

Was replaced by

Remarkably, the single-plex chip enabled reliable identification of positive samples with Ct values up to 35, while the pure CRISPR-Cas13a system failed to discriminate positive samples from negative samples, even for positive sample with Ct values down to 28 (Fig. S15).

7. Page 16, lines 421-429

As shown in Fig. 6c, S15 and Movie S7, when IAV-PB2 and IBV-PB1 RNA targets were mixed at equal concentrations within the range of 10 aM to 10 pM, the duplex chip successfully distinguished the two targets independently within 5 min, maintaining a detection limit of 100 aM. The detection performance of the duplex chip rivals that of conventional RT-PCR assays (Fig. S16 and Fig. S17).

Was replaced by

As shown in Fig. 6c and Movie S7, the duplex chip allowed IAV-PB2 and IBV-PB1 RNA targets to be simultaneously detected when they were mixed at equal concentrations, with an LOD of 100 aM. In three independent measurements (n=3), each positive group consistently generated visible "dam-break drainage" event within the 5 min readout period, while blank sample showed no "dam-break drainage" event and remained confined during the 10 min observation window, validating the duplex visual interpretation rule of 5 min rapid decision time (Fig. S17). The sensitivity of the duplex chip rivals that of conventional RT-PCR assays (Fig. S18 and Fig. S19) and vastly surpass the pure CRISPR-Cas13a system (Fig. S20). Moreover, the decision time of the developed duplex chip is 5 min, which is much shorter than that of the compared two methods.

8. Page 16, lines 444-447

As summarized in Fig. 6f and Fig. S18a, both RT-PCR and our duplex chip assay correctly identified all IAV-positive, IBV-positive and negative samples, while the duplex chip delivering results in a significantly shorter time. In contrast, the CRISPR-Cas13a system alone failed to discriminate between positive and negative samples.

Was replaced by

As summarized in Fig. 6f, both RT-PCR and our duplex chip assay correctly identified all IAV-positive, IBV-positive and negative nasal swab samples, while the duplex chip delivering results in a significantly shorter time. In contrast, the CRISPR-Cas13a system alone failed to distinguish RT-PCR-positive from RT-PCR-negative nasal swab samples of both IVA and IBV (Fig. S24).

Comment 2. The authors may attribute the ultrahigh sensitivity of their platform to CRISPR-Cas13a–mediated probe degradation; however, the accessibility of surface-anchored tri-block probes raises concerns compared with conventional tube-based CRISPR reactions. Covalent immobilization at the molecular dam, together with hydrophobic and sterically bulky moieties, may

substantially restrict access to the Cas13a-cleavable RNA segment. This limitation is further exacerbated in the duplex configuration, where crRNAs are also tethered prior to 6U-mediated release. Immobilization of both the substrate and guiding RNA is expected to reduce the effective encounter rate with activated Cas13a, potentially lowering apparent catalytic efficiency. Although Fig. S14 demonstrates retained RNase activity of modified crRNAs, it does not quantitatively address kinetic constraints imposed by surface confinement. Comparative cleavage-kinetics measurements using surface-tethered versus free probes would help determine whether the reported attomolar sensitivity arises from enzymatic amplification or from structural instability of the dam.

Response: We sincerely thank the reviewer for this highly professional and insightful comment. We fully agree with the reviewer's central concerns: (1) compared with the conventional FAM-6U-BHQ fluorescent reporter, the hydrophilic and hydrophobic moieties flanking the $(PEG_6)_2$-6U-$(CH_2)_{12}$H tri-block probe may affect the accessibility of Cas13a to the central 6U RNA-responsive segment; and (2) immobilization of both the tri-block probes and the guiding RNA may reduce the effective encounter rate with activated Cas13a, thereby lowering the catalytic efficiency.

To address concern (1), we synthesized a fluorescent tri-block reporter, FAM-$(PEG_6)_2$-6U-$(CH_2)_{12}$-BHQ, and performed a comparative experiment under otherwise identical CRISPR-Cas13a reaction conditions, with one group using the conventional FAM-6U-BHQ reporter and the other using the FAM-$(PEG_6)_2$-6U-$(CH_2)_{12}$-BHQ reporter. As a results, the two groups exhibited nearly identical fluorescence kinetic profiles, indicating that the $(PEG_6)_2$-6U-$(CH_2)_{12}$H tri-block architecture itself does not block Cas13a access to the central RNA-responsive segment (the added Fig.S1 in revised manuscript). We are grateful for this comment, as it prompted us to examine more carefully the accessibility of Cas13a to the $(PEG_6)_2$-6U-$(CH_2)_{12}$H tri-block probe and has helped improve the scientific rigor and logical clarity of the manuscript.

To address concern (2), we first note that in the present chip system, the effective surface concentrations of the immobilized tri-block probes and crRNA on the molecular dam are difficult to precisely control and determine. Therefore, we believe that a strict real-time kinetic comparison between surface-tethered tri-block probe/crRNA and their fully free counterparts is experimentally difficult to achieve in the current system, and at present we have not identified a sufficiently rigorous experimental design for such a direct comparison.

Nevertheless, to respond to the reviewer's concern as directly as possible, we added a more feasible control experiment in the revised manuscript by directly comparing surface-immobilized crRNAs without a 6U linker and with a 6U linker. To make this comparison as close as possible to the actual chip working state, we did not perform the experiment in free solution. Specifically, the two types of crRNA were first immobilized on glass surfaces using the same protocol as that used for the chip, and the resulting substrates were then cut into 6.5 mm circular pieces to fit a 96-well plate for fluorescence monitoring. During measurement, all CRISPR-Cas13a reaction components were kept identical except for the configuration of the surface-immobilized crRNA. The glass pieces carrying crRNA without the 6U linker or with the 6U linker were then introduced into two

corresponding reaction systems, and cleavage of the FAM-$(PEG_6)_2$-6U-$(CH_2)_{12}$-BHQ fluorescent reporter was monitored in real time using a microplate reader, with three replicates for each group.

Through this design, we were able to directly compare the target-triggered molecular cleavage efficiency of the two crRNA configurations under surface-immobilized conditions, rather than merely comparing their enzyme activity in free solution. The supplementary results showed that, compared with the linker-free design, introduction of the 6U linker markedly improved Cas13a-mediated molecular cleavage efficiency in the surface-confined system. In our view, this improvement does not simply indicate that the modified crRNA retains basic activity; rather, it reflects a difference in post-activation spatial behavior between the two configurations. In the linker-free design, the crRNA remains anchored on the glass surface and thus the activated Cas13a–crRNA complex remains confined near its original location and can act only within the immediate vicinity. By contrast, in the design containing a 6U linker, this linker can be rapidly cleaved by activated Cas13a after target recognition, thereby releasing the originally localized Cas13a–crRNA complex to gain greater spatial freedom and degrade hydrophobic molecular-dam probes located farther away. In other words, introduction of the 6U linker effectively enlarges the cleavage range of activated Cas13a in the surface-confined system, thereby improving the overall target-triggered cleavage efficiency.

We believe that this additional experiment directly addresses an important mechanistic point: surface-immobilized crRNA can indeed recruit Cas13a, be efficiently activated by the target, and mediate robust cleavage of the FAM-$(PEG_6)_2$-6U-$(CH_2)_{12}$-BHQ fluorescent reporter. Moreover, the activity of this surface-confined system can be further enhanced by introducing a 6U linker into the crRNA design.

We agree with the reviewer that, relative to a free-solution reaction, surface confinement may reduce the apparent encounter rate and local catalytic efficiency. However, the newly added control experiment indicates that such confinement does not abolish target-triggered cleavage activity. The proposed aM system-level sensitivity of the chip arises not from unrestricted enzymatic amplification in a free molecular environment, but from coupling the retained Cas13a collateral cleavage activity with the “dam-break drainage” event architecture operated near a critical threshold. Under this design, a limited number of molecular cleavage events can be converted into a pronounced macroscopic “dam-break drainage” event.

At the same time, we would also like to clarify that the original manuscript already provided multiple layers of evidence showing that the molecular dam does not undergo spontaneous “dam-break drainage” event in the absence of target. First, in the working principle section, we explicitly stated that, in the absence of target, the liquid remains stably confined within the sample reservoir and only breaches the dam after target recognition. Second, during structural optimization, we systematically examined the geometric parameter window of the dam and found that only within an appropriate structural range could the system establish a critical threshold state that is stable in the negative state yet triggerable upon target recognition, rather than a dam structure that fails randomly. Furthermore, in the duplex validation, Fig. 6b and Movies S4-S6 show that, in the absence of targets,

the liquid remained confined within the sample reservoir with no observable flow. In the clinical tests, negative samples still showed no “dam-break drainage” event even after 10 min. Likewise, in pooled testing, the negative pool remained entirely restricted to the sample reservoir. The single-plex results were fully consistent with these observations, with negative readouts always showing liquid retention in the sample reservoir. Taken together, these results from structural optimization, negative samples, negative pooled samples and supplementary videos support that the observed “dam-break drainage” event does not arise from nonspecific structural instability of the molecular dam itself, but from target-activated Cas13a-mediated tri-block probe degradation coupled with the critical dam structure.

Again, we sincerely thank the reviewer for this rigorous and constructive suggestion. In response, we have added feasible control experiments in the revised manuscript and further revised the relevant text to more clearly distinguish the cleavage capability of the free molecular system, the kinetic constraints potentially introduced by surface immobilization, and the true origin of the chip’s system-level sensitivity. We believe that these additions and revisions substantially strengthen the rigor and clarity of our mechanistic argument.

The corresponding modification has been made:

1.Page 3 SI

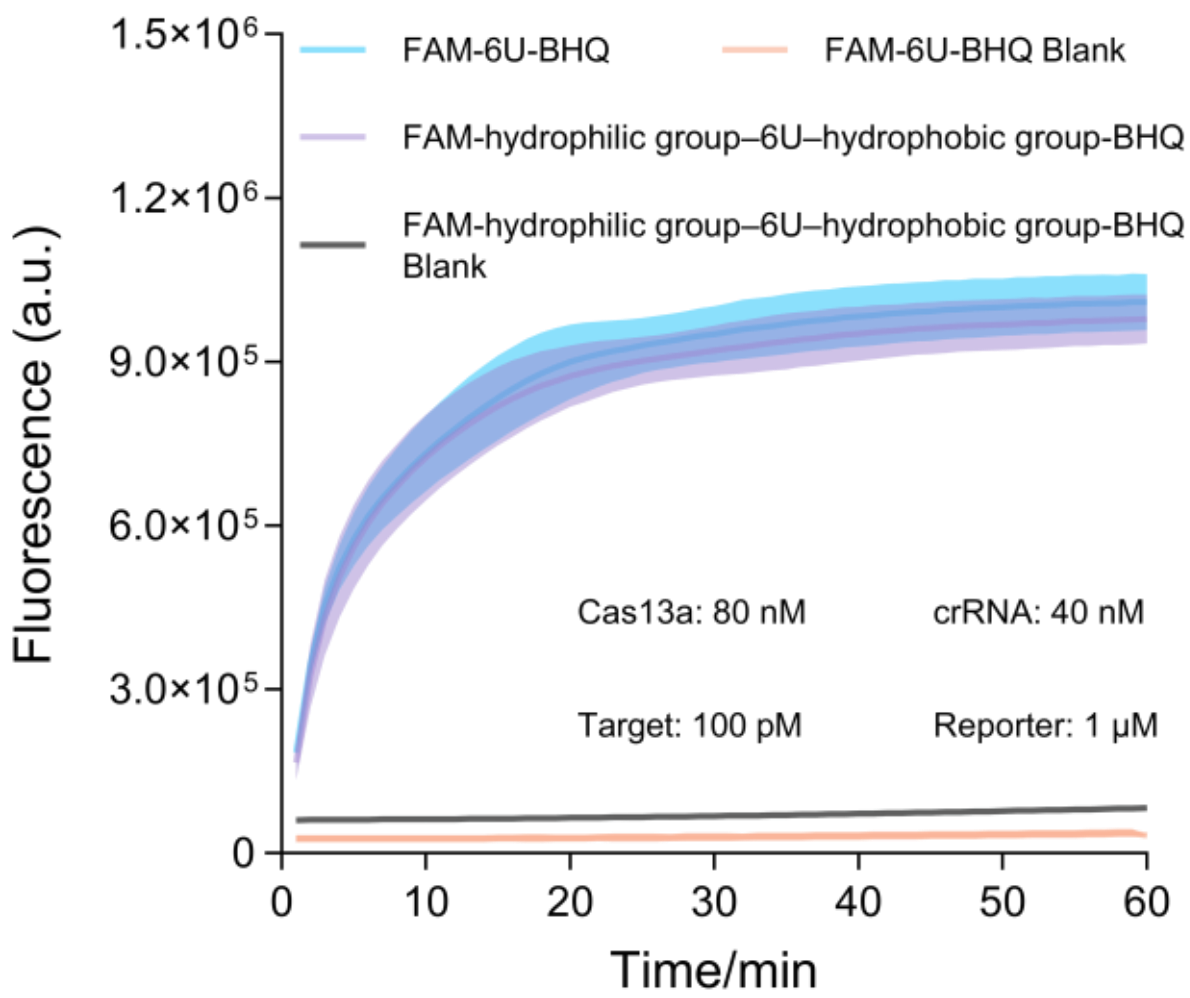

Supplementary Fig. 1. Collateral kinetics comparison of CRISPR-Cas13a to FAM-$(PEG_6)_2$-6U-$(CH_2)_{12}$-BHQ and FAM–6U–BHQ reporters. Solid lines represent the mean fluorescence intensity from three independent experiments, and shaded areas indicate the variation range among the three measurements.

Was added

2.Page 4, lines 114-120

To examine whether the central RNA-responsive segment in the tri-block probe might be less accessible to Cas13a due to the flanking hydrophobic module and possible steric hindrance, we performed parallel control experiment while keeping all other components of the CRISPR-Cas13a system unchanged. One group used the conventional FAM-6U-BHQ fluorescent reporter, whereas the another one used the FAM-$(PEG_6)_2$-6U-$(CH_2)_{12}$-BHQ fluorescent reporter. The two groups

exhibited nearly identical fluorescence kinetic profiles, indicating that the $(PEG_6)_2$-6U-$(CH_2)_{12}$H tri-block architecture itself does not impair Cas13a access to the central RNA-responsive segment (Fig. S1).

Was added

3.Page 15, lines 377-409

Although this strategy successfully ensures specificity, it introduces a new limitation: once the CRISPR-Cas13a complex is immobilized on the molecular dam, the activated Cas13a proteins are spatially confined and can only degrade the nearby tri-block probes (Fig. 5cI). This constraint significantly weakens the overall degradation efficiency and hampers the wettability transition of the dam surface, thereby limiting the detection sensitivity. To overcome this, we further optimized the crRNA design by inserting a Cas13a-responsive linker (6U) between the amino group and the crRNA sequence (Fig. 5cII). Upon target recognition, the activated Cas13a cleaves this linker, transiently releasing the local Cas13a-crRNA complex into a quasi-free state. This allows the activated Cas13a to degrade a broader range of the tri-block probes surrounding the molecular dam, resulting in a more efficient wettability transition and markedly enhanced detection sensitivity.

Was replaced by

Although this strategy successfully ensures specificity, it arise two questions: (1) whether the amino modified crRNA will decrease the ability of Cas13a collateral efficiency; (2) How to improve the degradation efficiency of the immobilized CRISPR-Cas13a. Once the CRISPR-Cas13a complex is immobilized on the molecular dam, the activated Cas13a proteins are spatially confined and can only degrade nearby tri-block probes, which weakens the overall degradation efficiency and hampers the wettability transition of the dam surface. To overcome this, we further optimized the crRNA design by inserting a Cas13a-responsive linker (6U) between the amino group and the crRNA sequence (Fig. 5cI). Then, we conducted the comparison experiments to evaluate how the amino-crRNA without the 6U linker and the amino-6U-crRNA containing the 6U linker affect the Cas13a collateral efficiency using the conventional crRNA as reference. We first compared these three crRNA designs in free solution. Results from three independent experiments showed that the three crRNA types exhibited essentially the same Cas13a collateral efficiency in free solution (Fig. 5cII). This indicates that amino modification of the crRNA does not impair Cas13a collateral efficiency, and that introduction of the 6U linker also does not affect Cas13a collateral efficiency. We then further examined how surface-immobilized amino-crRNA without the 6U linker and surface-immobilized amino-6U-crRNA containing the 6U linker affect Cas13a collateral efficiency. To make this comparison consistent with the actual chip-working condition, we used the same glass substrate and immobilization procedure as those used in the chip system. Specifically, the two crRNA designs were immobilized onto glass surfaces using the same method as in chip fabrication. The modified glass substrates were then cut into small disks with a diameter of 6.5 mm to fit a 96-well microplate reader. During the assay, all other components of the CRISPR–Cas13a reaction system were kept the same, and only the surface-immobilized crRNA design differed between the

two groups. The glass disks carrying immobilized crRNA without spacer or with spacer were added into the corresponding reaction solutions, and the fluorescence reporter-cleavage signal was monitored in real time using a microplate reader. Each group included three independent experiments. Through this design, we were able to directly compare Cas13a collateral efficiency of the two crRNA configurations under surface-immobilized conditions, rather than simply comparing their enzymatic activity in free solution. The results showed that the introduction of the 6U linker significantly increased molecular cleavage efficiency under surface-anchored conditions (Fig. 5cIII). Mechanistically, in the linker-free design, the activated Cas13a/crRNA complex remains locally tethered after target recognition and therefore mainly acts on nearby tri-block probes (Fig. 5cIV). In contrast, in the 6U-containing design, the activated Cas13a rapidly cleaves the linker, thereby releasing the Cas13a/crRNA complex from this confined local state and enabling degradation of more distant tri-block probes (Fig. 5cV). Thus, we selected the amino-6U-crRNA as the optimized crRNA configuration for the subsequent duplex chip experiments.

4.Page 12, line 309

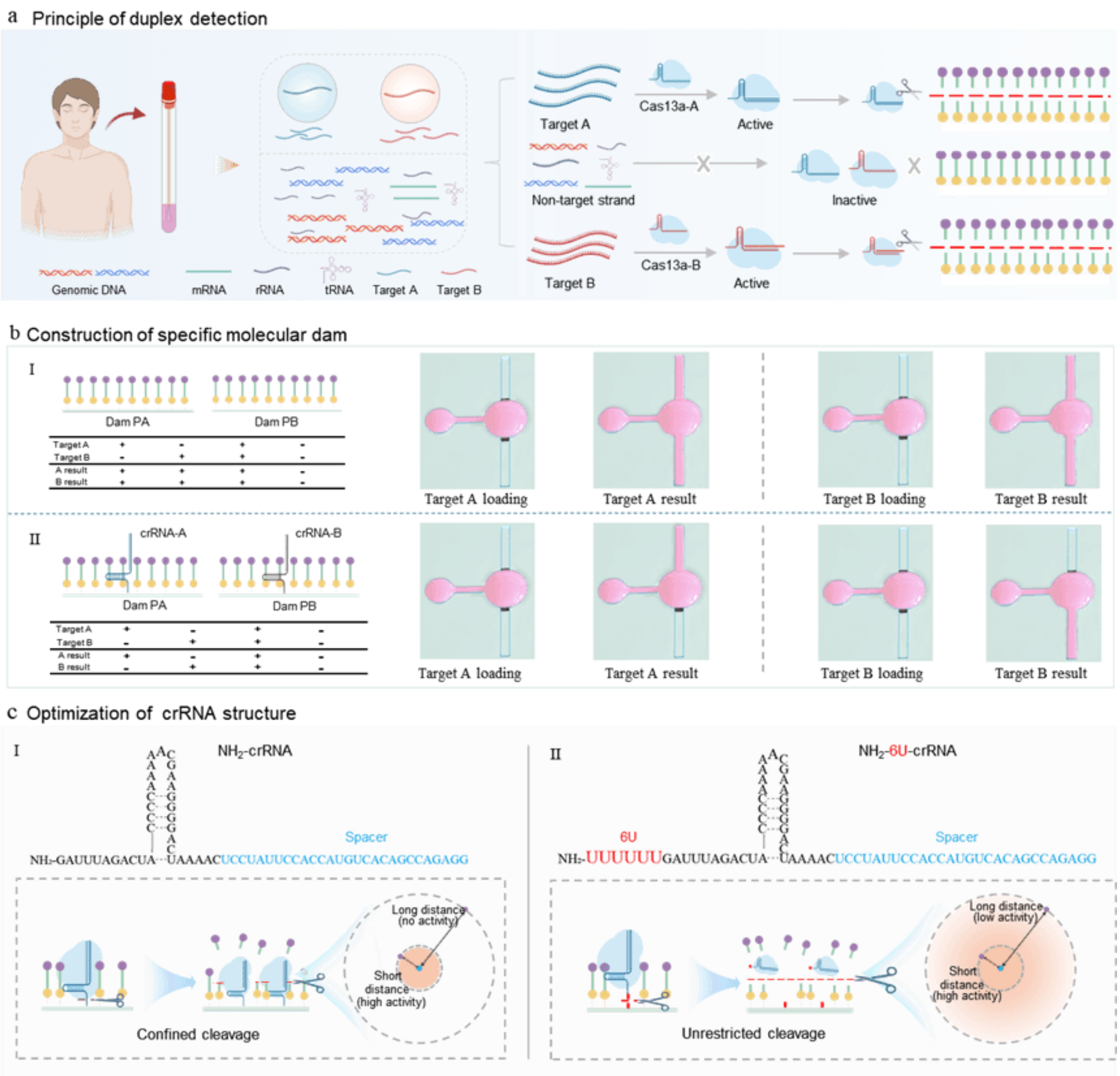

Was replaced by

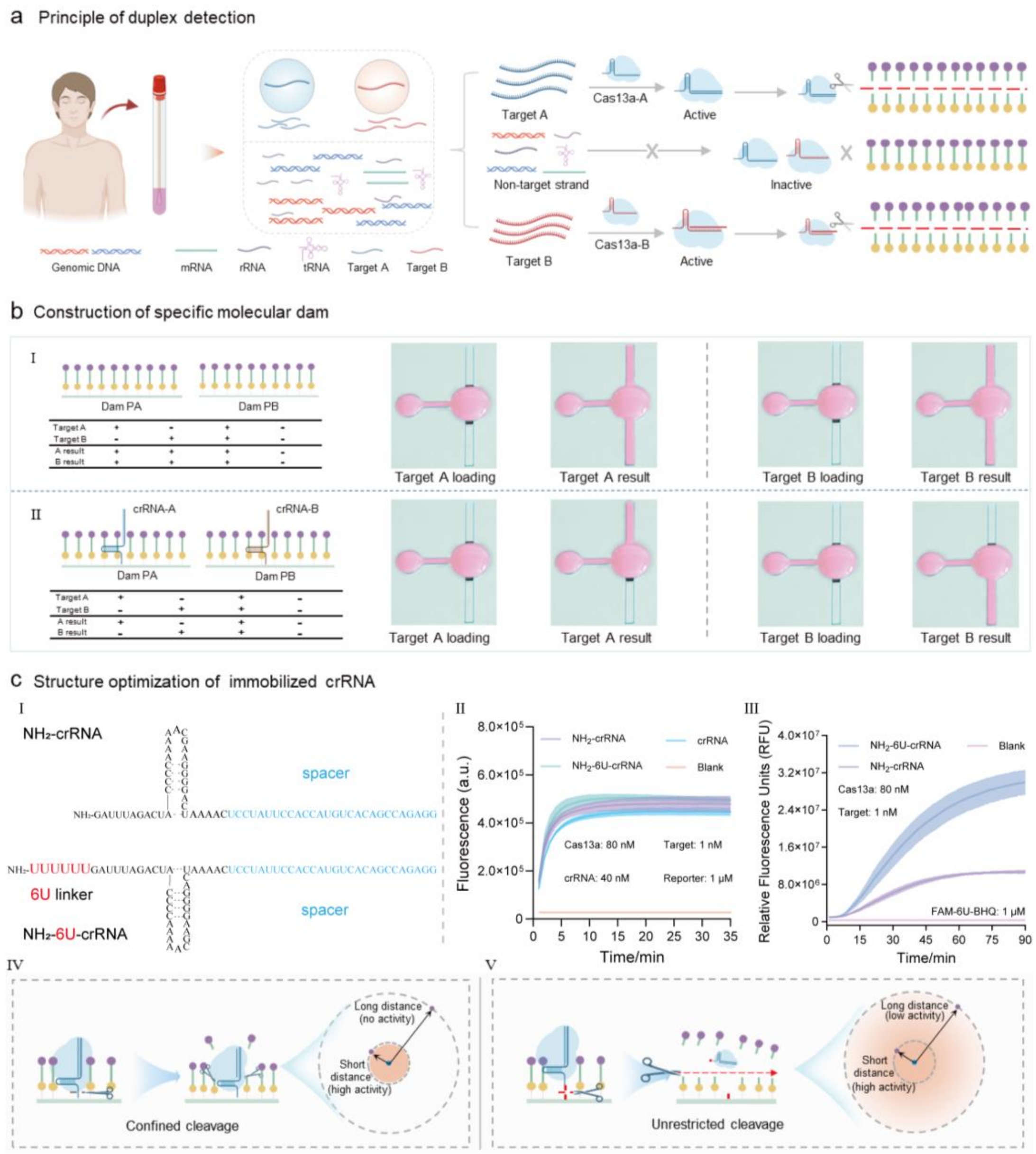

5.Page 12, lines 310-323

Figure 5 | Engineering specific molecular dams and optimizing crRNA architecture for duplex detection. (a) Working principle: two target RNAs orthogonally activate their respective CRISPR-Cas13a systems, leading to the specific cleavage of their corresponding tri-block probes on the molecular dams. **(b)** Formation of specific molecular dams: A non-specific dam, constructed from tri-block probes alone, is susceptible to be break by any activated Cas13a, leading to potential false-positive signals **(bI)**. A specific molecular dam, incorporating both tri-block probes and matched crRNA, ensures that drainage occurs only upon recognition of the intended target, thereby conferring specificity **(bII)**. **(c)** Overcoming spatial confinement of Cas13a. Direct immobilization of the crRNA anchors the activated Cas13a protein, limiting its collateral cleavage to adjacent tri-block probes **(cI)**. Engineering a 6U spacer that acts as a flexible tether between the amino group and crRNA allows partial mobility after Cas13a activation, thereby expanding the effective cleavage radius and enhancing tri-block probe degradation **(cII).**

Was replaced by

Figure 5 | Engineering specific molecular dams and optimizing crRNA architecture for duplex detection. (a) Working principle: two target RNAs orthogonally activate their respective CRISPR-Cas13a systems, leading to the specific cleavage of their corresponding tri-block probes on the molecular dams. (**b**) Formation of specific molecular dams: A non-specific dam, constructed from tri-block probes alone, is susceptible to be break by any activated Cas13a, leading to potential false-positive signals (**bI**). A specific molecular dam, incorporating both tri-block probes and matched crRNA, ensures that drainage occurs only upon recognition of the intended target, thereby conferring specificity (**bII**). (**c**) crRNA design for duplex chip. (**cI**) Structures of the two crRNA designs: amino-modified crRNA and amino-6U-modified crRNA. (**cII**) Comparison of Cas13a collateral kinetics between amino-crRNA and amino-6U-crRNA in free solution. (**cIII**) Comparison of activated Cas13a-mediated cleavage efficiency between amino-crRNA and amino-6U-crRNA under surface-anchored conditions. Three independent kinetic traces are shown for each condition. The solid lines show the average fluorescence intensity obtained from three independent experiments, whereas the shaded areas denote the error band. Schematic comparison of linker-free and 6U-linker crRNA designs for duplex chip construction, showing that the linker-free design confines the activated Cas13a complex near the anchoring site (**cIV**), whereas the 6U-linker design allows activation-triggered release of the Cas13a/crRNA complex, thereby extending its effective cleavage range (**cV**).

Comment 3. While the authors highlighted the impressive sensitivity (e.g., 10aM) and speed (e.g., 2 min), the mechanistic basis of the claimed amplification-free performance in their platform is not sufficiently articulated. Specifically, clearer explanation of how the "dam-break drainage" event readout and wettability switching provide effective signal amplification in the absence of nucleic acid pre-amplification would enable a fairer comparison with existing amplification-free CRISPR assays and help readers better appreciate the fundamental innovation of this work.

Response: We sincerely thank the reviewer for this important and insightful comment. We also apologize that the mechanistic basis for the high sensitivity and rapid readout was not presented with sufficient clarity in the original manuscript. We would like to clarify that the reported 10 aM sensitivity and approximately 2 min readout do not arise from conventional CRISPR signal generation. In typical CRISPR assays, reporter cleavage produces a fluorescence signal that accumulates gradually over time. In contrast, in our platform, Cas13a-mediated cleavage is not the final readout. It serves only as a trigger. Once activated by the target, Cas13a cleaves the RNA-responsive segment in the tri-block probe. This disrupts the molecular units that maintain the hydrophobicity of the dam surface. As a result, a local wettability transition from hydrophobic to hydrophilic is induced at the interface. The key design feature is that the droplet is held in a stable but near-threshold confined state (Fig.2). Under this condition, the system is highly sensitive to local perturbation. Extensive probe degradation is not required. Instead, limited local cleavage is sufficient to break the force balance and trigger a rapid macroscopic dam-break drainage event. Therefore, the performance of this platform does not rely on signal accumulation. It arises from the coupling of localized CRISPR-mediated cleavage with a threshold-triggered physical response at the chip level. In essence, this work converts a microscopic molecular event into a macroscopic liquid readout through interfacial wettability switching. Following the reviewer's suggestion, we have revised the manuscript to present this mechanism more clearly.

The corresponding modification has been made:

1.Page 6, lines 140-146

To maximize the chip′s sensitivity, it is essential to engineer a structural design with intrinsic signal amplification capability. We aimed to establish a critical state where a liquid droplet—much larger in volume than the underlying sample reservoir—remains stable at the drainage threshold. This configuration makes it susceptible to a "dam-break drainage" event triggered by even slight degradation of the tri-block probes. We realized this concept by designing a hydrophobic E region and a hydrophobic B region to confine the oversized droplet and prevent it from overflowing or collapsing beyond the A region.

Was replaced by

To improve chip sensitivity, we aimed to create a threshold-confined droplet that remained stably restricted in the sample reservoir while being readily triggered by target-induced probe cleavage. In this design, a droplet much larger than the underlying reservoir was maintained near the drainage threshold, allowing limited degradation of the tri-block probes to induce a clear "dam-break drainage" readout. To achieve this, both the E region and the B region were designed to be hydrophobic, so that the oversized droplet could be effectively confined without premature overflow or collapse beyond the A region.

Comment 4. An additional uncertainty concerns molecular transport to the hydrophobic dam. The dam is designed to be strongly hydrophobic to resist wetting and retain large sample volumes, raising the question of how RNA targets and activated Cas13a in aqueous solution efficiently access immobilized probes at this interface. Because hydrophobic surfaces are typically depleted of aqueous solutes, enzyme–surface encounter rates may be intrinsically low, particularly at attomolar concentrations. The manuscript does not address how this apparent mismatch between macroscopic hydrophobicity and microscopic enzymatic accessibility is resolved, nor whether cleavage is confined to a narrow interfacial region such as the pinned contact line.

Response: We sincerely thank the reviewer for raising this very thoughtful and important mechanistic question. We agree that, if one starts from the macroscopic impression of a "strongly hydrophobic surface," it is natural to further question whether the target RNA and Cas13a in the aqueous phase can readily access the dam surface. We also acknowledge that, in the original manuscript, we did not sufficiently distinguish between two different levels of description: the macroscopic anti-wetting behavior of the dam and the microscopic accessibility of the liquid–solid interface. As a result, this point was not explained clearly enough.

What we would like to clarify is that, in the present system, the "hydrophobicity" of the dam describes the interfacial energy barrier that resists forward liquid spreading and liquid crossing over the dam. It does not mean that the liquid is isolated from, or does not contact, the dam surface. On the contrary, the loaded liquid remains in direct contact with the dam–liquid interface throughout the process. It is precisely this direct contact, together with the hydrophobicity introduced by the

tri-block probe, that stably confines the liquid within the sample reservoir and prevents its spontaneous spreading toward the channel. On this basis, the target RNA and activated Cas13a in the liquid phase do not need to "penetrate" a hydrophobic layer that is separated from the aqueous phase. Rather, they can naturally reach the dam–liquid interface, which is already in direct contact with the sample, through molecular diffusion, and then interact with the immobilized probes there. In other words, the macroscopic hydrophobicity determines the wetting state of the liquid at this interface and the difficulty of crossing the dam region. It does not determine whether molecules in the liquid phase are unable to approach that interface. Therefore, in our system, the hydrophobic dam should not be understood as a barrier that aqueous molecules cannot reach, but rather as an anti-wetting interface that the liquid can contact but does not readily cross unless the interfacial properties are altered. In addition, as shown in our response to Comment 1, we experimentally verified that the conventional FAM-6U-BHQ reporter and the FAM-$(PEG_6)_2$-6U-$(CH_2)_{12}$-BHQ reporter used in this study exhibited nearly identical fluorescence kinetic profiles in the CRISPR-Cas13a system. This result indicates that the hydrophobic group in the $(PEG_6)_2$-6U-$(CH_2)_{12}$H tri-block probe does not noticeably hinder Cas13a access to the probe.

Regarding the reviewer's further question of whether cleavage may occur only in a narrow interfacial region, such as the pinned contact line, we believe this is a highly insightful interpretation. In the original manuscript, during optimization of the dam width, we observed that when the dam was too wide, a continuous wetting transition sufficient to span the full dam width could not be formed within a limited time, and no "dam-break drainage" event was generated. This observation suggests that cleavage is more likely to occur first in the interfacial region that is initially accessible to the liquid, and then progressively propagate along the advancing wetting front, rather than requiring uniform cleavage across the entire dam region at the same moment. In other words, the operating mode of this platform is inherently a process in which the liquid-accessible interfacial region responds first, followed by local propagation that eventually triggers the overall "dam-break drainage" event. It is not a process in which the entire hydrophobic dam fails simultaneously and uniformly. More importantly, through the structural design of the threshold-confined droplet, we intentionally tuned the liquid volume and dam width to a critical state that is highly sensitive to local interfacial perturbation. As a result, even slight and localized cleavage on the dam surface can be sufficient to trigger the overall "dam-break drainage" event.

The corresponding modification has been made:

1.Page 3, lines 93-99; Page 4, lines 110-125

The immobilized tri-block probes render the overall dam surface hydrophobic, thereby preventing the liquid from flowing beyond the sample reservoir. We employed the CRISPR-Cas13a system as a target-activated mediator to degrade the responsive spacer and trigger a hydrophobic-to-hydrophilic dam wettability transition. Owing to the programmability of crRNA, Cas13a′s RNase activity can be specifically activated by the target nucleic acid of any pathogen and a single activated Cas13a is able to cleave thousands of single-stranded RNA (ssRNA) substrates[22-24] (Fig. 1cIII).

Accordingly, we designed the responsive linker as a ssRNA consisting of six uridine residues (6U), while the proximal hydrophilic group was formed by two PEG_6 chains and the distal hydrophobic group featured a $C_{12}H_{25}$ tail (Fig. 1cII). As shown in Fig. 1cI, upon target recognition, the CRISPR-Cas13a system mediates efficient degradation of the ssRNA spacer, resulting in a pronounced hydrophobic-to-hydrophilic wettability transition at dam surface. This transition allows the liquid to breach the dam and flow along the channel producing a "dam-break drainage" phenomenon. Conversely, in the absence of target nucleic acid, the Cas13a protein remains inactive, preserving the integrity of the tri-block probe and preventing liquid flow. By integrating the sensing mechanism that CRISPR-Cas13a-mediated dam wettability switching with a "dam-break drainage" sensing model, this work opens a new window for point-of-care diagnostic solution.

Was replaced by

The immobilized tri-block probes render the overall dam surface hydrophobic, thereby increasing the wetting barrier for forward spreading of the liquid and preventing spontaneous flow beyond the sample reservoir. Notably, hydrophobic dam does not imply that it is inaccessible to the aqueous phase, rather, the loaded sample remains in direct contact with the dam surface and forms a confined liquid–dam interface. We employed the CRISPR-Cas13a system as a target-activated mediator to degrade the responsive spacer at this interface and trigger a hydrophobic-to-hydrophilic wettability transition. Owing to the programmability of crRNA, Cas13a′s RNase activity can be specifically activated by the target nucleic acid of any pathogen and a single activated Cas13a is able to cleave thousands of single-stranded RNA (ssRNA) substrates[23-25] (Fig. 1cIII). Accordingly, we designed the responsive linker as a ssRNA consisting of six uridine residues (6U), while the proximal hydrophilic group was formed by two PEG_6 chains and the distal hydrophobic group featured a $C_{12}H_{25}$ tail (Fig. 1cII). To examine whether the central RNA-responsive segment in the tri-block probe might be less accessible to Cas13a due to the flanking hydrophobic module and possible steric hindrance, we performed parallel control experiment while keeping all other components of the CRISPR-Cas13a system unchanged. One group used the conventional FAM-6U-BHQ fluorescent reporter, whereas the another one used the FAM-$(PEG_6)_2$-6U-$(CH_2)_{12}$-BHQ fluorescent reporter. The two groups exhibited nearly identical fluorescence kinetic profiles, indicating that the $(PEG_6)_2$-6U-$(CH_2)_{12}H$ tri-block architecture itself does not impair Cas13a access to the central RNA-responsive segment (Fig. S1). As shown in Fig. 1cI, upon target recognition, CRISPR-Cas13a-mediated cleavage of the middle ssRNA responsive group disrupts the interfacial hydrophobicity of the molecular dam, allowing the liquid to breach the dam and flow along the channel, thereby producing a "dam-break drainage" event. Conversely, in the absence of target nucleic acid, the Cas13a protein remains inactive, preserving the integrity of the tri-block probe and maintaining liquid confinement.

2.Page 5, lines 210-219

This is attributed to the interface-dependent and layer-by-layer degradation of the tri-block probes mediated by activated Cas13a on the molecular dam. The tri-block probes that were directly contact

with the liquid are cleaved first, with subsequent degradation occurring progressively. At excessive dam widths, the activated Cas13a protein cannot degrade the tri-block probes across the entire dam.

Was replaced by

This behavior is most consistent with an interface-dependent, layer-by-layer degradation process of the tri-block probes mediated by activated Cas13a on the molecular dam. The probes directly contacting the liquid are expected to be cleaved first, followed by progressive degradation toward the inner region of the dam. These results suggest that the hydrophobicity of the dam primarily acts as a macroscopic wetting barrier that restricts spontaneous liquid spreading, rather than preventing aqueous molecules from accessing the dam surface. Accordingly, probe cleavage initiated at the liquid–dam interface rather than occurring uniformly across the entire hydrophobic barrier. When the dam width is excessive, the wettability transition cannot propagate across the full width of the dam within the allowed time, and the liquid therefore remains confined in region A despite the presence of target nucleic acid. Therefore, we determined 90 μm in width and 0.27 mm in height as the optimal configuration for the physical dam.

▣ Minor Comment

Comment 1. The blank (no-template control) amplification curve is missing in Supplementary Fig. 6 (RT-PCR curves).

Response: We thank the reviewer for this careful comment. After rechecking Supplementary Fig. 6, we found that the NTC curve was difficult to distinguish in the original figure because it nearly overlapped with the 10 aM curve. In the revised Supplementary Fig. 6, we have explicitly labeled the NTC curve and changed its color to black to improve figure clarity. We appreciate the reviewer's careful observation, which helped us improve the clarity and rigor of the data presentation.

The corresponding modification has been made:

Page 9, SI

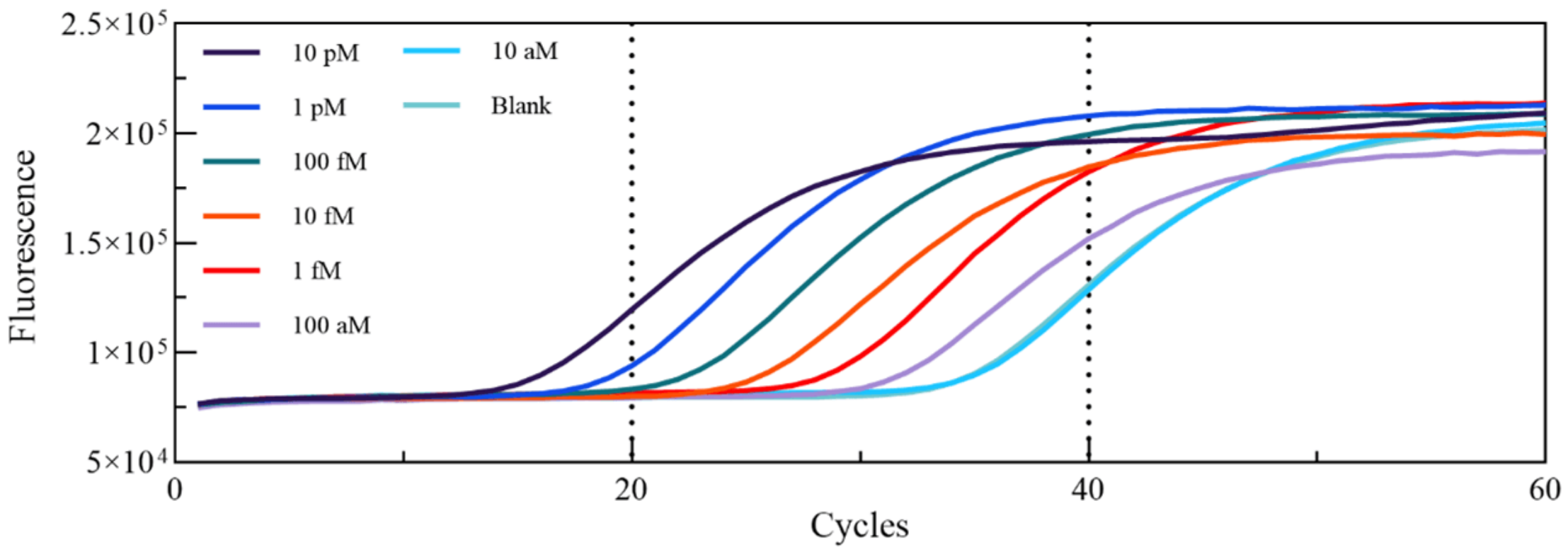

Was replaced by

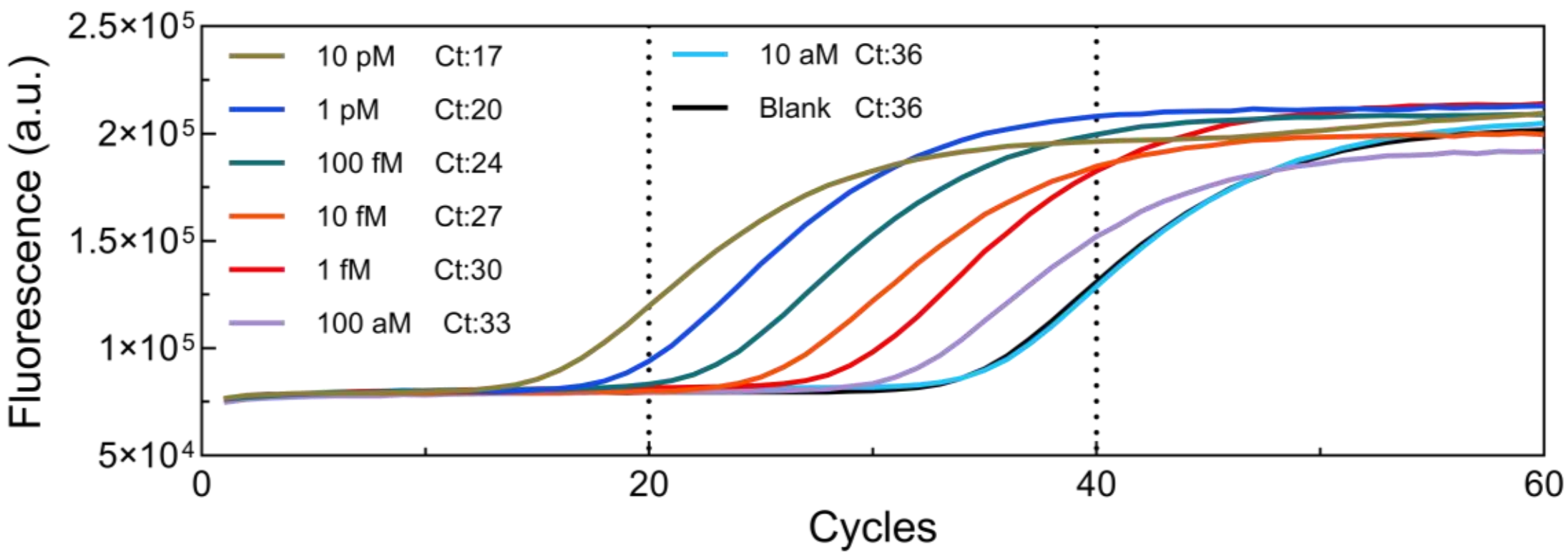

Comment 2. Consistency in Ct value annotation across supplementary RT-PCR figures. In several supplementary figures showing RT-PCR amplification curves (e.g., Supplementary Fig. 6 and related panels), Ct values are not explicitly annotated on the curves. For clarity and consistency, the authors are encouraged to label Ct values directly on the amplification plots, similar to the presentation style used in Supplementary Fig. 9.

Response: We sincerely thank the reviewer for this helpful suggestion. As recommended, we have revised all relevant supplementary RT-PCR amplification plots by directly annotating the corresponding Ct values on the curves, using the same presentation style as in Supplementary Fig. 9 to ensure consistency and clarity across the figures.

The corresponding modification has been made:

1.Page 9; SI

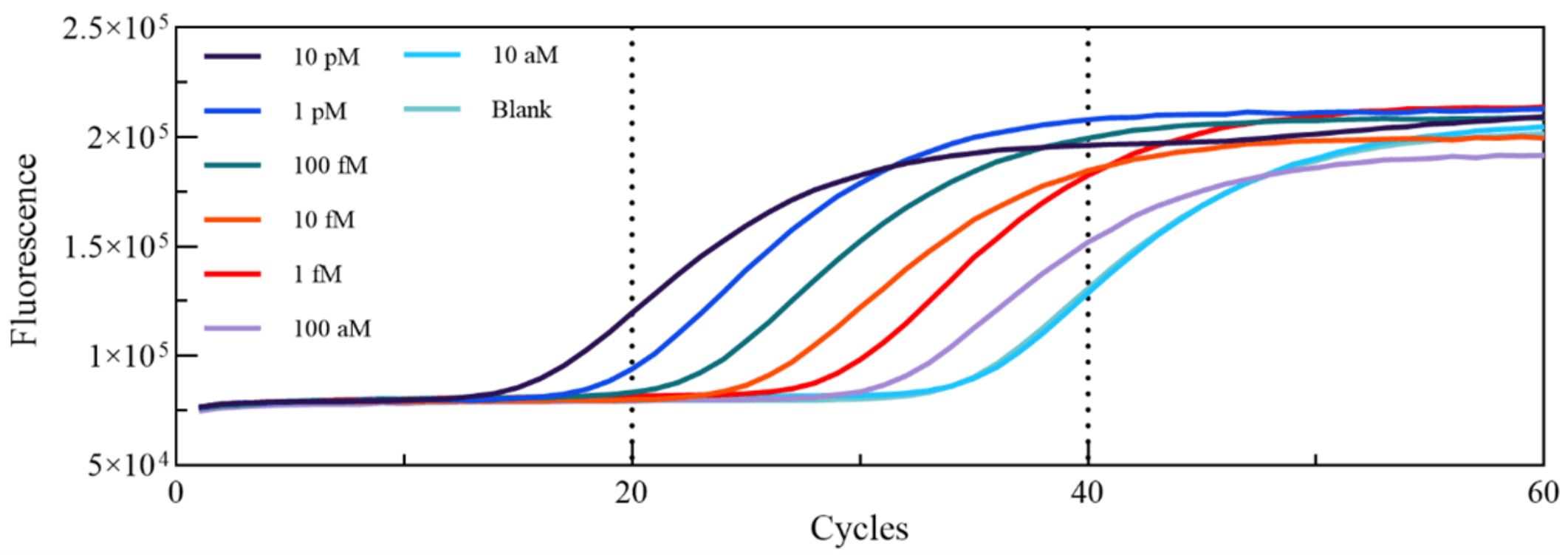

Was replaced by

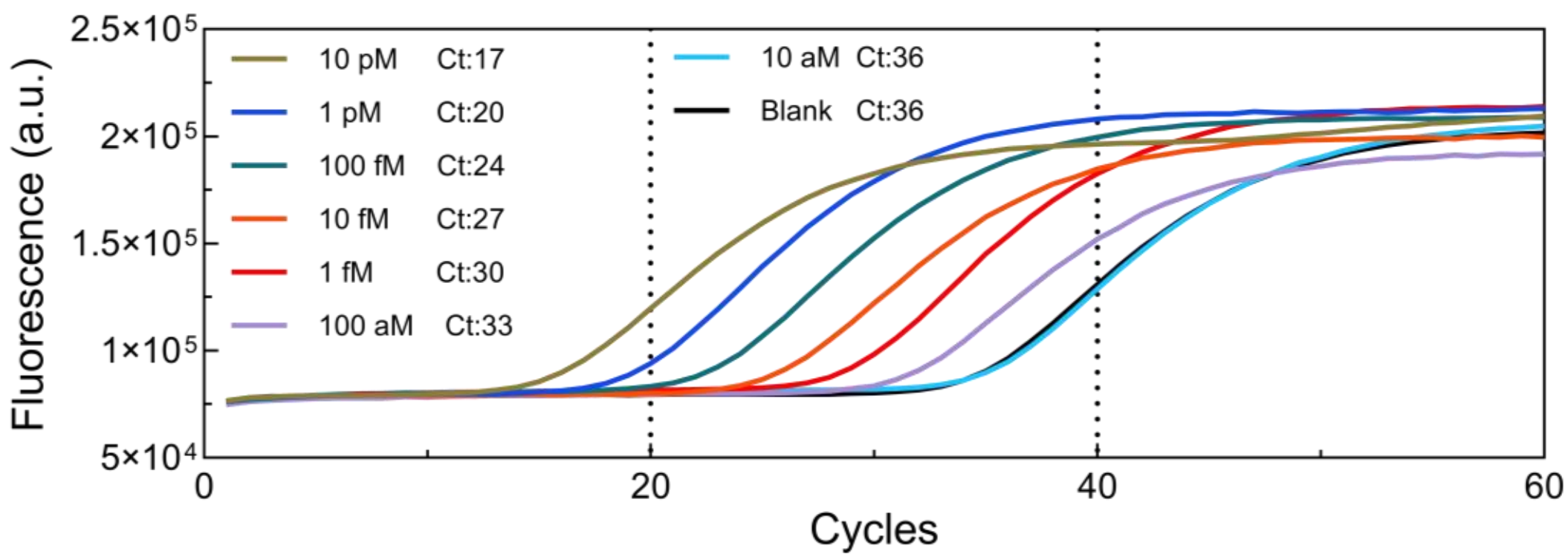

2.Page 20; SI

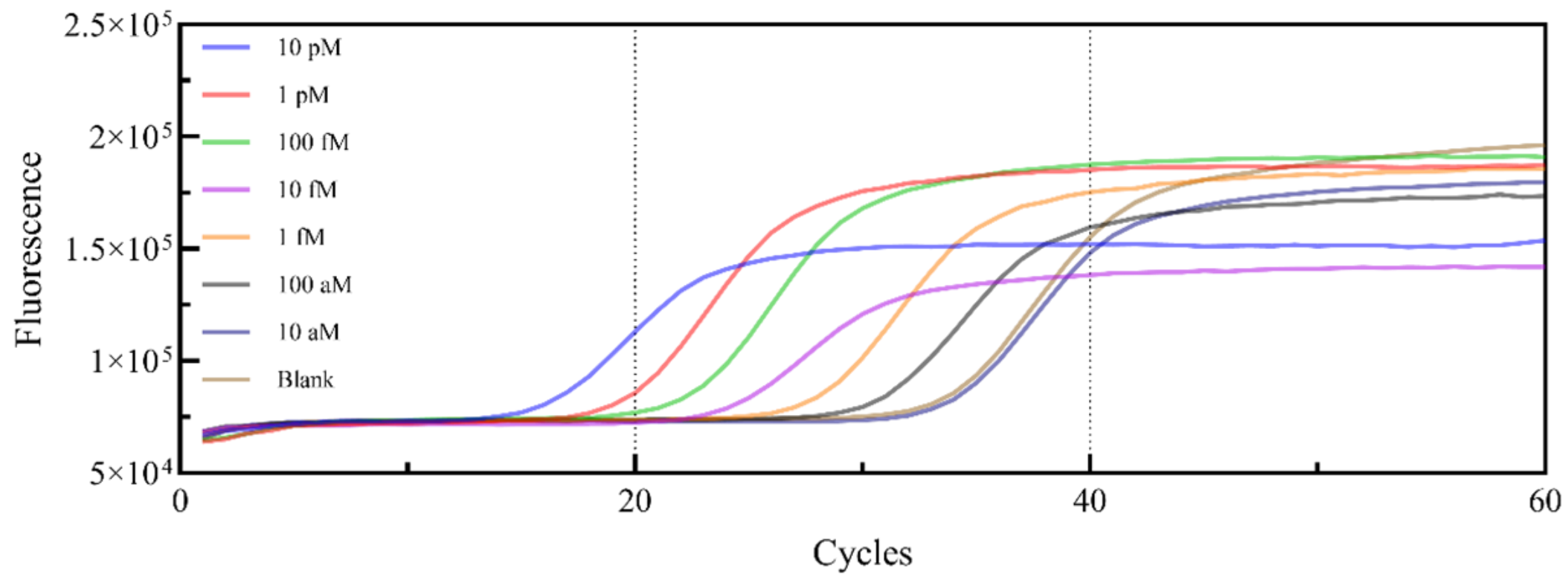

Was replaced by

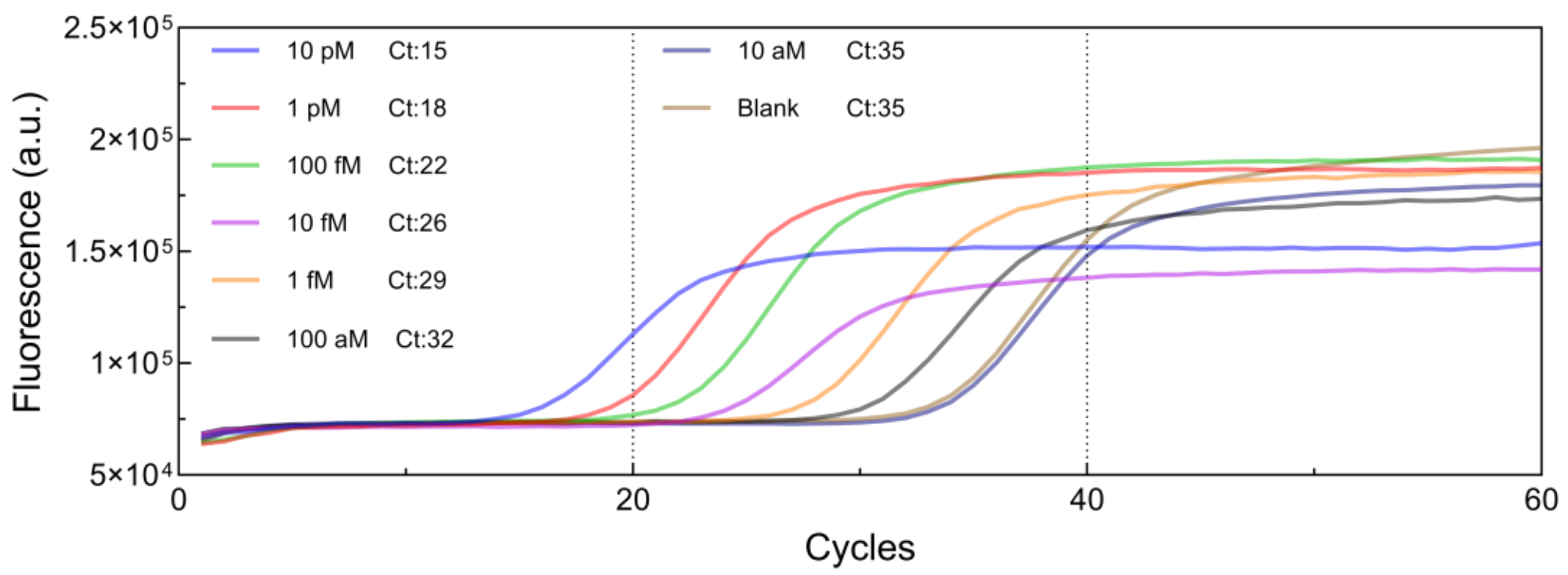

3.Page 21; SI

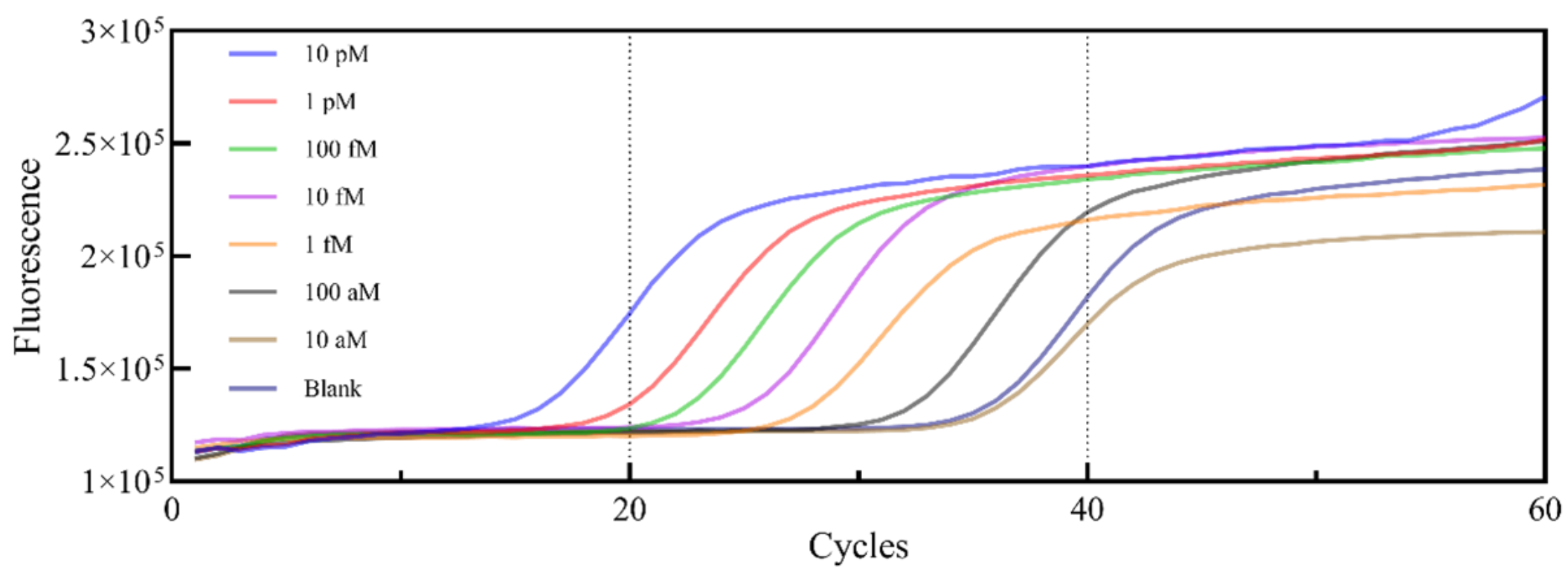

Was replaced by

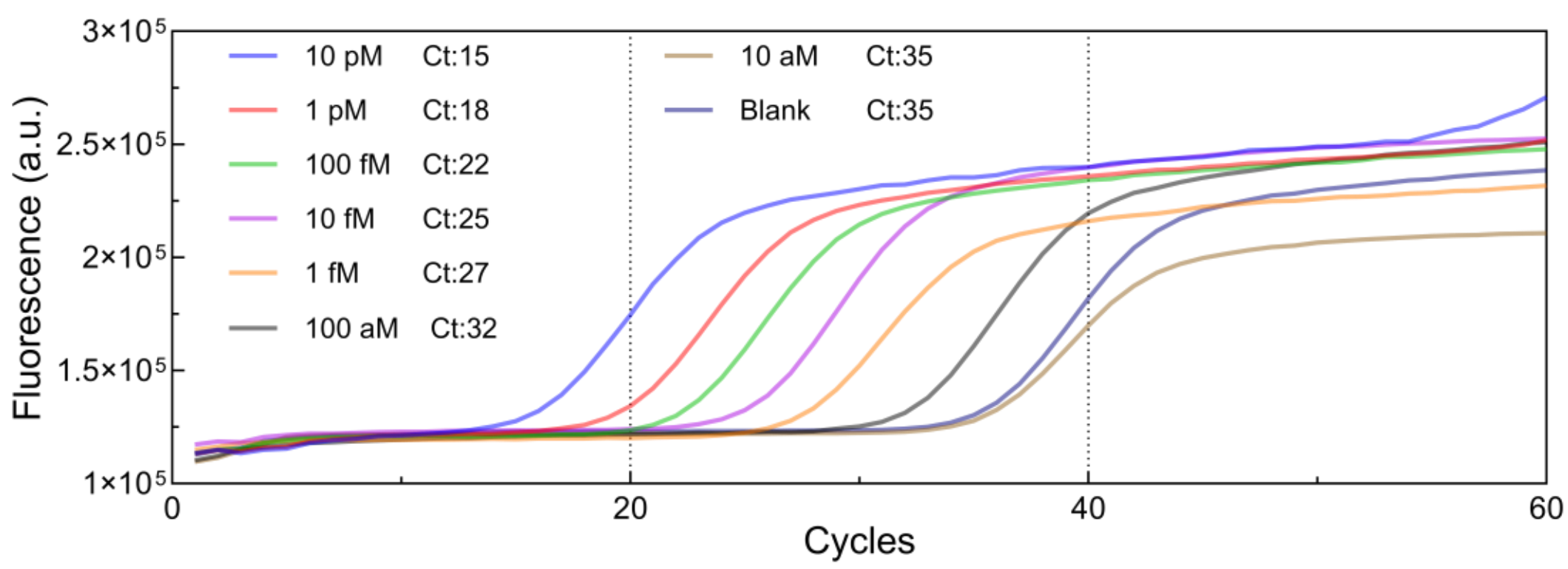

Comment 3. In Supplementary Fig. 12 and other figures presenting statistical plots, it is unclear what individual data points represent (e.g., independent experiments, technical replicates, or averaged values). The authors should explicitly state the nature of the data points in the figure captions or methods.

Response: We sincerely thank the reviewer for pointing out this important issue. In response to this helpful suggestion, we have revised the legends of Supplementary Figs. 5, 12, 15 and 20a to clarify the meanings of each data point and the corresponding error bars. We also appreciate this careful and valuable comment, which has helped us further improve the rigor and clarity of data presentation in the manuscript.

The corresponding modification has been made:

1.Page 8, lines 2-8; SI

Supplementary Fig. 5. Statistical analysis of detection times for the single-plex chip in detection of SARS-CoV-2 N gene across concentrations from 10 aM to 10 pM. All experiments were performed in three technical replicates (n=3), confirming reliable completion of detection within 2 minutes for all tested concentrations.

Was replaced by

Supplementary Fig. 6. Statistical analysis of readout times for the single-plex chip in detection of the SARS-CoV-2 N gene across concentrations from 10 aM to 10 pM. All experiments were performed in three independent measurements (n=3), showing that all tested positive concentrations triggered visible "dam-break drainage" event within 2 min rapid decision time, whereas blank samples remained confined throughout the 10 min observation window. Each orange data plot represents the mean detection time at the indicated concentration, and error bars indicate the range of detection times observed across three independent experiments.

2.Page 16, lines 2-10; SI

Supplementary Fig. 12. (a) The single-plex chip exhibited a detection time of less than 2 minutes for clinical samples with RT-PCR Ct values from 28 to 33. All experiments were performed in three independent measurements (n=3), confirming the robustness of its rapid detection performance (Note: Samples 1-10 represents the order of positive samples, Sample 11 represents the negative sample).

Was replaced by

Supplementary Fig. 14. (a) Detection-time analysis of the single-plex chip for clinical nasal-swab samples with RT-PCR Ct values from 28 to 33. All experiments were performed in three independent measurements (n=3), showing that all positive samples triggered visible "dam-break drainage" event within 2 min rapid decision time, whereas the negative sample remained confined in the sample reservoir throughout the 10 min observation window. Each black dot indicates the RT-PCR Ct value of an individual sample, whereas each orange dot indicates the mean detection time of the corresponding sample on the chip; green error bars denote the range of detection times observed across three independent experiments. (Note: Samples 1–10 denote the order of positive samples, and Sample 11 denotes the 10 in 1 negative sample.)

3.Page 19, lines 2-9; SI

Supplementary Fig. 15. Statics of detection time for duplex chip in simultaneously analyzing the IAV-PB2 and IBV-PB1 genes. Detection time of the duplex chip for concurrent analysis of the IAV-PB2 and IBV-PB1 genes over concentrations ranging from 100 aM to 10 pM, with both targets detected within 5 minutes. All measurements were conducted in triplicate (n=3).

Was replaced by

Supplementary Fig. 17. Statics of detection time for duplex chip in simultaneously analyzing the IAV-PB2 and IBV-PB1 genes. Detection time of the duplex chip for concurrent analysis of the IAV-PB2 and IBV-PB1 genes over concentrations ranging from 100 aM to 10 pM. In three independent measurements (n=3), visible "dam-break drainage" event was consistently observed for all tested positive concentrations within 5 min rapid decision time, while blank samples remained confined throughout the 10 min observation window. Each orange data plot indicates the mean detection time at the indicated concentration on the chip, and error bars denote the range of detection times observed across three independent experiments.

4.Page 25, lines 2-10; SI

Supplementary Fig. 20. (**a**) Statistical analysis of detection times for the duplex chip in simultaneous detection of IAV and IBV clinical samples. All experiments were performed in three independent measurements (n=3), confirming the robustness of its rapid detection performance (Note: Samples 1-10 represents ten groups of randomly mixed IAV RT-PCR-positive (Ct=28-33) and IBV RT-PCR-positive samples (Ct=28-33), Sample 11 represents the negative sample).

Was replaced by

Supplementary Fig. 23. (**a**) Statistical analysis of detection times for the duplex chip in simultaneous detection of IAV and IBV clinical samples. In three independent measurements (n=3), all positive samples reproducibly triggered visible "dam-break drainage" event within 5 min rapid decision time, whereas the negative sample remained confined in the sample reservoir throughout the 10 min observation window. Each blue data plot indicates the mean detection time of the corresponding sample on the chip, and error bars denote the range of detection times observed across three independent experiments. (Note: Samples 1-10 represents ten groups of randomly mixed IAV RT-PCR-positive (Ct=28-33) and IBV RT-PCR-positive samples (Ct=28-33), Sample 11 represents the 10 in 1 negative sample).

Was added

Comment 4. For all figures that include statistical analysis, the authors should specify how error bars are. This information should be consistently reported either in the figure captions or in the Methods section.

Response: We sincerely thank the reviewer for this valuable suggestion regarding the completeness of statistical information. In the revised manuscript, we have now uniformly clarified the definitions of error bars in all relevant figures and provided explicit descriptions in the corresponding figure captions to ensure the completeness and consistency of statistical reporting. These changes have been incorporated into the Figure captions of the revised manuscript. We again appreciate the

reviewer's careful and constructive comment, which has significantly improved the rigor and clarity of data presentation in the manuscript.

The corresponding modification has been made:

1.Page 5, lines 2-6; SI

Supplementary Fig. 2. Optimization of chip surface modification conditions. (a) Summary table of the chip surface-modification parameters, including the reagents, reaction time, and corresponding working concentrations. **(b)**Success rate of the sample reservoir successfully confined 600 μL water as a function of the probe addition concentration during the modification procedure. Note: Success rate refers to the proportion of chips whose A region successfully confined 600 μL of water among 12 chips.

Was replaced by

Supplementary Fig. 3. Optimization of chip surface modification conditions. (a) Summary table of the chip surface-modification parameters, including the reagents, reaction time and corresponding working concentrations. **(b)**Success rate of stable confinement of 600 μL water in the sample reservoir as a function of probe concentration during the surface modification procedure. Error bars indicate the variation range of the success rate across 12 independent chips.

2.Page 8, lines 2-8; SI

Supplementary Fig. 5. Statistical analysis of detection times for the single-plex chip in detection of SARS-CoV-2 N gene across concentrations from 10 aM to 10 pM. All experiments were performed in three technical replicates (n=3), confirming reliable completion of detection within 2 minutes for all tested concentrations.

Was replaced by

Supplementary Fig. 6. Statistical analysis of readout times for the single-plex chip in detection of the SARS-CoV-2 N gene across concentrations from 10 aM to 10 pM. All experiments were performed in three independent measurements (n=3), showing that all tested positive concentrations triggered visible "dam-break drainage" event within 2 min rapid decision time, whereas blank samples remained confined throughout the 10 min observation window. Each orange data plot represents the mean detection time at the indicated concentration, and error bars indicate the range of detection times observed across three independent experiments.

3.Page 16, lines 2-10; SI

Supplementary Fig. 12. (a) The single-plex chip exhibited a detection time of less than 2 minutes for clinical samples with RT-PCR Ct values from 28 to 33. All experiments were performed in three independent measurements (n=3), confirming the robustness of its rapid detection performance (Note: Samples 1-10 represents the order of positive samples, Sample 11 represents the negative sample).

Was replaced by

Supplementary Fig. 14. (a) Detection-time analysis of the single-plex chip for clinical nasal-swab samples with RT-PCR Ct values from 28 to 33. All experiments were performed in three independent measurements (n=3), showing that all positive samples triggered visible "dam-break drainage" event within 2 min rapid decision time, whereas the negative sample remained confined in the sample reservoir throughout the 10 min observation window.

Each black dot indicates the RT-PCR Ct value of an individual sample, whereas each orange dot indicates the mean detection time of the corresponding sample on the chip; green error bars denote the range of detection times observed across three independent experiments. (Note: Samples 1–10 denote the order of positive samples, and Sample 11 denotes the 10 in 1 negative sample.)

4.Page 19, lines 2-9; SI

Supplementary Fig. 15. Statics of detection time for duplex chip in simultaneously analyzing the IAV-PB2 and IBV-PB1 genes. Detection time of the duplex chip for concurrent analysis of the IAV-PB2 and IBV-PB1 genes over concentrations ranging from 100 aM to 10 pM, with both targets detected within 5 minutes. All measurements were conducted in triplicate (n=3).

Was replaced by

Supplementary Fig. 17. Statics of detection time for duplex chip in simultaneously analyzing the IAV-PB2 and IBV-PB1 genes. Detection time of the duplex chip for concurrent analysis of the IAV-PB2 and IBV-PB1 genes over concentrations ranging from 100 aM to 10 pM. In three independent measurements (n=3), visible "dam-break drainage" event was consistently observed for all tested positive concentrations within 5 min rapid decision time, while blank samples remained confined throughout the 10 min observation window. Each orange data plot indicates the mean detection time at the indicated concentration on the chip, and error bars denote the range of detection times observed across three independent experiments.

5.Page 25, lines 2-10 SI

Supplementary Fig. 20. (**a**) Statistical analysis of detection times for the duplex chip in simultaneous detection of IAV and IBV clinical samples. All experiments were performed in three independent measurements (n=3), confirming the robustness of its rapid detection performance (Note: Samples 1-10 represents ten groups of randomly mixed IAV RT-PCR-positive (Ct=28-33) and IBV RT-PCR-positive samples (Ct=28-33), Sample 11 represents the negative sample).

Was replaced by

Supplementary Fig. 23. (**a**) Statistical analysis of detection times for the duplex chip in simultaneous detection of IAV and IBV clinical samples. In three independent measurements (n=3), all positive samples reproducibly triggered visible "dam-break drainage" event within 5 min rapid decision time, whereas the negative sample remained confined in the sample reservoir throughout the 10 min observation window. Each blue data plot indicates the mean detection time of the corresponding sample on the chip, and error bars denote the range of detection times observed across three independent experiments. (Note: Samples 1-10 represents ten groups of randomly mixed IAV RT-PCR-positive (Ct=28-33) and IBV RT-PCR-positive samples (Ct=28-33), Sample 11 represents the 10 in 1 negative sample).

Comment 5. Fig.3f and Fig.6f, the "Fold Changes" should be explained in the manuscript.

Response: We sincerely thank the reviewer for this important suggestion. In response, we have clarified the definition of Fold Change in the revised manuscript. In this study, the RT-PCR results for the SARS-CoV-2 N gene shown in Fig. S5 indicate that a tenfold difference in target

concentration corresponds to an approximately three-cycle difference in Ct value. Therefore, we normalized the data using the positive sample with Ct=34 as the reference and defined its relative target abundance as 1. Based on this relationship, the relative target abundance in the sample with Ct=31 is approximately 10-fold higher than that of the reference sample, whereas that in the sample with Ct=28 is approximately 100-fold higher. This explanation has been added to the figure captions of Fig. 3f and Fig. 6f. We again thank the reviewer for this constructive suggestion, which has helped improve the clarity and accuracy of data interpretation in the manuscript.

The corresponding modification has been made:

1.Page 9, lines 221-230

Figure 3 | Performance evaluation of the single-plex chip for SARS-CoV-2 detection. (**a**) Workflow schematic for SARS-CoV-2 detection. (**b**) Detection of serial dilutions of the SARS-CoV-2 N gene ranging from 1 aM to 10 pM. (**c**) Analysis of clinical nasal swab samples with Ct values between 28 and 34. (**d**) 10 in 1 pooled testing: negative pools were confined, whereas pools containing a single positive sample produced a distinct "dam-break drainage" signal within 2 min. (**e**) Specificity assessment using Influenza A virus, Influenza B virus and respiratory syncytial virus (RSV)samples; only SARS-CoV-2 triggered the "dam-break drainage" signal. (**f**) Comparative evaluation of detection performance among the single-plex chip, RT-PCR and CRISPR-Cas13a. (**g**) Confusion matrix relative to clinical RT-PCR results, indicating 100% sensitivity, specificity and accuracy. (**h**) Benchmarking of the developed chip against previously reported detection technologies.

Was replaced by

Figure 3 | Performance evaluation of the single-plex chip for SARS-CoV-2 detection. (a) Workflow schematic for SARS-CoV-2 detection. **(b)** Detection of serial dilutions of the SARS-CoV-2 N gene ranging from 1 aM to 10 pM. **(c)** Analysis of clinical nasal swab samples with Ct values between 28 and 34. **(d)** 10 in 1 pooled testing: negative pools were confined, whereas pools containing a single positive sample produced a distinct "dam-break drainage" signal within 2 min. **(e)** Specificity assessment using Influenza A virus, Influenza B virus and respiratory syncytial virus (RSV) samples; only SARS-CoV-2 triggered the "dam-break drainage" event. **(f)** Comparative evaluation of detection performance among the single-plex chip, RT-PCR and CRISPR-Cas13a. Fold changes denote the normalized relative abundance of SARS-CoV-2 target RNA estimated from RT-PCR Ct values, using the positive sample with Ct=34 as the reference (set to 1). **(g)** Confusion matrix relative to clinical RT-PCR results, indicating 100% sensitivity, specificity and accuracy. **(h)** Benchmarking of the developed chip against previously reported detection technologies.

2.Page 14, lines 362-375

Figure 6 | Duplex chip evaluation for Influenza A and B virus detection. (**a**) Schematic illustration of the workflow for duplex detection of IAV and IBV on the duplex chip. (**b**) Representative detection outcomes for negative, IAV-positive, IBV-positive and dual-positive samples. The "dam-break drainage" signal appears exclusively at the corresponding channel within 5 min, indicating high target specificity. (**c**) Analytical sensitivity assessment using serial dilutions (10 aM–10 pM) of synthetic IAV-PB2 and IBV-PB1 RNA targets at equal concentrations. (**d**) Clinical validation with nasal swab samples: pooled analysis of ten IAV-positive (Ct=28-33) and

ten IBV-positive (Ct=28-33) clinical specimens showed accurate target identification without cross-reactivity. (**e**) 10 in 1 pooled testing: the negative pool produced no drainage signal, whereas pools containing a single IAV-positive or IBV-positive sample generated distinct signals on their respective sides within 5 min. Dual-positive pools triggered synchronous drainage signals on both channels. (**f**) Comparative analysis of detection performance across the duplex chip, RT-PCR and CRISPR-Cas13a assays, confirming consistent diagnostic accuracy and reliability.

Was replaced by

Figure 6 | Duplex chip evaluation for Influenza A and B virus detection. (**a**) Schematic illustration of the workflow for duplex detection of IAV and IBV on the duplex chip. (**b**) Representative detection outcomes for negative, IAV-positive, IBV-positive and dual-positive samples. The "dam-break drainage" event appears exclusively at the corresponding channel within 5 min. (**c**) Sensitivity assay for IAV-PB2 and IBV-PB1 RNA pooled samples with concentration ranging from 10 aM–10 pM in 5 min rapid decision time. (**d**) Clinical validation with nasal swab samples: pooled analysis of ten IAV-positive (Ct=28-33) and ten IBV-positive (Ct=28-33) clinical specimens showed accurate target identification without cross-reactivity. (**e**) 10 in 1 pooled testing: the negative pool produced no "dam-break drainage" event, whereas pools containing a single IAV-positive or IBV-positive sample generated clear "dam-break drainage" readout on their corresponding channel within 5 min rapid decision time. Dual-positive pools present "dam-break drainage" events on both channels. (**f**) Comparative analysis of detection performance across the duplex chip, RT-PCR and CRISPR-Cas13a assays in sensitivity and detection time. Fold changes denote the normalized relative abundance of the corresponding target estimated from RT-PCR Ct values, using the positive sample with Ct=34 as the reference (set to 1) for each target. Note: For the duplex assay, blank and negative controls showed stable liquid confinement over 10 min observation period, whereas all target-positive samples, across the tested concentration range, produced a "dam-break drainage" event within 5 min. Accordingly, 5 min was defined as the result rapid decision time.

Thank you for your nice comments and your recognition of our work again! Your professional and careful suggestions significantly improved our manuscript. Wish you a happy life and successful career!

Reviewer 2

▌Major Comment

Comment 1. The core approach uses Cas13a collateral cleavage to switch surface wettability and produce a naked-eye liquid-motion readout. This is conceptually very similar to a recent Science Advances single-tube liquid-motion assay (DOI: 10.1126/sciadv.adu2271), which also converts Cas13a-triggered wettability changes into instrument-free fluid movement. Beyond a different readout format, key performance metrics (time, cost, and LoD) appear broadly comparable. Thus, while this work improves chip design and enables dual-target detection, the advance is mainly in device/workflow optimization rather than a clear mechanistic breakthrough, and the incremental conceptual contribution is limited.

Response: We sincerely thank you for this exceptionally careful, insightful and thought-provoking evaluation of our manuscript. You not only accurately recognized the internal connection between the present work and our team's earlier study published in Science Advances, but also perceptively pointed out that, unless the boundary of innovation between the two is clearly defined, readers may find it difficult to distinguish a continuation of the original principle from an independent scientific advance. As the researchers who carried out both studies, we are fully aware of both the value and the limits of the earlier work. The previous study established the feasibility of the concept, whereas the present work begins precisely where that study stopped and advances it from proof of concept to method establishment. The reviewer's comment helped us realize more deeply that, precisely because this work continues our own research line, we had an even greater responsibility to explain clearly why this study represents an independent and necessary methodological advance. We feel fortunate to have received comments from a reviewer who is truly familiar with progress in this field and able to distinguish so clearly between continuation of principle and independent advance. We fully accept this criticism. We now clearly recognize that the most fundamental problem of the original manuscript was not what we had done, but that we did not present clearly and accurately why this work constitutes an independent and necessary advance. In response, we have fundamentally restructured the narrative framework of the revised manuscript.

When preparing the original submission, our writing was strongly shaped by urgent public health concerns. Faced with recurrent outbreak threats, the long-standing lack of testing capacity in resource-limited settings and the real cost borne by large populations during delayed early screening, our strongest intention was to convey the translational potential of this technology. We therefore placed strong emphasis on its low cost, instrument-free operation, speed, high sensitivity and its potential to move beyond the laboratory and serve communities and households. In other words, we tried first to show readers what this technology can do.

We now clearly recognize that, although this writing framework was sincerely motivated by real-world concerns, it did not align well with the central evaluation criteria of Nature Communications, which place particular weight on conceptual advance and mechanistic

contribution. We focused too heavily on application value, but did not clearly define the methodological position of the present work. This was the central weakness in the design of our narrative framework and we have now comprehensively corrected it in the revised manuscript.

We would also like to provide one point of background that may help your evaluation. The Science Advances paper (DOI: 10.1126/sciadv.adu2271) is our own previous work. I was the first author of that study and am the corresponding author of the present manuscript.

The central idea of the earlier study was that the conventional route of microscopic molecular recognition → optical/electrical excitation → signal readout is the fundamental reason why traditional detection methods face a trade-off between high sensitivity, instrument-free deployment and low cost. On this basis, in the Science Advances work, we first proposed a new direct signal-transduction route in which target molecules, through CRISPR-mediated interfacial wettability switching, allow microscopic molecular recognition to be converted directly into a macroscopic visible signal. We then implemented this direct transduction route in a tube-based sensor, in which the readout was obtained simply by observing whether the liquid in the tube would fall. In essence, the Science Advances study was a proof-of-concept demonstration of this direct signal-transduction route using a tube platform.

However, although the tube platform proved the validity of this concept, it also had two core limitations at the principle level. These limitations were embedded in its basic architecture and could not be overcome by simple parameter adjustment or workflow optimization. As a result, the concept remained at the stage of laboratory demonstration and could not readily develop into a standardized and clinically deployable methodology.

First, in the original tube-inversion readout, signal triggering was tightly coupled to the angle, speed and consistency of manual operation. This introduced strong operator dependence and could not meet the robustness required for standardized deployment.

Second, the original system could only support single-target readout. It did not establish any solution for parallel and distinguishable multi-target detection within a shared reaction system and therefore could not address the practical need for differential diagnosis of co-infections.

The independent methodological innovation of the present work lies in the development of a new platform for this concept, based on a chip architecture and a “dam-break drainage” event-readout mode. In this way, the direct signal-transduction route based on CRISPR-mediated interfacial wettability switching is advanced from proof of concept toward method establishment.

First, we fundamentally restructured the signal-triggering logic. Through a chip geometry consisting of a sample reservoir, hydrophobic dam, flow channel and collection reservoir, we changed the triggering mechanism from one dependent on manual operation to one determined solely by boundary conditions and interfacial state. Once the sample is loaded, no further manual action is required. Whether signal generation occurs, and when it occurs, are fully determined by the interfacial wettability change and the geometric parameters of the chip. This removes the error and uncertainty introduced by manual handling. More importantly, this is a fundamental shift from a demonstration-level system to an engineering-ready and standardizable system, rather than a

simple change in readout format.

Second, we established a methodological framework for parallel target detection within a shared reaction system. We successfully achieved independent and visually distinguishable detection of two pathogen targets on the same chip and within the same reaction system, without cross-interference. To make this possible, we developed two new core technical elements. The first is a directional self-supplementing structure based on differences in surface energy. This solves the key problem that, after "dam-break drainage" event triggered by the first target, the reduced liquid volume would otherwise disrupt the detection threshold of the second target and lead to false-negative results. The second is a dam-anchored yet self-releasable crRNA molecular design. This enables target-specific local activation within a shared Cas13a reaction system and extends the effective action range of target-activated Cas13a. Together, these two advances establish a new signal-organizing strategy for multi-target detection in a shared system.

Therefore, the present manuscript represents a different methodological state that did not exist in the previous work, namely an operating mechanism based on steady-state triggering and organized parallel readout within a shared system. In our view, this is an independent development at the methodological level.

We would also like to state, cautiously, that for a new signal-transduction route, the first successful proof of principle is often only the starting point, rather than the endpoint of method establishment. Subsequent advances that address operational determinacy, structural stability, signal-readout organization and compatibility with multi-target detection may also constitute independent and substantial scientific contributions. A useful analogy may be found in the development of PCR. From the original conceptual introduction to later advances such as TaqMan probes, multiplex PCR and digital PCR, each step remained rooted in the same core principle. Yet each breakthrough that resolved a key methodological bottleneck has been widely recognized as a substantial scientific advance in its own right. We believe the present work represents this kind of progression. It moves a promising proof of concept toward a nucleic acid detection methodology with steady-state triggering and parallel readout in a shared system, bringing it closer to real deployment conditions.

In the revised manuscript, we have thoroughly restructured the Abstract and the Introduction. The central narrative has been shifted from an application-first emphasis to a methodology-first emphasis. At the very beginning of the manuscript, we now clearly define the key conceptual advance and mechanistic contribution of the present work relative to the previous study and to existing reports. We also clarify the essential step from proof of concept to method establishment, so that readers can recognize the level of originality of this work from the outset. In this way, we have fully corrected the narrative weakness of the original submission.

We respectfully ask whether you would be willing to reconsider the independent contribution and scientific value of this work in light of the substantially restructured revised manuscript. We are sincerely grateful again for your time, thoughtful consideration and valuable insight.

The corresponding modification has been made:

1.Page 1, lines 11-22

The critical need for highly sensitive, rapid, low-cost and equipment-free pathogens diagnostics is acutely felt in resource-limited settings, such as in low-income region and at home, where existing methods force a compromise among these key metrics. We break this trade-off with a nucleic acid chip that leverages a novel This solves the key problem that, after "dam-break drainage" event triggered by the first target, the reduced liquid volume would otherwise disrupt the detection threshold of the second target and lead to false-negative results readout model and a CRISPR-Cas13a-mediated interfacial wettability switching sensing mechanism. This design unlocks both single-plex and duplex visual detection, delivering attomolar sensitivity (10 and 100 aM), rapid results (2 and 5 min) and low cost (0.20$ and 0.30$ per test), respectively. In a 40 clinical-samples validation for SARS-CoV-2, Influenza A virus and Influenza B virus, our platform demonstrated robust clinical concordance and successfully detected samples with ambiguous RT-PCR results (Ct=35). This work provides a powerful and accessible solution for next-generation point-of-care molecular testing.

Was replaced by

Here, we reported a stable "dam-break drainage" nucleic acid chip that supports both single-plex and duplex visual detection under fixed-boundary conditions after one-step sample loading, without requiring any subsequent instrument assistance or manual actuation. The chip achieves single-plex detection with a cost 0.2 $, a detection limit of 10 aM and a rapid decision time 2 min and duplex detection with a cost 0.3 $, a detection limit of 100 aM and a rapid decision time 5 min. We further developed a practical nasal swab workflow based on room-temperature lysis and RNase inhibition, under which the platform showed full concordance with RT-PCR in 40 clinical nasal-swab samples for SARS-CoV-2, Influenza A virus and Influenza B virus. By enabling both steady-state single-target detection and duplex parallel readout in a shared system, this work advances CRISPR-Cas-mediated wettability-switching detection from conceptual demonstration toward methodological establishment and provides a new window for organized visual biosensing.

2.Page 2, lines 25-84

Infectious pathogens have persisted throughout human history, continuously posing serious threats to public health and social stability[1, 2]. Some pathogens display periodic outbreaks—such as Influenza A (IAV) and B (IBV) viruses—that cause seasonal epidemics and significant healthcare burdens[3]. Meanwhile, newly emerging and highly pathogenic agents have repeatedly triggered sudden global health crises, including severe acute respiratory syndrome (SARS), Middle East respiratory syndrome (MERS), Ebola virus disease, coronavirus disease 2019 (COVID-19) and more recently, chikungunya virus (CHIKV) outbreaks[4-9]. These events highlight that the threat of infectious diseases is not incidental but an enduring challenge. Consequently, the development of affordable, rapid, accurate and easily deployable diagnostic technologies is seriously needed for infectious disease prevention and control-particularly in resource-limited and low-income regions

where such capabilities hold irreplaceable strategic value.

The COVID-19 pandemic has highlighted a persistent gap in diagnostic capabilities—lacking technologies that can simultaneously perform highly sensitivity, low cost, rapid turnaround and operational simplicity without reliance on specialized equipment[10]. Lateral flow immunoassays (LFAs)[11] offer simple, low-cost, rapid and instrument-free operation, yet their limited sensitivity restricts their use for early-stage pathogen screening. In contrast, nucleic acid amplification techniques represented by reverse transcription-polymerase chain reaction (RT-PCR) provide high sensitivity and flexible probe redesign for emerging variants[12], but require sophisticated temperature-control instruments, trained personnel and long assay times, making them unsuitable for low-cost, field-deployable and rapid testing. Alternative approaches based on SHERLOCK[13], DETECTR[14, 15], electrochemical[16, 17], colorimetric[18, 19] and optical signal transduction[20, 21] have further diversified the detection landscape, yet most still depend on nucleic acid pre-amplification or external readout instruments. Consequently, achieving high sensitivity, family-affordability, operational simplicity and rapidity within a single diagnostic platform remains a formidable challenge.

Here, we reported a breakthrough sensing paradigm—converting target recognition into a visible "dam-break drainage" event readout through a programmable interfacial wettability switch mediated by CRISPR-Cas13a system. Based on this paradigm, we developed a diagnostic chip architecture comprising a sample reservoir, a functional dam, a microfluidic channel and a collection zone. In the absence of the target, liquid is confined in the sample reservoir by the dam structure; upon target recognition, the solution breaches the dam and flows into the channel, enabling unambiguous signal interpretation. This architecture is further engineered as a single-plex chip for individual pathogen detection and a duplex chip for simultaneous identification of two distinct targets. Using SARS-CoV-2 as a model, the single-plex chip achieved a detection limit of 10 aM within 2 min, at a cost of less than $0.20 per test. The duplex chip, demonstrated by simultaneous detection of Influenza A and B viruses, attained sensitivity of 100 aM for both targets within 5 min, costing under $0.30 per test. Clinical validation using 40 nasal swab samples (including SARS-CoV-2, IAV and IBV) showed 100% concordance with RT-PCR results. Notably, the platform reliably detected positives even at high cycle thresholds (Ct=35), where conventional assays often fail. These results collectively highlight the developed chips as a highly sensitive, rapid, low-cost and equipment-free platform for pathogen screening, particularly suited for low-resource settings.

Was replaced by

Infectious pathogens have persisted throughout human history, continuously posing serious threats to public health and social stability[1, 2]. Some pathogens display periodic outbreaks—such as Influenza A (IAV) and B (IBV) viruses—that cause seasonal epidemics and significant healthcare burdens[3]. Meanwhile, newly emerging and highly pathogenic agents have repeatedly triggered sudden global health crises, including severe acute respiratory syndrome (SARS), Middle East respiratory syndrome (MERS), Ebola virus disease, coronavirus disease 2019 (COVID-19) and

more recently, chikungunya virus (CHIKV) outbreaks[4-9]. These events highlight that the threat of infectious diseases is not incidental but an enduring challenge. Consequently, the development of affordable, rapid, accurate and easily deployable diagnostic technologies is seriously needed for infectious disease prevention and control-particularly in resource-limited and low-income regions where such capabilities hold irreplaceable strategic value.

The COVID-19 pandemic has highlighted a persistent gap in diagnostic capabilities—lacking technologies that can simultaneously perform highly sensitivity, family-affordability, rapid turnaround and operational simplicity without reliance on specialized equipment[10]. Lateral flow immunoassays (LFAs)[11] offer simple, low-cost, rapid and instrument-free operation, yet their limited sensitivity restricts their use for early-stage pathogen screening. In contrast, nucleic acid amplification techniques represented by reverse transcription-polymerase chain reaction (RT-PCR) provide high sensitivity and flexible probe redesign for emerging variants[12], but require sophisticated temperature-control instruments, trained personnel and long assay times, making them unsuitable for low-cost, field-deployable and rapid testing. Alternative approaches based on SHERLOCK[13], DETECTR[14, 15], electrochemical[16, 17], colorimetric[18, 19] and optical signal transduction[20, 21] have further diversified the detection landscape, yet most still depend on nucleic acid pre-amplification or external readout instruments. Consequently, achieving high sensitivity, family-affordability, operational simplicity and rapidity within a single diagnostic platform remains a formidable challenge.

At the mechanistic level, this persistent trade-off arises from molecular recognition events in most high-sensitivity assays do not directly generate macroscopic readable outputs. Instead, they must be transduced through instrument-dependent optical or electrical excitation and signal-processing steps, resulting in an indirect "microscopic molecular interaction–instrument modulation–macroscopic signal readout" pathway that fundamentally constrains the simultaneous achievement of sensitivity, affordability, rapidity and instrument-free deployment. In our previous study, we established a direct signal-transduction route in which CRISPR-Cas mediated target recognition drives interfacial wettability switching, converting microscopic molecular recognition events into macroscopic visible liquid motion readouts[22]. This route was initially implemented in a tube-based sensor, where signal readout was achieved by simply observing whether the liquid droplet fell upon tube inversion. That work served as a proof-of-concept platform, demonstrating that a direct coupling between molecular recognition and visual fluidic output could, in principle, bypass the conventional indirect instrument-dependent signal transduction chain.

However, conceptual feasibility alone does not suffice to establish a practical sensing methodology. The initial tube-based proof-of-concept platform still retained two intrinsic methodological limitations that restricted further development of this direct signal-transduction route toward a standardized and deployable diagnostic format. First, signal triggering in the tube format remained closely coupled to manual flipping, making the readout dependent on operational factors such as flipping angle, speed and consistency and therefore difficult to standardize for robust use. Second, although the tube system effectively demonstrated binary yes/no visual readout, it did

not provide a structured solution for organized parallel readout under shared-system conditions. In particular, when multiple targets need to be detected within a common fluidic environment, how to maintain signal independence, avoid threshold perturbation and preserve readout distinguishability remained unresolved.

Here, we address these methodological challenges by developing a chip-based "dam-break drainage" event sensing architecture that enables fixed-boundary, steady-state triggering together with organized parallel visual readout in a shared system. Specifically, we constructed a "reservoir–dam–channel–collector" geometry in which signal triggering is governed by boundary conditions and interfacial states after one-step sample loading, without requiring subsequent manual actuation. To further support shared-system multi-target detection, we integrated a multi-dam layout, anchored-and-releasable crRNA strategies and a directional self-replenishment module, thereby enabling independent triggering and distinguishable visual output of two pathogen targets on a single chip. Under these conditions, the single-plex chip achieved a detection limit of 10 aM within 2 min at a cost below 0.2 $ per test, while the duplex chip simultaneously detected IAV and IBV with 100 aM sensitivity for both targets within 5 min at a cost below 0.3 $ per test. Under the developed practical nasal swab workflow based on room temperature lysis and RNase inhibition, Clinical validation conducted using 40 nasal swab samples (including SARS-CoV-2, IAV and IBV) demonstrated that the accuracy of both the single-plex and duplex chips was in complete agreement with the RT-PCR. Together, these results show that methodological innovations in triggering control and shared-system readout organization can advance CRISPR-Cas mediated wettability switching detection beyond concept validation and toward a more standardized and practically deployable sensing methodology.

Comment 2. The manuscript lacks a systematic evaluation of sample preparation and matrix interference, and the RNase-related error risk requires stronger data support. Although the authors note that adding an RNase inhibitor can mitigate RNase contamination, key interference factors are not quantitatively assessed. The authors should perform a structured stability evaluation and report misclassification rates across conditions such as collection/storage.

Response: We sincerely thank the reviewer for raising this important point. We fully agree that, for an instrument-free nucleic acid test intended for real clinical samples, sample pretreatment, matrix interference and RNase-related false readouts are all critical to reliability and practical use. Although we compared throat swab and nasal swab pretreatment conditions and used these results to establish the final nasal swab workflow, our previous presentation mainly showed which conditions were feasible. It did not assess, in a sufficiently systematic way, how robust this workflow would be under realistic variation in sample handling. We are grateful to the reviewer for pointing this out. In the original manuscript, we had already observed that endogenous RNases in different sample matrices could strongly affect detection results. Throat swab samples required heat lysis together with RNase inhibitor (RRI) to suppress nonspecific probe degradation. In contrast, nasal swab samples could be detected reliably using room-temperature lysis together with RRI. Based on

these results, we selected nasal swabs for clinical validation.

However, we agree that this was still not enough to answer a more practical question: could variation in sample collection or short-term handling introduce a risk of misclassification? The reviewer's comment made us realize that it is not enough to show that the method works under one selected condition. We also need to show that the final workflow remains basically robust within the practical operating window that we propose.

In response, we added a stability study in the revised manuscript based on the final workflow of nasal swab + room-temperature lysis + RRI. Because fresh positive clinical nasal swab samples were difficult to obtain, we used fresh negative nasal swab samples from 20 volunteers as the matrix background. We then generated simulated samples by spiking in known amounts of target RNA. This produced 10 positive samples with Ct values from 28 to 34 and 10 negative samples with Ct values from 35 to 39. We then tested several short-term handling conditions. These included immediate processing after sampling, processing after 1 h at room temperature and processing after 2 h at room temperature. We selected these time points because they represent the most practical use conditions for this method. They cover immediate testing, short on-site delay and moderate delay caused by handling multiple samples in parallel. The results showed good short-term robustness (the added Fig. S24 of revised manuscript). Under tested conditions, all 10 negative nasal swab samples remained stably confined within the sample reservoir and no obvious nonspecific "dam-break drainage" event was observed. All 10 positive samples produced the expected "dam-break drainage" event. These results show that reliable detection was maintained under the tested short-term handling variations. They also support that the workflow has basic robustness within a 0–2 h operating window. Because this method is intended for rapid, instrument-free use in point-of-care and community settings, rather than centralized testing after long-term storage, we believe that this time range covers the most relevant practical scenarios for the present study.

We would also like to clarify the main purpose of these additional experiments. The central issue here is not the difference between the single-plex and duplex formats themselves. Rather, it is whether the sample matrix, especially endogenous RNases, could nonspecifically degrade the tri-block probes on the molecular dam in the absence of target and thereby cause false triggering. Because this risk acts at the molecular-dam interface, once the final nasal swab pretreatment workflow is shown to suppress this matrix-driven nonspecific probe degradation, this conclusion is, in principle, relevant to both the single-plex and duplex modules, provided that they use the same sample source and the same basic pretreatment logic.

We revised the related wording in the manuscript. Our previous description could have left the impression that simply adding RRI is enough to solve matrix interference in a general way. We agree that this would be an oversimplification. To avoid this misunderstanding, we now state more clearly that RRI is one necessary part of the current nasal swab workflow, but this does not mean that all sampling media, all storage times, or all sample matrices have been systematically validated. We also changed the wording from sample pretreatment optimization to workflow optimization based on the currently validated sample type and selected operation window.

We agree that a more complete pre-analytical stability framework will be important for the future translation of this method. As the present study is mainly intended to establish the method and provide initial clinical validation, in this revision we added a focused robustness evaluation of the final intended nasal-swab workflow that is most directly relevant to the central conclusions of the work. We also narrowed the related statements accordingly to avoid extending the methodological claims beyond the current evidence.

The corresponding modification has been made:

1.Page 17, lines 453-474

Given that, in practical use, the time from sample collection to testing may vary among samples and the RNase-related interference may also differ across patient-derived sample matrices, we further performed a short-term storage robustness evaluation based on the final nasal swab workflow for both single-plex and duplex chip (Fig. S25 and S26). A total of 60 samples were tested, including 20 SARS-CoV-2, 20 IAV and 20 IBV samples, with 10 positive and 10 negative samples in each group. We compared three handling conditions: immediate testing after collection and testing after 1 h or 2 h of storage at room temperature following collection. These time points were selected because they cover the most practically relevant operating window for the intended use of this method. This includes immediate testing, short on-site delay and moderate delay caused by parallel handling of multiple samples. Specifically, because fresh positive clinical nasal swab samples were difficult to obtain, we added known concentrations of target RNA into 10 fresh negative nasal swab samples collected from 20 volunteers to form simulated positive samples. This produced 10 positive samples with Ct values from 28 to 34 and 10 negative samples with Ct values from 35 to 39. The results showed that, under these short-term handling variations, both the single-plex and duplex chips correctly distinguished negative and positive nasal swab samples within their respective rapid decision times. All 10 negative samples remained stably confined in the sample reservoir, with no obvious nonspecific “dam-break drainage” event. In contrast, all 10 positive samples produced the expected “dam-break drainage” event. As this method is designed for rapid, instrument-free testing in family and community settings, the 0–2 h window reflects the main use scenario considered in this study. The proposed results show that the developed nasal swab workflow remained feasible and stable under short-term handling variation within this window. We have therefore described these data as a focused robustness evaluation of the developed workflow, rather than as a full pre-analytical stability study covering all sample media, storage times and matrix conditions.

Was added

2.Page 9, lines 249-250

To overcome this, we optimized the sample pretreatment protocol

Was replaced by

To reduce matrix-derived false triggering and identify a practical operating workflow, we optimized the sample pretreatment conditions for clinical swab samples.

3.Page 27; SI

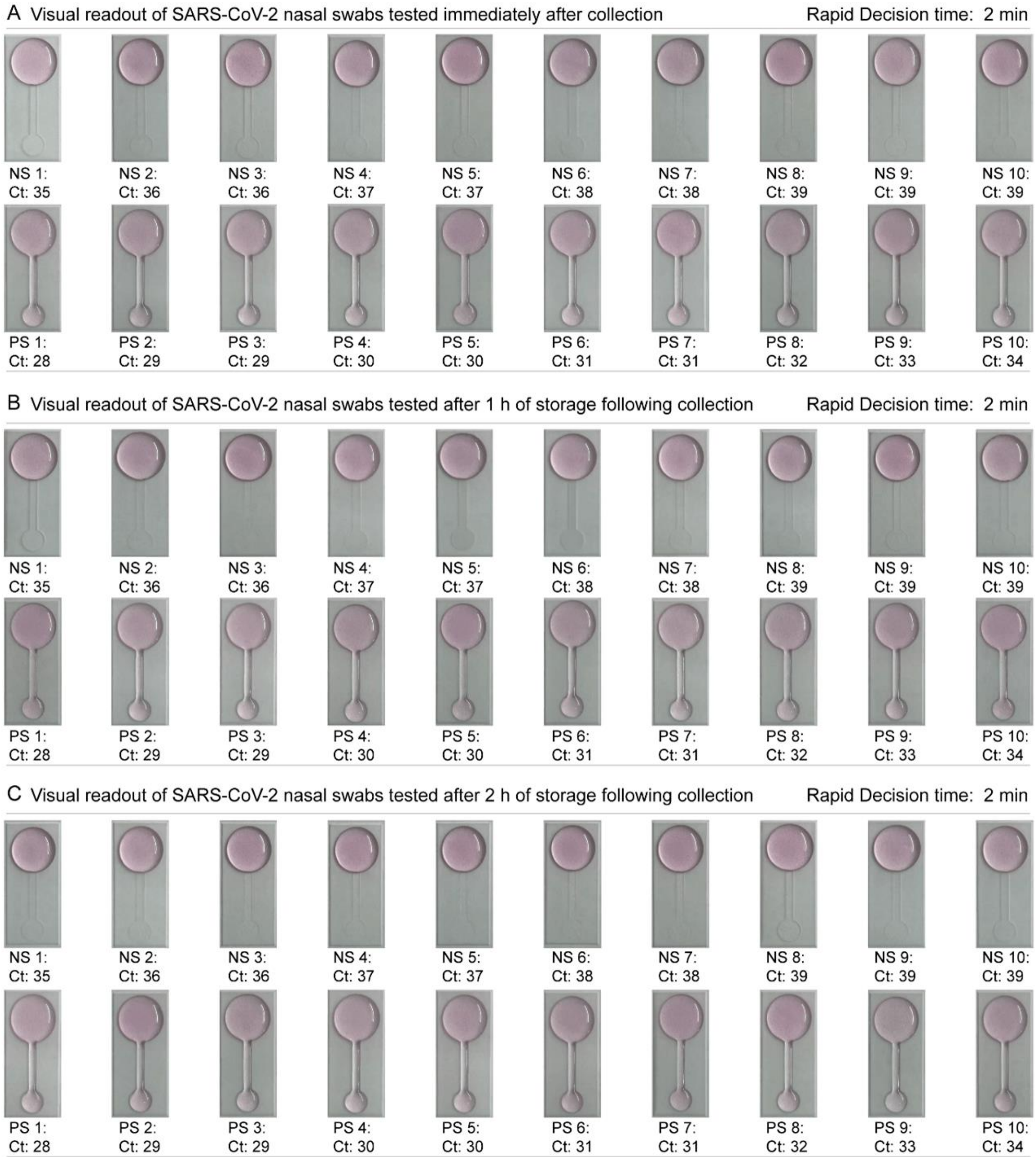

Supplementary Fig. 25. Detection results of the single-plex chip for SARS-CoV-2 nasal swabs after different storage durations. Visual readout immediately after collection (**a**), after 1 h storage (**b**) and after 2 h storage (**c**). NS denotes SARS-CoV-2-negative nasal swab samples (Ct=35–39), and PS denotes SARS-CoV-2-positive nasal swab samples (Ct=28–34). The corresponding RT–PCR Ct values are shown below each chip image.

Was added

4.Page 28; SI

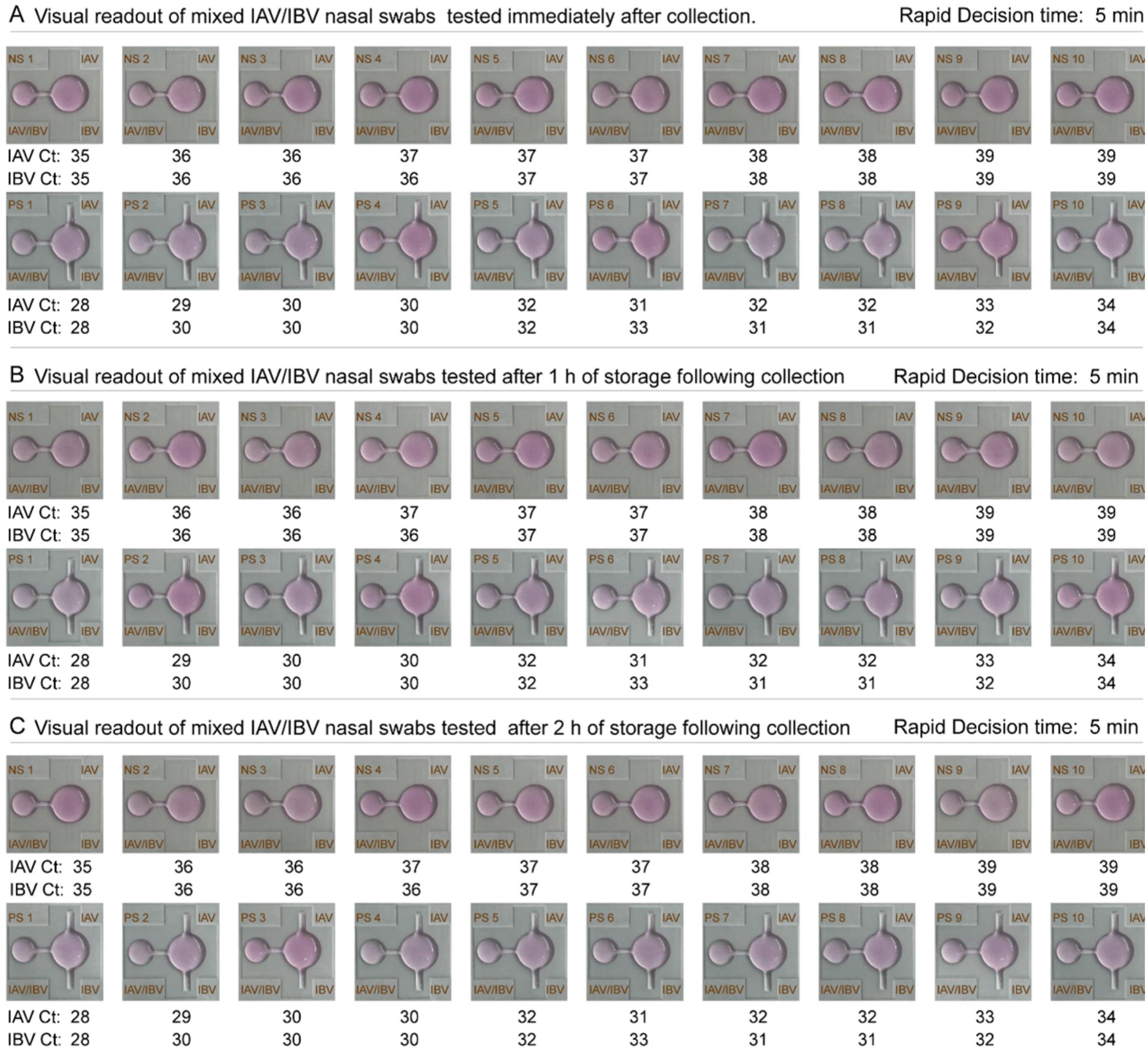

Supplementary Fig. 26. Detection results of mixed IAV and IBV nasal swabs after different storage durations. Visual readout of mixed IAV/IBV nasal swabs tested immediately after collection (**a**), after 1 h storage (**b**), after 2 h storage (**c**). NS indicates mixed nasal swabs classified as negative for both IAV (Ct=35-39) and IBV (35-39), whereas PS indicates pooled analysis of ten IAV-positive (Ct=28-34) and ten IBV-positive (Ct=28-34) nasal swabs. The RT-PCR Ct values corresponding to IAV and IBV nasal swabs are indicated below each chip image.

Was added

5.Page 9, lines 252-253

adding RRI to lysate was sufficient to inactivate RNases without the need for heating

Was replaced by

adding RRI to the lysate was sufficient to suppress matrix-derived RNase interference without the need for heating.

6.Page 9, lines 256-259

Based above, we select nasal swabs as sampling resource for easy operation and instrument-free detection.

Was replaced by

Based on these results, we selected nasal swabs processed by room-temperature lysis with RRI as the working sample workflow for subsequent clinical validation, owing to its operational simplicity, compatibility with Cas13a activity, and lower susceptibility to matrix-derived false triggering under the tested conditions.

Comment 3. The study remains primarily qualitative, and multiplexing is demonstrated only at the duplex level. Without broader experimental evidence, the platform's scalability and general applicability cannot be reliably assessed.

Response: We sincerely thank the reviewer for this important and constructive comment. We fully agree that the current detection format remains, in essence, a qualitative visual readout and that multiplex detection has only been experimentally validated at the duplex level. Therefore, without broader experimental support, it would not be sufficiently rigorous to describe the present work as a multiplex platform already proven to have broad scalability and general applicability. The reviewer's comment helped us recognize more clearly that the conclusions directly supported by the current data should be clearly separated from the platform's future potential.

We would like to clarify that the purpose of the duplex design in this study was not to claim that the platform has already been systematically proven to extend seamlessly to higher-order multiplex detection. Rather, it was intended to address a more fundamental question: within a shared reaction environment and a shared fluidic system, can different recognition events generate parallel, distinguishable and non-interfering visual outputs under fixed boundary conditions?

In fact, the original manuscript already showed that duplex detection is not a simple extension of the single-plex format. It requires solving two additional key problems. First, after the first target triggers "dam-break drainage" event, the resulting decrease in liquid volume can disrupt the threshold state required for the second target, thereby reducing sensitivity or even causing false-negative results. To address this issue, we introduced a directional transportation-based self-replenishment structure to maintain the liquid threshold state required for subsequent triggering. Second, in a shared system, both targets may activate Cas13a and cause probe degradation at both dam regions, which would compromise readout specificity. To solve this problem, we further introduced a dam-anchored and self-releasing crRNA design to perform local selective activation and the specificity of parallel readout. From this perspective, the duplex format presented here should be more accurately defined as a proof of principle for establishing parallel dual-target visual readout in a shared system, rather than as sufficient proof of scalability to higher-order multiplex detection.

We also acknowledge that some statements in the original Discussion and Conclusion extended beyond the current evidence. Phrases such as "new, versatile nucleic acid testing technology" and "generalizable bioanalytical tool for multi-analyte testing," as well as the discussion of possible extension to proteins, bacteria, exosomes and small molecules, were intended as forward-looking statements. However, we agree that, relative to the evidence provided in the present manuscript,

these statements were too broad and could give readers the impression that we had already achieved systematic validation for higher-order multiplexing or for a wider range of analyte classes.

We fully accept this criticism and have revised the manuscript accordingly. We now define the scope of the present work more explicitly as the establishment and validation of single-plex and duplex visual nucleic acid detection modes. We retain a limited discussion of future expansion, but now present it clearly as a prospective direction rather than as a conclusion supported by the current manuscript. We also state more explicitly in the Conclusion that the present study remains limited to qualitative detection, and that extension to higher-order multiplexing will require future systematic investigation. Therefore, we hope the revised manuscript now reflects the true scope of the present work more accurately.

The corresponding modification has been made:

1.Page 1, lines 11-22

The critical need for highly sensitive, rapid, low-cost and equipment-free pathogens diagnostics is acutely felt in resource-limited settings, such as in low-income region and at home, where existing methods force a compromise among these key metrics. We break this trade-off with a nucleic acid chip that leverages a novel "dam-break drainage" readout model and a CRISPR-Cas13a-mediated interfacial wettability switching sensing mechanism. This design unlocks both single-plex and duplex visual detection, delivering attomolar sensitivity (10 and 100 aM), rapid results (2 and 5 min) and low cost (0.20$ and 0.30$ per test), respectively. In a 40 clinical-samples validation for SARS-CoV-2, Influenza A virus and Influenza B virus, our platform demonstrated robust clinical concordance and successfully detected samples with ambiguous RT-PCR results (Ct=35). This work provides a powerful and accessible solution for next-generation point-of-care molecular testing.

Was replaced by

Here, we reported a stable "dam-break drainage" nucleic acid chip that supports both single-plex and duplex visual detection under fixed-boundary conditions after one-step sample loading, without requiring any subsequent instrument assistance or manual actuation. The chip achieves single-plex detection with a cost 0.2 $, a detection limit of 10 aM and a rapid decision time 2 min and duplex detection with a cost 0.3 $, a detection limit of 100 aM and a rapid decision time 5 min. We further developed a practical nasal swab workflow based on room-temperature lysis and RNase inhibition, under which the platform showed full concordance with RT-PCR in 40 clinical nasal-swab samples for SARS-CoV-2, Influenza A virus and Influenza B virus. By enabling both steady-state single-target detection and duplex parallel readout in a shared system, this work advances CRISPR-Cas-mediated wettability-switching detection from conceptual demonstration toward methodological establishment, and provides a new window for organized visual biosensing.

2. Page 2, lines 25-84

Infectious pathogens have persisted throughout human history, continuously posing serious threats to public health and social stability[1, 2]. Some pathogens display periodic outbreaks—such as

Influenza A (IAV) and B (IBV) viruses—that cause seasonal epidemics and significant healthcare burdens[3]. Meanwhile, newly emerging and highly pathogenic agents have repeatedly triggered sudden global health crises, including severe acute respiratory syndrome (SARS), Middle East respiratory syndrome (MERS), Ebola virus disease, coronavirus disease 2019 (COVID-19) and more recently, chikungunya virus (CHIKV) outbreaks[4-9]. These events highlight that the threat of infectious diseases is not incidental but an enduring challenge. Consequently, the development of affordable, rapid, accurate and easily deployable diagnostic technologies is seriously needed for infectious disease prevention and control-particularly in resource-limited and low-income regions where such capabilities hold irreplaceable strategic value.

The COVID-19 pandemic has highlighted a persistent gap in diagnostic capabilities—lacking technologies that can simultaneously perform highly sensitivity, low cost, rapid turnaround and operational simplicity without reliance on specialized equipment[10]. Lateral flow immunoassays (LFAs)[11] offer simple, low-cost, rapid and instrument-free operation, yet their limited sensitivity restricts their use for early-stage pathogen screening. In contrast, nucleic acid amplification techniques represented by reverse transcription-polymerase chain reaction (RT-PCR) provide high sensitivity and flexible probe redesign for emerging variants[12], but require sophisticated temperature-control instruments, trained personnel and long assay times, making them unsuitable for low-cost, field-deployable and rapid testing. Alternative approaches based on SHERLOCK[13], DETECTR[14, 15], electrochemical[16, 17], colorimetric[18, 19] and optical signal transduction[20, 21] have further diversified the detection landscape, yet most still depend on nucleic acid pre-amplification or external readout instruments. Consequently, achieving high sensitivity, family-affordability, operational simplicity and rapidity within a single diagnostic platform remains a formidable challenge.

Here, we reported a breakthrough sensing paradigm—converting target recognition into a visible “dam-break drainage” readout through a programmable interfacial wettability switch mediated by CRISPR-Cas13a system. Based on this paradigm, we developed a diagnostic chip architecture comprising a sample reservoir, a functional dam, a microfluidic channel and a collection zone. In the absence of the target, liquid is confined in the sample reservoir by the dam structure; upon target recognition, the solution breaches the dam and flows into the channel, enabling unambiguous signal interpretation. This architecture is further engineered as a single-plex chip for individual pathogen detection and a duplex chip for simultaneous identification of two distinct targets. Using SARS-CoV-2 as a model, the single-plex chip achieved a detection limit of 10 aM within 2 min, at a cost of less than $0.20 per test. The duplex chip, demonstrated by simultaneous detection of Influenza A and B viruses, attained sensitivity of 100 aM for both targets within 5 min, costing under $0.30 per test. Clinical validation using 40 nasal swab samples (including SARS-CoV-2, IAV and IBV) showed 100% concordance with RT-PCR results. Notably, the platform reliably detected positives even at high cycle thresholds (Ct=35), where conventional assays often fail. These results collectively highlight the developed chips as a highly sensitive, rapid, low-cost and equipment-free platform for pathogen screening, particularly suited for low-resource settings.

Was replaced by

Infectious pathogens have persisted throughout human history, continuously posing serious threats to public health and social stability[1, 2]. Some pathogens display periodic outbreaks—such as Influenza A (IAV) and B (IBV) viruses—that cause seasonal epidemics and significant healthcare burdens[3]. Meanwhile, newly emerging and highly pathogenic agents have repeatedly triggered sudden global health crises, including severe acute respiratory syndrome (SARS), Middle East respiratory syndrome (MERS), Ebola virus disease, coronavirus disease 2019 (COVID-19) and more recently, chikungunya virus (CHIKV) outbreaks[4-9]. These events highlight that the threat of infectious diseases is not incidental but an enduring challenge. Consequently, the development of affordable, rapid, accurate and easily deployable diagnostic technologies is seriously needed for infectious disease prevention and control-particularly in resource-limited and low-income regions where such capabilities hold irreplaceable strategic value.

The COVID-19 pandemic has highlighted a persistent gap in diagnostic capabilities—lacking technologies that can simultaneously perform highly sensitivity, family-affordability, rapid turnaround and operational simplicity without reliance on specialized equipment[10]. Lateral flow immunoassays (LFAs)[11] offer simple, low-cost, rapid and instrument-free operation, yet their limited sensitivity restricts their use for early-stage pathogen screening. In contrast, nucleic acid amplification techniques represented by reverse transcription-polymerase chain reaction (RT-PCR) provide high sensitivity and flexible probe redesign for emerging variants[12], but require sophisticated temperature-control instruments, trained personnel and long assay times, making them unsuitable for low-cost, field-deployable and rapid testing. Alternative approaches based on SHERLOCK[13], DETECTR[14, 15], electrochemical[16, 17], colorimetric[18, 19] and optical signal transduction[20, 21] have further diversified the detection landscape, yet most still depend on nucleic acid pre-amplification or external readout instruments. Consequently, achieving high sensitivity, family-affordability, operational simplicity and rapidity within a single diagnostic platform remains a formidable challenge.

At the mechanistic level, this persistent trade-off arises from molecular recognition events in most high-sensitivity assays do not directly generate macroscopic readable outputs. Instead, they must be transduced through instrument-dependent optical or electrical excitation and signal-processing steps, resulting in an indirect “microscopic molecular interaction–instrument modulation–macroscopic signal readout” pathway that fundamentally constrains the simultaneous achievement of sensitivity, affordability, rapidity and instrument-free deployment. In our previous study, we established a direct signal-transduction route in which CRISPR-Cas mediated target recognition drives interfacial wettability switching, converting microscopic molecular recognition events into macroscopic visible liquid motion readouts[22]. This route was initially implemented in a tube-based sensor, where signal readout was achieved by simply observing whether the liquid droplet fell upon tube inversion. That work served as a proof-of-concept platform, demonstrating

that a direct coupling between molecular recognition and visual fluidic output could, in principle, bypass the conventional indirect instrument-dependent signal transduction chain.

However, conceptual feasibility alone does not suffice to establish a practical sensing methodology. The initial tube-based proof-of-concept platform still retained two intrinsic methodological limitations that restricted further development of this direct signal-transduction route toward a standardized and deployable diagnostic format. First, signal triggering in the tube format remained closely coupled to manual flipping, making the readout dependent on operational factors such as flipping angle, speed and consistency, and therefore difficult to standardize for robust use. Second, although the tube system effectively demonstrated binary yes/no visual readout, it did not provide a structured solution for organized parallel readout under shared-system conditions. In particular, when multiple targets need to be detected within a common fluidic environment, how to maintain signal independence, avoid threshold perturbation and preserve readout distinguishability remained unresolved.

Here, we address these methodological challenges by developing a chip-based "dam-break drainage" event sensing architecture that enables fixed-boundary, steady-state triggering together with organized parallel visual readout in a shared system. Specifically, we constructed a "reservoir–dam–channel–collector" geometry in which signal triggering is governed by boundary conditions and interfacial states after one-step sample loading, without requiring subsequent manual actuation. To further support shared-system multi-target detection, we integrated a multi-dam layout, anchored-and-releasable crRNA strategies and a directional self-replenishment module, thereby enabling independent triggering and distinguishable visual output of two pathogen targets on a single chip. Under these conditions, the single-plex chip achieved a detection limit of 10 aM within 2 min at a cost below 0.2 $ per test, while the duplex chip simultaneously detected IAV and IBV with 100 aM sensitivity for both targets within 5 min at a cost below 0.3 $ per test. Under the developed practical nasal swab workflow based on room temperature lysis and RNase inhibition, Clinical validation conducted using 40 nasal swab samples (including SARS-CoV-2, IAV and IBV) demonstrated that the accuracy of both the single-plex and duplex chips was in complete agreement with the RT-PCR. Together, these results show that methodological innovations in triggering control and shared-system readout organization can advance CRISPR-Cas mediated wettability switching detection beyond concept validation and toward a more standardized and practically deployable sensing methodology.

3.Page 18, lines 476-498

In this study, we developed single-plex and duplex chip based on a target-activated CRISPR-Cas13a system that mediates a wettability switching at the solid-liquid interface, combined with a "dam-break drainage" readout. Both platforms exhibit three key advantages: First, the chips are fabricated using simple and low-cost processes, with total material expenses kept below $0.2 per test. This affordability makes them particularly suitable for large-scale screening in resource-limited settings and low-to-middle-income countries. Second, neither chip requires external temperature control or

complex instrumentation. They achieve attomolar-level sensitivity within minutes at room temperature, underscoring their utility for early pathogen detection and potential use in rapid at-home self-testing. Third, we demonstrated the high performance of both platforms in pooled testing, where they outperformed commercially available amplification kits in both sensitivity and speed. These results indicate the potential of our chips to serve as a new, versatile nucleic acid testing technology. One shortcoming of this method is that the current versions are limited to qualitative detection and do not support quantitative analysis. Future work will focus on developing quantification strategies and expanding multiplexing capability. It is also worth noting that while the current chips rely on CRISPR-Cas13a for nucleic acid recognition, the underlying design principle can be adapted to other classes of biomarkers. With ongoing advances in molecular biology and materials science, a wide range of targets—including proteins, bacteria, exosomes and small molecules—can induce detectable physical or chemical responses, such as pH changes, enzymatic activity modulation, or interfacial property shifts. By re-engineering the responsive module in the molecular probe, the platform could be extended to detect diverse biomarkers, positioning it as a generalizable bioanalytical tool for multi-analyte testing.

Was replaced by

In this study, we established single-plex and duplex "dam-break drainage" event chips based on CRISPR-Cas13a-mediated interfacial wettability switching and a visible macroscopic fluidic readout. The central advance of this work lies in creating a steady-state detection mode under fixed-boundary conditions, in which target recognition is directly converted into organized liquid-motion output without subsequent instrumentation or manual actuation. Compared with the previous tube-based implementation, the present chip architecture removes the operational uncertainty associated with manual flipping and enables stable signal triggering after one-step sample loading. In parallel, by establishing a practical nasal-swab workflow based on room-temperature lysis and RNase inhibition, we further show that this sensing strategy can operate reliably in clinical sample matrices rather than only under idealized reaction conditions. The significance of developed duplex module lies not merely in placing two targets on one chip, but in showing that parallel and distinguishable visual outputs can be organized under shared-system conditions. Achieving this required preserving the threshold state after the first triggering event and restoring local selectivity when both targets could activate Cas13a within the same reaction environment. The directional self-replenishment structure, together with the dam-anchored and self-releasing crRNA design, provides the structural basis for this organized duplex readout. At the same time, the current study remains limited to qualitative visual detection, and multiplexing has been experimentally established only at the duplex level. Further expansion to higher-order multiplexing will require systematic investigation of signal independence, threshold maintenance and readout organization in more complex shared systems. Likewise, although the underlying wettability switching readout principle may, in principle, be extendable to other analyte classes through appropriate upstream recognition modules, such broader applicability has not been experimentally demonstrated here. In this sense, the methodology

established in the present work provides a foundation for future exploration of higher-order multiplex visual biosensing based on interfacial wettability switching and macroscopic fluidic readout.

◆ Minor Comment

Comment 1. Figure 1: The readout rule is not clearly defined. Please clarify whether 2 min is the optimized decision time, how delayed drainage is handled (especially at low copy), and what calls apply beyond the time window (negative/invalid/retest).

Response: We sincerely thank the reviewer for this very important and careful comment. We fully agree that the description of the signal interpretation rule and the time window in Figure 1 was not sufficiently clear in the previous version. This could easily cause confusion between the optimized decision time, the later observation window and the specific meaning of results at different time points. In response, we have now added clear clarification in both the Figure 1 legend and the related text in the Results section.

For the single-plex detection mode in this study, the interpretation rule we established is a clear binary visual rule: samples that show a "dam-break drainage" event within 2 min are interpreted as positive, whereas samples that do not show this signal within 2 min are interpreted as negative. Here, the 2 min time point was not chosen arbitrarily, rather, it was defined as the optimized decision boundary based on the consistent experimental results obtained from all validated positive samples in the single-plex chip.

Specifically, across synthetic targets with different concentrations, pooled positive samples and clinical positive nasal swab samples, all samples that produced a true positive drainage signal completed "dam-break drainage" event within 2 min. In contrast, blank samples, pooled negative samples and clinical negative nasal swab samples all remained confined without specific "dam-break drainage" event throughout continuous observation up to 10 min. Therefore, in the present study, the 10 min observation window was not used to define delayed positives or to introduce additional interpretation categories such as positive, negative, invalid, or retest, instead, it was used to further confirm the reliability of 2 min as the decision boundary. In other words, our current data show that, in the single-plex system established here, all samples that should produce a specific positive signal do so within 2 min, whereas samples without "dam-break drainage" event within 2 min remain negative during continued observation up to 10 min. Therefore, the single-plex visual readout rule established in this work is essentially a clear rule based on a 2 min decision boundary, rather than a multistage interpretation system that depends on extended observation to reclassify results.

We sincerely apologize that this interpretation logic was previously only implicit in Fig. S6, Fig. S14, Movie S1 and Movie S2, rather than being stated explicitly in Figure 1 and the main text. Following the reviewer's suggestion, we have now clarified this point in the revised manuscript as follows: "2 min is the optimized decision time for single-plex detection, and samples without "dam-break drainage" event within 2 min are interpreted as negative; continued observation to 10 min

further confirms the stability of this decision boundary."

For the duplex chip, the same interpretation logic applies, but the optimized decision boundary is 5 min. We have therefore also clarified in the revised manuscript that the visual nucleic acid detection rule established in this study is based on a decision boundary of 2 min for single-plex detection and 5 min for duplex detection, whereas the later observation window is used mainly to confirm the stability of these decision boundaries, rather than to redefine delayed positive results.

The corresponding modification has been made:

1.Page 4, lines 101-109

Figure 1 | Working flow of the single-plex chip. (**a**) Signal readout for the negative sample: the test liquid is retained in the sample reservoir after a 2 min incubation. (**b**) Signal readout for the positive sample: the liquid breaches the hydrophobic dam and undergoes a "dam-break drainage" phenomenon within 2 min. (**c**) Chip structure and sensing pathway of the single-plex chip (**cI**); Architecture of the tri-block probe, consisting of a proximal hydrophilic moiety, a central responsive segment and a distal hydrophobic tail (**cII**). Mechanism of target RNA-induced tri-block probe cleavage by CRISPR-Cas13a mediation (**cIII**).

Was replaced by

Figure 1 | Working flow of the single-plex chip. (a) Negative readout of the single-plex chip: the test liquid remains confined in the sample reservoir within the optimized 2 min decision time. **(b)** Positive readout of the single-plex chip: the liquid breaches the hydrophobic dam and undergoes a visible "dam-break drainage" event within the optimized 2 min decision time. **(c)** Chip structure and sensing pathway of the single-plex chip **(cI)**; Architecture of the tri-block probe, consisting of a proximal hydrophilic moiety, a central responsive segment and a distal hydrophobic tail **(cII)**. Mechanism of target RNA-induced tri-block probe cleavage by CRISPR-Cas13a mediation **(cIII)**. Note: Under the validated performance of single-plex chip, both the blank and negative samples remained confined throughout a 10 min observation, while all positive samples with different concentration consistently trigger the "dam-break drainage" event within 2 min. Thus, 2 min is defined as the rapid decision time.

2.Page 9, lines 238-245

As shown in Fig. 3b, Fig. S5 and Movie S1, the chip enabled rapid (within 2 min), highly sensitive detection of the SARS-CoV-2 N gene over a wide concentration range (1 aM-10 pM), with a limit of detection (LOD) of 10 aM.

Was replaced by

As shown in Fig. 3b and Movie S1, the chip enabled rapid and highly sensitive detection of the SARS-CoV-2 N gene over a wide concentration range (1 aM–10 pM), with a limit of detection (LOD) of 10 aM. In three independent measurements (n=3), all tested positive groups consistently triggered visible "dam-break drainage" event within 2 min, whereas blank samples remained confined throughout the 10 min observation window, supporting the single-plex visual readout rule of rapid decision time (Fig. S6). As shown in Fig. S7 and Fig. S8, the developed chip outperforms both commercial RT-PCR kits (100 aM, 35 min) and pure CRISPR-Cas13a technology (10 pM, 40 min), exhibiting a lower detection limit and a faster readout.

3.Page 9, lines 260-261; Page 11, lines 275-278

As a result, all positive samples generated a "dam-break drainage" event within 2 min (Fig. S12a and Movie S2), while all negative samples were confined in the sample reservoir even after 10 min.

Was replaced by

As a result, all positive clinical nasal swab samples generated a clear "dam-break drainage" event, whereas pooled negative samples remained confined in the sample reservoir within 2 min and remained confined through the 10 min observation window (Fig. 3c and Movie S2). These results were confirmed by three independent measurements (n=3), further supporting the robustness of the developed workflow (nasal swab + room-temperature lysis + RRI) (Fig. S14a).

4.Page 8, lines 2-8; SI

Supplementary Fig. 5. Statistical analysis of detection times for the single-plex chip in detection of SARS-CoV-2 N gene across concentrations from 10 aM to 10 pM. All experiments were performed in three technical replicates (n=3), confirming reliable completion of detection within 2 minutes for all tested concentrations.

Was replaced by

Supplementary Fig. 6. Statistical analysis of readout times for the single-plex chip in detection of the SARS-CoV-2 N gene across concentrations from 10 aM to 10 pM. All experiments were performed in three independent measurements (n=3), showing that all tested positive concentrations triggered visible "dam-break drainage" event within 2 min rapid decision time, whereas blank samples remained confined throughout the 10 min observation window. Each orange data plot represents the mean detection time at the indicated concentration, and error bars indicate the range of detection times observed across three independent experiments.

5.Page 16, lines 2-10 SI

Supplementary Fig. 12. (**a**) The single-plex chip exhibited a detection time of less than 2 minutes for clinical samples with RT-PCR Ct values from 28 to 33. All experiments were performed in three independent measurements (n=3), confirming the robustness of its rapid detection performance (Note: Samples 1-10 represents the order of positive samples, Sample 11 represents the negative sample).

Was replaced by

Supplementary Fig. 14. (**a**) Detection-time analysis of the single-plex chip for clinical nasal-swab samples with RT-PCR Ct values from 28 to 33. All experiments were performed in three independent measurements (n=3), showing that all positive samples triggered visible "dam-break drainage" event within 2 min rapid decision time, whereas the negative sample remained confined in the sample reservoir throughout the 10 min observation window. Each black dot indicates the RT-PCR Ct value of an individual sample, whereas each orange dot indicates the mean detection time of the corresponding sample on the chip; green error bars denote the range of detection times observed across three independent experiments. (Note: Samples 1–10 denote the order of positive samples, and Sample 11 denotes the 10 in 1 negative sample.)

6. Page 14, lines 362-375

Figure 6 | Duplex chip evaluation for Influenza A and B virus detection. (**a**) Schematic illustration of the workflow for duplex detection of IAV and IBV on the duplex chip. (**b**) Representative detection outcomes for negative, IAV-positive, IBV-positive and dual-positive samples. The "dam-break drainage" signal appears exclusively at the corresponding channel within 5 min, indicating high target specificity. (**c**) Analytical sensitivity assessment using serial dilutions (10 aM–10 pM) of synthetic IAV-PB2 and IBV-PB1 RNA targets at equal concentrations. (**d**) Clinical validation with nasal swab samples: pooled analysis of ten IAV-positive (Ct=28-33) and ten IBV-positive (Ct=28-33) clinical specimens showed accurate target identification without cross-reactivity. (**e**) 10 in 1 pooled testing: the negative pool produced no drainage signal, whereas pools containing a single IAV-positive or IBV-positive sample generated distinct signals on their respective sides within 5 min. Dual-positive pools triggered synchronous drainage signals on both channels. (**f**) Comparative analysis of detection performance across the duplex chip, RT-PCR and CRISPR-Cas13a assays, confirming consistent diagnostic accuracy and reliability.

Was replaced by

Figure 6 | Duplex chip evaluation for Influenza A and B virus detection. (**a**) Schematic illustration of the workflow for duplex detection of IAV and IBV on the duplex chip. (**b**) Representative detection outcomes for negative, IAV-positive, IBV-positive and dual-positive samples. The "dam-break drainage" event appears exclusively at the corresponding channel within 5 min. (**c**) Sensitivity assay for IAV-PB2 and IBV-PB1 RNA pooled samples with concentration ranging from 10 aM–10 pM in 5 min rapid decision time. (**d**) Clinical validation with nasal swab samples: pooled analysis of ten IAV-positive (Ct=28-33) and ten IBV-positive (Ct=28-33) clinical specimens showed accurate target identification without cross-reactivity. (**e**) 10 in 1 pooled testing: the negative pool produced no "dam-break drainage" event, whereas pools containing a single IAV-positive or IBV-positive sample generated clear "dam-break drainage" readout on their corresponding channel within 5 min rapid decision time. Dual-positive pools present "dam-break drainage" events on both channels. (**f**) Comparative analysis of detection performance across the duplex chip, RT-PCR and CRISPR-Cas13a assays in sensitivity and detection time. Fold changes denote the normalized relative abundance of the corresponding target estimated from RT-PCR Ct values, using the positive sample with Ct=34 as the reference (set to 1) for each target. Note: For the duplex assay, blank and negative controls showed stable liquid confinement over 10 min observation period, whereas all target-positive samples, across the tested concentration range, produced a "dam-break drainage" event within 5 min. Accordingly, 5 min was defined as the result rapid decision time.

7.Page 16, lines 421-429

As shown in Fig. 6c, S15 and Movie S7, when IAV-PB2 and IBV-PB1 RNA targets were mixed at equal concentrations within the range of 10 aM to 10 pM, the duplex chip successfully distinguished the two targets independently within 5 min, maintaining a detection limit of 100 aM. The detection performance of the duplex chip rivals that of conventional RT-PCR assays (Fig. S16 and Fig. S17).

Was replaced by

As shown in Fig. 6c and Movie S7, the duplex chip allowed IAV-PB2 and IBV-PB1 RNA targets to be simultaneously detected when they were mixed at equal concentrations, with an LOD of 100 aM. In three independent measurements (n=3), each positive group consistently generated visible "dam-

break drainage" event within the 5 min readout period, while blank sample showed no "dam-break drainage" event and remained confined during the 10 min observation window, validating the duplex visual interpretation rule of 5 min rapid decision time (Fig. S17). The sensitivity of the duplex chip rivals that of conventional RT-PCR assays (Fig. S18 and Fig. S19) and vastly surpass the pure CRISPR-Cas13a system (Fig. S20). Moreover, the decision time of the developed duplex chip is 5 min, which is much shorter than that of the compared two methods.

8.Page 16, lines 432-436

Both detection channels produced distinct "dam-break drainage" signals within 5 min, whereas negative samples were confined in the sample reservoir without any "dam-break drainage" signal even after 10 min (Fig. 6d, Fig. S20a and Movie S8).

Was replaced by

As a result, both channels produced distinct "dam-break drainage" events within 5 min, whereas negative samples were confined in the sample reservoir without any "dam-break drainage" event even after 10 min (Fig. 6d and Movie S8). The reproducibility of these results was verified across three independent measurements (n=3), demonstrating the robustness of the dup-plex visual assay in clinical sample matrices under the developed working sample flow (Fig. S23a).

9.Page 19, lines 2-9; SI

Supplementary Fig. 15. Statics of detection time for duplex chip in simultaneously analyzing the IAV-PB2 and IBV-PB1 genes. Detection time of the duplex chip for concurrent analysis of the IAV-PB2 and IBV-PB1 genes over concentrations ranging from 100 aM to 10 pM, with both targets detected within 5 minutes. All measurements were conducted in triplicate (n=3).

Was replaced by

Supplementary Fig. 17. Statics of detection time for duplex chip in simultaneously analyzing the IAV-PB2 and IBV-PB1 genes. Detection time of the duplex chip for concurrent analysis of the IAV-PB2 and IBV-PB1 genes over concentrations ranging from 100 aM to 10 pM. In three independent measurements (n=3), visible "dam-break drainage" event was consistently observed for all tested positive concentrations within 5 min rapid decision time, while blank samples remained confined throughout the 10 min observation window. Each orange data plot indicates the mean detection time at the indicated concentration on the chip, and error bars denote the range of detection times observed across three independent experiments.

10.Page 25, lines 2-10; SI

Supplementary Fig. 20. (**a**) Statistical analysis of detection times for the duplex chip in simultaneous detection of IAV and IBV clinical samples. All experiments were performed in three independent measurements (n=3), confirming the robustness of its rapid detection performance (Note: Samples 1-10 represents ten groups of randomly mixed IAV RT-PCR-positive (Ct=28-33) and IBV RT-PCR-positive samples (Ct=28-33), Sample 11 represents the negative sample).

Was replaced by

Supplementary Fig. 23. (**a**) Statistical analysis of detection times for the duplex chip in simultaneous detection of IAV and IBV clinical samples. In three independent measurements (n=3), all positive samples reproducibly triggered visible "dam-break drainage" event within 5 min rapid decision time, whereas the negative sample remained confined in the sample reservoir throughout the 10 min observation window. Each blue data plot indicates the mean detection time of the corresponding sample on the chip, and error bars denote the range of detection times observed across three independent experiments. (Note: Samples 1-10 represents ten groups of randomly mixed IAV RT-PCR-positive (Ct=28-33) and IBV RT-PCR-positive samples (Ct=28-33), Sample 11 represents the 10 in 1 negative sample).

Comment 2. Figure 2(e): The parameter φ is not adequately defined or measured. Please define φ, describe the measurement method, and report fabrication tolerances/statistics to support reproducibility and sensitivity analysis.

Response: We sincerely thank the reviewer for this very careful and important comment. We fully agree that, in Figure 2(e), the structural parameters used to support the analysis of threshold-droplet formation and sensitivity must be clearly defined. If their definition, measurement method and statistical basis are not clearly described, readers may reasonably question the reproducibility and engineering reliability of this part of the study. The reviewer's comment prompted us to re-examine the logical relationship between Figure 2(d) and Figure 2(e), as well as the way these parameters were described.

After carefully checking the original manuscript, we found that there is in fact no parameter φ in the current version. We therefore suspect that the reviewer's mention of "φ" may reflect either a typographical error or a general reference to the relevant geometric parameters.

Following the reviewer's suggestion, we have further clarified the logic of this part in the revised manuscript. Figure 2(d) is mainly used to present a volume-dependent force-balance model. As the liquid volume in region A increases, the contact angle θ at the liquid–dam interface increases, and the liquid–vapor interfacial force term $F_{lv}\cos\theta$ decreases. This progressively breaks the original force balance and eventually drives forward liquid flow. Figure 2(e), in contrast, experimentally validates this volume-dependent threshold-regulation mechanism. It is also used to determine the final retained volume (600 μL) and the corresponding structural parameters that can stably maintain the threshold state.

At the same time, we also realized that the original manuscript did not clearly explain the reproducibility and statistical basis of the results shown in Figure 2(e). In fact, each threshold-state result shown in Figure 2(e) was obtained from three independent experiments, and the figure presents representative results. This point was not explicitly stated in the original version. Following the reviewer's suggestion, we have now added this repeat-experiment information in the revised manuscript and have further clarified the reproducibility of the retained-volume and structural-optimization conclusions.

Based on these revisions, we have further clarified in both the main text and the figure legend the relationship between Figure 2(d) and Figure 2(e). The logic of this part is now more clearly

presented: Figure 2(d) explains the principle of volume-dependent force balance and threshold regulation, whereas Figure 2(e) experimentally validates this mechanism and further identifies the retained volume and corresponding structural parameters that can stably maintain the threshold state.

The corresponding modification has been made:

1.Page 6, lines 156-161

Consequently, the liquid in A region overflow into C and D regions. However, the testing liquid volume of 100 μL in A region was insufficient to form the desired hemispherical "threshold" droplet.

Was replaced by

As a result, modification with 1 μM tri-block probe in 60 min allowed the B region to completely prevent leakage of 100 μL liquid from the A region, with 100% success across 12 chips (Fig. 2eI and Fig. S3). However, although 100 μL could still be held at the threshold state, this retained volume was too small to produce a sufficiently sustained and visible "dam-break drainage" event after triggering. In other words, the liquid was exhausted before a clear macroscopic "dam-break drainage" event could fully develop.

2.Page 6, lines 170-186

Similarly, a standalone physical dam also fails to establish this threshold state and hard to perform sensing (Fig. 2cII and 2eII). Specifically, when the liquid volume in region A was 80 μL, the liquid in A region was subjected to forces as depicted in Fig. 2 dII:

$$F_{PD} + F_{sl} + F_{lv}cos\theta = F_{SV} \quad (3)$$

where F_{PD} represents the resistance provided by a standalone physical dam. Under this condition, the liquid remained stably confined within region A. However, when the liquid volume exceeded 80 μL, the force balance in region A changed to:

$$F_{PD} + F_{sl} + F_{lv}cos\theta < F_{SV} \quad (4)$$

As a result, the liquid from A region spilled over into C and D regions (Fig. 2eII). To bridge this gap, we developed a composite dam (Fig. 2cIII).

Was replaced by

Similarly, a standalone physical dam also fails to establish a threshold state to generate a sufficiently continuous and obvious "dam-break drainage" event readout and hard to perform sensing (Fig. 2cII and 2eII). Specifically, when the liquid volume in A region was 80 μL, the liquid in A region was subjected to forces as depicted in Fig. 2 dII:

$$F_{PD} + F_{sl} + F_{lv}cos\theta = F_{SV} \quad (3)$$

where F_{PD} represents the resistance provided by a standalone physical dam. Under this condition, the liquid remained stably confined within A region. However, when the liquid volume exceeded 80 μL, the force balance in A region changed to:

$$F_{PD} + F_{sl} + F_{lv}cos\theta < F_{SV} \quad (4)$$

As a result, the liquid from A region spilled over into C and D regions (Fig. 2eII). Together, these analyses indicate that Fig. 2d establishes a volume-dependent force balance model for threshold

state regulation: as the retained liquid volume increases, the contact angle θ correspondingly increases, the $F_{lv}\cos\theta$ decreases, and the original balance is progressively destabilized until forward "dam-break drainage" event occurs. Guided by this model, we next asked how structural design in B region could increase the retained liquid volume while still maintaining the system at a clear "dam-break drainage" event threshold. To address this, we developed a composite dam (Fig. 2cIII).

Comment 3. Figure 5(c) proposes a 6U spacer on the crRNA to expand Cas13a's effective cleavage range, but this is supported mainly by a schematic. Please add a with/without-spacer comparison and quantify the impact (e.g., trigger time, detection rate, or LOD).

Response: We sincerely thank the reviewer for this very important and constructive suggestion. We fully agree that, in the original manuscript, the discussion in Figure 5(c) that the 6U linker expands the effective cleavage range of Cas13a was supported mainly by a mechanistic schematic, without a direct with/without-linker comparison. The reviewer's comment made us realize that, as this part serves as a preliminary optimization step before establishing the final duplex chip configuration, it should be supported by more direct experimental evidence to explain why the crRNA design containing the 6U linker was ultimately selected.

We would like to clarify that the purpose of Figure 5 is not to provide a full validation of the final duplex platform performance. Rather, like our earlier optimization of dam width, dam height and probe concentration, this part is intended to screen key molecular design elements that affect duplex implementation. In other words, the central question here is whether, before entering the formal duplex chip experiments in Figure 6, the crRNA design containing the 6U linker provides higher target-triggered molecular cleavage efficiency than the crRNA design without the linker, and is therefore more suitable for the final duplex configuration.

Following the reviewer's suggestion, we added new comparison experiments in the revised manuscript to evaluate how amino-crRNA without the 6U linker, amino-6U-crRNA containing the 6U linker, and conventional crRNA affect Cas13a collateral effect. We first compared these three crRNA designs in free solution. Results from three independent experiments showed that all three crRNA types exhibited essentially Cas13a collateral efficiency. This indicates that amino modification of the crRNA does not impair Cas13a collateral efficiency, and that introduction of the 6U linker also does not affect Cas13a collateral efficiency in free solution (Fig. 5cII). We then further examined how surface-immobilized amino-crRNA without the 6U linker and surface-immobilized amino-6U-crRNA containing the 6U linker affect Cas13a collateral efficiency. To make this comparison consistent with the actual chip-working condition, we used the same glass substrate and immobilization procedure as those used in the chip system. Specifically, the two crRNA designs were immobilized onto glass surfaces using the same method as in chip fabrication. The modified glass substrates were then cut into small disks with a diameter of 6.5 mm to fit a 96-well microplate reader. During the assay, all other components of the CRISPR–Cas13a reaction system were kept the same, and only the surface-immobilized crRNA design differed between the two groups. The glass disks carrying immobilized crRNA without linker or with linker were added

into the corresponding reaction solutions, and the fluorescence reporter-cleavage signal was monitored in real time using a microplate reader. Each group included three parallel experiments. Through this design, we were able to directly compare the target-triggered molecular cleavage efficiency of the two crRNA configurations under surface-immobilized conditions, rather than simply comparing their enzymatic activity in free solution.

The results showed that, compared with the design without linker, introduction of the 6U linker markedly improved the Cas13a-mediated molecular cleavage efficiency in the surface-immobilized system (Fig. 5cIII). In our view, the reason is not simply that the modified crRNA retains basic activity, but that the two designs behave differently in space after target activation. In the design without the 6U linker, the crRNA remains immobilized on the glass surface throughout the process. As a result, the target-activated Cas13a–crRNA complex remains confined near its original position and can mainly act on nearby regions. In contrast, in the design containing the 6U linker, the linker is rapidly cleaved by activated Cas13a after target recognition. This releases the originally localized Cas13a–crRNA complex from its confined state and gives it greater freedom to act, allowing it to degrade hydrophobic molecular-dam probes located farther away. In other words, introduction of the 6U linker effectively expands the effective cleavage range of activated Cas13a in the surface-immobilized system, thereby improving the overall target-triggered cleavage efficiency. These results provide direct experimental support for the design logic proposed in Figure 5(c) and also explain why the duplex chip experiments in Figure 6 ultimately adopted the 6U linker-containing crRNA as the optimized working configuration.

The corresponding comparison results have now been added to the revised manuscript (see Fig. 5c). We have also further clarified in the Results section that Figure 5 presents the preliminary optimization process before establishing the duplex configuration, whereas Figure 6 presents the establishment and validation of the final duplex chip mode based on that optimization. We again sincerely thank the reviewer for this key suggestion. It not only helped us add direct experimental support for the 6U linker design, but also made the logical relationship between Figure 5 as preliminary optimization and Figure 6 as subsequent validation much clearer.

The corresponding modification has been made:

1.Page 15, lines 377-409

Although this strategy successfully ensures specificity, it introduces a new limitation: once the CRISPR-Cas13a complex is immobilized on the molecular dam, the activated Cas13a proteins are spatially confined and can only degrade the nearby tri-block probes (Fig. 5cI). This constraint significantly weakens the overall degradation efficiency and hampers the wettability transition of the dam surface, thereby limiting the detection sensitivity. To overcome this, we further optimized the crRNA design by inserting a Cas13a-responsive linker (6U) between the amino group and the crRNA sequence (Fig. 5cII). Upon target recognition, the activated Cas13a cleaves this linker, transiently releasing the local Cas13a-crRNA complex into a quasi-free state. This allows the

activated Cas13a to degrade a broader range of the tri-block probes surrounding the molecular dam, resulting in a more efficient wettability transition and markedly enhanced detection sensitivity.

Was replaced by

Although this strategy successfully ensures specificity, it arise two questions: (1) whether the amino modified crRNA will decrease the ability of Cas13a collateral efficiency; (2) How to improve the degradation efficiency of the immobilized CRISPR-Cas13a. Once the CRISPR-Cas13a complex is immobilized on the molecular dam, the activated Cas13a proteins are spatially confined and can only degrade nearby tri-block probes, which weakens the overall degradation efficiency and hampers the wettability transition of the dam surface. To overcome this, we further optimized the crRNA design by inserting a Cas13a-responsive linker (6U) between the amino group and the crRNA sequence (Fig. 5cI). Then, we conducted the comparison experiments to evaluate how the amino-crRNA without the 6U linker and the amino-6U-crRNA containing the 6U linker affect the Cas13a collateral efficiency using the conventional crRNA as reference. We first compared these three crRNA designs in free solution. Results from three independent experiments showed that the three crRNA types exhibited essentially the same Cas13a collateral efficiency in free solution (Fig. 5cII). This indicates that amino modification of the crRNA does not impair Cas13a collateral efficiency, and that introduction of the 6U linker also does not affect Cas13a collateral efficiency. We then further examined how surface-immobilized amino-crRNA without the 6U linker and surface-immobilized amino-6U-crRNA containing the 6U linker affect Cas13a collateral efficiency. To make this comparison consistent with the actual chip-working condition, we used the same glass substrate and immobilization procedure as those used in the chip system. Specifically, the two crRNA designs were immobilized onto glass surfaces using the same method as in chip fabrication. The modified glass substrates were then cut into small disks with a diameter of 6.5 mm to fit a 96-well microplate reader. During the assay, all other components of the CRISPR–Cas13a reaction system were kept the same, and only the surface-immobilized crRNA design differed between the two groups. The glass disks carrying immobilized crRNA without spacer or with spacer were added into the corresponding reaction solutions, and the fluorescence reporter-cleavage signal was monitored in real time using a microplate reader. Each group included three independent experiments. Through this design, we were able to directly compare Cas13a collateral efficiency of the two crRNA configurations under surface-immobilized conditions, rather than simply comparing their enzymatic activity in free solution. The results showed that the introduction of the 6U linker significantly increased molecular cleavage efficiency under surface-anchored conditions (Fig. 5cIII). Mechanistically, in the linker-free design, the activated Cas13a/crRNA complex remains locally tethered after target recognition and therefore mainly acts on nearby tri-block probes (Fig. 5cIV). In contrast, in the 6U-containing design, the activated Cas13a rapidly cleaves the linker, thereby

releasing the Cas13a/crRNA complex from this confined local state and enabling degradation of more distant tri-block probes (Fig. 5cV). Thus, we selected the amino-6U-crRNA as the optimized crRNA configuration for the subsequent duplex chip experiments.

2.Page 12, line 309

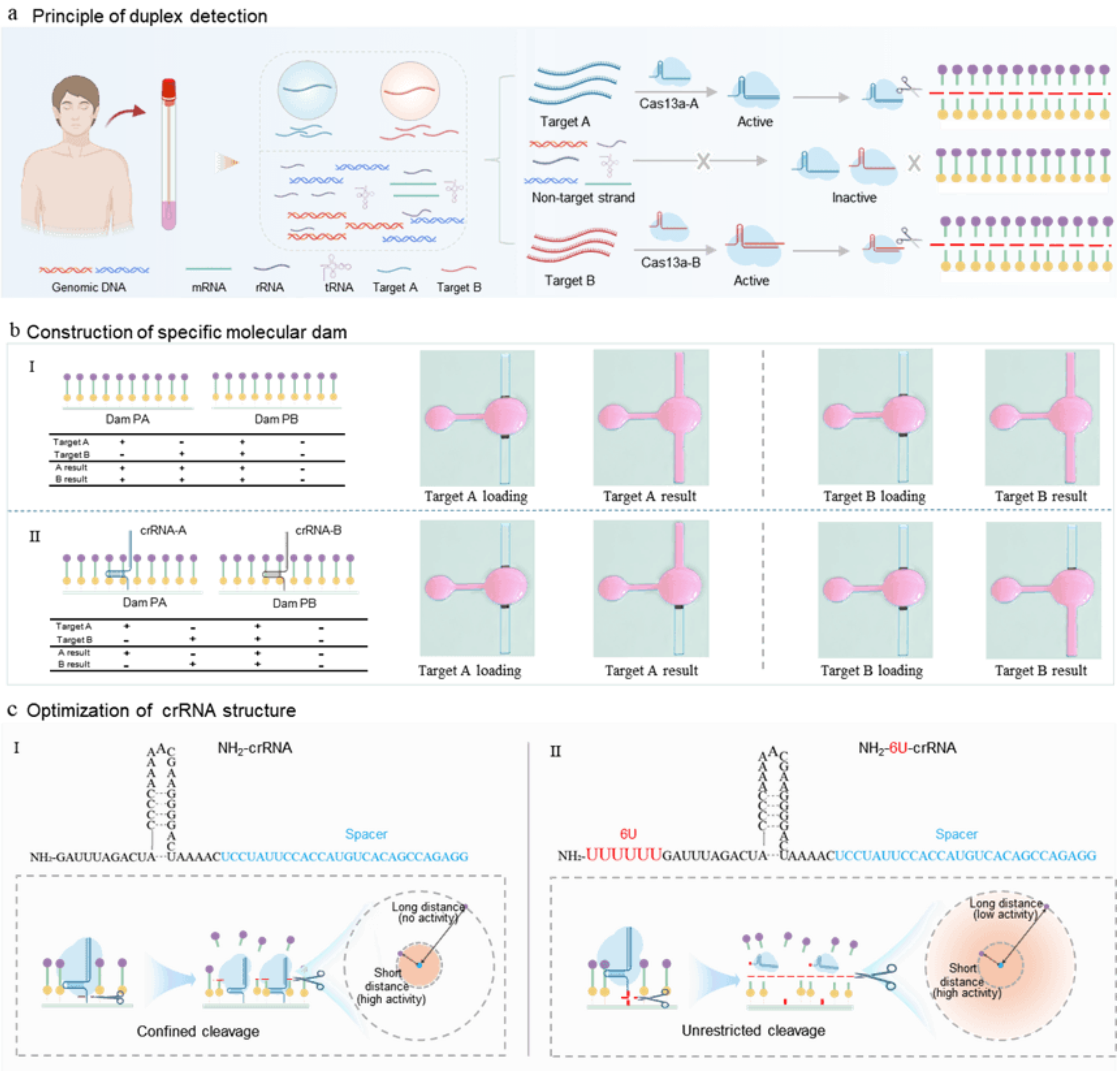

Was replaced by

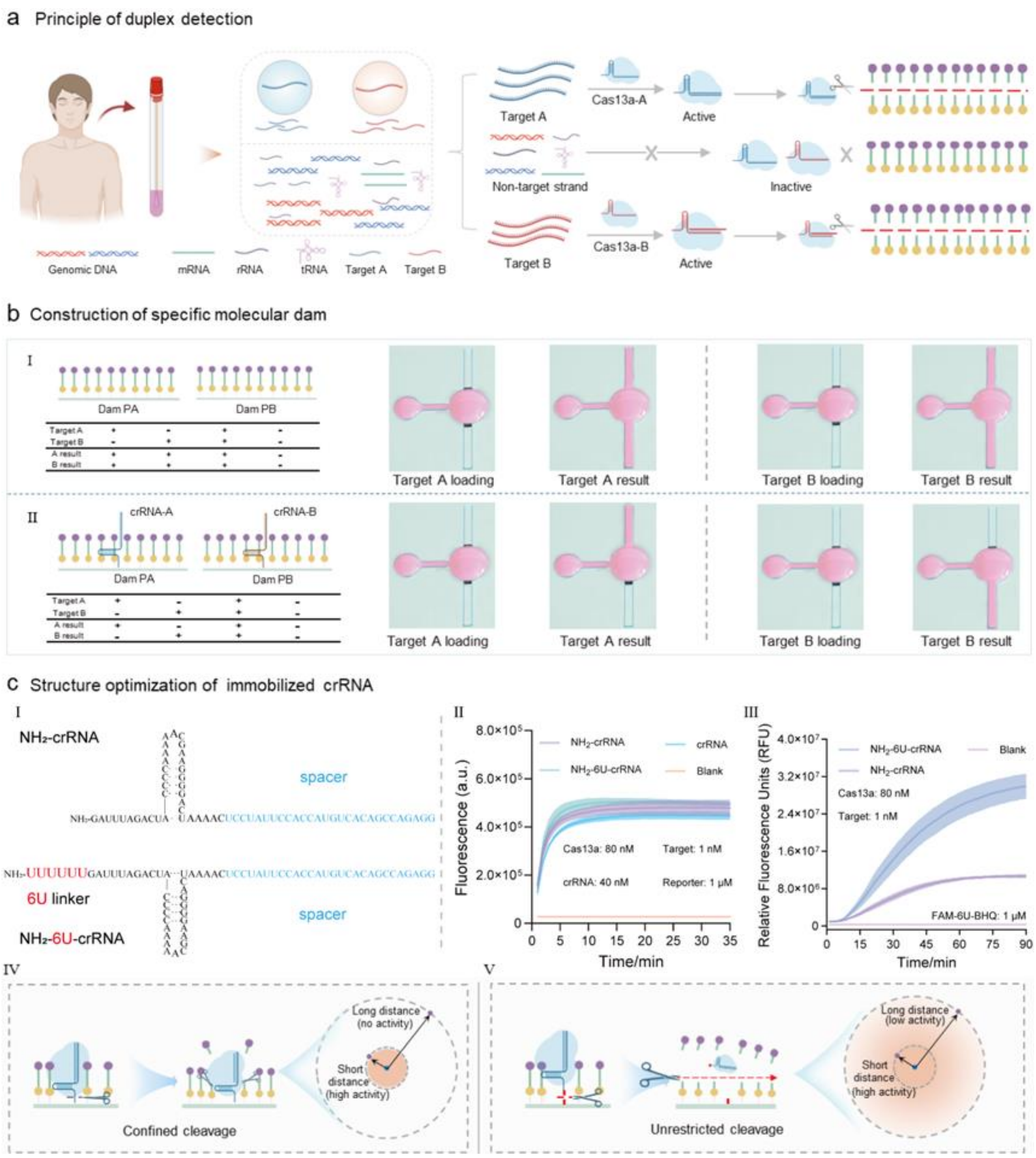

3.Page 12, lines 310-323

Figure 5 | Engineering specific molecular dams and optimizing crRNA architecture for duplex detection. (a) Working principle: two target RNAs orthogonally activate their respective CRISPR-Cas13a systems, leading to the specific cleavage of their corresponding tri-block probes on the molecular dams. **(b)** Formation of specific molecular dams: A non-specific dam, constructed from tri-block probes alone, is susceptible to be break by any activated Cas13a, leading to potential false-positive signals **(bI)**. A specific molecular dam, incorporating both tri-block probes and matched crRNA, ensures that drainage occurs only upon recognition of the intended target, thereby conferring specificity **(bII)**. **(c)** Overcoming spatial confinement of Cas13a. Direct immobilization of the crRNA anchors the activated Cas13a protein, limiting its collateral cleavage to adjacent tri-block probes **(cI)**. Engineering a 6U spacer that acts as a flexible tether between the amino group and crRNA allows partial mobility after Cas13a activation, thereby expanding the effective cleavage radius and enhancing tri-block probe degradation **(cII)**.

Was replaced by

Figure 5 | Engineering specific molecular dams and optimizing crRNA architecture for duplex detection. (**a**) Working principle: two target RNAs orthogonally activate their respective CRISPR-Cas13a systems, leading to the specific cleavage of their corresponding tri-block probes on the molecular dams. (**b**) Formation of specific molecular dams: A non-specific dam, constructed from tri-block probes alone, is susceptible to be break by any activated Cas13a, leading to potential false-positive signals (**bI**). A specific molecular dam, incorporating both tri-block probes and matched crRNA, ensures that drainage occurs only upon recognition of the intended target, thereby conferring specificity (**bII**). (**c**) crRNA design for duplex chip. (**cI**) Structures of the two crRNA designs: amino-modified crRNA and amino-6U-modified crRNA. (**cII**) Comparison of Cas13a collateral kinetics between amino-crRNA and amino-6U-crRNA in free solution. (**cIII**) Comparison of activated Cas13a-mediated cleavage efficiency between amino-crRNA and amino-6U-crRNA under surface-anchored conditions. Three independent kinetic traces are shown for each condition. The solid lines show the average fluorescence intensity obtained from three independent experiments, whereas the shaded areas denote the error band. Schematic comparison of linker-free and 6U-linker crRNA designs for duplex chip construction, showing that the linker-free design confines the activated Cas13a complex near the anchoring site (**cIV**), whereas the 6U-linker design allows activation-triggered release of the Cas13a/crRNA complex, thereby extending its effective cleavage range (**cV**).

Comment 4. Several key conclusions rely primarily on representative images and schematics, without sufficient replication, error estimates, or clear statistical definitions.

Response: We sincerely thank the reviewer for this highly comprehensive and very important comment. We fully agree that, if the key conclusions of a manuscript rely mainly on representative images and mechanistic schematics, without sufficient repeat experiments, error estimation and clear definitions of statistics and interpretation rules, the overall rigor and persuasiveness of the study will be weakened. The reviewer's comment was therefore extremely helpful to us. It prompted us not only to respond to each specific issue, but also to re-evaluate the completeness of the manuscript as a whole in terms of experimental support, repeat validation and statistical presentation.

In response to this general comment, we have systematically strengthened the revised manuscript in several aspects.

1. Clear definition of the single-plex readout rule. We further clarified the readout rule in Figure 1. Specifically, 2 min is now explicitly defined as the optimized rapid decision time, whereas 10 min is defined as the observation window used to confirm the continued confined state of negative samples. This avoids overreliance on representative images for result interpretation.

2. Additional repeat-experiment support for the single-plex readout rule. We incorporated into the main text information that had previously been shown mainly in the Supplementary Figures and Movies. We now state clearly that, across three independent experiments, positive samples with different target loads all triggered "dam-break drainage" event within 2 min, whereas blank and negative samples remained confined throughout the 10 min observation window. This provides direct support for the current visual readout rule.

3. Clarification of structural parameters and fabrication reproducibility in Figure 2. For the structural optimization shown in Figure 2, we further clarified the key structural parameters used

in the analysis. We also added the corresponding measurement methods, fabrication reproducibility statistics and error definitions. These additions strengthen the engineering reproducibility of the structural optimization and sensitivity analysis.

4. Direct experimental validation of the 6U linker design of crRNA in Figure 5. In the original manuscript, the discussion of the 6U linker was mainly based on a mechanistic schematic. Following the reviewer's suggestion, we added a direct with/without-linker crRNA comparison experiment. Under surface-immobilized conditions consistent with the chip system, we compared the target-triggered molecular cleavage efficiency of the two crRNA designs. As a result, Figure 5 is now supported not only by a schematic rationale, but also by direct experimental evidence as a preliminary optimization step.

5. Clearer logic between Figure 5 and Figure 6. We further emphasized in the main text that Figure 5 presents the molecular-design optimization process before establishment of the duplex chip configuration, whereas Figure 6 presents the establishment and validation of the final duplex chip mode based on that optimization. This makes the overall experimental logic of the manuscript clearer.

6. More systematic validation of sample preparation and matrix interference. For the final nasal swab + room-temperature lysis + RRI workflow, we added a more focused stability evaluation. This includes performance under short-term storage and handling delay conditions. We also provided statistical assessment of false-positive risk in negative samples and false-negative risk in low-positive samples. These additions strengthen the quantitative support for the clinical sample workflow.

7. Replenish the repeat number, error-bar definition and statistical description. In both the main text and the Supplementary Information, we have now replenished the repeat numbers, the definition of error bars, and the statistical descriptions used in the figures. This makes the presentation of the data clearer and more transparent.

8. Narrowing of conclusions that extended beyond the evidence. For parts of the original manuscript that may have appeared to rely too heavily on schematics or to extend the conclusions too broadly, we revised the Abstract, Introduction, Discussion and Conclusion accordingly. The conclusions of the revised manuscript are now more strictly aligned with the experimental evidence currently provided.

Through these revisions, we hope that the key conclusions of the revised manuscript no longer rely mainly on representative images and mechanistic schematics, but are instead supported by clearer interpretation rules, more complete repeat experiments, more transparent error and statistical definitions and newly added direct comparison experiments.

We again sincerely thank the reviewer for this very important general comment. It was precisely this reminder that allowed us to improve the manuscript at a broader level and significantly strengthen its quality in data presentation, statistical rigor and overall argumentation.

Comment 5. The manuscript contains multiple English issues (grammar, word choice, typos, and

formatting consistency). A thorough language edit is recommended.

Response: We sincerely thank the reviewer for this valuable suggestion regarding the language quality of the manuscript. As you suggested, we have carefully revised the manuscript throughout and performed a thorough check of the language and grammar. These revisions include corrections of grammatical errors, word choice, spelling and formatting consistency. We also refined parts of the sentence structure to improve the overall clarity and flow of the manuscript. We believe that these revisions have significantly improved the readability and professionalism of the paper. We again thank the reviewer for this careful and important suggestion.

Thank you for your nice comments and your recognition of our work again! Your professional and careful suggestions significantly improved our manuscript. Wish you a happy life and successful career!

Reviewer #1 (Remarks to the Author): We appreciate the authors' efforts in providing additional experiments, which have addressed some of our previous comments. However, several major concerns, including the claimed attomolar sensitivity, minute-scale detection speed, and mechanistic understanding, remain insufficiently addressed. The authors performed a tube-based enzyme kinetic analysis using a conventional fluorescence reporter and demonstrated a detection sensitivity in the picomolar range (e.g., 10 pM) (Supplementary Figures 8 and 20), which is consistent with the existing literature. In addition, they showed that CRISPR-Cas13a reactions using FAM-$(PEG6)_2$-6U-$(CH_2)_{12}$-BHQ and FAM–6U–BHQ reporters exhibited nearly identical fluorescence kinetic profiles (Supplementary Fig. 1), indicating that the $(PEG6)_2$-6U-$(CH_2)_{12}$-BHQ tri-block probe does not enhance the trans-cleavage activity of Cas13a. Therefore, the reported 10^6-fold improvement in sensitivity (from 10 pM to 10 aM) and the 30-fold reduction in detection time (from 60 minutes to 2 minutes) must be attributed entirely to the "Dam-Break Drainage" nucleic acid chip. The claimed detection limit of 10 aM RNA corresponds to approximately 60 RNA copies. Based on both the published literature (e.g., Huyke DA et al., Analytical Chemistry, 2022, 94(27): 9826–9834) and the authors' own tube-based kinetic data obtained with conventional fluorescence reporters, we are not convinced that such a small number of SARS-CoV-2 genomic RNA molecules can consistently diffuse to and activate sufficient Cas13a enzymes, resulting in enough cleavage of the immobilized probes to generate a reliable visual readout within only 2 minutes. Furthermore, the authors have not clearly explained how the "dam-break drainage" readout and wettability-switching mechanism achieve such dramatic signal amplification without nucleic acid pre-amplification. The current experimental data do not adequately support this conclusion. Additional characterization at the single-molecule level is needed to validate the proposed amplification mechanism. For example, direct evidence of Cas13a-mediated degradation of the immobilized tri-block probes, as well as a quantitative correlation between molecular cleavage events and the resulting macroscopic drainage behavior, would substantially strengthen the mechanistic basis for the claimed performance. In the absence of such evidence, it remains unclear how a small number of target RNA molecules can trigger a sufficiently large wettability change to produce a robust and reproducible visual signal.

Response: We sincerely thank you for the careful evaluation of our revised manuscript, and we appreciate the reviewer's acknowledgement that some of the additional experiments included in the previous revision have addressed part of the earlier concerns. We also take very seriously the reviewer's continued questions regarding the attomolar sensitivity, minute-scale readout and mechanism of the "dam-break drainage" system. After carefully considering these comments, we realized that the previous manuscript did not explain the origin of signal amplification in the chip with sufficient clarity, which may have led readers to attribute the 10 aM detection capability mainly to the enzymatic performance of Cas13a itself. In fact, the high performance of this system does not arise from an intrinsic enhancement of Cas13a trans-cleavage activity, but from the coupling between CRISPR-Cas13a-mediated molecular perturbation and chip-level threshold-triggered

fluidic amplification. Based on this understanding, we have reorganized and revised the relevant mechanistic descriptions in the Abstract, Introduction, Results and Discussion. We also added fluorescence confocal characterization using fluorescently labelled tri-block probes to directly verify target-activated CRISPR-Cas13a-mediated degradation of the immobilized probes on the chip surface. We hope that these additional data and revised mechanistic explanations now more clearly address the reviewer's concerns regarding detection sensitivity, rapid readout and signal amplification mechanism.

Comment 1: Based on both the published literature (e.g., Huyke DA et al., Analytical Chemistry, 2022, 94(27): 9826 – 9834) and the authors' own tube-based kinetic data obtained with conventional fluorescence reporters, we are not convinced that such a small number of SARS-CoV-2 genomic RNA molecules can consistently diffuse to and activate sufficient Cas13a enzymes, resulting in enough cleavage of the immobilized probes to generate a reliable visual readout within only 2 minutes. Furthermore, the authors have not clearly explained how the "dam-break drainage" readout and wettability-switching mechanism achieve such dramatic signal amplification without nucleic acid pre-amplification.

Response: After carefully considering the reviewer's comment, we agree that, in conventional CRISPR-Cas13a assays, the signal output mainly depends on the accumulation of cleaved reporter molecules. The detection sensitivity is therefore largely limited by the molecular reaction kinetics of Cas13a. Consistently, the tube-based control experiments that we added according to the reviewer's suggestion showed that, under the same crRNA, Cas13a and buffer conditions, the standalone CRISPR-Cas13a assay remained at the pM-level sensitivity.

We apologize that this point was not sufficiently clear in the previous version. The 10 aM detection achieved on the chip should not be interpreted as evidence that Cas13a itself reaches aM-level detection capability, or that the tri-block probe intrinsically enhances the enzymatic activity of Cas13a. Rather, the rapid and sensitive visual readout of the chip arises from the coupling between CRISPR-Cas13a-mediated molecular perturbation and chip-level threshold fluidic amplification. In this architecture, Cas13a collateral cleavage provides target-dependent tri-block probe perturbation, while the chip maintains the assay liquid in a threshold-confined state by regulating the force balance at the liquid–dam interface.

Specifically, in the study corresponding to Figure 2, we optimized the composite dam structure, dam height, dam width and loading volume to pre-position the assay droplet close to the drainage threshold before detection. In this state, the forward spreading tendency of the oversized droplet is nearly balanced by the wetting barrier at the hydrophobic molecular dam interface. In the absence of target, this barrier keeps the liquid stably confined. In the presence of target, activated Cas13a cleaves the immobilized tri-block probes and locally weakens the wetting barrier at the dam interface. Once this local perturbation breaks the interfacial balance, the threshold-confined droplet undergoes a nonlinear transition and generates a visible macroscopic "dam-break drainage" event.

This can be understood figuratively as the chip having been designed, through interfacial

wettability and chip structure, to pre-construct a near-critical “reservoir–dam” state: the reservoir can store liquid several times its own volume and is maintained near the drainage threshold. Under this state, local weakening of the molecular dam interface is analogous to opening a small breach in a dam that is already close to its critical point, which immediately triggers a flood-like signal release without needing to destroy the entire dam. Under this mechanism, a limited number of molecular cleavage events can trigger a fluidic response far larger than their own scale. This mechanism explains why limited molecular events can be converted into a larger-scale macroscopic fluidic response without nucleic acid pre-amplification. Therefore, the amplification process in this work is essentially not a “linear accumulation of molecular counts,” but rather a “macroscopic physical transition triggered by crossing a critical threshold.”

The structural optimization results (Fig.2) also support this interpretation. Stable detection was achieved only within a specific range of dam width and dam height. When the dam constraint was slightly increased, even target-containing samples could not break through the dam. If the signal were entirely derived from the linear accumulation of molecular cleavage products, a continuous change in response would be expected. In contrast, we observed an abrupt transition from complete confinement to rapid drainage, which is more consistent with a threshold-controlled instability process.

To make this mechanism clearer, we have revised the Abstract, Introduction, Results section describing the threshold-confined droplet, and the Discussion. In these revisions, we further clarified that the chip response results from the coupling between CRISPR-Cas13a-mediated molecular perturbation and chip-level threshold fluidic amplification. Together, these two processes account for the rapid response and high sensitivity of the chip.

The corresponding modifications has been made as shown below

1.Page 1, lines 16-18

Abstract: Here, we reported a stable “dam-break drainage” nucleic acid chip that supports both single-plex and duplex visual detection under fixed-boundary conditions after one-step sample loading, without requiring any subsequent instrument assistance or manual actuation. The chip achieves single-plex detection with a cost 0.2 $, a detection limit of 10 aM and a rapid decision time 2 min and duplex detection with a cost 0.3 $, a detection limit of 100 aM and a rapid decision time 5 min. We further developed a practical nasal swab workflow based on room-temperature lysis and RNase inhibition, under which the platform showed full concordance with RT-PCR in 40 clinical nasal-swab samples for SARS-CoV-2, Influenza A virus and Influenza B virus. By enabling both steady-state single-target detection and duplex parallel readout in a shared system, this work advances CRISPR-Cas-mediated wettability-switching detection from conceptual demonstration toward methodological establishment, and provides a new window for organized visual biosensing.

Was replaced by

Abstract: Here, we reported a stable “dam-break drainage” nucleic acid chip that supports both

single-plex and duplex visual detection under fixed-boundary conditions after one-step sample loading, without requiring any subsequent instrument assistance or manual actuation. The chip achieves single-plex detection with a cost 0.2 $, a detection limit of 10 aM and a rapid decision time 2 min and duplex detection with a cost 0.3 $, a detection limit of 100 aM and a rapid decision time 5 min. Mechanistically, the chip operates in a threshold-confined fluidic state, in which localized CRISPR-Cas13a-mediated wettability perturbations are converted into visible fluidic outputs through a threshold-triggered amplification process. We further developed a practical nasal swab workflow based on room-temperature lysis and RNase inhibition, under which the platform showed full concordance with RT-PCR in 40 clinical nasal-swab samples for SARS-CoV-2, Influenza A virus and Influenza B virus. By enabling both steady-state single-target detection and duplex parallel readout in a shared system, this work advances CRISPR-Cas-mediated wettability-switching detection from conceptual demonstration toward methodological establishment, and provides a new window for organized visual biosensing.

2.Page 3, lines 72-79; 89-95

Introduction: Here, we address these methodological challenges by developing a chip-based "dam-break drainage" event sensing architecture that enables fixed-boundary, steady-state triggering together with organized parallel visual readout in a shared system. Specifically, we constructed a "reservoir–dam–channel–collector" geometry in which signal triggering is governed by boundary conditions and interfacial states after one-step sample loading, without requiring subsequent manual actuation. To further support shared-system multi-target detection, we integrated a multi-dam layout, anchored-and-releasable crRNA strategies and a directional self-replenishment module, thereby enabling independent triggering and distinguishable visual output of two pathogen targets on a single chip. Under these conditions, the single-plex chip achieved a detection limit of 10 aM within 2 min at a cost below 0.2 $ per test, while the duplex chip simultaneously detected IAV and IBV with 100 aM sensitivity for both targets within 5 min at a cost below 0.3 $ per test. Under the developed practical nasal swab workflow based on room temperature lysis and RNase inhibition, Clinical validation conducted using 40 nasal swab samples (including SARS-CoV-2, IAV and IBV) demonstrated that the accuracy of both the single-plex and duplex chips was in complete agreement with the RT-PCR. Together, these results show that methodological innovations in triggering control and shared-system readout organization can advance CRISPR-Cas mediated wettability switching detection beyond concept validation and toward a more standardized and practically deployable sensing methodology.

Was replaced by

Introduction: Here, we address these methodological challenges by developing a chip-based "dam-break drainage" sensing architecture that enables fixed-boundary, steady-state triggering together with organized parallel visual readout in a shared system. The architecture is deliberately engineered to operate in a threshold-confined fluidic state, in which the assay liquid in reservoir is maintained

near a critical transition point prior to target introduction. Under this condition, target-activated Cas13a generates amplified molecular perturbations through collateral cleavage of tri-block probes, whereas the threshold-confined fluidic architecture converts the resulting wettability perturbation into a macroscopic "dam-break drainage" event through threshold-triggered fluidic amplification. Specifically, we constructed a "reservoir–dam–channel–collector" geometry in which signal triggering is governed by boundary conditions and interfacial states following one-step sample loading, without requiring any subsequent manual actuation. To further support shared-system multi-target detection, we integrated a multi-dam layout, anchored-and-releasable crRNA strategies and a directional self-replenishment module, thereby enabling independent triggering and distinguishable visual outputs of two pathogen targets on a single chip. Under these conditions, the single-plex chip achieved a detection limit of 10 aM within 2 min at a cost below $0.2 per test, while the duplex chip simultaneously detected Influenza A virus and Influenza B virus with a sensitivity of 100 aM for both targets within 5 min at a cost below $0.3 per test. Under a practical nasal-swab workflow based on room-temperature lysis and RNase inhibition, clinical validation using 40 nasal-swab samples containing SARS-CoV-2, Influenza A virus and Influenza B virus demonstrated complete agreement with RT-PCR. Together, these results demonstrate that coupling CRISPR-Cas-mediated molecular amplification with threshold-triggered fluidic amplification establishes a dual-stage signal amplification framework, enabling localized molecular events to be converted into robust macroscopic visual outputs. Combined with innovations in triggering control and shared-system readout organization, this framework advances CRISPR-Cas-mediated wettability-switching detection beyond conceptual validation toward a standardized, deployable and instrument-free sensing methodology.

3.Page 6, lines 150-159

Structural strategy to create a threshold-confined droplet. To improve chip sensitivity, we aimed to create a threshold-confined droplet that remained stably restricted in the sample reservoir while being readily triggered by target-induced probe cleavage. In this design, a droplet much larger than the underlying reservoir was maintained near the drainage threshold, allowing limited degradation of the tri-block probes to induce a clear "dam-break drainage" readout.

Was replaced by

Engineering a threshold-confined droplet for chip-level signal amplification. Although CRISPR-Cas13a collateral cleavage provides molecular signal amplification, this amplification alone is insufficient to support highly sensitive visual detection given its pM-level standalone sensitivity[26]. We therefore sought to introduce an additional chip-level amplification strategy by regulating the threshold state of the assay liquid. Specifically, we aimed to create a threshold-confined droplet, namely an oversized droplet that could remain stably restricted in the sample reservoir but was maintained close to the drainage threshold. In this threshold state, the forward spreading tendency of the oversized droplet is nearly balanced by the wetting barrier generated at

the hydrophobic dam interface: the barrier is sufficient to prevent spontaneous overflow in the absence of target, but small enough that limited target-induced degradation of the tri-block probes can easily break the balance, reduce the local wetting barrier and trigger a clear "dam-break drainage" readout.

4.Page 6, lines 161-163

To achieve this, both the E region and the B region were designed to be hydrophobic, so that the oversized droplet could be effectively confined without premature overflow or collapse beyond the A region. The process for E region hydrophobic modification began with a laser micromachining to create the micro-nanostructured surface, followed by chemical modification with fluoroalkyl silane (FAS-17) to introduce hydrophobic groups[26, 27] (Fig. 2aII). As shown in Fig. 2aI and Fig. 2aIII, the obtained E region displayed a superhydrophobic contact angle of 159.2° under a laser matrix scanning interval of 0.01 mm (Laser power and scanning speed are provided in Table S1). For the B region, hydrophobic modification was realized by covalently anchoring amine-modified tri-block probes onto the amine-modified B region via glutaraldehyde crosslinking (Fig. 2b).

Was replaced by

To achieve this, both the E region and the B region were designed to be hydrophobic, so that the oversized droplet could be effectively confined without premature overflow or collapse beyond the A region. The hydrophobic E region was designed to prevent lateral collapse of the oversized droplet, whereas the hydrophobic B region served as the tunable molecular dam that directly defined the drainage threshold. The process for E region hydrophobic modification began with a laser micromachining to create the micro-nanostructured surface, followed by chemical modification with fluoroalkyl silane (FAS-17) to introduce hydrophobic groups[26, 27] (Fig. 2aII). As shown in Fig. 2aI and Fig. 2aIII, the obtained E region displayed a superhydrophobic contact angle of 159.2° under a laser matrix scanning interval of 0.01 mm (Laser power and scanning speed are provided in Table S1). For the B region, hydrophobic modification was realized by covalently anchoring amine-modified tri-block probes onto the amine-modified B region via glutaraldehyde crosslinking (Fig. 2b).

4.Page 9, lines 230-235

Experimentally, when the physical dam height in the composite dam reached 0.27 mm, three independent chips consistently confined 600 μL of liquid within A region under the proposed conditions. This volume established an ideal threshold state that remained poised for target-triggered breakthrough and was sufficient to generate a clear "dam-break drainage" event (Fig. 2eVI).

Was replaced by

Experimentally, when the physical dam height in the composite dam reached 0.27 mm, three independent chips consistently confined 600 μL of liquid within the A region, whereas increasing

the loaded volume to 700 μL caused spontaneous breakthrough into the downstream regions (Fig. 2eVI). Thus, 600 μL represented the maximum stable retention volume immediately below the drainage threshold and was selected as the threshold-confined volume for sensing. Under this condition, the oversized droplet remained stable in the absence of target but was poised for target-triggered drainage, enabling limited molecular perturbation at the dam interface to be converted into a clear macroscopic "dam-break drainage" readout.

5.Page 9, lines 252-255

When the dam width is excessive, the wettability transition cannot propagate across the full width of the dam within the allowed time, and the liquid therefore remains confined in A region despite the presence of target nucleic acid. Therefore, we determined 90 μm in width and 0.27 mm in height as the optimal configuration for the physical dam.

Was replaced by

When the dam width is excessive, the wettability transition cannot propagate across the full width of the dam within the allowed time, and the liquid therefore remains confined in A region despite the presence of target nucleic acid. Together, these width-dependent results define the operational threshold window of the molecular dam, in which the assay liquid is stably confined without target but remains sensitive to target-induced weakening of the wetting barrier. Therefore, we determined 90 μm in width and 0.27 mm in height as the optimal configuration for the physical dam.

6.Page 18, lines 510-519

In this study, we established single-plex and duplex "dam-break drainage" event chips based on CRISPR-Cas13a mediated interfacial wettability switching and a visible macroscopic fluidic readout. The central advance of this work lies in creating a steady-state detection mode under fixed-boundary conditions, in which target recognition is directly converted into organized liquid-motion output without subsequent instrumentation or manual actuation. Compared with the previous tube-based implementation, the present chip architecture removes the operational uncertainty associated with manual flipping and enables stable signal triggering after one-step sample loading. In parallel, by establishing a practical nasal swab workflow based on room-temperature lysis and RNase inhibition, we further show that this sensing strategy can operate reliably in clinical sample matrices rather than only under idealized reaction conditions.

The significance of developed duplex module lies not merely in placing two targets on one chip, but in showing that parallel and distinguishable visual outputs can be organized under shared-system conditions. Achieving this required preserving the threshold state after the first triggering event and restoring local selectivity when both targets could activate Cas13a within the same reaction environment. The directional self-replenishment structure, together with the dam-anchored and self-releasing crRNA design, provides the structural basis for this organized duplex readout.

At the same time, the current study remains limited to qualitative visual detection, and

multiplexing has been experimentally established only at the duplex level. Further expansion to higher-order multiplexing will require systematic investigation of signal independence, threshold maintenance and readout organization in more complex shared systems. Likewise, although the underlying wettability switching readout principle may, in principle, be extendable to other analyte classes through appropriate upstream recognition modules, such broader applicability has not been experimentally demonstrated here. In this sense, the methodology established in the present work provides a foundation for future exploration of higher-order multiplex visual biosensing based on interfacial wettability switching and macroscopic fluidic readout.

Was replaced by

In this study, we established single-plex and duplex "dam-break drainage" event chips based on CRISPR-Cas13a mediated interfacial wettability switching and a visible macroscopic fluidic readout. The central advance of this work lies in creating a steady-state detection mode under fixed-boundary conditions, in which target recognition is directly converted into organized liquid-motion output without subsequent instrumentation or manual actuation. Compared with the previous tube-based implementation, the present chip architecture removes the operational uncertainty associated with manual flipping and enables stable signal triggering after one-step sample loading. In parallel, by establishing a practical nasal swab workflow based on room-temperature lysis and RNase inhibition, we further show that this sensing strategy can operate reliably in clinical sample matrices rather than only under idealized reaction conditions.

Mechanistically, the rapid and highly sensitive performance of the chip arises from the coupling of CRISPR-Cas13a-mediated molecular amplification with chip-level threshold conversion. Cas13a collateral cleavage provides target-dependent molecular perturbations, while the chip architecture regulates the force balance at the liquid–dam interface and maintains the assay liquid in a threshold-confined state. Under this condition, a limited reduction in the local wetting barrier is sufficient to break the interfacial balance and nonlinearly trigger a macroscopic "dam-break drainage" transition , establishing a threshold-dependent relationship between molecular probe degradation and macroscopic drainage behavior. This coupled amplification process enables molecular recognition events to be read out beyond the sensitivity range achievable by the standalone CRISPR-Cas13a assay, without requiring nucleic acid pre-amplification or external instrumentation.

The significance of developed duplex module lies not merely in placing two targets on one chip, but in showing that parallel and distinguishable visual outputs can be organized under shared-system conditions. Achieving this required preserving the threshold state after the first triggering event and restoring local selectivity when both targets could activate Cas13a within the same reaction environment. The directional self-replenishment structure, together with the dam-anchored and self-releasing crRNA design, provides the structural basis for this organized duplex readout.

At the same time, the current study remains limited to qualitative visual detection, and

multiplexing has been experimentally established only at the duplex level. Further expansion to higher-order multiplexing will require systematic investigation of signal independence, threshold maintenance and readout organization in more complex shared systems. Likewise, although the underlying wettability switching readout principle may, in principle, be extendable to other analyte classes through appropriate upstream recognition modules, such broader applicability has not been experimentally demonstrated here. In this sense, the methodology established in the present work provides a foundation for future exploration of higher-order multiplex visual biosensing based on interfacial wettability switching and macroscopic fluidic readout.

Comment 2: The current experimental data do not adequately support this conclusion. Additional characterization at the single-molecule level is needed to validate the proposed amplification mechanism. For example, direct evidence of Cas13a-mediated degradation of the immobilized tri-block probes, as well as a quantitative correlation between molecular cleavage events and the resulting macroscopic drainage behavior, would substantially strengthen the mechanistic basis for the claimed performance. In the absence of such evidence, it remains unclear how a small number of target RNA molecules can trigger a sufficiently large wettability change to produce a robust and reproducible visual signal.

Response: We sincerely appreciate the reviewer's thoughtful suggestion that more direct evidence is needed to support the proposed mechanism. To validate the cleavage capability of target-activated Cas13a toward immobilized tri-block probes, we prepared a substrate containing triangular positioning reference structures using the same fabrication process as that used for the molecular dam, and further modified the substrate with Cy3-labeled tri-block probes. The triangular structures served as spatial positioning references to facilitate comparison of the probe fluorescence signals before and after the cleavage reaction. The results (Fig.S1) showed that, in the presence of target, the fluorescence signal in the molecular dam region markedly decreased, whereas the fluorescence signal remained stable in the no-target control. These results support that the immobilized tri-block probes on the chip surface can be degraded by target-activated CRISPR-Cas13a, providing direct surface evidence for Cas13a-mediated degradation of immobilized tri-block probes.

Regarding the reviewer's request for a quantitative correlation between molecular cleavage events and macroscopic drainage behavior, we agree that this point needed clearer explanation in the previous version. In conventional fluorescence CRISPR assays, the signal usually increases continuously with the number of cleaved reporter molecules. Therefore, a linear or quasi-linear relationship can often be established between cleavage amount and fluorescence intensity. Our chip, however, uses a different readout principle. The macroscopic drainage output is a threshold-triggered event rather than a continuously accumulated fluorescence signal.

Specifically, Cas13a-mediated probe degradation first weakens the local wetting barrier at the molecular dam interface. When this weakening is sufficient to disturb the force balance at the liquid–dam interface, the droplet that has been pre-positioned in a threshold-confined state can cross the drainage threshold and generate the "dam-break drainage" output. This can be understood

figuratively as the chip having been designed, through interfacial wettability and chip structure, to pre-construct a near-critical “reservoir–dam” state: the reservoir can store liquid several times its own volume and is maintained near the drainage threshold. Under this state, local weakening of the molecular dam interface is analogous to opening a small breach in a dam that is already close to its critical point, which immediately triggers a flood-like signal release without needing to destroy the entire dam. Under this mechanism, a limited number of molecular cleavage events can trigger a fluidic response far larger than their own scale. This mechanism explains why limited molecular events can be converted into a larger-scale macroscopic fluidic response without nucleic acid pre-amplification. Therefore, the amplification process in this work is essentially not a “linear accumulation of molecular counts,” but rather a “macroscopic physical transition triggered by crossing a critical threshold.” Thus, in this system, the relationship between molecular cleavage events and macroscopic drainage behavior is more appropriately described as a threshold-dependent relationship, rather than as a simple linear quantitative correlation.

To better support this interpretation, we have strengthened two lines of evidence in the revised manuscript. First, the fluorescence confocal experiment provides direct surface evidence that target-activated CRISPR-Cas13a can degrade the tri-block probes immobilized on the chip surface, supporting target-dependent local weakening of the molecular dam. Second, the structural optimization experiments (Fig.2) show that this local molecular weakening can be converted into macroscopic drainage only when the dam width, dam height and liquid volume together place the system within an appropriate threshold window. For example, 600 μL of liquid can be stably confined in region A with a composite dam height of 0.27 mm, whereas 700 μL causes spontaneous breakthrough. Similarly, when the dam is too narrow, the liquid is difficult to confine even without target; when the dam is too wide, drainage cannot be triggered even in the presence of target. These results support a threshold-type linkage from immobilized probe degradation to wetting-barrier weakening, and then to threshold crossing and macroscopic drainage.

We thank the reviewer again for raising this important point. This comment helped us realize that the distinction between a linear reporter-accumulation process and a threshold-triggered fluidic conversion was not sufficiently clear in the previous manuscript. In the revised manuscript, we have added fluorescence confocal evidence for Cas13a-mediated degradation of immobilized tri-block probes and reorganized the relevant mechanistic descriptions.

The corresponding modifications has been made as shown below

1.Page 3, SI

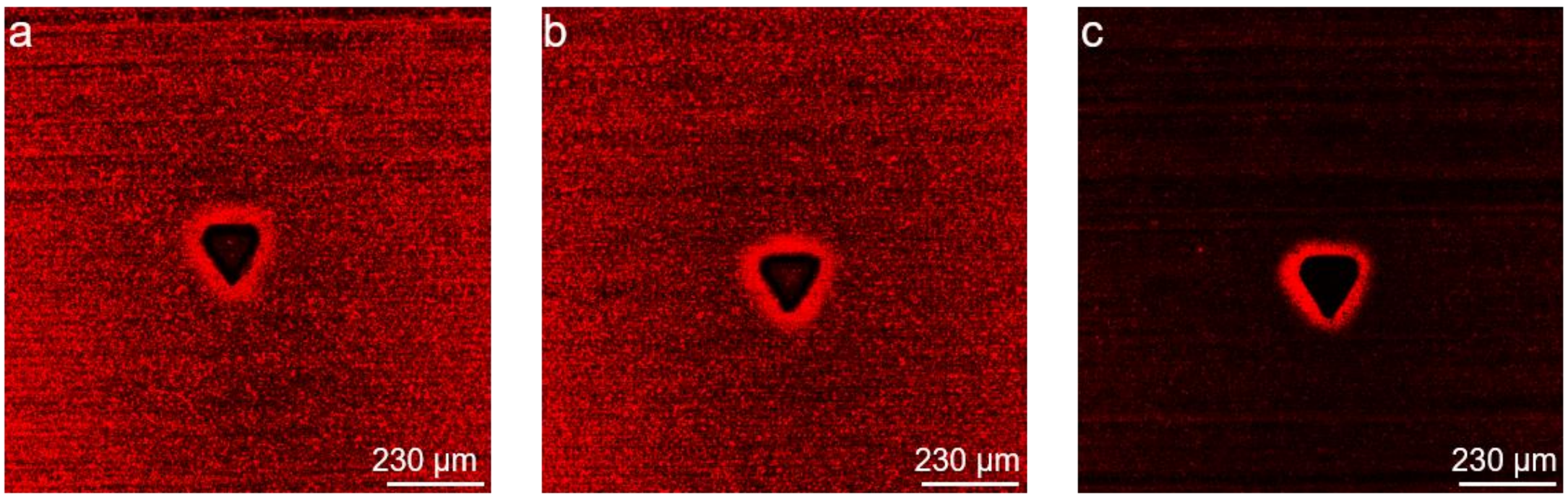

Supplementary Fig. 1. Cleavage of surface-immobilized tri-block probes by target-activated Cas13a. Cy3-labeled tri-block probes were immobilized on a substrate with triangular positioning references to enable confocal fluorescence imaging. **(a)** Initial fluorescence image. **(b)** Fluorescence image after incubation with the non-target control, showing stable fluorescence. **(c)** Fluorescence image after incubation with the target-activated Cas13a system, showing a marked fluorescence decrease. These results indicate effective cleavage of surface-immobilized tri-block probes by target-activated Cas13a. Note: The assay was performed using 80 nM Cas13a, 40 nM crRNA, 1 nM target RNA, and NEBuffer 4.

Was added

2.Page 4, lines 108-111

We employed the CRISPR-Cas13a system as a target-activated mediator to degrade the responsive spacer of the tri-block probe at this interface and trigger a hydrophobic-to-hydrophilic wettability transition.

Was replaced by

As validated in Fig. S1, the CRISPR-Cas13a system can function as a target-activated mediator to efficiently cleave the responsive spacer of surface-immobilized tri-block probes, thereby inducing a hydrophobic-to-hydrophilic wettability transition.

2.Page 18, lines 514-517

In this study, we established single-plex and duplex "dam-break drainage" event chips based on CRISPR-Cas13a mediated interfacial wettability switching and a visible macroscopic fluidic readout. The central advance of this work lies in creating a steady-state detection mode under fixed-boundary conditions, in which target recognition is directly converted into organized liquid-motion output without subsequent instrumentation or manual actuation. Compared with the previous tube-based implementation, the present chip architecture removes the operational uncertainty associated with manual flipping and enables stable signal triggering after one-step sample loading. In parallel, by establishing a practical nasal swab workflow based on room-temperature lysis and RNase inhibition, we further show that this sensing strategy can operate reliably in clinical sample matrices rather than only under idealized reaction conditions.

The significance of developed duplex module lies not merely in placing two targets on one chip, but in showing that parallel and distinguishable visual outputs can be organized under shared-system conditions. Achieving this required preserving the threshold state after the first triggering event and restoring local selectivity when both targets could activate Cas13a within the same reaction environment. The directional self-replenishment structure, together with the dam-anchored and self-releasing crRNA design, provides the structural basis for this organized duplex readout.

At the same time, the current study remains limited to qualitative visual detection, and multiplexing has been experimentally established only at the duplex level. Further expansion to higher-order multiplexing will require systematic investigation of signal independence, threshold maintenance and readout organization in more complex shared systems. Likewise, although the underlying wettability switching readout principle may, in principle, be extendable to other analyte classes through appropriate upstream recognition modules, such broader applicability has not been experimentally demonstrated here. In this sense, the methodology established in the present work provides a foundation for future exploration of higher-order multiplex visual biosensing based on interfacial wettability switching and macroscopic fluidic readout.

Was replaced by

In this study, we established single-plex and duplex "dam-break drainage" event chips based on CRISPR-Cas13a mediated interfacial wettability switching and a visible macroscopic fluidic readout. The central advance of this work lies in creating a steady-state detection mode under fixed-boundary conditions, in which target recognition is directly converted into organized liquid-motion output without subsequent instrumentation or manual actuation. Compared with the previous tube-based implementation, the present chip architecture removes the operational uncertainty associated with manual flipping and enables stable signal triggering after one-step sample loading. In parallel, by establishing a practical nasal swab workflow based on room-temperature lysis and RNase inhibition, we further show that this sensing strategy can operate reliably in clinical sample matrices rather than only under idealized reaction conditions.

Mechanistically, the rapid and highly sensitive performance of the chip arises from the coupling of CRISPR-Cas13a-mediated molecular amplification with chip-level threshold conversion. Cas13a collateral cleavage provides target-dependent molecular perturbations, while the chip architecture regulates the force balance at the liquid–dam interface and maintains the assay liquid in a threshold-confined state. Under this condition, a limited reduction in the local wetting barrier is sufficient to break the interfacial balance and nonlinearly trigger a macroscopic "dam-break drainage" transition , establishing a threshold-dependent relationship between molecular probe degradation and macroscopic drainage behavior. This coupled amplification process enables molecular recognition events to be read out beyond the sensitivity range achievable by the standalone CRISPR-Cas13a assay, without requiring nucleic acid pre-amplification or external instrumentation.

The significance of developed duplex module lies not merely in placing two targets on one chip, but in showing that parallel and distinguishable visual outputs can be organized under shared-system conditions. Achieving this required preserving the threshold state after the first triggering event and restoring local selectivity when both targets could activate Cas13a within the same reaction environment. The directional self-replenishment structure, together with the dam-anchored and self-releasing crRNA design, provides the structural basis for this organized duplex readout.

At the same time, the current study remains limited to qualitative visual detection, and multiplexing has been experimentally established only at the duplex level. Further expansion to higher-order multiplexing will require systematic investigation of signal independence, threshold maintenance and readout organization in more complex shared systems. Likewise, although the underlying wettability switching readout principle may, in principle, be extendable to other analyte classes through appropriate upstream recognition modules, such broader applicability has not been experimentally demonstrated here. In this sense, the methodology established in the present work provides a foundation for future exploration of higher-order multiplex visual biosensing based on interfacial wettability switching and macroscopic fluidic readout.

Thank you for your nice comments and your recognition of our work again! Your professional and careful suggestions significantly improved our manuscript. Wish you a happy life and successful career!

Reviewer #2 (Remarks to the Author): No more comments.

Response: Thank you for your recognition of our work. Wish you a happy life and successful career!

Reviewer #3 (Remarks to the Author): I co-reviewed this manuscript with one of the reviewers who provided the listed reports. This is part of the Nature Communications initiative to facilitate training in peer review and to provide appropriate recognition for Early Career Researchers who co-review manuscripts.

Response: Thank you for your recognition of our work. Wish you a happy life and successful career!
