# Supplementary material for: A CRISPR-based ‘dam-break drainage’ sensing architecture for rapid, affordable and ultrasensitive pathogen detection
Source: Nat Commun. 2026 Aug 22;17:11194. doi: 10.1038/s41467-026-77042-w (PMC13714288; doi:10.1038/s41467-026-77042-w)
Supplement: Supplementary file 3 [file 41467_2026_77042_MOESM1_ESM.pdf]

Supplementary Information for

A CRISPR-based 'dam-break drainage' sensing architecture for rapid, affordable and ultrasensitive pathogen detection

Kai Liu[1]†, Xiang Li[1]†, Meng Zhao[1], Zhuo Yang[1], Jingshuai Wen[1], Jinpeng Liu[1], Haoli Zhang[1], Yuecan Zhao[1], Yang Sun[1*], Zhengping Li[1*]

[1] Beijing Key Laboratory for Bioengineering and Sensing Technology, School of Chemistry and Biological Engineering, University of Science and Technology Beijing, 30 Xueyuan Road, Haidian District, Beijing, 100083, PR China.

†These authors contributed equally to this work.

*Corresponding authors: lzpbd@ustb.edu.cn (Z.L.); yangsunbio@ustb.edu.cn (Y.S.)

The file includes:

Supplementary Discussion
Supplementary Figs. 1 to 29
Supplementary Tables 1 to 11
Supplementary References

Supplementary Discussion

The clinical sample pretreatment protocol

We systematically investigated the RNase activity in lysates obtained from SARS-CoV-2 clinical samples subjected to different pretreatment methods. For throat swabs, four approaches were compared: room-temperature lysis (10 min), heat lysis (95°C, 10 min), room-temperature lysis with an RNase inhibitor (RRI) and heat lysis with RRI. Room-temperature lysis produced lysates with high RNase activity, leading to substantial RNA degradation. In contrast, heat lysis or room-temperature lysis with RRI only partially inhibited RNase activity; complete suppression was achieved only when both heat lysis and RRI were combined (Supplementary Fig. 10a). For nasal swabs, both room-temperature and heated lysis yielded lysates exhibiting high RNase activity. This activity was effectively eliminated by supplementing either lysate with RRI (Supplementary Fig. 11a). Given the reliance of our chip on CRISPR-Cas13a, we further examined whether the lysates with completely suppressed RNase activity would interfere with Cas13a activity. When these lysates were introduced into the CRISPR-Cas13a solution containing RRI, the RNase activity of Cas13a was not inhibited (Supplementary Figs. 10b and 11b). Considering that SARS-CoV-2 nasal swabs were selected as the clinical testing samples, we next evaluated the effect of different lysis methods on RNA extraction efficiency. The lysate obtained from the same SARS-CoV-2–positive nasal swab sample was divided into two portions, which were lysed either at room temperature for 10 min or by heating at 95°C for 10 min. The resulting lysates were serially diluted (1, 1/2, 1/4, 1/6) and analyzed using an RT–PCR system containing RRI. At the same dilution levels, the room-temperature lysates consistently yielded lower Ct values than the heat-lysed ones, indicating higher RNA extraction efficiency (Supplementary Fig. 12). Finally, we assessed whether varying concentrations of RRI would affect Cas13a RNase activity. As shown in Supplementary Fig. 13, adding different volumes of RRI (0.02, 0.1, 0.2, 0.4, or 0.6 μL per 10 μL reaction) did not inhibit Cas13a RNase activity.

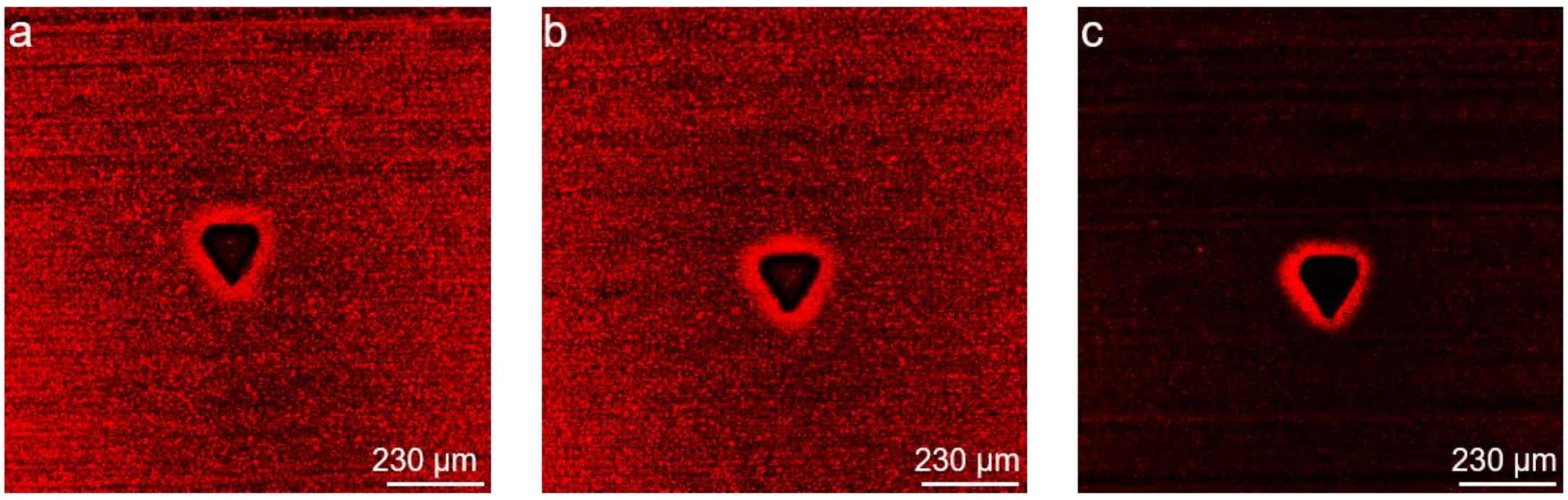

Supplementary Fig. 1. Cleavage of surface-immobilized tri-block probes by target-activated Cas13a. Cy3-labeled tri-block probes were immobilized on a substrate with triangular positioning references to enable confocal fluorescence imaging. **(a)** Initial fluorescence image. **(b)** Fluorescence image after incubation with the non-target control, showing stable fluorescence. **(c)** Fluorescence image after incubation with the target-activated Cas13a system, showing a marked fluorescence decrease. These results indicate effective cleavage of surface-immobilized tri-block probes by target-activated Cas13a. Note: The assay was performed using 80 nM Cas13a, 40 nM crRNA, 1 nM target RNA, and NEBuffer 4.

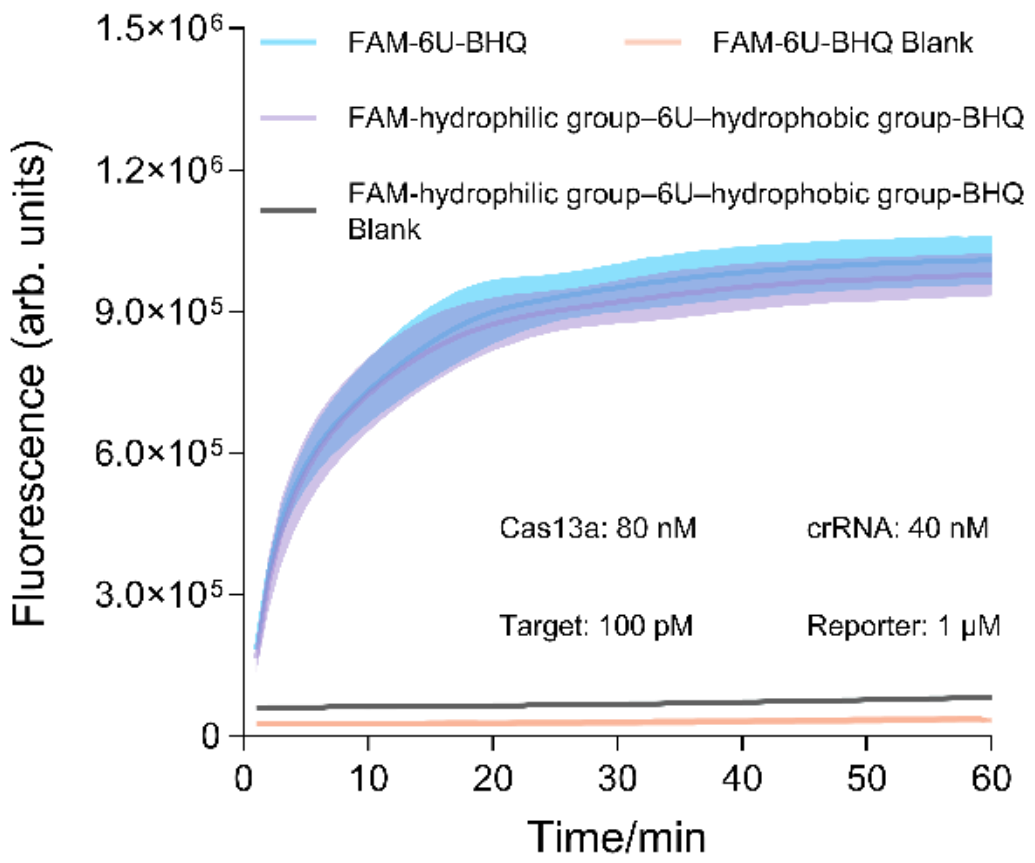

Supplementary Fig. 2. Collateral kinetics comparison of CRISPR-Cas13a to FAM-$(PEG_6)_2$-6U-$(CH_2)_{12}$-BHQ and FAM–6U–BHQ reporters. Solid lines represent the mean fluorescence intensity and the shaded areas indicate the min-max range (n=3 independent experiments). Source data are provided as a Source Data file.

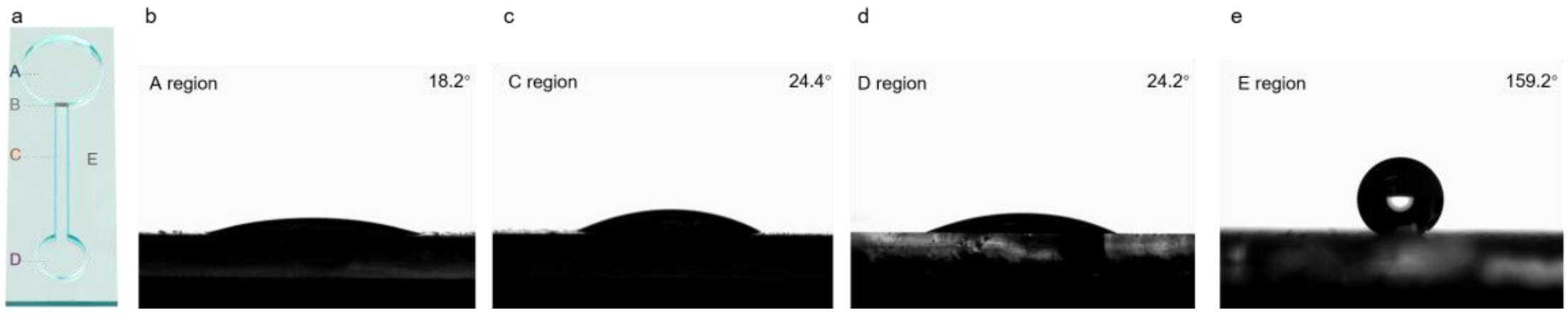

Supplementary Fig. 3. Wettability characterization of the designed functional regions. (a) Chip structure. **(b)** The contact angle of A region (the sample reservoir) is 18.2°. **(c)** The contact angle of C region (the channel) is 24.4°. **(d)** The contact angle of D region (the collection zone) is 24.2°. **(e)** The contact angle of E region (the exterior surface) is 159.2°.

a

Reagents	Time	Concentration
APTES	60 min	60%
Glutaraldehyde	60 min	50%
Tri-block probe	60min	?
B region width	90 µm	

b

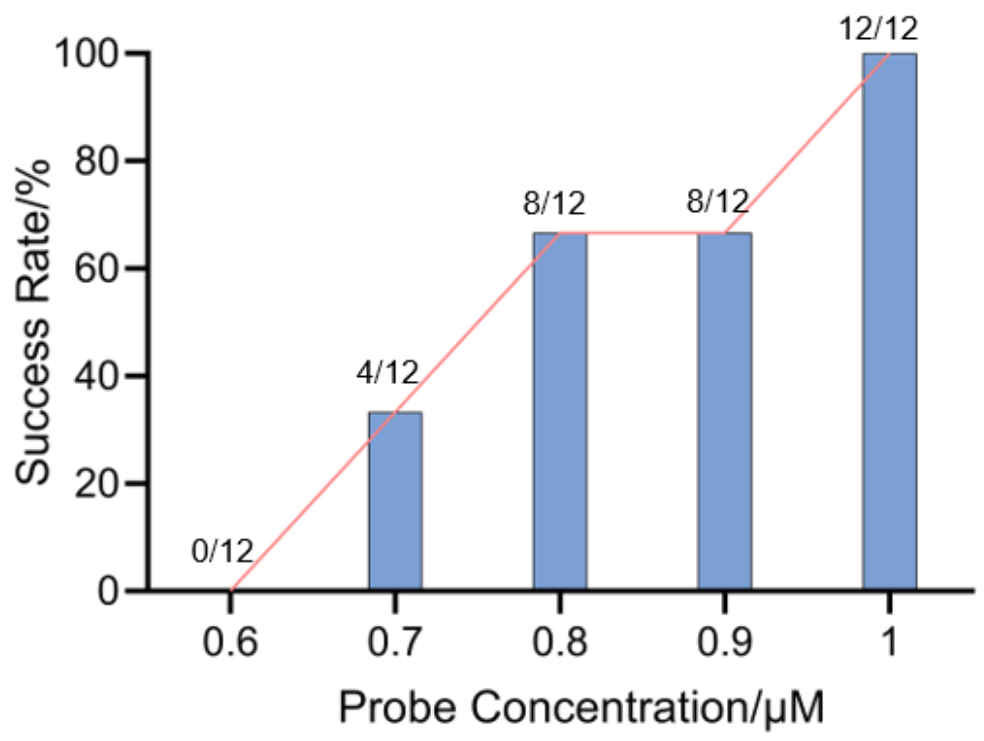

Supplementary Fig. 4. Optimization of chip surface modification conditions. (a) Summary table of the chip surface-modification parameters, including the reagents, reaction time and corresponding working concentrations. **(b)** Success rate of stable confinement of 100 µL water in the sample reservoir as a function of probe concentration during surface modification. Bars represent the percentage of chips achieving stable confinement (*n*=12 independent chips). Source data are provided as a Source Data file.

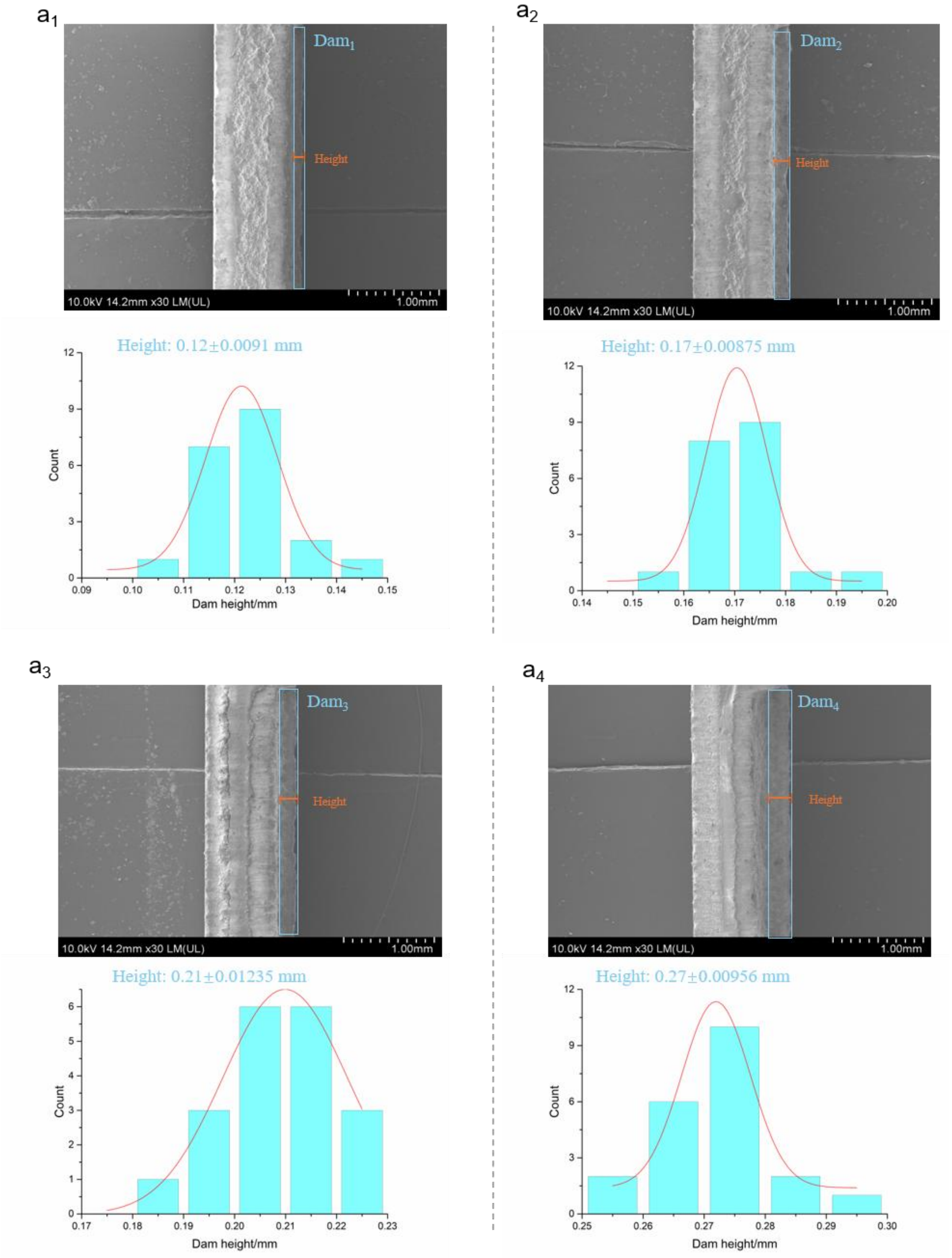

Supplementary Fig. 5. Scanning electron microscopy characterization of physical dam height. Four physical dams with a fixed width of 0.09 mm were fabricated at different heights by adjusting the laser etching power: Dam_1 **(a_1)**, Dam_2 **(a_2)**, Dam_3 **(a_3)** and Dam_4 **(a_4)**. The mean heights were 0.12 ± 0.0091 mm (Dam_1), 0.17 ± 0.00875 mm (Dam_2), 0.21 ± 0.01235 mm (Dam_3) and 0.27 ± 0.00956 mm (Dam_4), respectively (mean ± s.d.; n=20 measurements taken at distinct positions on each dam). Histograms represent the height distributions, and solid curves indicate the Gaussian fits. Source data are provided as a Source Data file.

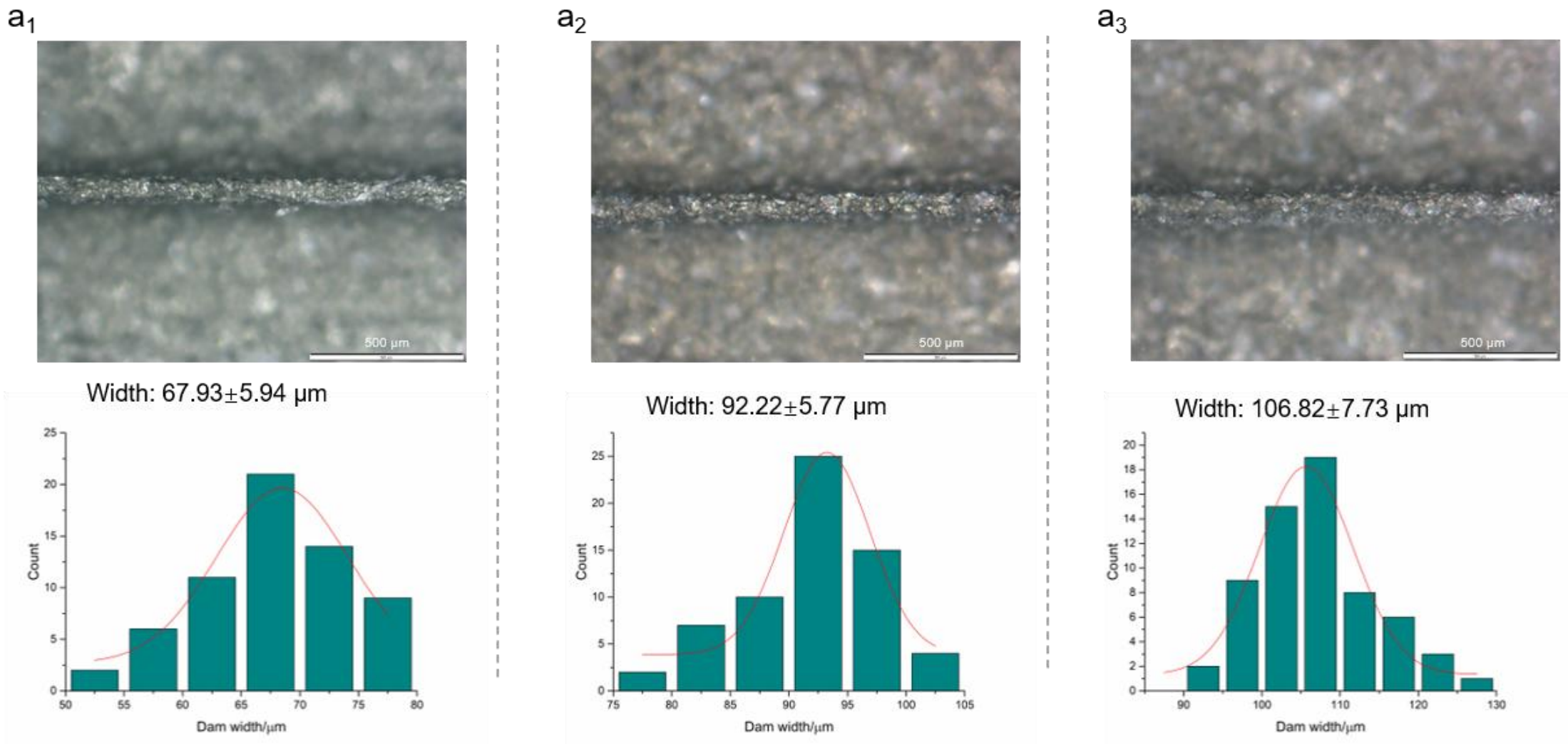

Supplementary Fig. 6. Metallurgical microscopy characterization of physical dam width. Three ranges of physical dam widths were fabricated at a fixed height of 0.27 mm **(a_1-a_3)**. The mean widths were 67.93±5.94 µm **(a_1)**, 92.22±5.77 µm **(a_2)** and 106.82±7.73 µm **(a_3)** respectively (mean ± s.d.; n=63 measurements taken at distinct positions on each dam). Histograms show the width distributions, and solid curves show the Gaussian fits. Source data are provided as a Source Data file.

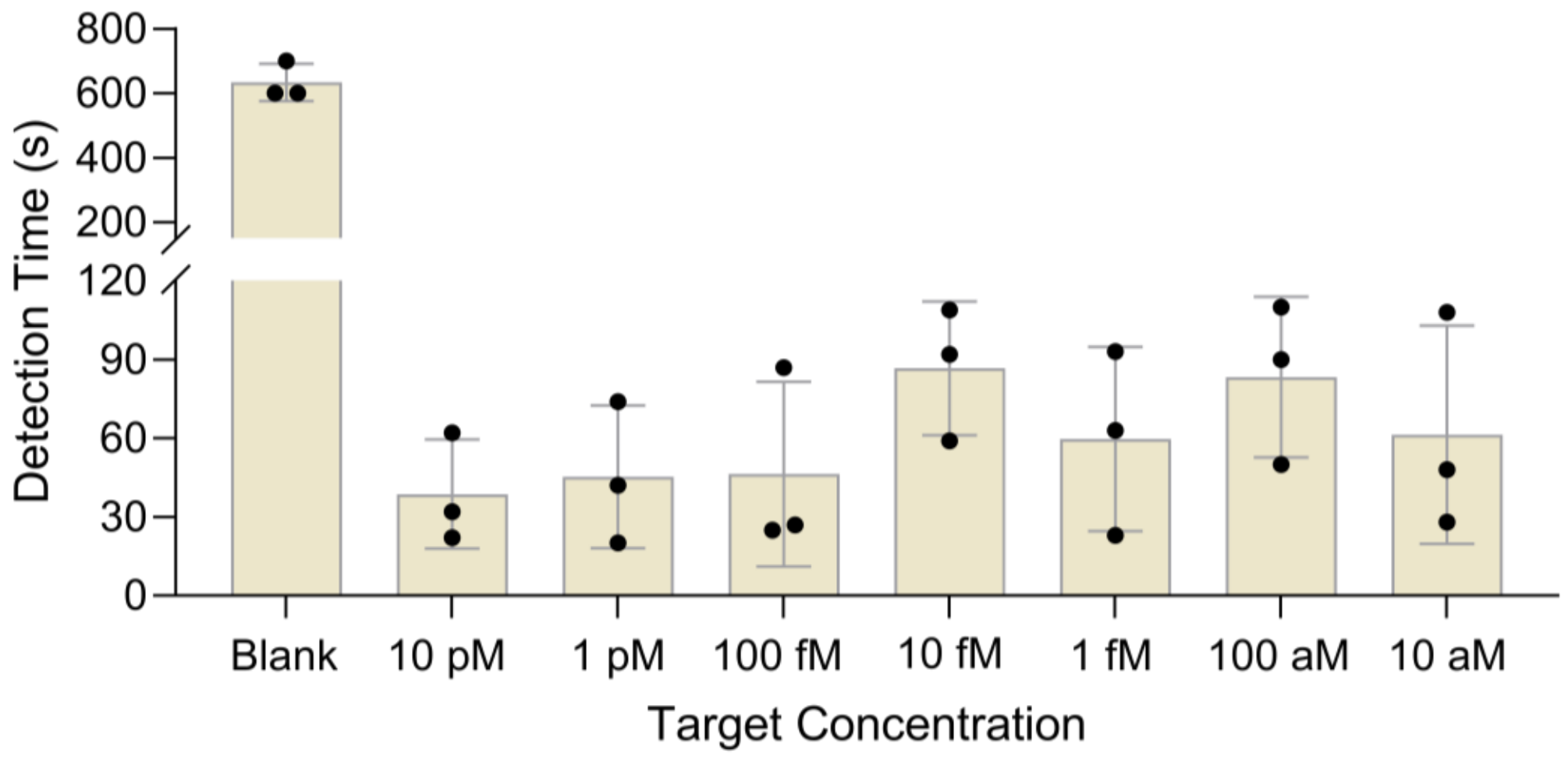

Supplementary Fig. 7. Statistical analysis of readout times for the single-plex chip in detection of the SARS-CoV-2 *N* gene across concentrations from 10 aM to 10 pM. Data are presented as mean ± s.d. (*n*=3 independent chips). All tested positive concentrations triggered visible "dam-break drainage" event within 2 min rapid decision time, whereas blank samples remained confined throughout the 10 min observation window. Source data are provided as a Source Data file.

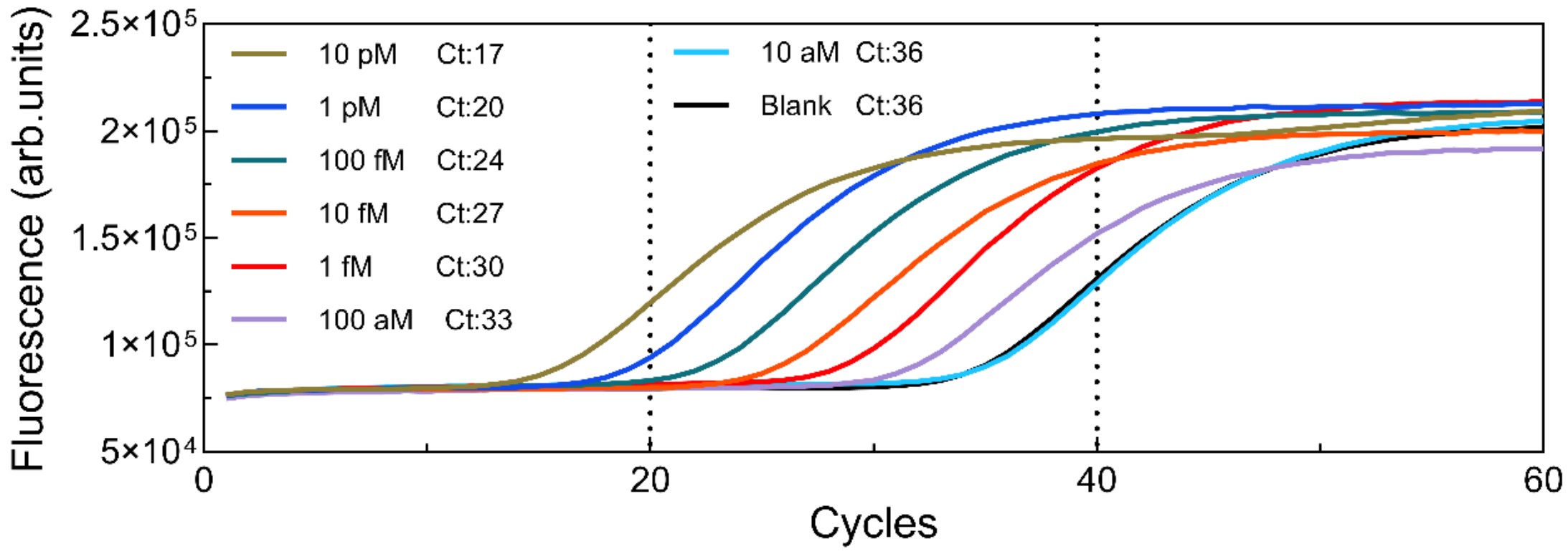

Supplementary Fig. 8. RT-PCR results of SARS-CoV-2 *N* gene in the concentration range of 10 aM-10 pM. The limit of detection is 100 aM. Source data are provided as a Source Data file.

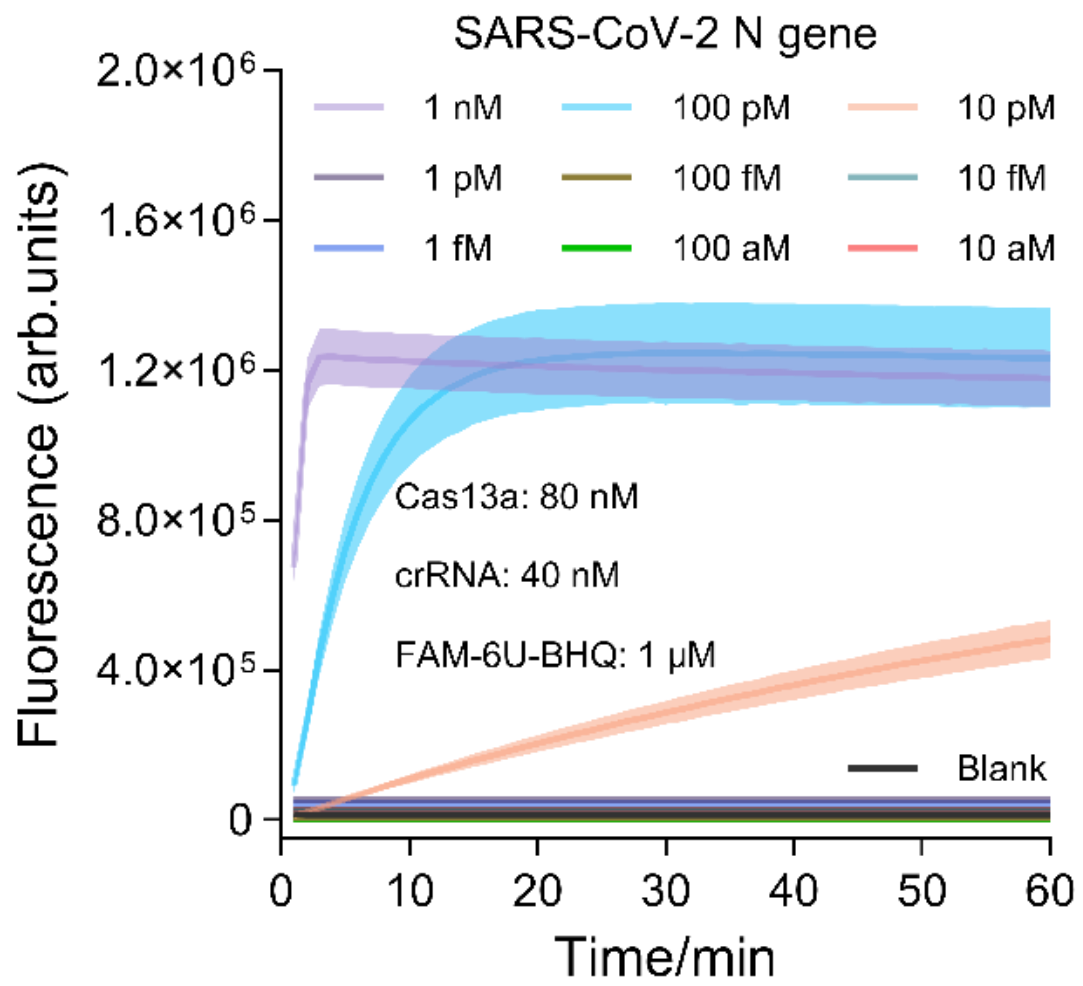

Supplementary Fig. 9. Sensitivity of the standalone CRISPR-Cas13a system for SARS-CoV-2 *N* gene detection. Solid lines represent the mean fluorescence intensity and the shaded areas indicate the min-max range (n=3 independent experiments). For SARS-CoV-2 *N* gene concentrations ranging from 10 aM to 1 pM, the fluorescence signal traces remained close to background and were comparable to the Blank control. Source data are provided as a Source Data file.

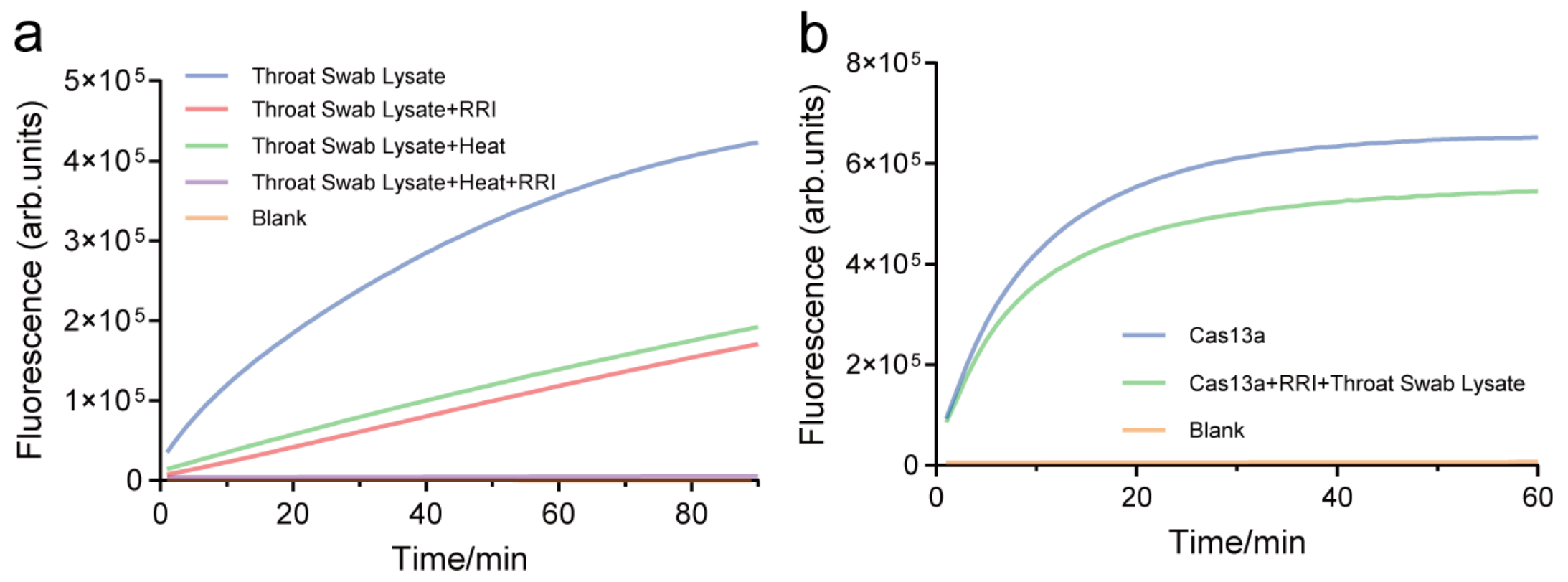

Supplementary Fig. 10. Evaluation of RNase suppression by different throat-swab pretreatments and compatibility of the resulting lysates with Cas13a. (a) RNase activity in throat-swab lysates following different pretreatment methods: (i) room-temperature lysis, (ii) heat lysis (95°C, 10 min), (iii) room-temperature lysis + RNase inhibitor (RRI) and (iv) heat lysis + RRI. **(b)** Comparison of Cas13a RNase activity between the CRISPR-Cas13a system alone and the system containing throat-swab lysates with RRI. Source data are provided as a Source Data file.

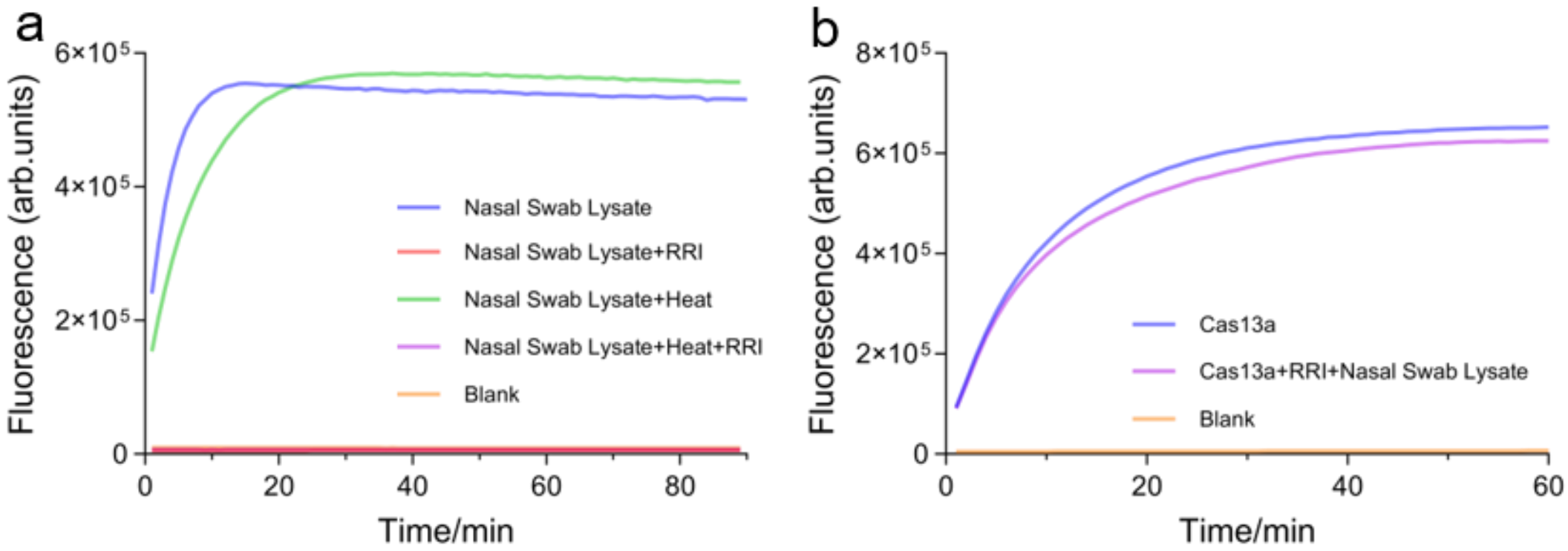

Supplementary Fig. 11. RNase suppression by different nasal-swab pretreatments and compatibility of the resulting lysates with Cas13a. (a) RNase activity in nasal-swab lysates following different pretreatment methods: (i) room-temperature lysis, (ii) heat lysis (95℃, 10 min), (iii) room-temperature lysis + RNase inhibitor (RRI) and (iv) heat lysis + RRI. **(b)** Comparison of Cas13a RNase activity between the CRISPR-Cas13a system alone and the system containing nasal-swab lysates with RRI. Source data are provided as a Source Data file.

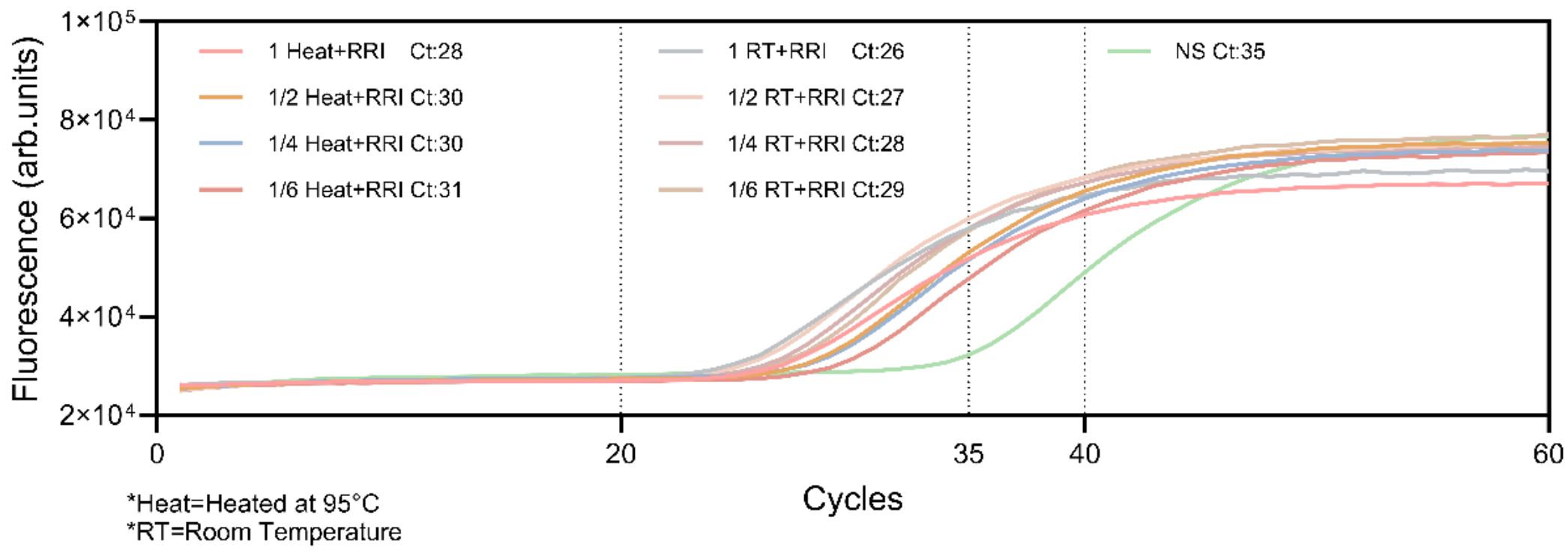

Supplementary Fig. 12. Comparison of RNA target extraction efficiency from SARS-CoV-2 nasal swabs by heat lysis or room-temperature lysis. Source data are provided as a Source Data file.

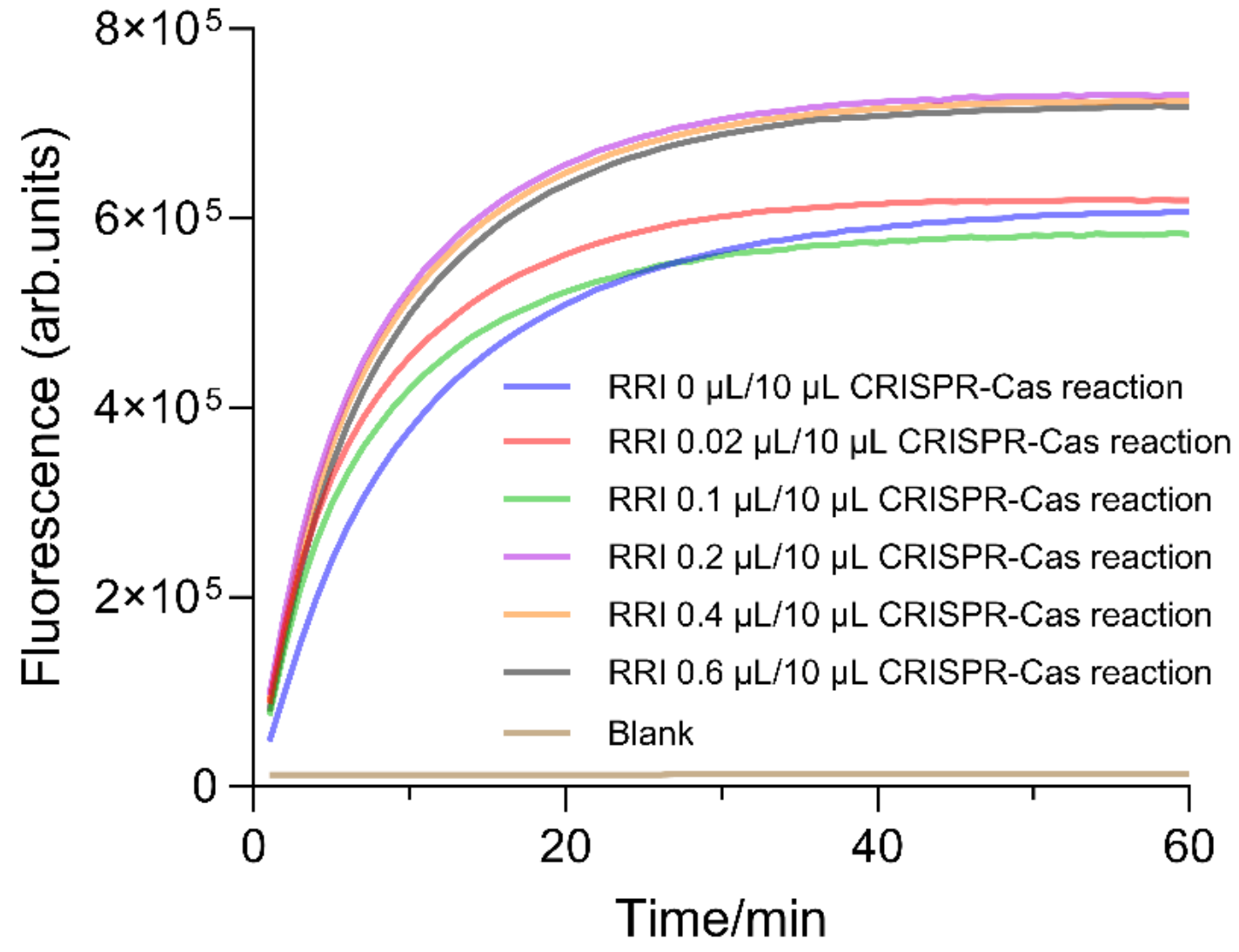

Supplementary Fig. 13. Comparison of Cas13a RNase activity in the CRISPR-Cas13a system with varying amounts of added RRI. Source data are provided as a Source Data file.

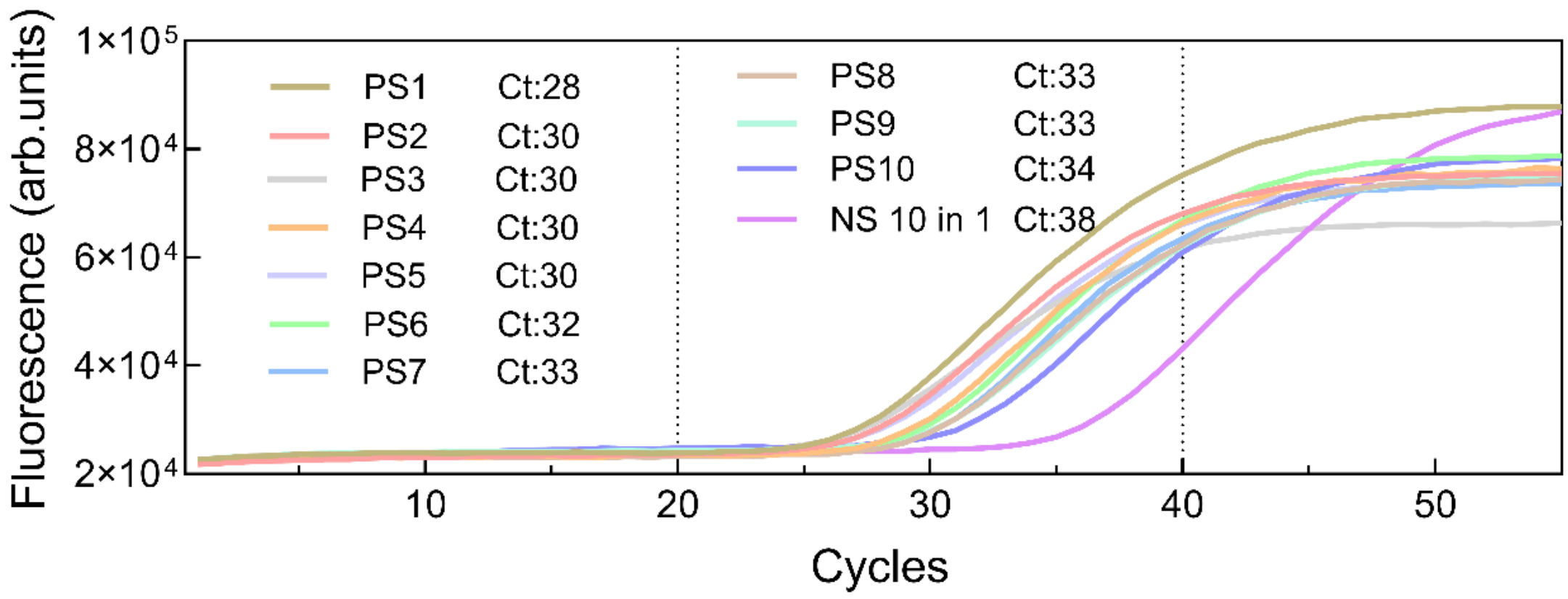

Supplementary Fig. 14. RT-PCR results of SARS-CoV-2 nasal swabs.

Notes：PSx indicates the index of each positive sample, while NS 10 in 1 refers to nucleic acids pooled from ten negative samples. The limit of detection is defined as a Ct value of 35. Source data are provided as a Source Data file.

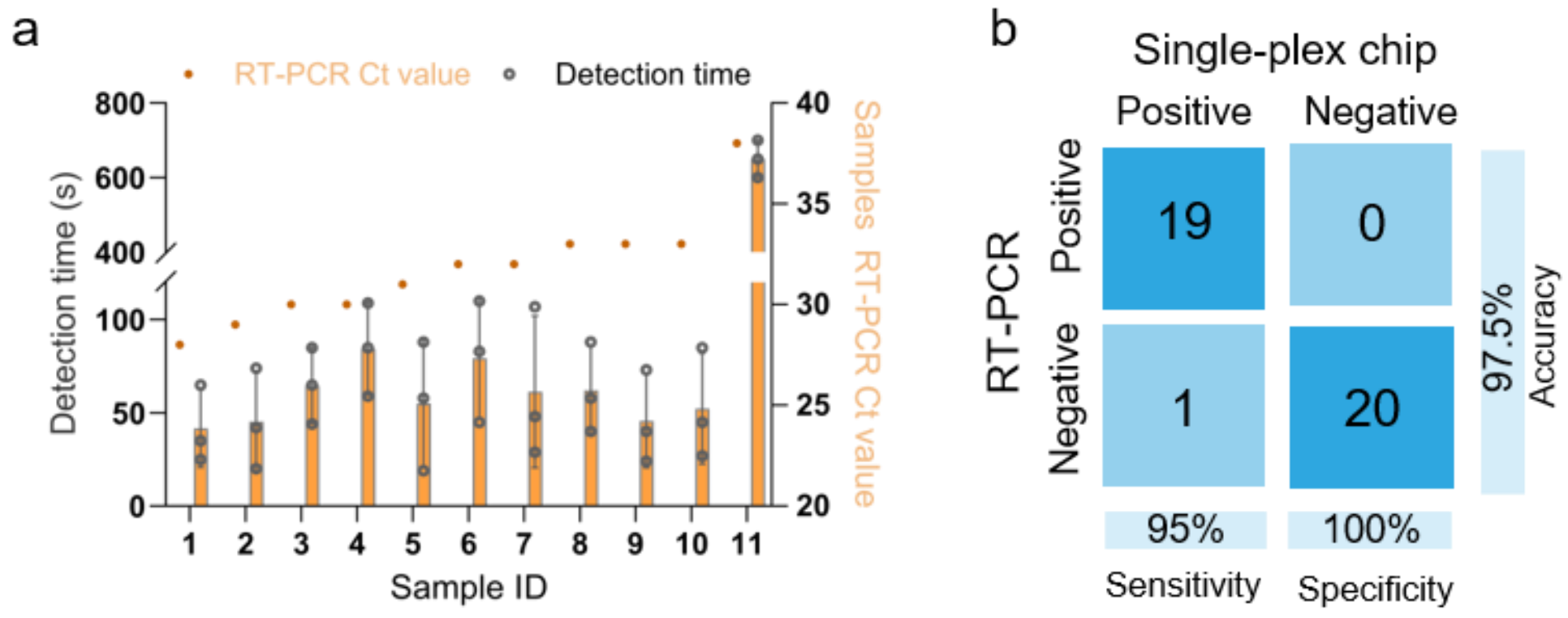

Supplementary Fig. 15. Performance characterizations of single-plex chips. (a) Detection-time analysis of the single-plex chip for clinical nasal-swab samples with RT-PCR Ct values from 28 to 33. Each orange dot indicates the RT-PCR Ct value of an individual sample. Grey dots represent individual chip-detection time, data are presented as mean ± s.d. (n=3 independent chips). All positive samples triggered visible dam-break drainage event within 2 min rapid decision time, whereas the negative sample remained confined in the sample reservoir throughout the 10 min observation window. (Note: Samples 1–10 denote the order of positive samples, and Sample 11 denotes the 10 in 1 negative sample.). **(b)** Confusion matrix analysis of sensitivity and specificity compared with the single-plex chip detection results, showing 95% sensitivity, 100% specificity and 97.5% accuracy.

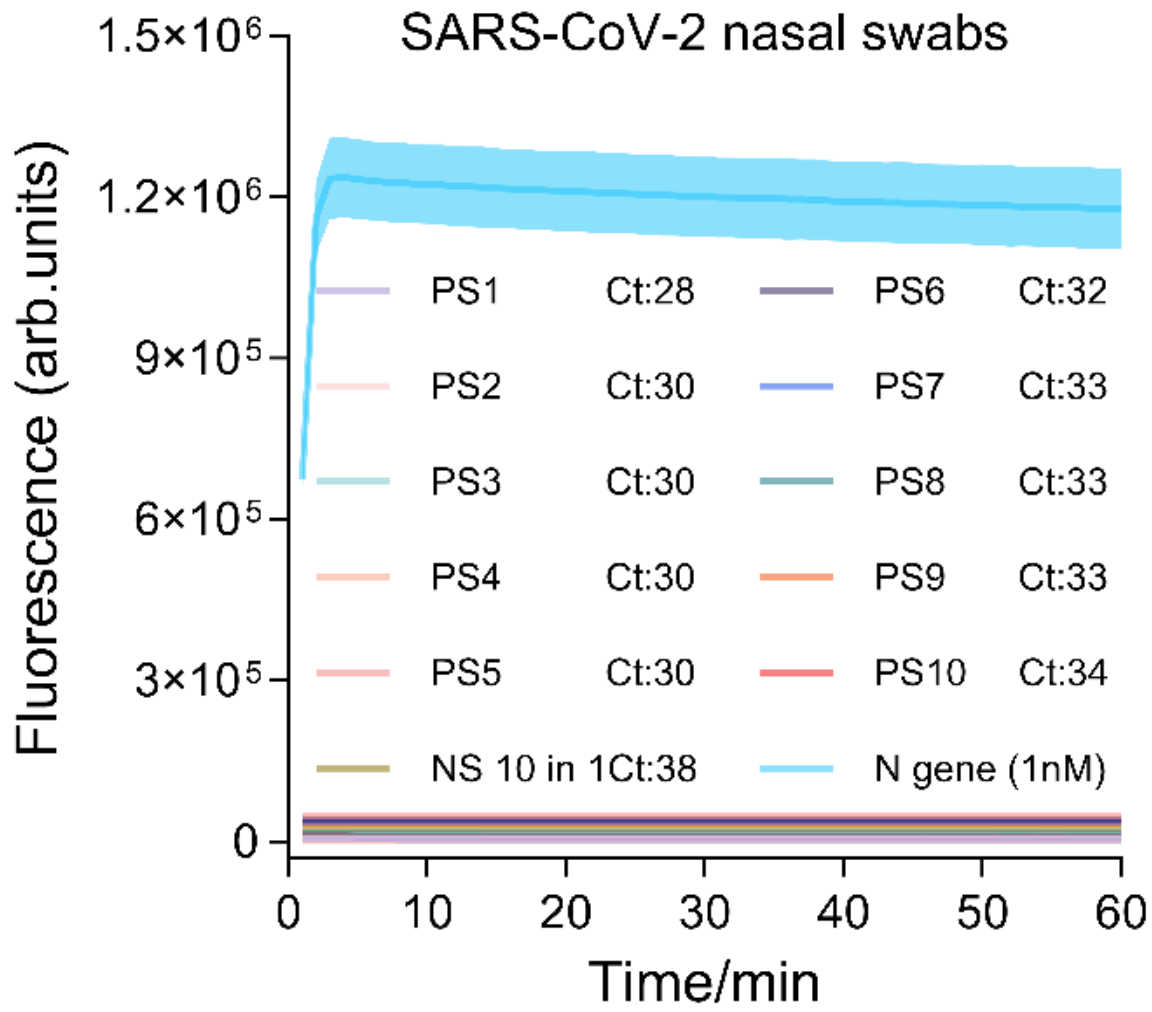

Supplementary Fig. 16. Sensitivity of the standalone CRISPR-Cas13a assay for ten SARS-CoV-2 RT–PCR-positive nasal-swab samples (Ct = 28–34) and one 10-in-1 pooled negative sample (Ct = 38). Solid lines represent the mean fluorescence intensity and the shaded areas indicate the min-max range (*n*=3 independent experiments). 1 nM SARS-CoV-2 N gene was used as the control group. Source data are provided as a Source Data file.

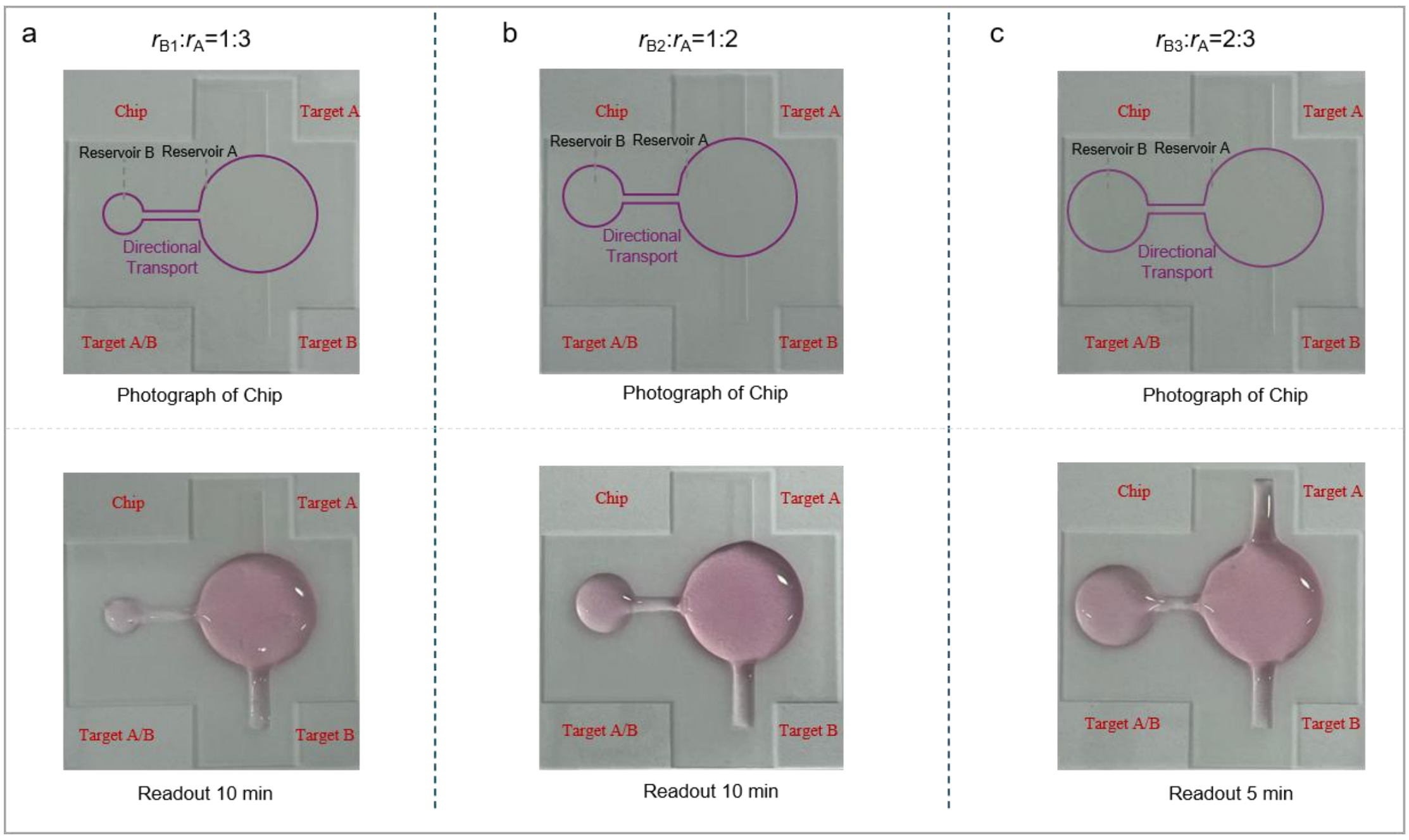

Supplementary Fig. 17. Self-replenishing performance of the duplex chip under different size ratios between reservoir B (r_B) and reservoir A (r_A). (a) When the ratio of r_B:r_A was 1:3, the duplex chip equipped with the self-replenishment system detected only one target nucleic acid even after 10 minutes. **(b)** When the ratio was 1:2, the chip likewise only detected one target after 10 minutes. **(c)** In contrast, at a ratio of 2:3, the duplex chip successfully completed simultaneous detection of both nucleic acid targets within 5 min. Source data are provided as a Source Data file.

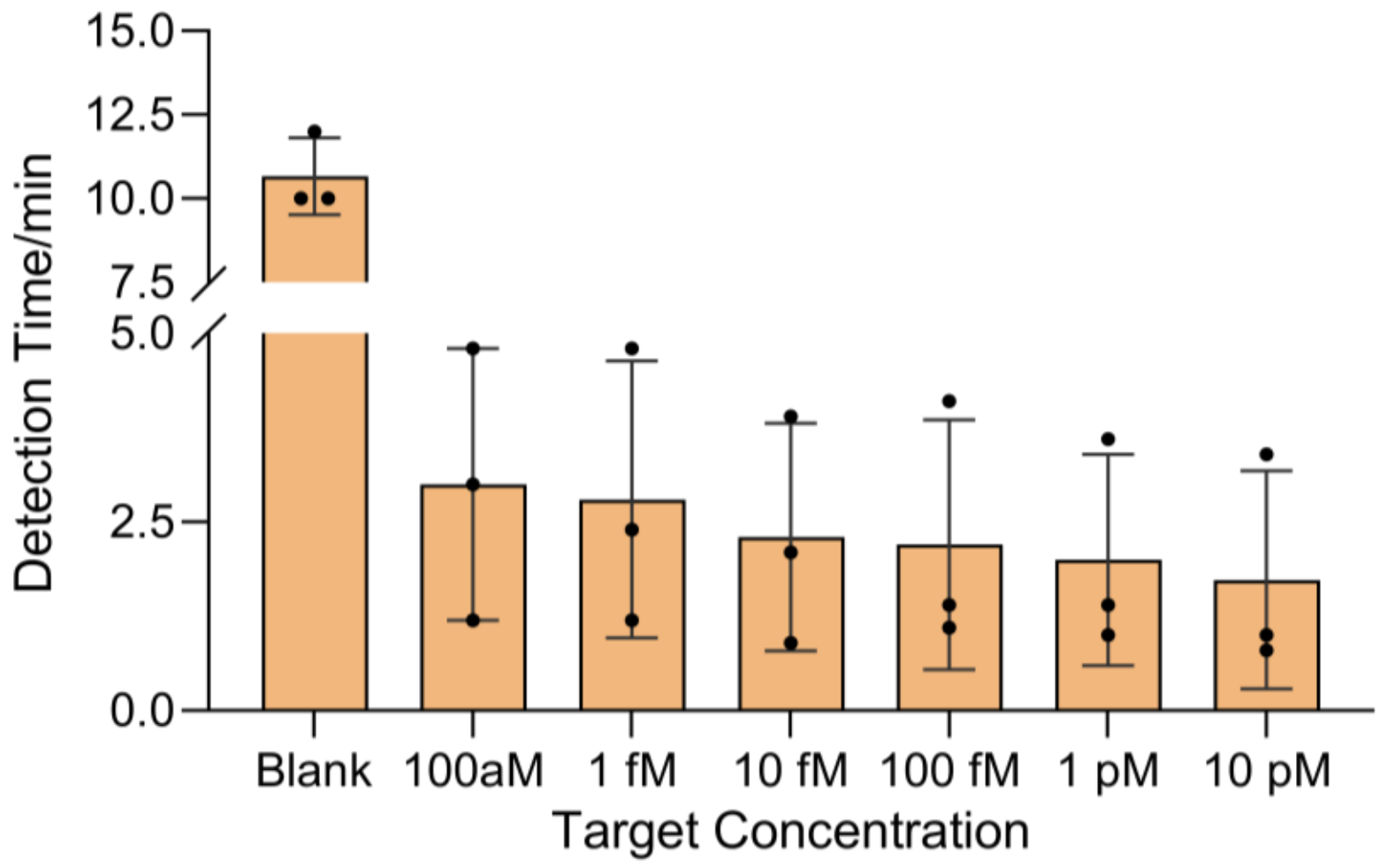

Supplementary Fig. 18. Statistics of detection time for the duplex chip in simultaneously analyzing the IAV *PB2* and IBV *PB1* genes. Detection time of the duplex chip for concurrent analysis of the IAV *PB2* and IBV *PB1* genes over concentrations ranging from 100 aM to 10 pM. Data are presented as mean ± s.d. (*n*=3 independent chips). Visible "dam-break drainage" event was consistently observed for all tested positive concentrations within 5 min rapid decision time, while blank samples remained confined throughout the 10 min observation window. Source data are provided as a Source Data file.

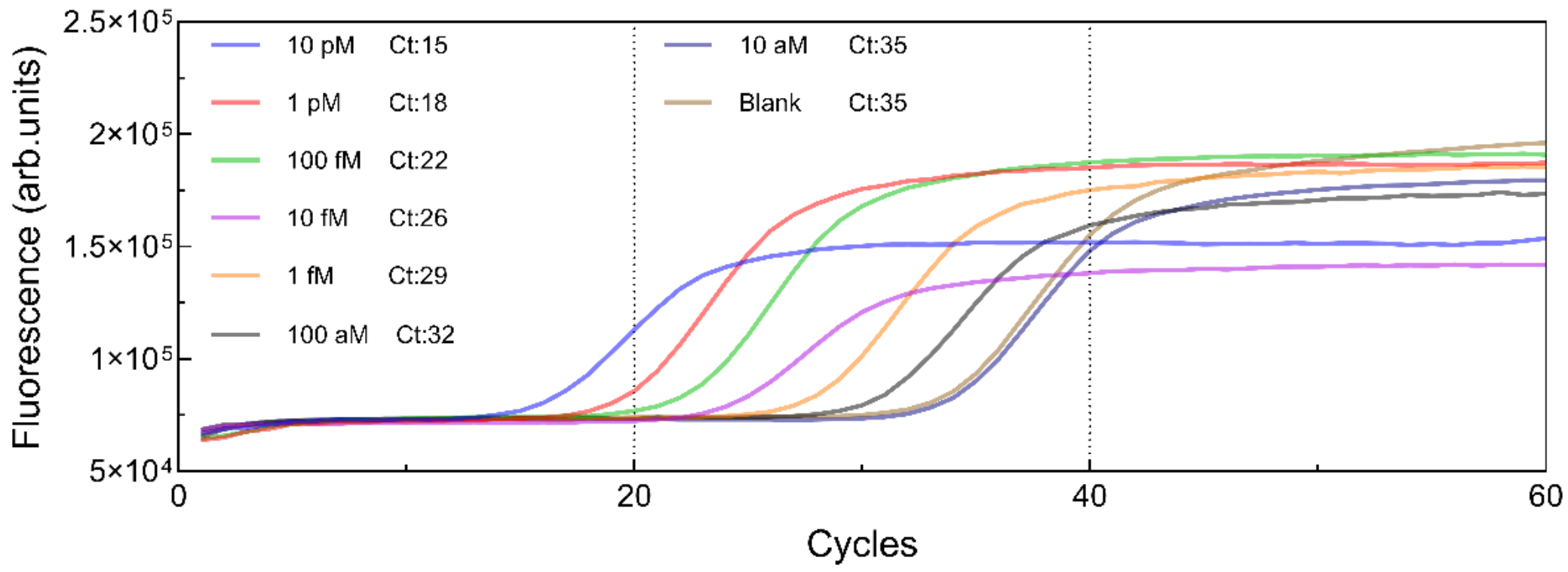

Supplementary Fig. 19. RT-PCR results of Influenza A virus *PB2* gene in the concentration range from 100 aM to 10 pM. Source data are provided as a Source Data file.

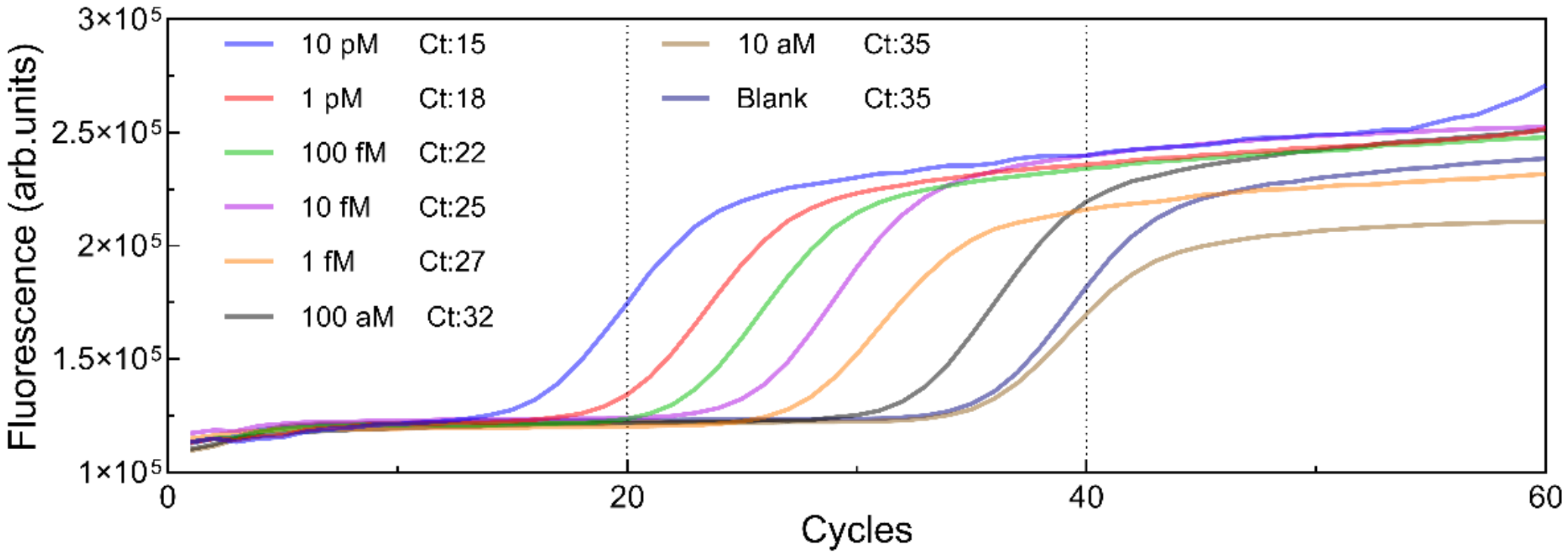

Supplementary Fig. 20. RT-PCR results of Influenza B virus *PB1* gene in the concentration range from 100 aM to 10 pM. Source data are provided as a Source Data file.

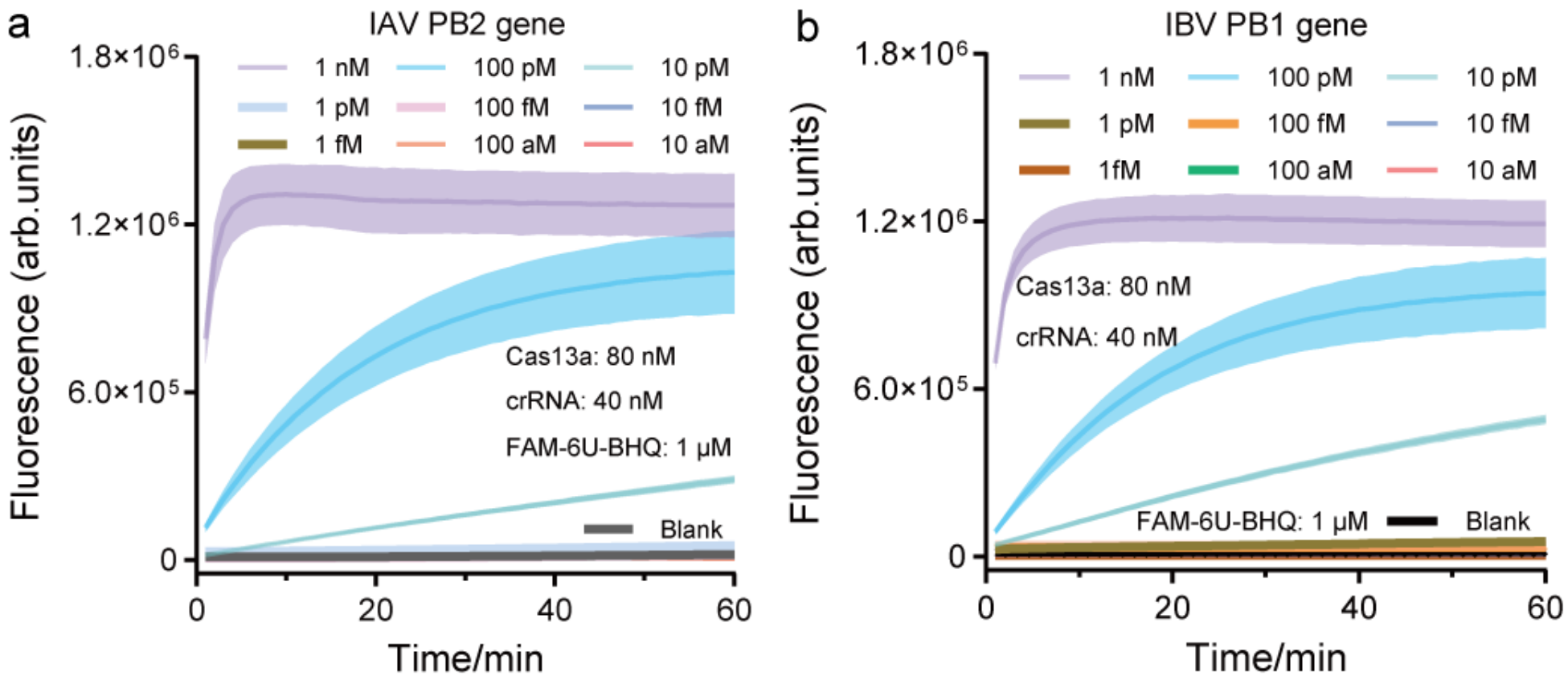

Supplementary Fig. 21. Sensitivity of the pure CRISPR–Cas13a system in detecting the IAV *PB2* gene (a), IBV *PB1* gene (b). Solid lines represent the mean fluorescence intensity and the shaded areas indicate the min-max range (*n*=3 independent experiments). In the CRISPR–Cas13a systems containing IAV-PB2 or IBV-PB1 RNA targets at concentrations ranging from 10 aM to 1 pM, real-time fluorescence signals remained close to the background level and were indistinguishable from the Blank control. Source data are provided as a Source Data file.

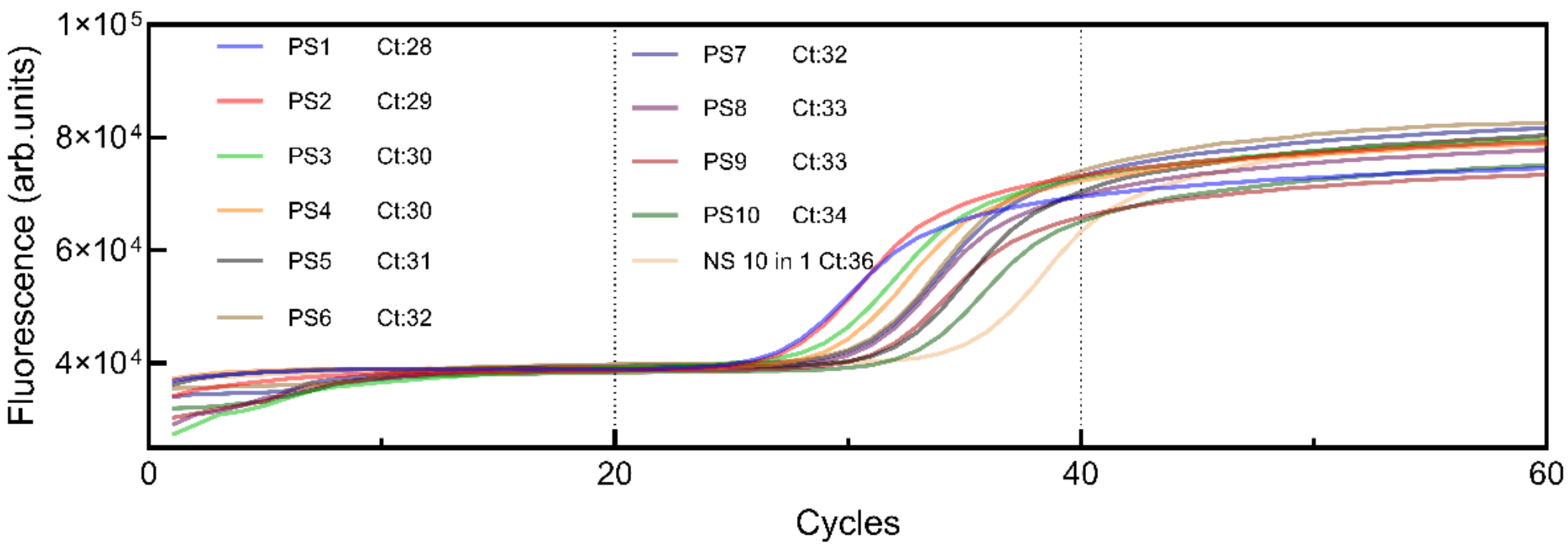

Supplementary Fig. 22. RT-PCR results of Influenza A virus nasal swabs. Source data are provided as a Source Data file.

Note: PSx represents the index value of each positive sample, and NS represents negative sample. The detection limit is defined as a Ct value of 35.

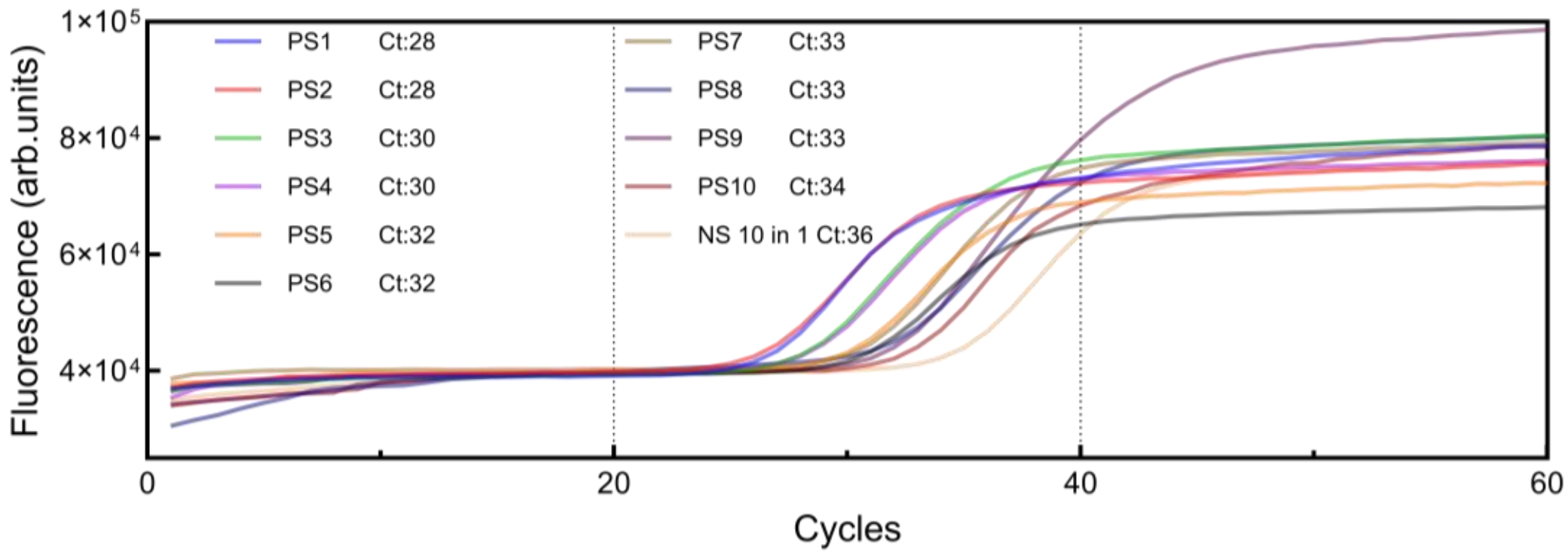

Supplementary Fig. 23. RT-PCR results of clinical samples of Influenza B virus nasal swabs. Source data are provided as a Source Data file.

Note: PSx denotes the numbering of individual positive samples, and NS refers to the negative sample. The detection limit is set at a Ct value of 35.

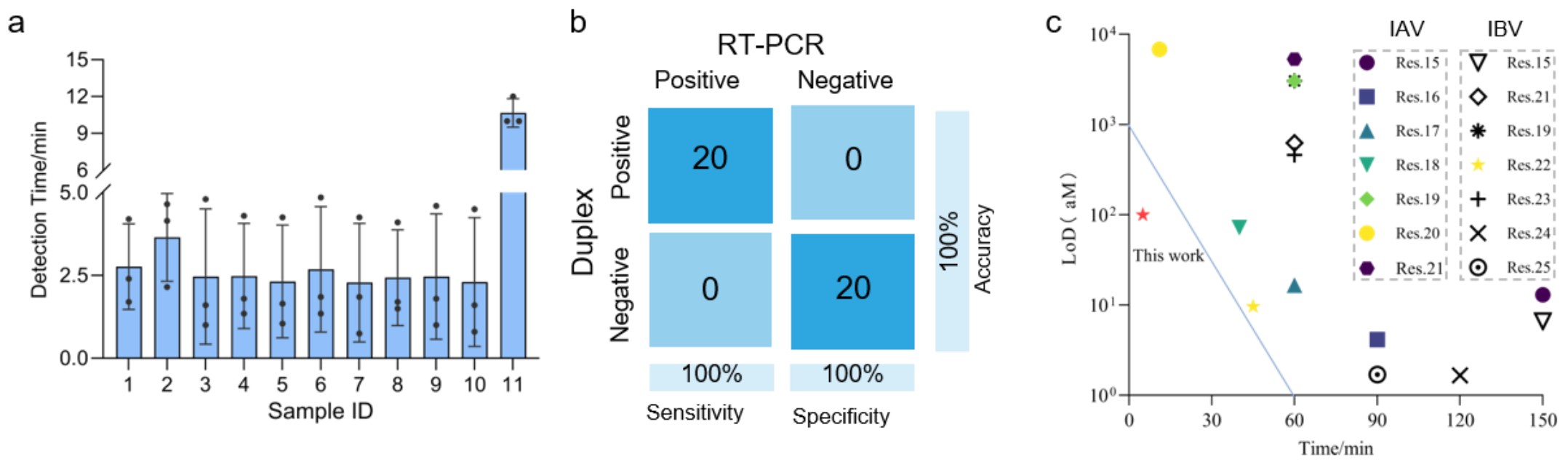

Supplementary Fig. 24. Performance characterization of duplex chips. (a) Statistical analysis of detection times for the duplex chip in simultaneous detection of IAV and IBV clinical samples. Black dots represent individual chip-detection time, data are presented as mean ± s.d. (n=3 independent chips). All positive samples reproducibly triggered visible "dam-break drainage" event within 5 min rapid decision time, whereas the negative sample remained confined in the sample reservoir throughout the 10 min observation window. (Note: Samples 1-10 represents ten groups of randomly mixed IAV RT-PCR-positive (Ct=28-34) and IBV RT-PCR-positive samples (Ct=28-34), Sample 11 represents the 10 in 1 negative sample). **(b)** Confusion matrix analysis relative to clinical RT-PCR results, demonstrating 100% sensitivity, specificity and accuracy. **(c)** Comparison of the developed chip with previously reported technologies. Source data are provided as a Source Data file.

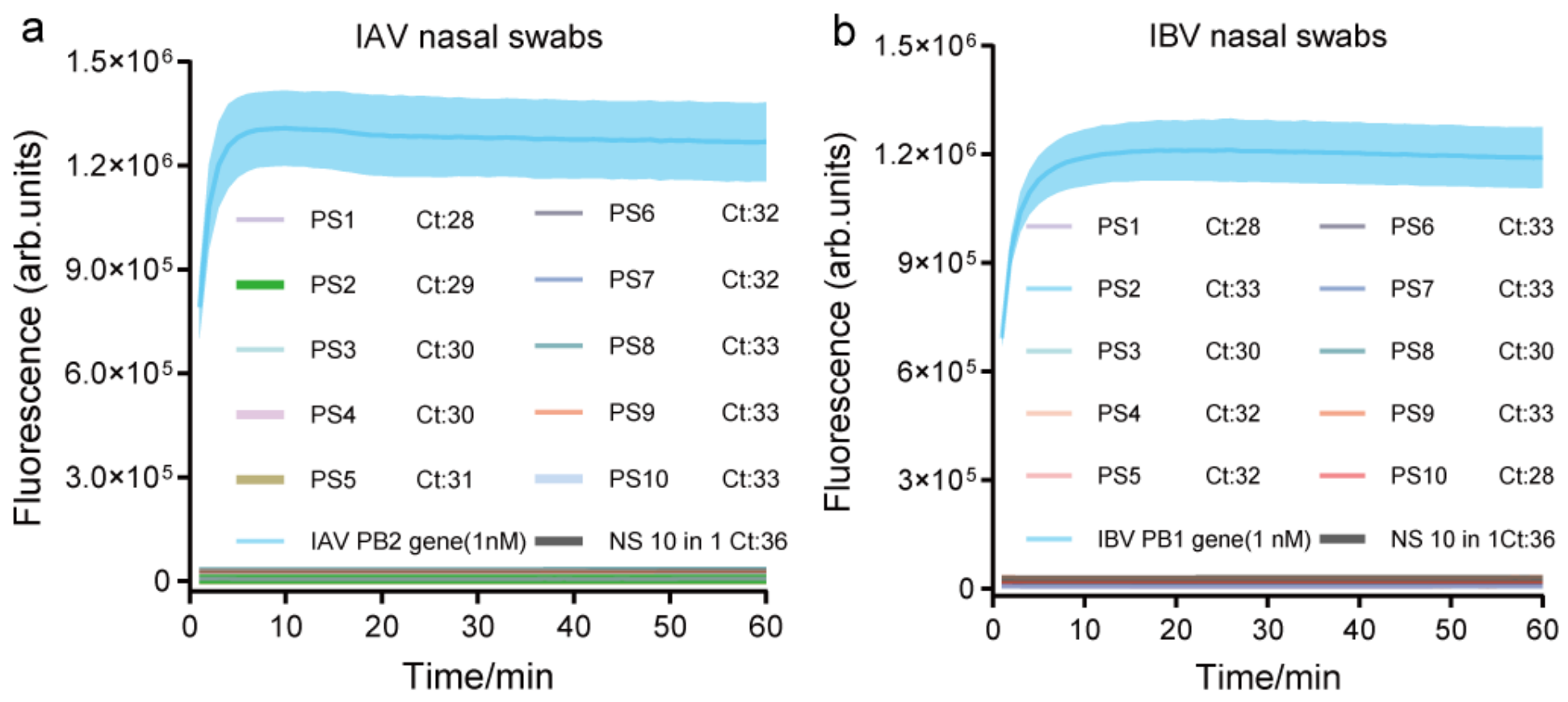

Supplementary Fig. 25. Sensitivity of the standalone CRISPR-Cas13a system in detecting IAV nasal swabs and IBV nasal swabs. Real-time fluorescence responses of ten IAV-positive (**a**) and ten IBV-positive (**b**) nasal swabs with Ct values ranging from 28 to 34, together with the corresponding negative 10 in 1 nasal swabs with Ct values of 36. 1 nM IAV *PB2* and IBV *PB1* RNA targets were served as control groups for the respective assays. Solid lines represent the mean fluorescence intensity and the shaded areas indicate the min-max range (*n*=3 independent experiments). Source data are provided as a Source Data file.

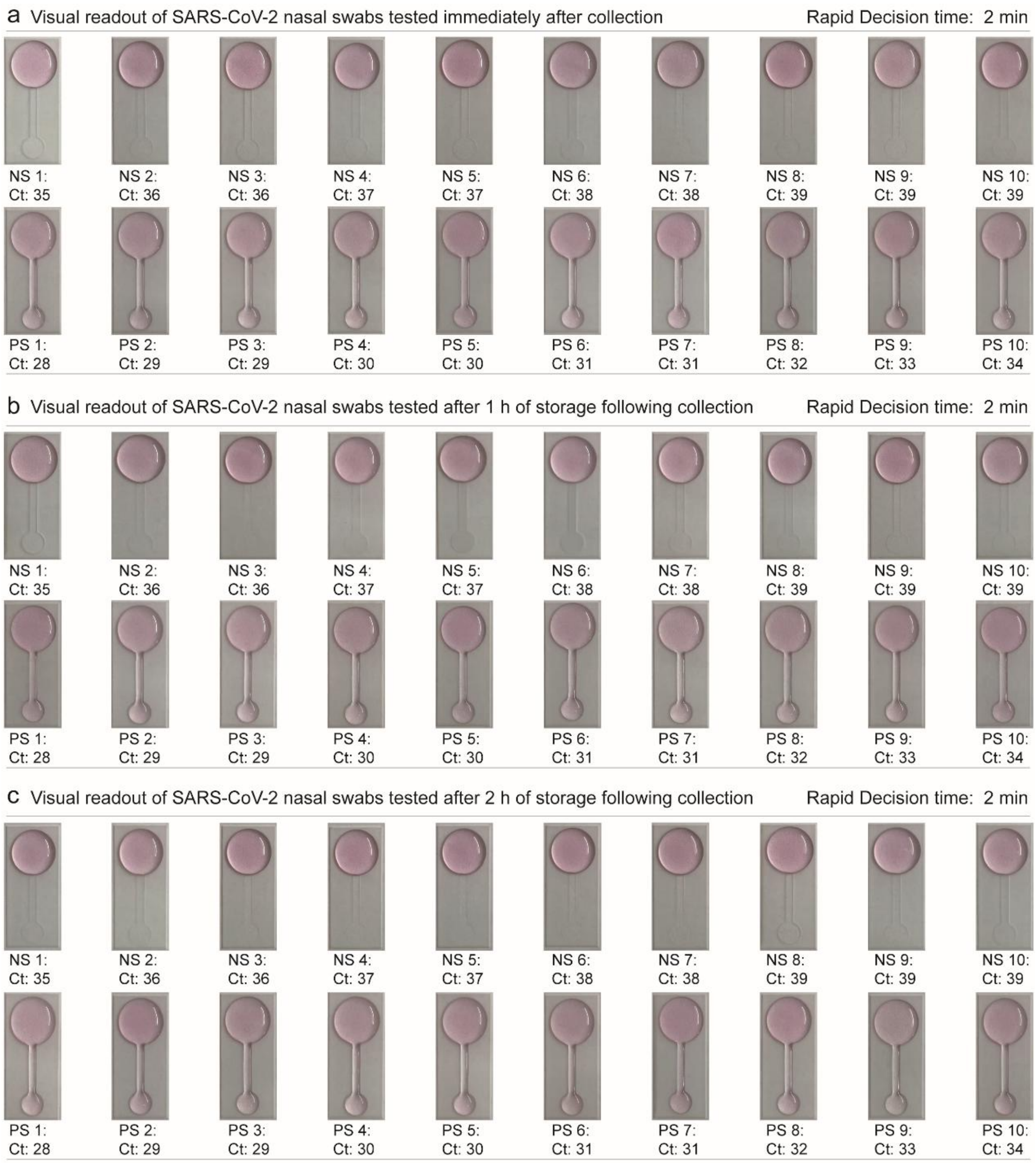

Supplementary Fig. 26. Detection results of the single-plex chip for SARS-CoV-2 nasal swabs after different storage durations. Visual readout immediately after collection (**a**), after 1 h storage (**b**) and after 2 h storage (**c**). NS denotes SARS-CoV-2-negative nasal swab samples (Ct=35–39), and PS denotes SARS-CoV-2-positive nasal swab samples (Ct=28-34). The corresponding RT–PCR Ct values are shown below each chip image.

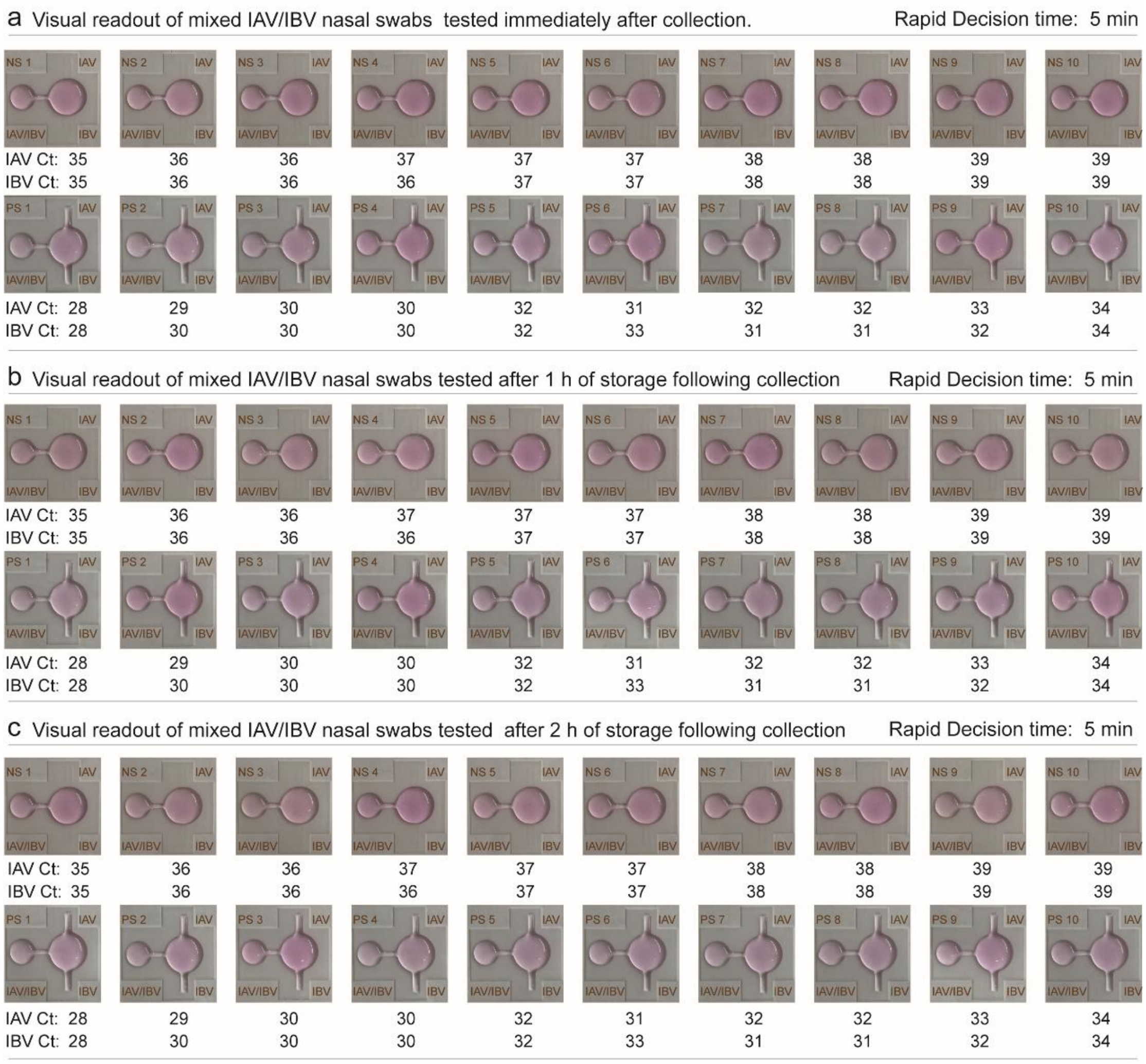

Supplementary Fig. 27. Detection results of mixed IAV and IBV nasal swabs after different storage durations. Visual readout of mixed IAV/IBV nasal swabs tested immediately after collection (**a**), after 1 h storage (**b**), after 2 h storage (**c**). NS indicates mixed nasal swabs classified as negative for both IAV (Ct=35-39) and IBV (35-39), whereas PS indicates pooled analysis of ten IAV-positive (Ct=28-34) and ten IBV-positive (Ct=28-34) nasal swabs. The RT-PCR Ct values corresponding to IAV and IBV nasal swabs are indicated below each chip image.

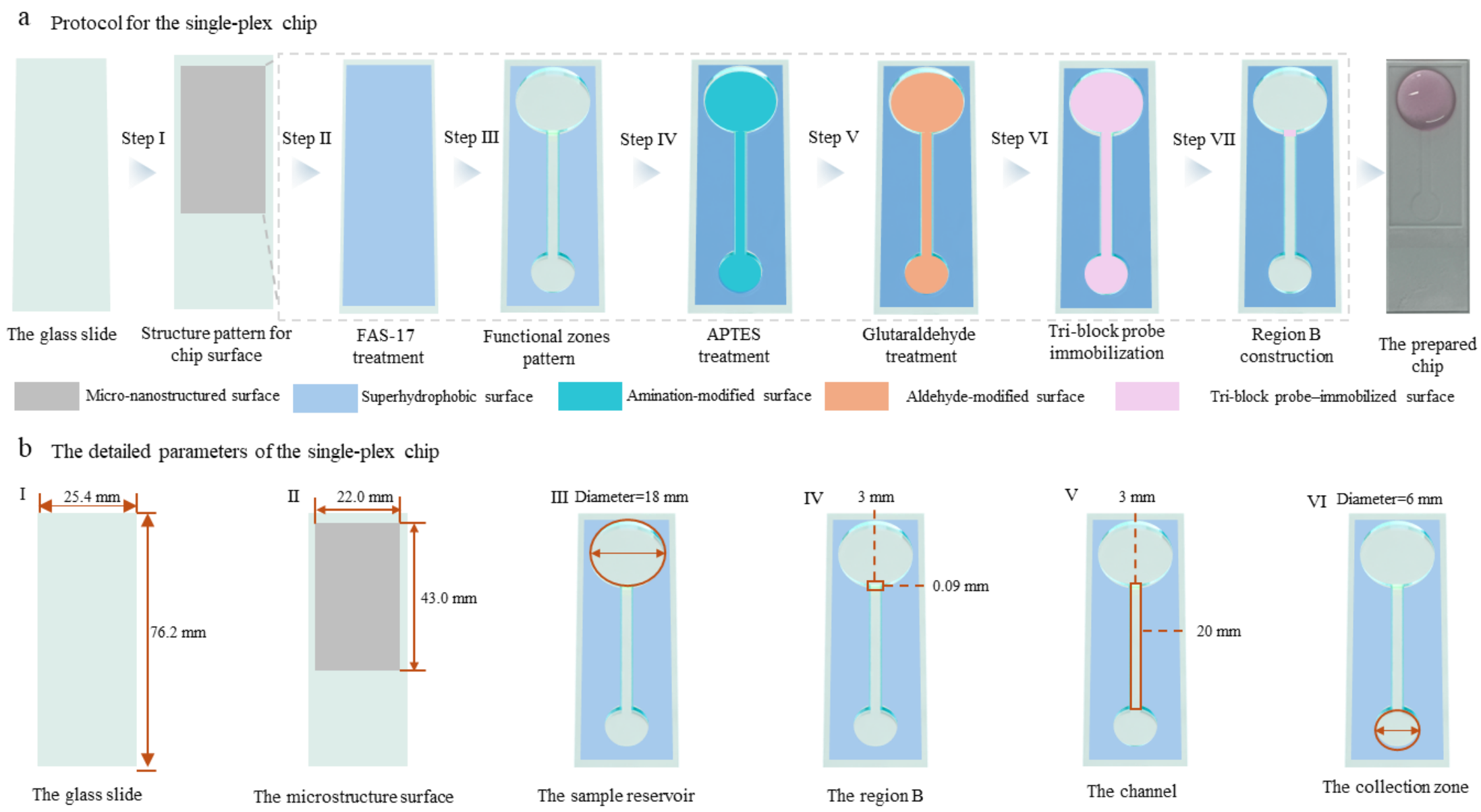

Supplementary Fig. 28. Fabrication workflow and structural dimensions of the single-plex chip. (a) Protocol for the single-plex chip. **(b)** The detailed parameters of the single-plex chip.

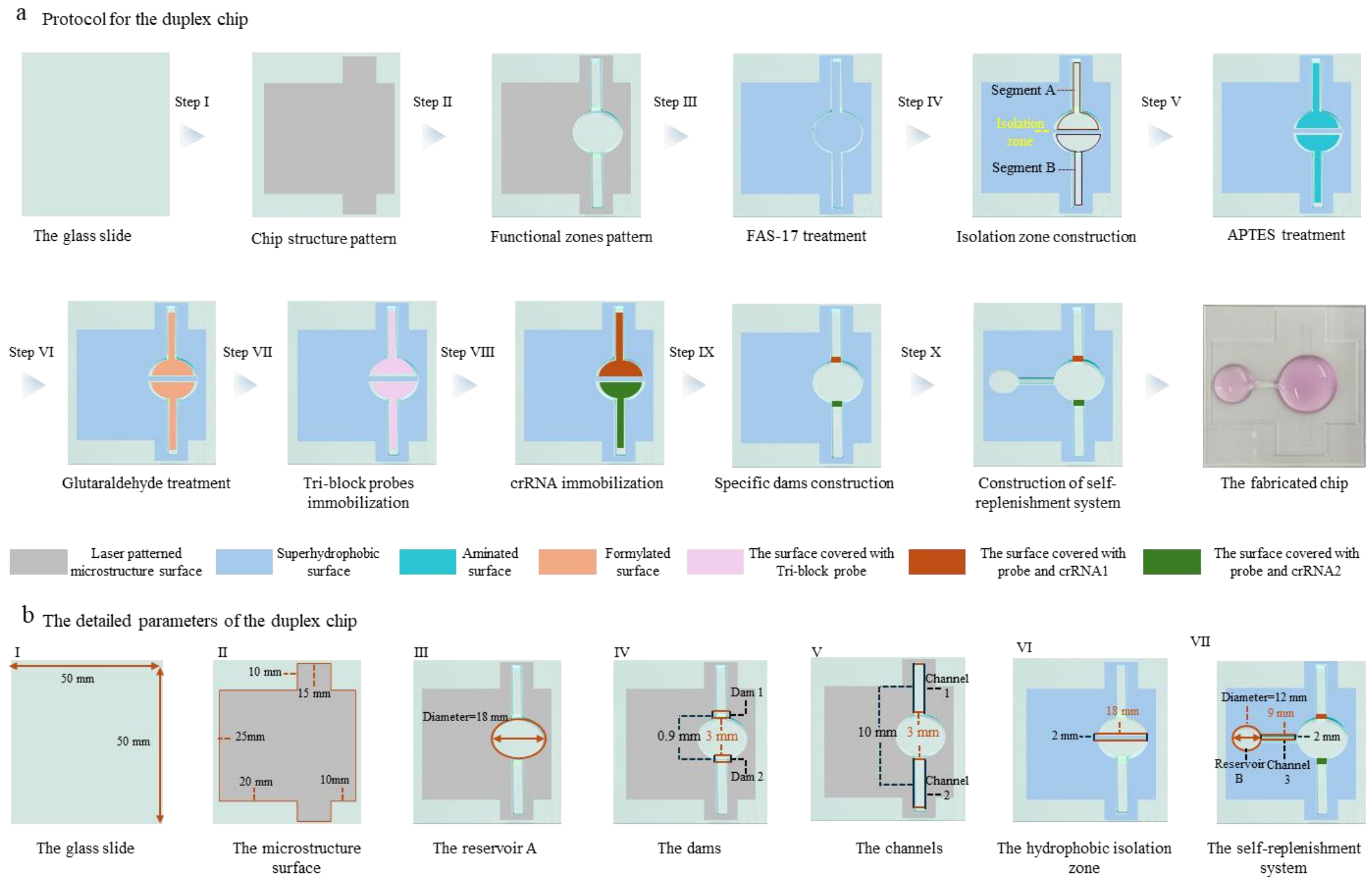

Supplementary Fig. 29. Fabrication procedure and geometric specifications of the duplex chip. **(a)** Protocol for the duplex chip. **(b)** The detailed parameters of the duplex chip.

Supplementary Table 1. The sequences of tri-block probes used in this work.

Name		Sequence (5'-3')
**Tri-block probes**	Tri-block probe 1 for Supplementary Fig. 1	NH_2-$(PEG_6)_2$-UUUUUU-$(CH_2)_{12}$-Cy3
	Tri-block probe 2 for Supplementary Fig. 2	FAM-$(PEG_6)_2$-UUUUUU-$(CH_2)_{12}$-BHQ
	Tri-block probe 3 for Chips	NH_2-$(PEG_6)_2$-UUUUUU-$(CH_2)_{12}H$

Supplementary Table 2. Laser parameter settings.

Name	Parameter	Name	Parameter
Operating voltage	220V	Power	10 w
Laser Scan Speed	1000 mm/s	Q pulse width	1 μs
Current	1 A	Light-on delay	-100 μs
Frequency	60 KHz	Light-off delay	200 μs
End Delay	200 μs	Corner Delay	100 μs

Supplementary Table 3. Performance comparison between the single-plex chip and the reported works in SARS-CoV-2 detection.

Target (gene)	Assay time (min)	Limit of detection (aM)	Refs.
N	25	666	1
ORF1ab	45	41.5	2
N	30	30	3
S	90	333	4
ORF1ab	120	100	5
N	40	47.44	6
N	60	66.4	7
N	10	400	8
N	30	166	9
N	120	216	10
O and N	60	13.3	11
N	60	200	12
N	45	16.6	13
N	30	470	14
N	2	10	This work

Supplementary Table 4. Performance comparison between the duplex chip and the reported works in IAV and IBV detection.

pathogen	Target (gene)	Assay time (min)	Limit of detection (aM)	Refs.
IAV	M	150	13	15
	No clear	90	4.15	16
	M	60	16.6	17
	No clear	40	72	18
	M1	60	3050	19
	M	11	6800	20
	HA	60	5300	21
IBV	M	150	6.64	15
	HA	60	625	21
	NS1 and NEP	60	3050	19
	No clear	45	9.63	22
	MP	60	460	23
	No clear	120	1.66	24
	NP	90	1.7	25
IAV&IBV	IAV-PB2& IBV-PB1	5	100	This work

Supplementary Table 5. Primer sequences used for amplification of in vitro transcription templates.

Name		Sequence (5'-3')
Forward primer	F1	ATACGACTCACTATAGGGATGTCTGATAATGGACCCCAAAATCA (for SARS-CoV-2, *N* gene)
Reverse primer	R1	TTAGGCCTGAGTTGAGTCAGCA (for SARS-CoV-2, *N* gene)

Supplementary Table 6. The sequences of primers and probes used in RT-PCR kits.

Name		Sequence (5'-3')
Forward primer	F1	TTACAAACATTGGCCGCAAA<br>(for amplifying a segment of the SARS-CoV-2 *N* gene)
	F2	GACAGGAGAAGAACCCAGCA<br>(for amplifying a segment of the IAV *PB2* gene)
	F3	GAAGAAGGAACGGCATCTCT<br>(for amplifying a segment of the IBV *PB1* gene)
Reverse primer	R1	GCGCGACATTCCGAAGAA<br>(for amplifying a segment of the SARS-CoV-2 *N* gene)
	R2	GAGATCTGCATGACCAGGAT<br>(for amplifying a segment of the IAV *PB2* gene)
	R3	TGCAGTCCATCCCATAAGTAT<br>(for amplifying a segment of the IBV *PB1* gene)
**TaqMan Probe**	P1 FAM- ACAATTTGCCCCCAGCGCTTCAG- BHQ1 (*N* gene)<br>P2 FAM-TCCTATTCCACCATGTCACAGCC-BHQ1 (IAV *PB2* gene)<br>P3 FAM-AGGAGTAGCCGCACTAGGGATAA-BHQ1 (IBV *PB1* gene)	

Supplementary Table 7. The sequences of crRNAs used in this work.

Name		Sequence (5'-3')
**crRNA**	crRNA1 for *N* gene	GAUUUAGACUACCCCAAAAACGAAGGGGACUAAAACUCUGAGGGUCCACCAAACGUAAUGCGGG
	crRNA2 for IAV *PB2* gene	NH_2-UUUUUUGAUUUAGACUACCCCAAAAACGAAGGGGACUAAAACUCCUAUUCCACCAUGUCACAGCCAGAGG
	crRNA3 for IBV *PB1* gene	NH_2-UUUUUUGAUUUAGACUACCCCAAAAACGAAGGGGACUAAAACUCCAAUGUUUUUGAUGCCUAGUGCUGCU

Supplementary Table 8. The sequences of targets used in this work.

Name		Sequence (5'-3')
**Target**	SARS-CoV-2 *N* gene	AAUCAGCGAAAUGCACCCCGCAUUACGUUUGGUG GACCCUCAGAUUCAACUGGCAGUAAC
	IAV *PB2* gene	UGAUGGUAUCACCUCUGGCUGUGACAUGGUGGAA UAGGAAUGGCCCAACA
	IBV *PB1* gene	GUGUUGGGAGUAGCAGCACUAGGCAUCAAAAACA UUGGAAACAAGGAAUA
	RSV *M* gene	CUCCACAUACACAGCUGCUGUUCAAUACAAUGUC UUAGAAAAAGACGAUG

Supplementary Table 9. The nucleotide sequences of SARS-CoV-2 *N*, IAV *PB2* and IBV *PB1* genes

Name		Sequence (5'-3')
SARS-CoV-2 gene	*N*	ATGTCTGATAATGGACCCCAAAATCAGCGAAATGCACC<br>CCGCATTACGTTTGGTGGACCCTCAGATTCAACTGGCA<br>GTAACCAGAATGGAGAACGCAGTGGGGCGCGATCAAA<br>ACAACGTCGGCCCCAAGGTTTACCCAATAATACTGCGT<br>CTTGGTTCACCGCTCTCACTCAACATGGCAAGGAAGAC<br>CTTAAATTCCCTCGAGGACAAGGCGTTCCAATTAACAC<br>CAATAGCAGTCCAGATGACCAAATTGGCTACTACCGAA<br>GAGCTACCAGACGAATTCGTGGTGGTGACGGTAAAATG<br>AAAGATCTCAGTCCAAGATGGTATTTCTACTACCTAGGA<br>ACTGGGCCAGAAGCTGGACTTCCCTATGGTGCTAACAA<br>AGACGGCATCATATGGGTTGCAACTGAGGGAGCCTTGA<br>ATACACCAAAAGATCACATTGGCACCCGCAATCCTGCT<br>AACAATGCTGCAATCGTGCTACAACTTCCTCAAGGAAC<br>AACATTGCCAAAAGGCTTCTACGCAGAAGGGAGCAGA<br>GGCGGCAGTCAAGCCTCTTCTCGTTCCTCATCACGTAGT<br>CGCAACAGTTCAAGAAATTCAACTCCAGGCAGCAGTAG<br>GGGAACTTCTCCTGCTAGAATGGCTGGCAATGGCGGTG<br>ATGCTGCTCTTGCTTTGCTGCTGCTTGACAGATTGAACC<br>AGCTTGAGAGCAAAATGTCTGGTAAAGGCCAACAACA<br>ACAAGGCCAAACTGTCACTAAGAAATCTGCTGCTGAGG<br>CTTCTAAGAAGCCTCGGCAAAAACGTACTGCCACTAAA<br>GCATACAATGTAACACAAGCTTTCGGCAGACGTGGTCC<br>AGAACAAACCCAAGGAAATTTTGGGGACCAGGAACTA<br>ATCAGACAAGGAACTGATTACAAACATTGGCCGCAAA(<br>FP)TTGCACAATTTGCCCCCAGCGCTTCAG(TaqMan)CGT<br>TCTTCGGAATGTCGCGC(RP)ATTGGCATGGAAGTCACA<br>CCTTCGGGAACGTGGTTGACCTACACAGGTGCCATCAA<br>ATTGGATGACAAAGATCCAAATTTCAAAGATCAAGTCA<br>TTTTGCTGAATAAGCATATTGACGCATACAAAACATTCC<br>CACCAACAGAGCCTAAAAAGGACAAAAAGAAGAAGG<br>CTGATGAAACTCAAGCCTTACCGCAGAGACAGAAGAA<br>ACAGCAAACTGTGACTCTTCTTCCTGCTGCAGATTTGG<br>ATGATTTCTCCAAACAATTGCAACAATCCATGAGCAGTG<br>CTGACTCAACTCAGGCCTAA
IAV Gene	*PB2*	AGCGAAAGCAGGTCAATTATATTCAATATGGAAAGAATA<br>AAAGAACTAAGAAATCTAATGTCGCAGTCTCGCACCCG<br>CGAGATACTCACAAAAACCACCGTGGACCATATGGCCA<br>TAATCAAGAAGTACACATCAGGAAGACAGGAGAAGAA<br>CCCAGCA(FP)CTTAGGATGAAATGGATGATGGCAATGAA<br>ATATCCAATTACAGCAGACAAGAGGATAACGGAAATGA<br>TTCCTGAGAGAAATGAGCAAGGACAAACTTTATGGAGT

		AAAATGAATGATGCCGGATCAGACCGAGTGATGGTATC<br>ACCTCTGGCTGTGACATGGTGGAATAGGA(TaqMan)ATG<br>GCCCAACAACAAATACAGTTCATTATCCAAAAATCTACA<br>AAACTTATTTTGAAAGAGTCGAAAGGCTAAAGCATGGA<br>ACCTTTGGCCCTGTCCATTTTAGAAACCAAGTCAAAATA<br>CGTCGGAGAGTTGACATAAATCCTGGTCATGCAGATCTC<br>(RP)AGTGCCAAGGAGGCACAGGATGTAATCATGGAAGT<br>TGTTTTCCCTAACGAAGTGGGAGCCAGGATACTAACAT<br>CGGAATCGCAACTAACGATAACCAAAGAGAAGAAAGA<br>AGAACTCCAGGATTGCAAAATTTCTCCTTTGATGGTTGC<br>ATACATGTTGGAGAGAGAACTGGTCCGCAAAACGAGAT<br>TCCTCCCAGTGGCTGGTGGAACAAGCAGTGTGTACATT<br>GAAGTGTTGCATTTGACTCAAGGAACATGCTGGGAACA<br>GATGTATACTCCAGGAGGGGAAGTGAAGAATGATGATG<br>TTGATCAAAGCTTGATTATTGCTGCTAGGAACATAGTGA<br>GAAGAGCTGCAGTATCAGCAGACCCACTAGCATCTTTAT<br>TGGAGATGTGCCACAGCACACAGATTGGTGGAATTAGG<br>ATGGTAGACATCCTTAAGCAGAACCCAACAGAAGAGCA<br>AGCCGTGGGTATATGCAAGGCTGCAATGGGACTGAGAA<br>TTAGCTCATCCTTCAGTTTTGGTGGATTCACATTTAAGA<br>GAACAAGCGGATCATCAGTCAAGAGAGAGGAAGAGGT<br>GCTTACGGGCAATCTTCAAACATTGAAGATAAGAGTGC<br>ATGAGGGATATGAAGAGTTCACAATGGTTGGGAGAAGA<br>GCAACAGCCATACTCAGAAAAGCAACCAGGAGATTGAT<br>TCAGCTGATAGTGAGTGGGAGAGACGAACAGTCGATTG<br>CCGAAGCAATAATTGTGGCCATGGTATTTTCACAAGAG<br>GATTGTATGATAAAAGCAGTTAGAGGTGATCTGAATTTC<br>GTCAATAGGGCGAATCAGCGACTGAATCCTATGCATCA<br>ACTTTTAAGACATTTTCAGAAGGATGCGAAAGTGCTTTT<br>TCAAAATTGGGGAGTTGAACCTATCGACAATGTGATGG<br>GAATGATTGGGATATTGCCCGACATGACTCCAAGCATCG<br>AGATGTCAATGAGAGGAGTGAGAATCAGCAAAATGGG<br>TGTAGATGAGTACTCCAGCACGGAGAGGGTAGTGGTGA<br>GCATTGACCGGTTCTTGAGAGTCCGGGACCAACGAGG<br>AAATGTACTACTGTCTCCCGAGGAGGTCAGTGAAACAC<br>AGGGAACAGAGAAACTGACAATAACTTACTCATCGTCA<br>ATGATGTGGGAGATTAATGGTCCTGAATCAGTGTTGGTC<br>AATACCTATCAATGGATCATCAGAAACTGGGAAACTGTT<br>AAAATTCAGTGGTCCCAGAACCCTACAATGCTATACAAT<br>AAAATGGAATTTGAACCATTTCAGTCTTTAGTACCTAAG<br>GCCATTAGAGGCCAATACAGTGGGTTTGTGAGAACTCT<br>GTTCCAACAAATGAGGGATGTGCTTGGGACATTTGATA<br>CCGCACAGATAATAAAACTTCTTCCCTTCGCAGCCGCTC<br>CACCAAAGCAAAGTAGAATGCAGTTCTCCTCATTTACT

		GTGAATGTGAGGGGATCAGGAATGAGAATACTTGTAAG<br>GGGCAATTCTCCTGTATTCAACTACAACAAGGCCACGA<br>AGAGACTCACAGTTCTCGGAAAGGATGCTGGCACTTTA<br>ACCGAAGACCCAGATGAAGGCACAGCTGGAGTGGAGT<br>CCGCTGTTCTGAGGGGATTCCTCATTCTGGGCAAAGAA<br>GACAGGAGATATGGGCCAGCATTAAGCATCAATGAACT<br>GAGCAACCTTGCGAAAGGAGAGAAGGCTAATGTGCTA<br>ATTGGGCAAGGAGACGTGGTGTTGGTAATGAAACGAA<br>AACGGGACTCTAGCATACTTACTGACAGCCAGACAGCG<br>ACCAAAAGAATTCGGATGGCCATCAATTAGTGTCGAAT<br>AGTTTAAAAACGACCTTGTTTCTACT
IBV Gene	*PB1*	AGCAGAAGCGGAGCTTTAAGATGAATATAAATCCATATT<br>TTCTTTTCATAGATGTACCTATACAGGCAGCAATTTCAA<br>CAACATTCCCATACACCGGTGTTCCCCCTTATTCTCATG<br>GAACGGGAACAGGCTACACAATAGACACCGTGATTAGA<br>ACACACGAGTACTCAAACAAGGGAAAACAATACATTTC<br>TGATGTTACAGGATGTGTAATGGTAGATCCAACAAATGG<br>GCCATTACCCGAAGACAATGAACCGAGTGCCTATGCAC<br>AATTGGATTGTGTTCTGGAGGCTTTGGATAGAATGGATG<br>AAGAACATCCAGGTCTGTTTCAAGCAGGGTCACAGAAT<br>GCCATGGAGGCACTAATGGTCACAACAGTGGACAAATT<br>GACTCAGGGGAGACAGACCTTTGATTGGACGGTGTGTA<br>GAAACCAACCTGCTGCAACGGCACTGAACACAACAAT<br>AACCTCTTTTAGGTTGAATGATTTAAATGGAGCCGACAA<br>GGGTGGATTAGTGCCCTTTTGCCAAGATATCATTGATTC<br>ATTAGACAAACCTGAAATGATTTTCTTCACAGTAAAGA<br>ATATAAAGAAAAAATTGCCTGCTAAAAACAGAAAGGGT<br>TTCCTTATAAAAAGAATACCTATGAAGGTAAAAGACAG<br>AATAACAAGAGTGGAATACATCAAAAGAGCATTATCATT<br>AAACACAATGACTAAAGATGCTGAAAGAGGCAAACTA<br>AAAAGAAGAGCAATTGCCACCGCTGGGATACAAATCAG<br>AGGATTTGTATTAGTAGTTGAAAACTTGGCTAAAAATAT<br>CTGTGAAAATCTAGAGCAAAGTGGTTTACCCGTAGGTG<br>GAAACGAAAAGAAGGCCAAACTATCAAATGCAGTGGC<br>TAAAATGCTCAGTAATTGTCCACCAGGAGGGATCAGTAT<br>GACTGTGACAGGAGACAATACTAAATGGAATGAATGCT<br>TAAATCCAAGAATCTTTTTGGCTATGACTGAAAGAATAA<br>CCAGAGACAGCCCAATTTGGTTCCGGGATTTTTGTAGTA<br>TAGCACCGGTCTTGTTCTCCAATAAAATAGCTAGATTGG<br>GAAAAGGGTTCATGATAACAAGTAAAACAAAAAGACT<br>AAAAGCTCAAATACCTTGTCCCGATCTGTTTAATATACC<br>ATTAGAAAGATATAATGAAGAAACAAGGGCAAAACTGA<br>AAAAGCTAAAACCTTTCTTCAATGAAGAAGGAACGGC<br>ATCTCT(FP)TTCGCCAGGAATGATGATGGGAATGTTTAAT

		ATGCTATCTACAGTATTAGGAGTAGCCGCACTAGGGATA<br>A(TaqMan)AAAACATTGGAAACAAAGAATACTTATGGGA<br>TGGACTGCA(RP)GTCTTCGGATGATTTTGCTCTGTTTGT<br>TAATGCAAAAGATGAAGAGACATGTATGGAAGGAATAA<br>ACGATTTTTACCGAACATGTAAGCTATTGGGAATAAACA<br>TGAGCAAAAAGAAAAGTTACTGTAATGAAACTGGGATG<br>TTTGAATTTACCAGCATGTTTTACAGAGATGGATTTGTAT<br>CTAATTTTGCAATGGAACTCCCTTCATTTGGAGTCGCTG<br>GAGTGAATGAATCAGCAGACATGGCAATAGGAATGACA<br>ATAATAAAGAACAATATGATCAACAATGGGATGGGCCCA<br>GCAACGGCACAAACAGCCATACAATTATTCATAGCTGA<br>CTATAGATACACCTACAAATGCCACAGGGGAGATTCCA<br>AAGTGGAAGGGAAGAGAATGAAAATTATAAAGGAGCT<br>ATGGGAAAACACTAAAGGAAGAGATGGTCTATTAGTAG<br>CAGATGGTGGGCCTAATCTTTACAATTTGAGAAACCTGC<br>ATATTCCAGAAATAATATTAAAATACAACATAATGGACCC<br>TGAGTACAAAGGACGGTTACTGCATCCTCAAAATCCCT<br>TTGTAGGACATTTGTCTATTGAGGGTATCAAAGAAGCAG<br>ATATAACACCTGCACATGGCCCAATAAAGAAAATGGAC<br>TACGATGCGGTATCTGGAACTCATAGTTGGAGAACCAA<br>AAGGAACAGATCTATACTAAACACTGATCAGAGGAACA<br>TGATTCTTGAGGAACAATGCTACGCTAAGTGTTGCAAC<br>CTTTTTGAGGCTTGCTTTAACAGTGCGTCATACAGGAA<br>ACCAGTAGGCCAGCACAGCATGCTTGAAGCTATGGCCC<br>ACAGATTAAGAATGGATGCACGACTGGACTATGAGTCA<br>GGAAGGATGTCAAAAGAGGATTTCGAAAAAGCAATGG<br>CTCACCTTGGTGAGATTGGGTACATGTAAGCTCCGGAA<br>ATGTCTATGGGGTTATTGGTCATCGTTGAATACATGCGGT<br>GCACAAATGATTAAAATGAAAAAAGGCTCGTGTTTCTA<br>CT

Supplementary Table 10. Fabrication parameters of the single-plex chip.

Name	Length	Width	Height
The glass slide	76.2 mm	25.4 mm	1 mm
The microstructure surface	43 mm	22 mm	0.05 mm
The sample reservoir	18 mm (diameter)		0.3 mm
The dam	3 mm	0.09 mm	0.27 mm
The channel	20 mm	3 mm	0.3 mm
The collection zone	6 mm (diameter)		0.3 mm

Supplementary Table 11. Pattern parameters of the duplex chip.

Name	Length	Width	Height
The glass slide	50 mm	50 mm	1 mm
The microstructure surface	As shown in supplementary Fig. 29bII		0.005 mm
The reservoir A	18 mm (diameter)		0.3 mm
The dam 1-2	3 mm	0.9 mm	0.27 mm
Channel 1-2	10 mm	3 mm	0.30 mm
The isolation zone	18 mm	2 mm	0.005 mm
The reservoir B	12 mm (diameter)		0.01 mm
The channel 3	10 mm	3 mm	0.01 mm

Supplementary References

1. Zhang T*, et al.* A paper-based assay for the colorimetric detection of SARS-CoV-2 variants at single-nucleotide resolution. *Nat. Biomed. Eng.* **6**, 957-967 (2022).
2. Yamazaki W, Matsumura Y, Thongchankaew-Seo U, Yamazaki Y, Nagao M. Development of a point-of-care test to detect SARS-CoV-2 from saliva which combines a simple RNA extraction method with colorimetric reverse transcription loop-mediated isothermal amplification detection. *J. Clin. Virol.* **136**, 104760 (2021).
3. Huang Z*, et al.* Electrochemical detection of viral nucleic acids by DNA nanolock-based porous electrode device. *Anal. Chem.* **95**, 16668-16676 (2023).
4. Heo W, Lee K, Park S, Hyun K-A, Jung H-I. Electrochemical biosensor for nucleic acid amplification-free and sensitive detection of severe acute respiratory syndrome coronavirus 2 (SARS-CoV-2) RNA via CRISPR/Cas13a trans-cleavage reaction. *Biosens. Bioelectron.* **201**, 113960 (2022).
5. Wang Z*, et al.* Amplification-Free, Sequencing-Free, Detection of Viral RNAs with Variant Specification by Discrete Nanocounting. *Adv. Funct. Mater.* **35**, 2310157 (2025).
6. Lu R, Wu X, Wan Z, Li Y, Jin X, Zhang C. A novel reverse transcription loop-mediated isothermal amplification method for rapid detection of SARS-CoV-2. *Int. J. Mol. Sci.* **21**, 2826 (2020).
7. Chandrasekaran SS*, et al.* Rapid detection of SARS-CoV-2 RNA in saliva via Cas13. *Nat. Biomed. Eng.* **6**, 944-956 (2022).
8. Yu H*, et al.* Rapid and unamplified detection of SARS-CoV-2 RNA via CRISPR-Cas13a-modified solution-gated graphene transistors. *ACS Sens.* **7**, 3923-3932 (2022).
9. Fozouni P*, et al.* Amplification-free detection of SARS-CoV-2 with CRISPR-Cas13a and mobile phone microscopy. *Cell* **184**, 323-333. e329 (2021).
10. Wang Y*, et al.* CRISPR-Cas13a cascade-based viral RNA assay for detecting SARS-CoV-2 and its mutations in clinical samples. *Sens. Actuators B Chem.* **362**, 131765 (2022).
11. Tian T, Qiu Z, Jiang Y, Zhu D, Zhou X. Exploiting the orthogonal CRISPR-Cas12a/Cas13a trans-cleavage for dual-gene virus detection using a handheld device. *Biosens. Bioelectron.* **196**, 113701 (2022).
12. Zhang D, Zhou Y, Li X, Luan Q. CRISPR/Cas13a-Enhanced Porous Hydrogel Encapsulated Photonic Barcodes for Multiplexed Detection of Virus. *Small* **21**, 2408725 (2025).
13. Mahas A*, et al.* Characterization of a thermostable Cas13 enzyme for one-pot detection of SARS-CoV-2. *PNAS* **119**, e2118260119 (2022).
14. Shan X*, et al.* Nucleic acid amplification-free digital detection method for SARS-CoV-2 RNA based on droplet microfluidics and CRISPR–Cas13a. *Anal. Chem.* **95**, 16489-16495 (2023).
15. Chung H-Y*, et al.* Novel dual multiplex real-time RT-PCR assays for the rapid detection of SARS-CoV-2, influenza A/B, and respiratory syncytial virus using

the BD MAX open system. *Emerg. Microbes Infect.* **10**, 161-166 (2021).
16. Lin M*, et al.* Cas12a Cis-cleavage mediated lateral flow assay enables multiplex and ultra-specific nucleic acid detection. *Nat. Commun.* **16**, 5597 (2025).
17. Zhao X, Zheng X, Yang X, Guo Q, Zhang Y, Lou J. Establishment of a Lateral Flow Dipstick Detection Method for Influenza A Virus Based on CRISPR/Cas12a System. *China CDC Wkly.* **6**, 946 (2024).
18. Ma Y-D, Chen Y-S, Lee G-B. An integrated self-driven microfluidic device for rapid detection of the influenza A (H1N1) virus by reverse transcription loop-mediated isothermal amplification. *Sens. Actuators B Chem.* **296**, 126647 (2019).
19. Ji J*, et al.* AM-DMF-ddRPA: An All-in-One Digital Microfluidic Platform for Rapid and Automatic Digital Nucleic Acid Analysis. *Angew. Chem. Int. Ed.* **64**, e202501913 (2025).
20. Kumar N*, et al.* Label-free peptide nucleic acid biosensor for visual detection of multiple strains of influenza A virus suitable for field applications. *Anal. Chim. Acta.* **1093**, 123-130 (2020).
21. Wang R*, et al.* Rapid detection of multiple respiratory viruses based on microfluidic isothermal amplification and a real-time colorimetric method. *Lab Chip.* **18**, 3507-3515 (2018).
22. Xu D*, et al.* Fast-Flu: RT-RPA-CRISPR/Cas12a assisted one-step platform for rapid influenza B virus detection. *Microbiol. Spectr.* **13**, e00365-00325 (2025).
23. Li R*, et al.* Evaluation of a novel micro/nanofluidic chip platform for the detection of influenza A and B virus in patients with influenza-like illness. *AMB Express* **9**, 77 (2019).
24. Li X, Wang M, Chen J, Lin F, Wang W. Nucleic acid detection and quantitative analysis of influenza virus using exhaled breath condensate. *J. Breath Res.* **15**, 026001 (2021).
25. Ji M*, et al.* Automated multiplex nucleic acid tests for rapid detection of SARS-CoV-2, influenza A and B infection with direct reverse-transcription quantitative PCR (dirRT-qPCR) assay in a centrifugal microfluidic platform. *RSC Adv.* **10**, 34088-34098 (2020).
