# Supplementary material for: A CRISPR-based ‘dam-break drainage’ sensing architecture for rapid, affordable and ultrasensitive pathogen detection
Source: Nat Commun. 2026 Aug 22;17:11194. doi: 10.1038/s41467-026-77042-w (PMC13714288; doi:10.1038/s41467-026-77042-w)
Supplement: Supplementary file 4 [file 41467_2026_77042_MOESM2_ESM.pdf]

Description of Additional Supplementary Files

File Name: Supplementary Movie 1
Description: Real-time detection of serial dilutions of the SARSCoV-2 N gene ranging from 10 aM to 10 pM. The video shows the target concentration-dependent dam-break drainage process on the chip.

File Name: Supplementary Movie 2
Description: Real-time analysis of clinical nasal swab samples with RT–PCR Ct values ranging from 28 to 34. Representative drainage responses of SARS-CoV-2-positive clinical samples are shown.

File Name: Supplementary Movie 3
Description: Effect of the dimension ratio between reservoir B and reservoir A (rB:rA) on directional transport efficiency. The video compares fluid transport behaviors under different reservoir dimension ratios.

File Name: Supplementary Movie 4
Description: Detection outcome for an influenza A virus (IAV)- positive sample. The video demonstrates the target-triggered drainage response in the duplex detection chip.

File Name: Supplementary Movie 5
Description: Detection outcome for an influenza B virus (IBV)- positive sample. The video demonstrates the target-triggered drainage response in the duplex detection chip.

File Name: Supplementary Movie 6
Description: Detection results for mixed IAV-positive/IBV-positive samples and the blank control. The video illustrates the corresponding drainage patterns for dual-positive and negative samples.

File Name: Supplementary Movie 7
Description: Analytical sensitivity assessment using serial dilutions (100 aM–10 pM) of synthetic IAVPB2 and IBV-PB1 RNA targets at equal concentrations. The video demonstrates concentration-dependent drainage responses during duplex detection.

File Name: Supplementary Movie 8
Description: Clinical validation using pooled nasal swab samples comprising ten IAV-positive (Ct = 28–33) and ten IBV-positive (Ct = 28–33) clinical specimens. Representative drainage responses are shown for the pooled clinical samples.
